# The Low Oxidation State Paradigm is More Consistent with XFEL Observations of the S₃ → [S₄] → S₀ Transition in Photosystem II

**DOI:** 10.1002/chem.202501010

**Published:** 2025-06-18

**Authors:** Alireza Ariafard, Matthew Longhurst, Gerhard F. Swiegers, Robert Stranger

**Affiliations:** ^1^ Research School of Chemistry Australian National University Canberra Australia; ^2^ Intelligent Polymer Research Institute University of Wollongong Wollongong Australia

**Keywords:** photosystem II (PSII), oxygen‐evolving complex (OEC), low oxidation state (LOS), paradigm density functional theory (DFT), oxo‐oxyl coupling

## Abstract

Photosynthetic water splitting catalyzed by the Mn_4_CaO_5/6_ cluster in the oxygen‐evolving complex (OEC) of photosystem II (PSII) is crucial for sustaining the supply of oxygen on the Earth. A recent serial femtosecond X‐ray crystallography (XFEL) study has provided unprecedented insights into the structural dynamics of the OEC during the S₃ → [S₄] → S₀ transition, revealing that this process involves a peroxide intermediate formed via oxo‐oxyl radical coupling between O5 and O_x_. However, computational models based on the high oxidation state (HOS) paradigm have failed to explain key XFEL observations, including the apparent loss of O_x_ upon peroxide formation and the largely unchanged Mn4─O5 distance from S₄ to the peroxide intermediate. Here, we apply density functional theory to remodel the S_4_ → S_0_ transition within the low oxidation state (LOS) paradigm and show that this model yields results more consistent with the XFEL observations. Notably, this study demonstrates that the LOS paradigm can support the formation of an oxyl radical species essential for O─O coupling and subsequent O_2_ generation, a capability previously thought to be exclusive to the HOS model. Our findings offer an alternative explanation that complements existing models and broadens our understanding of the OEC mechanism.

## Graphical abstract

Density functional theory (DFT) calculations within the LOS paradigm reproduce key XFEL observations of the S₃ → [S₄] → S₀ transition in PSII. In contrast to the HOS model, the LOS framework explains both the disappearance of O_x_ during O–O bond formation and the unchanged Mn4–O5 distance in the peroxide intermediate.

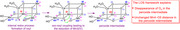

## 1 Introduction

**Figure 1:**
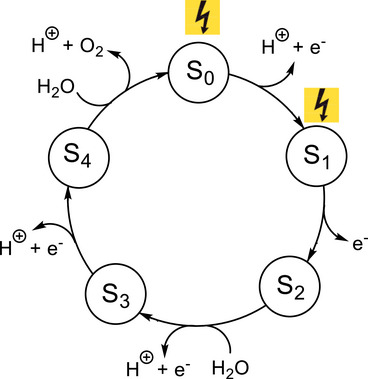
Kok cycle of the water oxidation in the PSII system.

Plants, algae, and cyanobacteria generate essential oxygen (O_2_) through photosynthetic water splitting, a process catalyzed by the Mn_4_CaO_5/6_ cluster within the oxygen‐evolving complex (OEC) embedded in photosystem II (PSII). During this catalytic process, known as the Kok cycle, the OEC progresses through a series of transitions referred to as the “S_i_ states” (i = 0–4) to facilitate the sequential extraction of electrons and protons, ultimately leading to the formation of O_2_, as shown in Figure 1.^[^ ^1^ ^]^ This series includes four relatively stable intermediates (S_0 _− S_3_) and a highly reactive transient state (S_4_), crucial for forming the O─O bond that resets the cycle to the S_0_ state. Electron transfer from the cluster is mediated by a redox‐active tyrosine residue, Y_z_.

The early EPR study of the S_2_ state of the OEC by Dismukes and coworkers established an S = 1/2 ground state consistent with the presence of at least one Mn(III) and one Mn(IV) center in the metal cluster.^[^ ^2^ ^]^ As a result, two oxidation state assignments for the Mn centers in the tetranuclear Mn cluster are possible in the S_2_ state, namely (III, IV, IV, IV) or (III, III, III, IV), with the latter assignment favored by the authors of that paper. Historically, this two‐electron difference between the oxidation state assignments has given rise to two distinct paradigms to describe the sequence of Mn oxidation states in the OEC throughout the redox S state cycle from S_0_ to S_4_, known as the “high oxidation state” (HOS)^[^ ^3^ ^]^ and “low oxidation state” (LOS)^[^ ^4^ ^]^ schemes, respectively. In the HOS model, the Mn oxidation states (Mn1…. Mn4) are (III, IV, III, III) in S_0_, (III, IV, IV, III) in S_1_, (III, IV, IV, IV) in S_2_, and (IV, IV, IV, IV) in S_3_. Conversely, in the LOS model, the proposed oxidation states are (III, III, III, II) in S_0_, (III, III, III, III) or (III, IV, III, II) in S_1_, (III, IV, III, III) in S_2_, and (III, IV, IV, III) in S_3_. Over the last four decades, numerous structural, spectroscopic, and direct electron counting studies have been reported that favor one paradigm over the other. Here the reader is referred to published reviews and key studies that summarize the relevant data in relation to both paradigms.^[^ ^5^, ^6^, ^7^, ^8^ ^]^

The HOS model is currently favored by most workers in the field due to a widely held belief that it provides a better fit with the experimental structural and spectroscopic data,^[^ ^5^ ^]^ rather than definitive evidence ruling out the LOS paradigm.^[^ ^9^ ^]^

Another reason for this preference is the nature of the transient S₄ state, which has been proposed, based on computational studies,^[^ ^3^, ^10^, ^11^ ^]^ to contain an oxyl radical species that is highly reactive toward O─O bond formation, as discussed below. However, no direct experimental evidence currently confirms the presence of such a radical species. Pivotal to this HOS mechanism is the insertion of an additional oxo group, denoted O_x_, during the S₂ → S₃ transition. This O_x_ ligand, first proposed by Siegbahn,^[^ ^12^ ^]^ is coordinated to Mn1 and is suggested to participate in O─O bond formation. This additional oxygen atom in the HOS model was originally postulated as necessary because, without it, Mn1 would remain five‐coordinate in the S_3_ state, an unfavorable geometry for Mn(IV) within the constrained architecture of the OEC. While there are documented cases of stable five‐ and even four‐coordinate Mn(IV) complexes, their stability is a result of the macrocyclic or multidentate ligand system which enforces the low‐coordinate environment.^[^ ^13^ ^]^

It is commonly assumed that an energetically feasible mechanism for O─O bond formation within the LOS paradigm is not viable due to the presence of Mn(III) centers which are prone to further oxidation and thus do not permit the formation of an active oxyl radical species. Although several mechanisms for O─O coupling have been proposed within the LOS paradigm, and some of these even suggest the involvement of an oxyl radical species (vide infra), none have, to date, been computationally verified to establish whether oxyl radical formation is energetically viable.

Before proceeding further, we first provide a brief overview of the mechanistic proposals for O₂ evolution under the LOS paradigm in order to place the present study within the context of previous work.

### 1.1 Overview of Mechanistic Proposals for O₂ Evolution in the LOS Model

The presence of the O_x_ atom in the S₃ state remains a subject of ongoing debate in the literature. Several high‐resolution XFEL studies,^[^ ^14^ ^]^ including those by Ibrahim et al.,^[^ ^15^ ^]^ have reported the appearance of an additional oxo or hydroxo ligand (O_x_) bridging Mn1 and Ca during the S₂ → S₃ transition. However, this interpretation has been contested by Wang and coworkers, who reanalyzed the same XFEL datasets and argued that the electron density attributed to O_x_ can be adequately explained without invoking an additional ligand.^[^ ^16^, ^17^ ^]^ They suggest that the observed features may arise from refinement bias or model overfitting and that structurally consistent models can be achieved in the absence of O_x_. Notably, earlier XFEL structures at 2.25 Å resolution did not show clear evidence for the O_x_ ligand, further contributing to the uncertainty surrounding its presence.^[^ ^18^ ^]^ Several studies, including reinterpretations of spectroscopic data, support the view that the O_x_ ligand may not be present in the S₃ state.^[^ ^4^, ^7^, ^19^ ^]^ Consistent with this perspective, all previously proposed mechanisms for O₂ evolution under the LOS paradigm have been developed based on models that exclude the O_x_ ligand, as briefly outlined below.

In the early LOS model proposed in 1982, well before any X‐ray crystallographic structures of PSII were available and at a time when the oxidation states and electronic structure of the Mn ions in the OEC were still poorly understood, O─O bond formation was suggested to occur between two hydroxide ligands initially bound to Mn(III) centers.^[^ ^2^ ^]^ Upon oxidation to Mn(IV), these ligands were positioned for coupling, leading to O₂ release during the spontaneous decay from the S₄ to the S₀ state. Early studies also recognized that O─O bond formation in the OEC should occur late in the S‐state cycle, most likely during the S₃ to S₀ transition, where the Mn cluster operates predominantly within a LOS framework involving Mn(III) and Mn(IV) ions.^[^ ^20^ ^]^ Subsequent studies proposed two general possibilities for the O─O bond formation step.^[^ ^21^ ^]^ One pathway involves homolytic coupling between two μ‐oxo ligands already bound within the Mn₄CaO₅ core.^[^ ^22^ ^]^ Alternatively, a heterolytic pathway has been suggested, in which a nucleophilic OH⁻, possibly generated by ionization of water bound to Ca^2^⁺, attacks an electrophilic oxygen atom such as a bridging oxyl radical.^[^ ^23^ ^]^

In 2013, Dismukes and coworkers proposed that, following the third flash, Y_z_^•^ is formed, which initiates the deprotonation of W_2_ via the adjacent water network. Subsequently, the Y_z_^•^ hole is transferred to the Mn₄CaO₅ cluster, generating an oxyl radical cation at the O5 site and leading to O─O bond formation through coupling between O5 and the deprotonated W_2_.^[^ ^6^ ^]^

In a recent mechanistic model, based on reanalysis of high‐resolution XFEL crystallographic data, Wang proposed that during the S‐state transitions from S₀ to S₄ within the LOS paradigm, the substrate oxygen atoms remain coordinated to the Mn₄CaO₅ cluster as hydroxide ligands (─OH), with two of them being converted to μ‐oxo character during the S₃ and S₄ states.^[^ ^17^ ^]^ The addition of two water molecules during the S₀ → S₂ transitions, followed by sequential proton removals during the S₂ → S₃ and S₃ → [S₄] transitions, generates two substrate oxygen atoms with μ‐oxo character, most likely O1 and O3, bringing them into close proximity and setting the stage for O─O bond formation in the S₄ state.

However, Wang noted that coupling between O3 and O5 cannot be entirely excluded.

Although valuable mechanistic insights into O₂ evolution within the LOS paradigm have been proposed, our current computational work focuses on addressing the key questions raised by recent XFEL structural observations related to the S₃ → [S₄] → S₀ transition reported by Bhowmick and coworkers,^[^ ^24^, ^25^ ^]^ where the presence of the O_x_ ligand has been observed. Investigation of O₂ evolution pathways under models lacking the O_x_ ligand will be the subject of our future studies.

### 1.2 Mechanism of O─O Bond Formation in the HOS Model and Its Inconsistency with XFEL Observations of the S₃ → [S₄] → S₀ Transition

**Figure 2:**
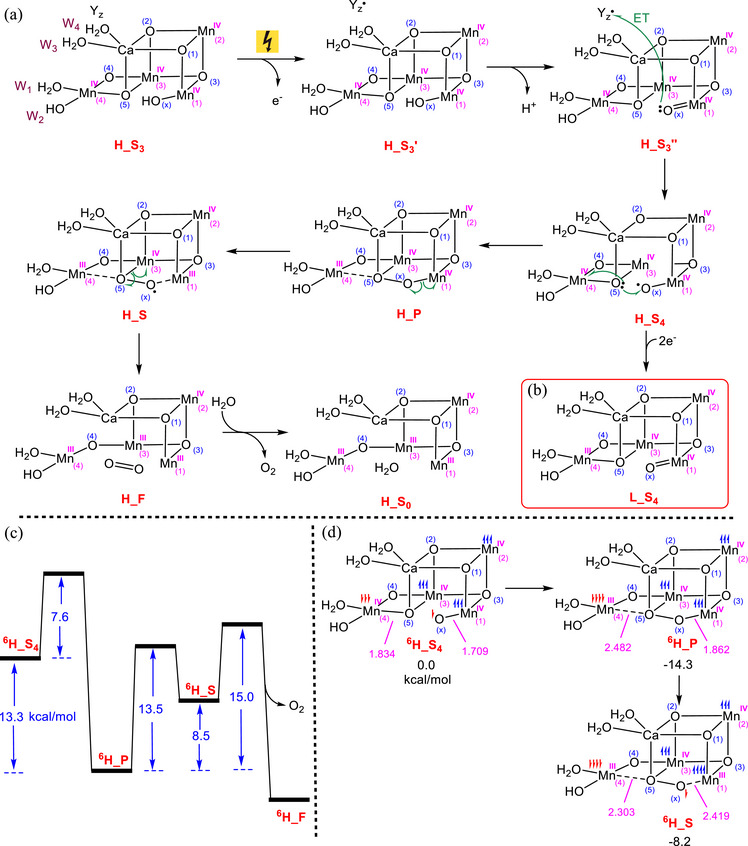
a) The HOS‐oxo‐oxyl mechanism for O_2_ formation, starting from the S_3_ state (**H_S_3_**). b) The proposed S_4_ state in the LOS model, achieved by adding two electrons to the S_4_ state in the HOS. c) Energy profile reported by Song and Wang for the conversion of **H_S_4_** to **H_F** + O_2_, modeled under the HOS paradigm and calculated using QM/MM methodology for the antiferromagnetically coupled state with *S*=5/2 (spin multiplicity of 6). d) Our QM calculations for the key structures **H_S_4_**, **H_P**, and **H_S**. The relative energies are given in kcal/mol and selected bond distances in Å.

Within the HOS paradigm, two principal mechanisms for O─O bond formation have been proposed: the oxo‐oxyl radical coupling mechanism^[^ ^3^, ^12^, ^14^, ^26^, ^27^ ^]^ and the nucleophilic attack mechanism.^[^ ^1^, ^28^ ^]^ The latter involves nucleophilic attack by a water‐derived species on an electrophilic oxygen ligand, which may exist in the form of a Mn(IV)‐oxyl radical or a Mn(V)‐oxo species. However, several computational studies have demonstrated that the oxo‐oxyl coupling mechanism is more energetically favorable.^[^ ^10^, ^26^, ^29^ ^]^ This oxo‐oxyl coupling mechanism, beginning from the S₃ state, is illustrated in Figure 2a. Accordingly, the reaction initiates with the photo‐oxidation process, ultimately resulting in the oxidation of tyrosine (Y_z_) to Y_z_^●^, followed by the deprotonation of the hydroxide ligand (O_x_H) bound to Mn1 to form a Mn(IV)‐oxo species. Subsequently, the Mn_4_CaO_6_ cluster is oxidized by Y_z_^●^, leading to the formation of the highly reactive Mn1(IV)‐oxyl complex H_S_4_. The oxyl fragment in H_S_4_ then participates in O─O bond formation through nucleophilic attack by the bridged oxo ligand O5 to give the peroxide complex H_P, a process known as oxo‐oxyl radical coupling. The O─O coupling reduces Mn4 from an oxidation state of IV in H_S_4_ to III in H_P. Subsequently, the superoxide complex H_S is formed through the homolytic cleavage of the Mn1‐O_x_ bond, resulting in the reduction of Mn1 from an oxidation state of IV in H_P to III in H_S. Previous computational studies have discussed the preference for cleavage of the Mn1─O_x_ bond over the Mn3─O5 bond in superoxide formation.^[^ ^27^ ^]^ This preference is attributed to O_x_ being primarily bonded to Mn1, whereas O5 is bonded to Ca, Mn3, and Mn4, making O_x_ more accessible for cleavage. Finally, the O_2_ molecule is formed by the homolytic cleavage of the Mn3‐O5 bond, reducing Mn3 from an oxidation state of IV in H_S to III in H_F.

In this HOS‐oxo‐oxyl radical coupling mechanism, the critical step involves the formation of the S_4_ structure (**H_S_4_**), which includes the active oxyl site essential for triggering the O─O coupling. Indeed, in the S_3_ state of the HOS paradigm, all the Mn atoms are in an oxidation state of + 4, each harboring three unpaired electrons. These electrons, which reside in the nonbonding t_2_g d‐orbitals, are relatively stable and unlikely to leave the system. Consequently, the oxidation of the Mn_4_CaO_6_ cluster by Y_z_^●^ preferentially occurs at the unbridged oxo ligand (O_x_) bound to Mn1, resulting in the formation of the active oxyl species.

In contrast, in the LOS paradigm, oxidation of the deprotonated intermediate Mn1‐O_x_ by Y_z_^●^ is not expected to generate an oxyl fragment. This is attributed to the presence of two Mn(III) atoms, Mn1 and Mn4, in the S_3_ state, possessing a d^4^ configuration with a single electron in the antibonding e_g_* orbitals. This configuration renders the Mn(III) atoms more susceptible to oxidation compared to the unbridged oxo ligand. Consequently, the S_4_ redox state under the LOS paradigm is generally considered unreactive toward O─O coupling due to the absence of the critical oxyl ligand required to initiate O─O bond formation and, subsequently, O_2_ evolution (Figure 2b). One of the main objectives of this study is to demonstrate how the most stable form of the S_4_ redox state under the LOS paradigm can be activated to generate an oxyl ligand, making it reactive toward O─O coupling.

In 2023, a comprehensive computational study conducted by Song and Wang, modeled within the HOS paradigm, investigated the mechanistic details of the S_3_ to S_0_ transition in the Kok cycle.^[^ ^27^ ^]^ We discuss this recent study below as a representative example, noting that it incorporates data and insights from other computational studies, such as those by Siegbahn et al.,^[^ ^3^, ^10^, ^12^, ^26^ ^]^ Yang et al.,^[^ ^26f^ ^]^ Sun et al.,^[^ ^11^ ^]^ and O'Malley et al.,^[^ ^26c,d^ ^]^ which have significantly contributed to understanding O_2_ formation under the HOS paradigm. Figure 2c presents the energy profile associated with the transformation from S_4_ to S_0_, as reported by Song and Wang,^[^ ^27^ ^]^ utilizing QM/MM methodology with the B3LYP functional used for the QM portion. Additionally, in 2023, Bhowmick et al. used serial femtosecond X‐ray crystallography to capture room‐temperature snapshots of PSII for the same S_3_ to S_0_ transition.^[^ ^24^, ^25^ ^]^ They observed that the S_4_ state forms around 730 µs and suggested that O─O bond formation is completed by 1200 µs, with the release of molecular oxygen (O_2_) likely concluding by 2000 µs.

However, a number of inconsistencies emerge between the computational results based on the HOS model and the experimental findings of Bhowmick et al. Notably, based on omit map densities, Bhowmick et al. showed that upon completion of O─O coupling and prior to O_2_ evolution at 1200 µs, the electron density of O_x_ falls below the 2.5σ threshold.^[^ ^24^ ^]^ This indicates that O_x_ is predominantly displaced from its original position, while O5 still retains significant density. This observation might be explained by the fact that, upon formation of the peroxide intermediate, the Mn1─O_x_ bond is significantly weakened, resulting in increased positional flexibility of O_x_ and, consequently, uncertainty in accurately determining its location.

The asymmetric omission of O_x_ compared to O5 cannot be adequately explained by computational modeling within the HOS paradigm.^[^ ^3^, ^10^, ^11^, ^26^, ^27^ ^]^ As depicted in Figure 2a, in the peroxide complex **H_P**, O_x_ still binds strongly to Mn1, and its omission would only be expected upon the formation of the superoxide complex **H_S,** where Mn1 has been reduced to the oxidation state (III). However, according to the calculations by Song and Wang,^[^ ^27^ ^]^ the superoxide complex **^6^H_S** lies 8.5 kcal/mol higher in energy than the peroxide complex **^6^H_P** (Figure 2c). This energy difference implies that the concentration of **^6^H_S** should be approximately 6 × 10^7^ times smaller than that of **^6^H_P**, as calculated using the fundamental thermodynamic equation Δ*G* = −*RT* Ln *K*. Consequently, since the population of **^6^H_S** is estimated to be negligible, it is unlikely to be detected experimentally. Based on the computational results shown in Figure 2c, it is expected that the omission of density for both O_x_ and O5 should be essentially symmetrical, which directly contradicts the experimental observations of Bhowmick et al.^[^ ^24^ ^]^

To confirm that the structure **^6^H_S** has a negligible concentration and is not experimentally detectable, we calculated the energy of this intermediate using QM and implicit solvation models at the IEFPCM/B3LYP‐D3/6–311 + G(2d, p), SDD//IEFPCM/B3LYP‐D3/6–31G(d), SDD level of theory (Figure 2d). These calculations were performed on a 200 + atom model, based on the exact model recently utilized by Siegbahn and coworkers,^[^ ^26a^ ^]^ with an antiferromagnetically coupled *S* = 5/2 state (corresponding to a spin multiplicity of 6), as supported by other studies.^[^ ^5^, ^27^, ^30^ ^]^ Our results indicate that, consistent with the findings reported by Song and Wang, **^6^H_S** lies significantly higher in energy than **^6^H_P**, with an energy difference of 6.1 kcal/mol. This further confirms that the concentration of **H_S** should be too low to be detectable experimentally.

**Figure 3:**
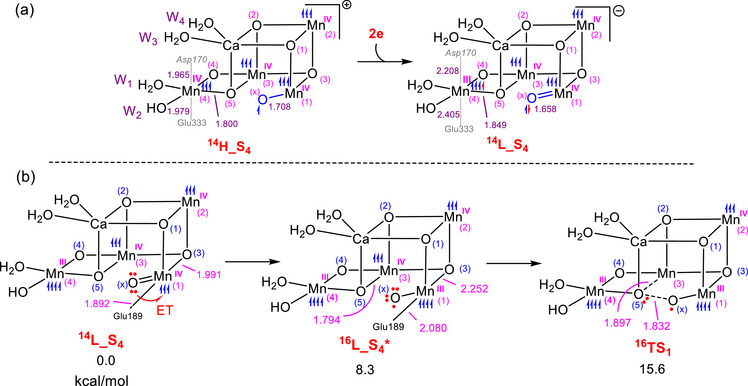
a) Changes in important structural parameters upon the addition of two electrons to the S_4_ state in the HOS model, leading to the formation of the equivalent S_4_ state in the LOS model. b) Generation of the oxyl entity in the LOS model, initiated from the S_4_ state where all Mn atoms are ferromagnetically coupled, followed by oxo‐oxyl coupling via the transition structure **^16^TS_1_**. The relative free energies are given in kcal/mol and selected bond distances in Å.

The XFEL study by Bhowmick et al.^[^ ^24^, ^25^ ^]^ highlights another discrepancy between the experimental observations and theoretical models based on the HOS paradigm.^[^ ^24^ ^]^ Their analysis of population‐averaged data, based on structural refinements that incorporate population heterogeneity and experimental uncertainties, indicates that the Mn4─O5 bond distance changes negligibly between 730 µs and 1200 µs, measuring 2.09 ± 0.10 Å and 2.11 ± 0.14 Å, respectively; however, a notable increase is observed at 2000 µs, reaching 2.21 ± 0.15 Å (see Figure 3 and Extended Data Table 4 ref. [24]). These refined distances provide a reliable basis for observing trends across the S_3_→S_0_ transition. Although these values should not be interpreted as absolute due to the challenges of precisely resolving Mn─O distances in XFEL studies, which stem from the diffuse electron densities of oxygen atoms near electron‐rich Mn centers, the observed trend in bond distances suggests that the Mn4─O5 bond remains largely unchanged from the S₄ state to the peroxide complex.

In contrast, computational models based on the HOS paradigm fail to replicate these experimental trends. As shown in Figure 2d, the calculated Mn4‐O5 bond distance in the S_4_ state intermediate **^6^H_S_4_** is 1.834 Å, significantly shorter than the 2.482 Å predicted for the peroxide intermediate **^6^H_P**. This discrepancy of approximately 0.65 Å is attributed to the reduction of Mn4 from an oxidation state of + 4 to + 3 during O─O coupling, which results in an elongation of the Mn4‐O5 bond in **^6^H_P** due to the Jahn‐Teller effect. The inability of HOS‐based models to reproduce the experimentally observed stability of the Mn4‐O5 bond distance prior to 2000 µs, as reported by Bhowmick et al.,^[^ ^24^ ^]^ suggests that this paradigm may not fully capture the structural and electronic changes in the OEC during the S_3_→S_0_ transition.

From the above comparison, it is evident that computational studies within the HOS paradigm struggle to account for key experimental observations in PSII related to the S_3_ → S_4_ → S_0_ transition. Given these inconsistencies, it is of interest to consider whether the alternative LOS model can deliver results that align more closely with the observed XFEL data. On this matter, it is important to note that the LOS paradigm is supported by various experimental data, including XRD/XFEL studies, spectroscopy (XAS, EXAFS, EPR), and photo‐assembly studies (for a summary, see ref. [7]). Furthermore, a recent study by Dismukes et al. using electron‐counting methods provides additional support for the LOS model in PSII O₂ evolution.^[^ ^8^ ^]^ This conclusion aligns with earlier works by Dismukes et al., which consistently support the LOS paradigm based on various approaches, including mechanistic insights and kinetic studies of water oxidation.^[^ ^19^, ^31^ ^]^ Separately, substrate water exchange data, which cannot be explained within the HOS model, is easily rationalized in the LOS paradigm.^[^ ^32^ ^]^ Additionally, the recent re‐refinement of XFEL structures in the S_3_ state by Wang shows that both Mn1 and Mn4 are in the + 3 oxidation state, providing further structural evidence for the LOS paradigm.^[^ ^17^ ^]^

As well‐documented and discussed above, the formation of an oxyl species (or a radical) is a prerequisite in the S_4_ redox state for O─O bond coupling to occur with a low activation barrier. While this requirement is readily met in the HOS paradigm, we aim to demonstrate that it is also achievable in the LOS equivalent. To accomplish this, we used the same 200 + atom S_4_ structure model discussed above for O─O coupling in the HOS model and added two electrons to it to obtain the equivalent S_4_ state in the LOS paradigm.

Assuming that the O─O bond formation arises through the generally accepted oxyl‐oxo coupling mechanism depicted in Figure 2a, we undertook computational modeling to assess whether this mechanism, or similar one, is viable within the LOS paradigm. In this study we demonstrate that the LOS model is not only capable of O─O bond formation via an oxyl‐oxo coupling mechanism but also generates O_2_ with a barrier comparable to that of the HOS model. Furthermore, we will show that our results using the LOS model align more closely with the experimental findings of Bhowmick et al.^[^ ^24^, ^25^ ^]^ compared to those modeled under the HOS paradigm. This study demonstrates that oxyl formation in the S₄ state is feasible within the LOS paradigm, providing critical support for the possibility of O₂ generation in PSII under these conditions.

## 2 Results and discussion

We begin our investigation by examining how an S_4_ state species in the LOS model can be activated for O─O coupling. To achieve this, we added two electrons to the fully ferromagnetic S_4_ state species in the HOS model, **^14^H_S_4_**, resulting in **^14^L_S4**. This new species retains a spin multiplicity of 14, as the additional electrons were added to Mn4 in the spin‐up orientation and to O_x_ in the spin‐down orientation (see Figure 3a). Frontier orbital analysis of **^14^H_S_4_** demonstrates why the two added electrons should occupy Mn4 and O_x_. According to our calculations, the first two unoccupied orbitals (LUMO and LUMO + 1) in **^14^H_S_4_**, ordered by energy, are primarily localized on O_x_ (at ‐4.39 eV) and on the Mn4 d_z_^2^ orbital (at ‐3.96 eV), making these two orbitals preferentially occupied upon addition of the electrons (Figure S4).

In this study, we performed calculations on all structures at the IEFPCM/B3LYP‐D3/6–311 + G(2d, p), SDD//IEFPCM/B3LYP‐D3/6–31G(d), SDD level of theory. As shown in Figure 3a, the electron addition results in the elongation of both the Mn4‐Asp170 and Mn4‐Glu333 bonds, while the Mn1‐O_x_ bond is shortened. The bond elongations result from the reduction of Mn4(IV) to Mn4(III), which places a single electron in the Mn4 e_g_* orbital and triggers the Jahn‐Teller effect. On the other hand, the shortening of the Mn1‐O_x_ bond results from the O_x_ atom transitioning from oxyl in **^14^H_S_4_** to oxo in **^14^L_S_4_**. The Natural Localized Molecular Orbital (NLMO) analysis for **^14^L_S_4_** indicates that, while the α‐spin electrons of the lone pairs with p‐orbital character on O_x_ are primarily nonbonding, the β‐spin electrons of these lone pairs exhibit significant interaction with the Mn1 d_π_ orbitals (Figure S1). This suggests that Mn1(IV)‐oxo is the most accurate description for the character of this species.

An analysis of the frontier orbitals of **^14^L_S_4_** reveals that the O_x_ atom makes a significant contribution to the HOMO orbital, while all Mn atoms with a formal oxidation state of + 4, namely Mn1, Mn2, and Mn3, are the main components of the LUMO and LUMO + 1 orbitals (Figure S2). We also found that the HOMO, with an energy of ‐5.04 eV, is relatively close in energy to the LUMO and LUMO + 1 orbitals, at ‐2.10 eV and ‐1.94 eV, respectively. These small energy differences between the HOMO and LUMO, as well as between the HOMO and LUMO + 1 levels, suggest that an internal redox process may be feasible. This process could involve the transfer of an electron from O_x_ to one of the Mn(IV) atoms, resulting in its reduction to Mn(III). To confirm this hypothesis, we optimized the structure of **L_S_4_** with a spin multiplicity of 16 and found that the resultant complex, ^16^L_S_4_^*^, lies only 8.3 kcal/mol higher in energy than **^14^L_S_4_** (Figure 3b). This geometry optimization on a surface with a multiplicity of 16 prompts the transfer of one electron from the O_x_ ligand to Mn1, thereby reducing Mn1 with an oxidation state of + 4 to Mn(III) in ^16^L_S_4_^*^. Crucially, the electron transfer from O_x_ to the Mn1(IV) center leads to the formation of an oxyl species, which is essential for initiating the O─O bond coupling process.

The reduction of Mn1(IV) to Mn1(III) results in significant elongation of both the Mn1‐O3 and Mn1‐Glu189 bonds in ^16^L_S_4_^*^, as shown in Figure 3b, due to the Jahn‐Teller effect. The internal redox process is further supported by an increase in spin density for the Mn1 and O_x_ atoms, increasing from 2.725 and 0.365 in **^14^L_S_4_** to 4.075 and 0.692 in ^16^L_S_4_^*^, respectively.

Subsequently, we investigated the reactivity of ^16^L_S_4_^*^ with an oxyl ligand toward O─O coupling. This investigation enabled us to identify the transition structure **^16^TS_1_**, which has a relative free energy of 15.6 kcal/mol. This result clearly shows that oxo‐oxyl coupling is achievable in the LOS, similar to the HOS model, challenging previous assumptions that O_2_ generation in the LOS model was not viable.^[^ ^5^, ^33^ ^]^

There is a difference, however, between the oxyl‐oxo coupling in the HOS model compared to the LOS arrangement. In the HOS model, the oxyl‐oxo coupling leads to the reduction of Mn4 from an oxidation state of + 4 to + 3 (Figure 2a), whereas in the LOS model, Mn3 undergoes this reduction, as shown below. This difference arises because Mn4 in the LOS model is already in an oxidation state of + 3 and is thus less susceptible to reduction compared to Mn3, which is in an oxidation state of + 4.

### 2.1 Impact of Antiferromagnetic Couplings on the Key Species L_S_4_, L_S_4_^*^, and TS_1_

In the above discussion, we have examined the energetics of key species (**^14^L_S_4_**, ^16^L_S_4_^*^, and **^16^TS_1_**) involved in the oxo‐oxyl coupling within the LOS system, starting from an S_4_ state where all Mn atoms and the oxyl ligand are ferromagnetically coupled. In this subsection, our objective is to explore the impact of antiferromagnetic coupling, using the broken‐symmetry methodology^[^ ^34^ ^]^ for these key species.

**Figure 4:**
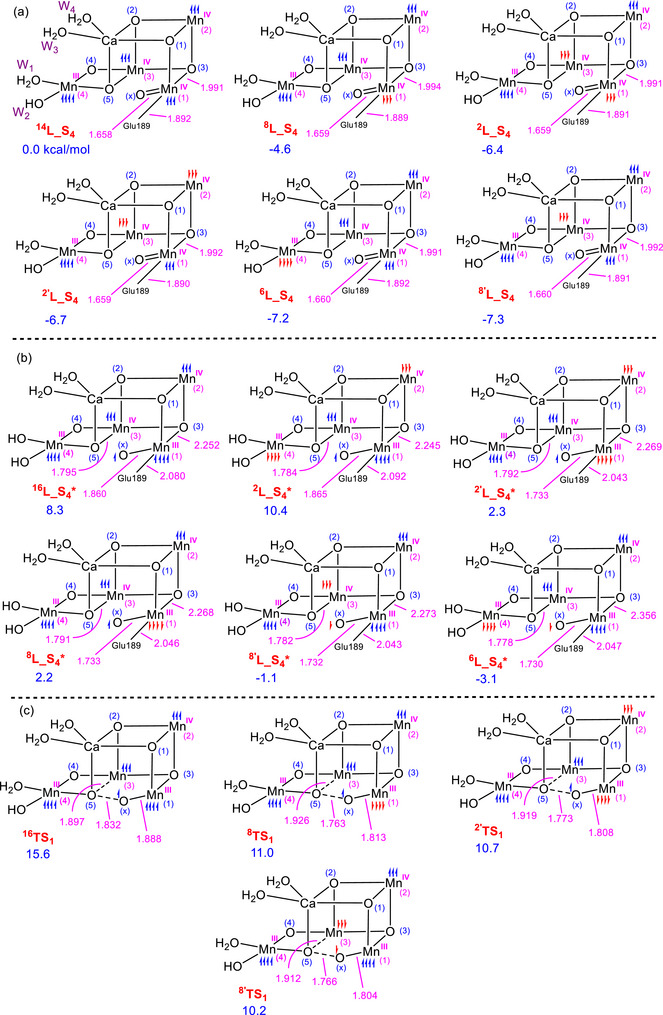
The relative free energies for the LOS species of (a) **L_S_4_**, (b) L_S_4_*, and (c) **TS_1_** with different multiplicities arising from antiferromagnetic coupling. The relative free energies are given in kcal/mol and selected bond distances in Å.

As seen from Figure 4a, complexes **L_S_4_**, in which Mn atoms are coupled antiferromagnetically, exhibit greater stability compared to **^14^L_S_4_**. Furthermore, the relative stability of the **L_S_4_** species is minimally affected by the different multiplicities of the complex in cases involving antiferromagnetic coupling. Among the complexes investigated in this study, complex **^8′^L_S_4_** is slightly more stable than the other antiferromagnetic species.

The stability of the oxyl intermediates, L_S_4_^*^, is determined by the alignment of electrons on the oxyl ligand and the Mn1 atom (Figure 4b). Our calculations indicate that the stability is greatest when the oxyl and Mn1 are coupled antiferromagnetically. For example, the complexes ^8′^L_S_4_^*^ and ^6^L_S_4_^*^, which exhibit antiferromagnetic coupling, are much more stable than the complexes ^2^L_S_4_^*^ and ^16^L_S_4_^*^, where the electrons on Mn1 are ferromagnetically coupled with the oxyl ligand. This result is consistent with previous computational studies in this field.^[^ ^27^, ^33^, ^36^ ^]^

The NLMO calculations suggest that when the oxyl and Mn1 are coupled ferromagnetically, the single electron on the oxyl ligand remains in a nonbonding orbital. Conversely, when they are coupled antiferromagnetically, the single electron on the oxyl ligand engages in a π interaction with a t_2g_ orbital on Mn1, thereby stabilizing the system (Figure S3). The second‐order perturbation energy (E^2^) for such an interaction is about 19 kcal/mol, a value significant enough to cause complexes ^8′^L_S_4_^*^ and ^6^L_S_4_^*^ to be more stable than ^2^L_S_4_^*^ and ^16^L_S_4_^*^. The presence of such a π interaction is corroborated by the shorter Mn1‐O_x_ bond distances in complexes ^8′^L_S_4_^*^ and ^6^L_S_4_^*^ compared to ^2^L_S_4_^*^ and ^16^L_S_4_^*^ (Figure 4b).

Among the low‐energy intermediates shown in Figure 4b, the most stable, ^6^L‐S_4_^*^, is not reactive toward O─O coupling. This is because, in this intermediate, the O_x_ has a spin‐down electron, while Mn3, which is reduced during the O─O coupling, has three spin‐up electrons. This forces the transfer of a spin‐down electron to Mn3 upon completion of the O─O coupling, creating an unfavorable low‐spin electron configuration (t_2g_^4^) on this atom and resulting in a high activation barrier for the O─O coupling.^[^ ^36^ ^]^

In contrast, the three low‐energy intermediates ^2′^L_S_4_^*^, ^8^L_S_4_^*^, and ^8′^L_S_4_^*^ are all reactive toward the O─O coupling, as both Mn3 and the oxyl group have the same spin alignment, either spin‐up or spin‐down. The reactivity of these intermediates is further corroborated by the identification of three transition structures, **^2′^TS_1_**, **^8^TS_1_**, and **^8′^TS_1_**, all of which are significantly lower in energy than **^16^TS_1_**. This result indicates that antiferromagnetic coupling has a stabilizing effect on the O─O coupling transition structures.

### 2.2 Detailed Free Energy Profile for O_2_ Evolution

**Figure 5:**
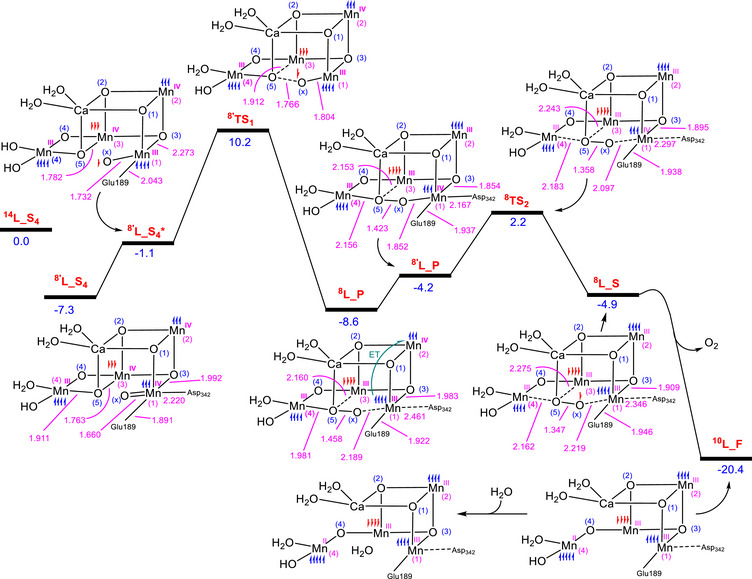
The free energy profile for O_2_ evolution starting from the S_4_ structure in the LOS model. The relative free energies are given in kcal/mol and selected bond distances in Å.

With the understanding that **^8′^TS_1_** is lower in energy than other transition structures, we investigated the full energy profile within the LOS paradigm for O_2_ evolution on this surface (spin state S = 7/2), where **^8′^TS_1_** serves as the transition structure responsible for O─O coupling, starting from the S_4_ state species **^8′^L_S_4_** and ending with the S_0_ state species **^10^L_F** (Figure 5). As shown in Figure 5, the reaction begins with an electron transfer from O_x_ to Mn1 in **^8′^L‐S_4_**, producing ^8′^L_S_4_^*^. This is followed by O─O coupling through the transition structure **^8′^TS_1_**, leading to the formation of the peroxide intermediate **^8^L_P** in a slightly exergonic manner; **^8^L_P** is calculated to be 1.3 kcal/mol lower in energy than **^8′^L‐S_4_**.

An intriguing aspect of the O─O bond coupling in the LOS system is that once the peroxide intermediate **^8^L_P** forms, the single electron in the Mn1 e_g_* orbital shifts its orientation from the O3‐Mn1‐Glu189 axis to the O_x_‐Mn1‐Asp342 axis. This shift results in the shortening of the Mn1‐O3 and Mn1‐Glu189 bonds, while the Mn1‐O_x_ and Mn1‐Asp342 bonds lengthen upon moving from ^8′^L_S_4_^*^ to **^8^L_P**. This structural change can be attributed to the weaker σ‐donating characteristic of the peroxide ligand compared to the oxyl ligand. This favors the Jahn‐Teller effect occurring along the O_x_‐Mn1‐Asp342 axis rather than the O3‐Mn1‐Glu189 axis.

As discussed above, O─O bond coupling in the LOS model results in the reduction of Mn3 from an oxidation state of + 4 to + 3. This reduction places an electron in the e_g_* orbital of Mn3, triggering a Jahn‐Teller effect, which is evidenced by the elongation of the Mn3‐O5 bond from 1.763 Å in **^8′^L_S_4_** to 2.160 Å in **^8^L_P**. As a result, in contrast to the HOS model, the O─O coupling does not affect the electronic structure of the Mn4 atom, and thus the Mn4‐O5 bond distance remains largely unchanged during this process, consistent with the study of Bhowmick et al.^[^ ^24^ ^]^

Once formed, the peroxide ligand is expected to be oxidized by a Mn(IV) atom, resulting in the formation of a superoxide complex. The only Mn(IV) center available in complex **^8^L_P** is Mn2. Our attempts to directly transfer an electron from the peroxide ligand to Mn2 resulted in an electron being transferred from Mn1 to Mn2 instead, producing a new peroxide complex, **^8′^L_P**, which is 3.4 kcal/mol less stable than **^8^L_P**. This electron transfer results in the complete emptying of the e_g_* orbital on Mn1, which allows the Mn1‐O_x_ and Mn1‐Asp342 bonds to shorten to 1.852 Å and 2.167 Å, respectively. The resultant **^8′^L_P** is now reactive toward generation of the superoxide complex **^8^L_S** through the transition structure **^8^TS_2_**. The resultant superoxide complex is calculated to be 3.7 kcal/mol higher in energy than the peroxide complex **^8^L_P**. Since all Mn atoms in **^8^L_S** are in the oxidation state of + 3 with a t_2g_^3^e_g_*^[^ ^1^ ^]^ electron configuration, the superoxide ligand coordinates weakly to Mn4, Mn3, and Mn1 as a consequence of the Jahn‐Teller effect. This weak coordination renders the superoxide ligand highly reactive toward the reduction of Mn4 from the + 3 to + 2 oxidation state, prompting its departure from the system as O_2_ and resulting in the formation of complex, **^10^L_F**. This new complex **^10^L_F**, which features a cavity between Mn1, Mn3, and Mn4 suitable for accepting a water molecule, is calculated to form in a highly exergonic reaction and corresponds to the start of the water oxidation cycle, that is, S_0_.

### 2.3 Alignment with Experimental Thermodynamic and Kinetic Data

Notably, the S_4_ → S_0_ transition has been reported as highly exergonic, with an equilibrium constant estimated between 1.0 × 10⁷ and 1.0 × 10¹⁵, based on oxygen‐water isotope exchange experiments and other studies.^[^ ^37^ ^]^ Given this estimated equilibrium constant, the Gibbs free energy change (Δ*G*) for the S_4_ → S_0_ transition is expected to fall within the range of ‐10 kcal/mol to ‐20 kcal/mol. This aligns with our computational results, as Δ*G* based on our calculations is found to be ‐13.1 kcal/mol, calculated from the energy difference between **^10^L_F** and **^8′^L_S_4_** (Figure 5).

In terms of kinetics, the S_3_ → S_0_ transition occurs within a timescale of approximately 2 ms, as observed through methods such as O₂ release kinetics, Fourier‐transform infrared (FTIR) spectroscopy, photothermal beam deflection, YZ^•^ reduction, time‐resolved X‐ray spectroscopy, and time‐resolved O₂ polarography.^[^ ^24^, ^26^, ^38^ ^]^ From these observations, the Gibbs free energy of activation (Δ*G* ^‡^) for the rate‐limiting step of the S_3_ → S_0_ transition is inferred to be around 14 kcal/mol. This implies that the activation energy for the O─O coupling step should not exceed 14 kcal/mol. However, our calculations, based on the free energy profile presented in Figure 5, do not align with this threshold, instead predicting an activation free energy of 17.5 kcal/mol (the energy difference between **^8′^L_S_4_** and **^8′^TS_1_**). This discrepancy naturally raises the question as to whether an alternative pathway for O─O coupling exists in the LOS paradigm, one that proceeds with a barrier lower than 14 kcal/mol. In the following section, we explore this alternative pathway in detail.

### 2.4 A Lower‐Energy Pathway for O─O Coupling and the Role of His337

One reason for the higher activation energy of O─O coupling in the pathway outlined in Figure 5 is the substantial energy gap between the oxo complex **^8′^L_S_4_** and the oxyl complex ^8′^L_S_4_*, with a difference of 6.2 kcal/mol. Reducing this energy gap is expected to facilitate the O─O coupling process. In this subsection, we demonstrate the crucial role of His337 in lowering this energy gap, enabling O─O coupling to proceed with an overall energy barrier of less than 14 kcal/mol, consistent with experimental studies.^[^ ^24^, ^26^, ^38^ ^]^ As pointed out in an earlier study by us, the unique orientation of the His337 residue close to the μ_3_‐oxo bridge (O3) in the PSII crystal structures strongly suggests that an H‐bonding interaction, even proton transfer, is likely between this residue and the O3 bridge.^[^ ^39^ ^]^

**Figure 6:**
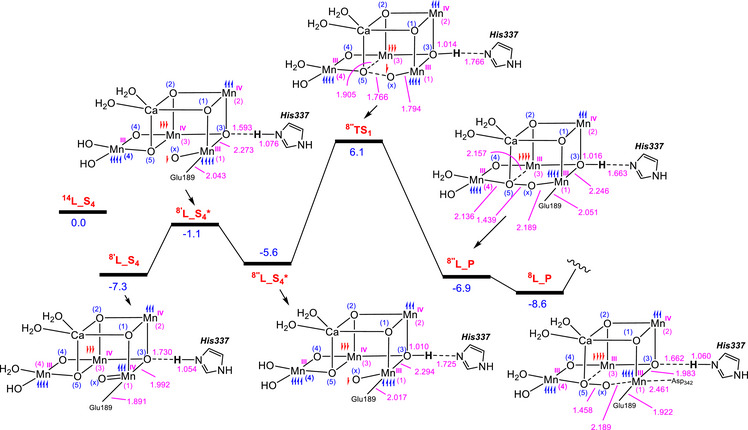
Free energy profile for O─O bond formation in the LOS model via the oxo‐oxyl coupling mechanism, facilitated by His337. Relative free energies are shown in kcal/mol, with key bond distances in Å.

As discussed above, an internal redox process initiates the formation of the oxyl complex ^8′^L_S_4_* through electron transfer from the oxo ligand to Mn1 in **^8′^L_S_4_**, reducing the oxidation state of Mn1 from + 4 to + 3. The Jahn‐Teller effect associated with this oxidation state change weakens the Mn1─O3 bond, polarizing it toward O3 and increasing the electron density on O3. This enhanced electron density raises the basicity of O3 in ^8′^L_S_4_* compared to **^8′^L_S_4_**. Evidence for this claim is provided by the shortened donor‐acceptor distance in the hydrogen bond between O3 and His337, which decreases from 1.730 Å in **^8′^L_S_4_** to 1.593 Å in ^8′^L_S_4_* (Figure 6). As a result, O3 can accept a proton from His337, forming a more stable oxyl complex, ^8′′^L_S_4_*, with a relative free energy of ‐5.6 kcal/mol. This proton transfer reduces the energy difference between the oxo complex **^8′^L_S_4_** and the oxyl complex ^8′′^L_S_4_* to 1.7 kcal/mol, facilitating O─O coupling in ^8′′^L_S_4_* via transition structure **^8′′^TS_1_**, with an overall activation free energy of 13.4 kcal/mol (Figure 6). This transition structure leads to the formation of the peroxide complex **^8′′^L_P**, which further stabilizes through a proton transfer back from O3 to His337, resulting in the more stable peroxide complex **^8^L_P**.

### 2.5 Alignment with Experimental XFEL Data

**Figure 7:**
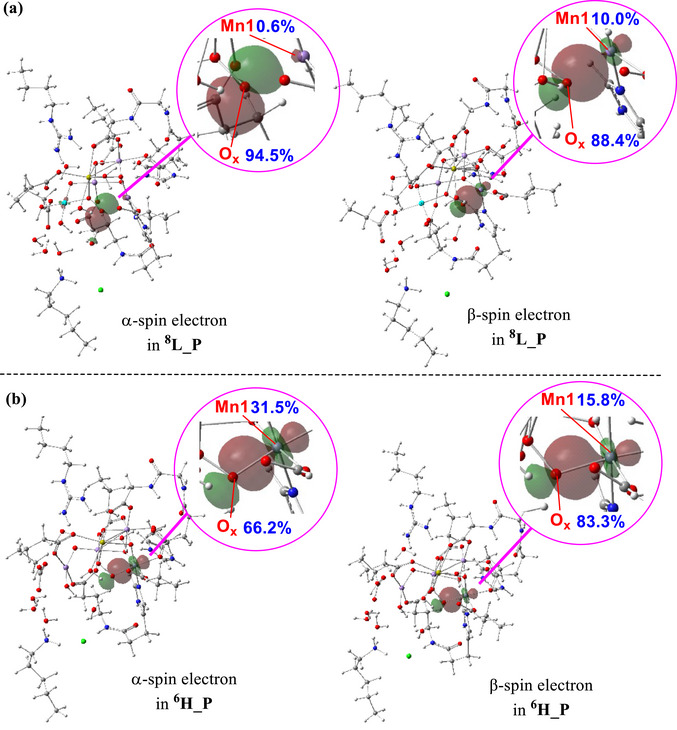
Spatial plots illustrating the NLMO orbital for interaction between Mn1 and O_x_ in the peroxide intermediates a) **^8^L_P** and b) **^6^H_P**.

As shown in Figure 5, the structural changes during O─O coupling under the LOS paradigm align well with the femtosecond X‐ray crystallography findings of Bhowmick et al.^[^ ^24^ ^]^ The intermediates **^8′^L_S_4_** and **^8^L_P**, having the lowest energies, are therefore anticipated to be experimentally detectable. As discussed in the Introduction, the experimental XFEL data indicate a substantial omission of O_x_ upon peroxide formation at 1200 µs. Notably, the long Mn1─O_x_ distance of 2.189 Å in the peroxide intermediate **^8^L_P**, resulting from the Jahn‐Teller effect, suggests a substantially reduced electron density between Mn1 and O_x_, helping to explain the observed omission of O_x_ at this time point. To further confirm this reduced electron density, we conducted an NLMO analysis on the Mn1─O_x_ interaction in **^8^L_P** (Figure 7). The analysis revealed that the α‐spin electron is fully localized on O_x_, while the β‐spin electron is primarily on O_x_ with only a 10% contribution from Mn1. This significantly reduced electron density between O_x_ and Mn1 in **^8^L_P**, which can increase the positional flexibility of O_x_, accounts for the difficulty in detecting O_x_ in the XFEL experiment upon O─O bond formation.

In contrast, the NLMO analysis for the Mn1─O_x_ bond in the peroxide complex **^6^H_P** formed under the HOS paradigm reveals a very different result. As shown in Figure 7, Mn1 contributes significantly to the bond, with a 31% contribution for the α‐spin electron and a 16% contribution for the β‐spin electron. Consequently, there is a relatively high electron density between Mn1 and O_x_ in intermediate **^6^H_P**, suggesting that O_x_ should be detectable once the peroxide intermediate is formed. This result, however, is not consistent with the experimental data reported by Bhowmick et al.^[^ ^24^ ^]^

Another consistency between experiment and theory when considering the LOS paradigm is the change in the Mn4‐O5 bond distance. As discussed in the Introduction, structural refinements reported by Bhowmick et al.^[^ ^24^ ^]^ indicate that the Mn4─O5 bond distance remains relatively unchanged in both the intermediate formed after the oxidation of the OEC cluster by Y^●^ (S_4_ state) and the peroxide complex.^[^ ^24^ ^]^ However, in the HOS model, the significant change in the Mn4‐O5 bond distance from 1.834 Å in **^6^H_S_4_** to 2.482 Å in **^6^H_P** (Figure 2d) is inconsistent with experimental observations. In contrast, the analogous changes in the LOS model are not significant, increasing only slightly from 1.911 Å in **^8′^L_S_4_** to 1.981 Å in **^8^L_P**.

Further agreement between the LOS model and the XFEL experimental data relates to the delay observed in the O─O bond formation process. The XFEL data indicate a significant delay between the initial onset of O─O bond formation, observed at 500–730 µs, and the onset of O₂ release at 1200 µs.^[^ ^24^ ^]^ Computational results for the HOS model suggest an almost instantaneous O─O bond formation, with an activation barrier of only 7.6 kcal/mol (Figure 2c). In contrast, the LOS model, with a higher activation energy of 13.4 kcal/mol for O─O coupling, provides a more convincing explanation for the observed delay (Figure 6).

### 2.6 The Effect of Net Charge

The cluster used for the LOS model has a formal net charge of ‐1. To investigate how changes in the net charge might affect the conclusions of this study, we removed a Cl⁻ anion, an outer‐sphere ligand, from the OEC and recalculated the key structures for a neutral LOS system (see the Computational Details for clarity of the discussion). For this system, we found that O─O coupling occurs with an overall activation free energy of 12.4 kcal/mol and produces O₂ from the S_4_ state with Δ*G* = −16.4 kcal/mol (Figure S5). These results indicate that changing the net charge of the complex has only a minimal effect on the energetic conclusions drawn in this study.

### 2.7 The Effect of Tyrosine Y_z_ and His190

To investigate the impact of Y_z_ and His190 on the kinetics and thermodynamics of O₂ evolution within the LOS paradigm, we used a simplified model derived from the 730 µs crystallographic data reported by Bhowmick et al.,^[^ ^24^ ^]^ focusing on a core cluster with approximately 190 atoms and a net charge of ‐1. For this system, we found that O─O coupling occurs with an overall activation free energy of 14.1 kcal/mol and produces O₂ from the S_4_ state with Δ*G* = −16.1 kcal/mol (Figure S6). These results suggest that Y_z_ and His190 do not substantially influence the energetic conclusions drawn in this study.

### 2.8 Mechanistic Insights into Proton Transfer in the LOS Paradigm

**Figure 8:**
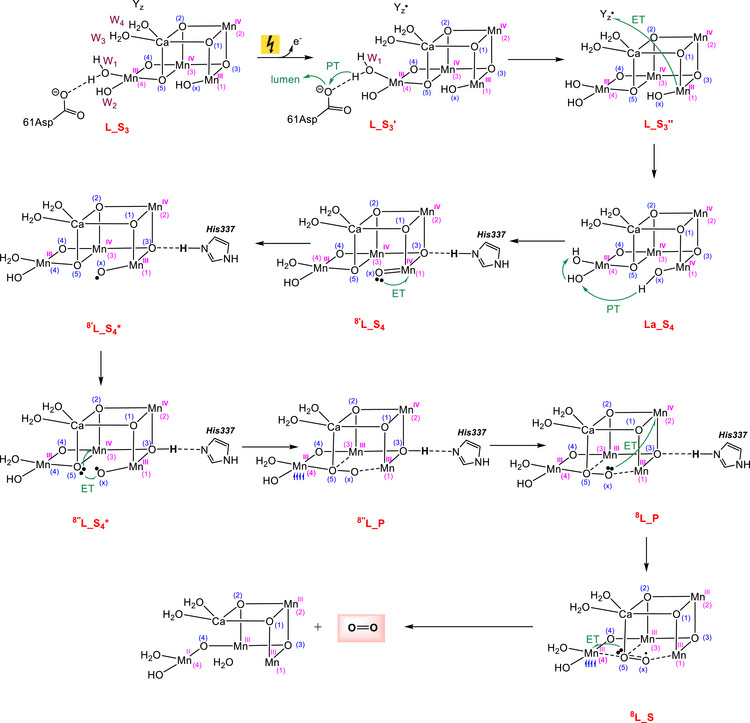
A plausible mechanism proposed for O₂ evolution in the LOS model during the transition from the S₃ to S₀ state.

It is well accepted that O₂ evolution begins with the photo‐oxidation process, ultimately resulting in the oxidation of tyrosine (Y_z_) to Y_z_^●^, driven by P680^+^. The oxidation of Y_z_ is thought to promote proton release from the OEC, facilitating the transfer of a proton from W_1_ to Asp61 and subsequently to the lumen (Figure 8).^[^ ^24^ ^]^ A recent computational study by Allgöwer et al. supports this assertion, reporting that proton release from protonated Asp61 occurs rapidly, with an overall activation barrier of approximately 6.9 kcal/mol.^[^ ^40^ ^]^ This study highlights the role of Y_z_ oxidation in generating an electric field that promotes efficient proton transfer along the Cl1 channel to the luminal bulk. The fact that proton release occurs at an early stage during the S₃ → S₀ transition has also been reported in numerous previous studies.^[^ ^41^ ^]^

**Figure 9:**
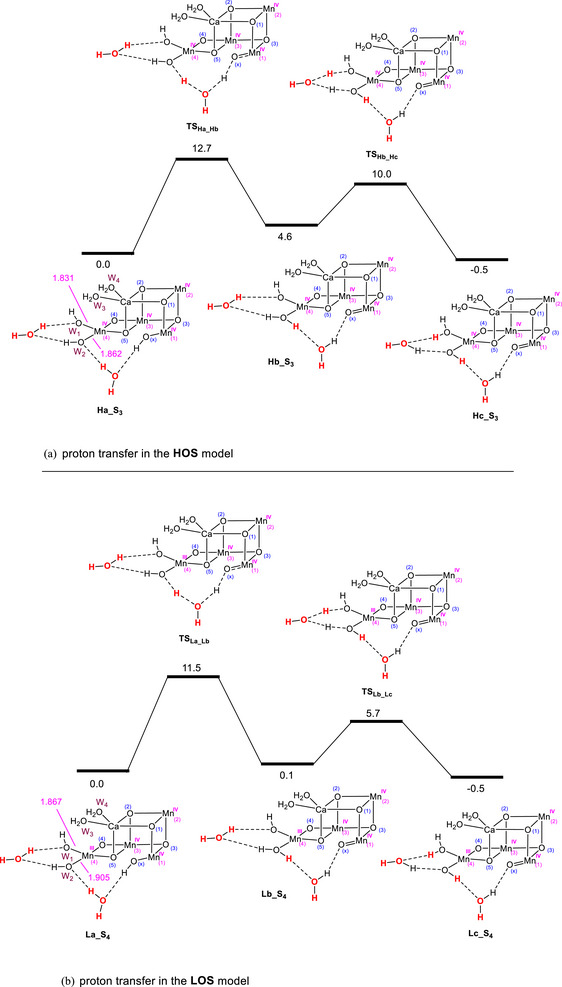
Comparison of potential energy profiles for proton transfer through the sequence O_x_H → W_2_ → W_3_ in the a) HOS model and b) LOS model. Since previous studies ref. [7] used relative potential energies to investigate this transformation, we have adopted the same approach here for the sake of comparison. The corresponding profiles based on free energy changes are provided in Figure S7. For a consistent comparison between these two models, ferromagnetic couplings were considered between all Mn atoms. The forward IRC calculations from **TS_Lb‐Lc_** yield **Lc_S_4_**, a conformer of **^14^L_S_4_**, which is approximately 2.1 kcal/mol less stable than **^14^L_S_4_** in terms of potential energy. The relative potential energies are given in kcal/mol and selected bond distances in Å.

After the initial proton release, the next step involves proton‐coupled electron transfer (PCET). In the HOS model, this step is proposed to begin with proton transfer from O_x_ → W_2_ → W_1_, followed by the oxidation of O_x_ to oxyl by Y_z_^●^. The proton transfer converts the hydroxide ligand bonded to Mn1 (O_x_H) into an oxo species with higher electron density, making it susceptible to oxidation. We calculated the potential energy profile for this proton transfer in the HOS paradigm, and our results are presented in Figure 9a. This process, mediated through a hydrogen bond network involving free water molecules positioned between O_x_H and W_2_ and between W_2_ and W_1_, is calculated to occur with an activation energy of 12.7 kcal/mol and a favorable reaction energy of ‐0.5 kcal/mol. Notably, the activation energy of 12.7 kcal/mol is comparable to the 11.8 kcal/mol reported by Song and Wang for the same proton transfer.^[^ ^27^ ^]^

In the LOS model, the presence of two Mn(III) centers with unpaired electrons in antibonding e_g_^∗^ orbitals makes the Mn(III) atoms highly susceptible to oxidation. This enables electron removal from the cluster to occur prior to O_x_ deprotonation in the PCET process, leading to the formation of intermediate **La_S_4_** (Figure 8). The resultant intermediate subsequently undergoes a proton transfer via the sequence O_x_ → W_2_ → W_1_, converting O_x_ into an oxo ligand primed for oxidation to generate the reactive oxyl species. We calculated this proton transfer process and found that it proceeds with an overall activation energy of 11.5 kcal/mol (approximately 1 kcal/mol less than the HOS model value) and a favorable reaction energy of ‐0.5 kcal/mol (Figure 9b).

After proton transfer, the oxo ligand in intermediate **^8′^L_S_4_** is oxidized by Mn1(IV) to form the oxyl complex ^8′^L_S_4_*, in which Mn1 has an oxidation state of + 3. The oxyl intermediate then gains more stability through the proton transfer from His337 to O3, producing intermediate **^8^** ^′′^ L_S_4_*, from which O─O coupling occurs and peroxide complex **^8^L_P** is formed. The oxidation of the peroxide ligand by Mn2(IV) generates the superoxide complex **^8^L_S**, in which all Mn atoms adopt an oxidation state of + 3. Finally, the transfer of an electron from the superoxide to Mn4 results in the release of an O_2_ molecule and a complex with S_0_ character.

An interesting finding from our calculations is that proton transfer proceeds with very similar energetics in both the HOS and LOS models (Figure 9). In the HOS model, all Mn atoms are in the + 4 oxidation state, whereas in the LOS model, Mn1, Mn2, and Mn3 are in the + 4 oxidation state, while Mn4 adopts the + 3 oxidation state. The nearly identical energetics for proton transfer suggest that the oxidation state of Mn4 has a minimal effect on the process. This can be understood by considering that the single electron in the e_g_* orbital of Mn4 resides along the Glu333–Mn4–Asp170 axis, as discussed above (Figure 3a), a direction orthogonal to the Mn4─W_1_ and Mn4─W_2_ bonds, and therefore has minimal impact on the acidity and basicity of W_1_ and W_2_. This claim is further supported by the comparable Mn4─W_1_ and Mn4─W_2_ bond distances in **Ha_S_3_** (1.831 and 1.862 Å, respectively) and **La_S_4_** (1.867 and 1.905 Å, respectively) (Figure 9).

To conclude this subsection, we revisited our earlier assertion that, in the LOS model, oxidation of the cluster occurs before proton transfer. To validate this, we explored the alternative scenario where proton transfer occurs prior to cluster oxidation and found that this pathway is not energetically favorable, with reaction energies exceeding +10 kcal/mol for the proton transfer. This finding reinforces the assertion that cluster oxidation must precede proton transfer; otherwise, the acidity of O_x_H is insufficient to make the proton transfer energetically favorable.

### 2.9 Alternative Pathways and Future Work

The results of our study suggest that the structural changes observed experimentally during the S_4_ → S_0_ transformation in PSII may be better rationalized when the reaction is modeled under the LOS paradigm. This lays a foundation for exploring other proposed mechanisms of the OEC within the LOS paradigm. For example, based on their femtosecond X‐ray crystallography findings, Bhowmick et al.^[^ ^24^ ^]^ proposed that, while coupling between O5 and O_x_ best aligns with the experimental data, two alternative O─O bond coupling pathways may also warrant consideration. These possibilities include the bond formation between W_2_ and O5 or between W_3_ and O5. Another possibility, suggested by isotope labeling experiments, is the proposed coupling between W_2_ and W_3_.^[^ ^42^ ^]^

It is important to note that these mechanisms, known as “water nucleophilic attack”^[^ ^1^, ^28^ ^]^ have been extensively investigated computationally using the HOS model and found to be much less favorable than oxo‐oxyl coupling.^[^ ^26^, ^29^ ^]^ However, as yet no studies have examined these pathways under the LOS model, leaving the question open as to whether the “water nucleophilic attack” mechanism could be more favorable than oxo‐oxyl coupling. Addressing this question will be the objective of our future studies.

## 3 Conclusion

This study provides valuable insights into the mechanism of oxygen evolution in PSII, suggesting that the OEC may operate within the LOS paradigm. Using density functional theory (DFT) calculations, we investigated the S_4_ → S_0_ transformation within the LOS paradigm and demonstrated that this model aligns more closely with recent experimental XFEL data than the HOS model, particularly regarding the coordination environment of Mn1 and the Mn4─O5 bond distance during peroxide intermediate formation.

Importantly, this model explains the significant omission of O_x_ observed experimentally at 1200 µs after peroxide complex formation. This omission, indicated by the drop in electron density below the 2.5σ threshold, may result from the weakening of the Mn1─O_x_ bond, leading to increased positional flexibility of O_x_ and, consequently, challenges in accurately determining its location in omit maps. This weak interaction is consistent with Mn1 being in the + 3 oxidation state within the LOS paradigm, where the Jahn‐Teller effect significantly weakens the Mn─Ox bond, potentially contributing to the difficulty in experimentally detecting O_x_.

We demonstrate that the LOS paradigm supports the formation of an oxyl species essential for triggering O─O bond coupling, providing clear evidence that oxyl formation is feasible within this paradigm, contrary to previous assumptions.

These findings provide a mechanistic framework that complements the widely accepted HOS paradigm, offering new perspectives on the intricate process of O₂ evolution in the OEC. By bridging computational and experimental insights, this study highlights the inherent complexity of PSII and paves the way for exploring new or refined mechanistic models that could further elucidate the process of oxygen evolution.

### 3.1 Computational Details

Gaussian 16^[^ ^43^ ^]^ was used to fully optimize all the structures reported in this study at the B3LYP level of theory.^[^ ^44^ ^]^ For all calculations, solvent effects were incorporated using the IEFPCM solvation model^[^ ^45^ ^]^ with a dielectric constant (ε) of 6.0. The Grimme empirical dispersion was added with the GD3 term for all the calculations.^[^ ^46^ ^]^ For geometry optimizations, the SDD basis set with effective core potential (ECP) was chosen to describe the Ca and Mn ions, and the 6–31G(d) basis set was employed for all other atoms.^[^ ^47^ ^]^ This basis set combination will be referred to as BS1. Frequency calculations were carried out at the same level of theory as those for structural optimization. Transition structures were located using the Berny algorithm. Intrinsic reaction coordinate (IRC) calculations were used to confirm the connectivity between transition structures and minima.^[^ ^48^ ^]^ To enhance the precision of the energies derived from the IEFPCM/B3LYP‐D3/SDD, 6–31G(d) calculations, we performed single‐point energy calculations for all structures using the B3LYP‐D3 functional method with a larger basis set (BS2), while considering the IEFPCM solvation model. This expanded basis set incorporates SDD‐ECP for the Mn atom and 6–311 + G(2d,p) for all other atoms. The antiferromagnetic electronic configurations were obtained through the application of the broken symmetry (BS) approach.^[^ ^34^ ^]^

**Figure 10:**
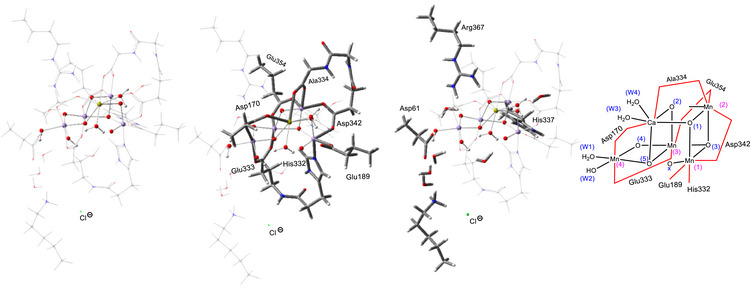
Model complex used in this study for S_4_ state. The first structure from the left represents the active site of PSII. The second structure includes the first coordination sphere ligands along with the active site. The third structure encompasses the second coordination sphere ligands and the active site.

In the OEC model (Figure 10), all amino acid residues directly bound to Mn and Ca are included, namely His332, Glu189, Asp342, Ala344, Glu354, Asp170, and Glu333. Additionally, the model incorporates His337 and Arg357, and the backbones of Leu343 and Ser169, as well as a portion of Gly171. Seven water molecules, positioned at crystallographic locations, are also included. The cluster in the LOS model formally has a ‐1 net charge, while the HOS model carries a + 1 net charge. We have adopted this structural model from a recent computational study by Siegbahn et al.,^[^ ^26a^ ^]^ who interpreted the 1.9 Å crystallographic data of PSII as representative of the S₃ state and used it to build their computational model.^[^ ^49^ ^]^ To investigate the impact of Y_z_ and His190 on O₂ evolution, we used a simplified model derived from the 730 µs crystallographic data reported by Bhowmick et al.,^[^ ^24^ ^]^ focusing on a core cluster with approximately 190 atoms and a net charge of ‐1.

## Conflict of Interest

The authors declare no conflict of interest.

## Supporting information



Supporting Information

## Data Availability

The data that support the findings of this study are available in the supplementary material of this article.
