# Supplementary material for: The Low Oxidation State Paradigm is More Consistent with XFEL Observations of the S₃ → [S₄] → S₀ Transition in Photosystem II
Source: Chemistry. 2025 Jun 18;31(38):e202501010. doi: 10.1002/chem.202501010 (PMC12238923; doi:10.1002/chem.202501010)
Supplement: Supplementary file 1 — Supporting Information [file CHEM-31-e202501010-s001.docx]

**Supporting information for**

**The Low Oxidation State Paradigm is more Consistent with XFEL Observations of the S₃ → [S₄] → S₀ Transition in Photosystem II**

*Alireza Ariafard,*^a^ Matthew Longhurst,^b^ Gerhard F. Swiegers,^b^ Robert Stranger*^a^*

^a^ Research School of Chemistry, Australian National University, Canberra, Australia

^b^ Intelligent Polymer Research Institute, University of Wollongong, Wollongong, Australia

Table of contents:

**Figure S1.** NLMO orbitals for two lone pairs on the O_x_ ligand with p character in **^14^L_S_4_**. (page S2)

**Figure S2.** The spatial plots for the HOMO, LUMO, and LUMO+1 along with their energies for complex **^14^L_S_4_**. (page S3)

**Figure S3.** NLMO orbital for the single electron on the O_x_ ligand in **^8'^L_S_4_*** and **^16^L_S_4_***. (page S3)

**Figure S4.** Spatial plots of the LUMO and LUMO+1 orbitals, along with their corresponding energies, for the **^14^H_S_4_** species. (page S4)

**Figure S5.** Calculated key structures and their corresponding relative free energies (in kcal/mol) involved in O₂ evolution from the S_4_ state with a net charge of zero. (page S5)

**Figure S6.** Calculated key structures and their corresponding relative free energies (in kcal/mol) involved in O₂ evolution from the S_4_ state, incorporating Y_z_ and His190. (page S6)

**Figure S7.** Comparison of free energy profiles for proton transfer through the sequence O_x_H → W_2_ → W_3_ in the HOS and LOS models. (page S7)

**Table S1.** Cartesian coordinates for all structures optimized in this study. (page S8)

**Figure S1.** NLMO orbitals for two lone pairs on the O_x_ ligand with p character in **^14^L_S_4_**. The α spin of the lone pairs remains primarily on the O_x_ ligand (two top spatial plots), while the β spin of the lone pairs interacts significantly with the dπ orbitals of Mn1 (two bottom spatial plots).

**Figure S2.** The spatial plots for the HOMO, LUMO, and LUMO+1 along with their energies for complex **^14^L_S_4_**.

**Figure S3.** NLMO orbital for the single electron on the O_x_ ligand in **^8'^L_S_4_*** (left) and **^16^L_S_4_*** (right). The NLMO calculations suggest that when the oxyl and Mn1 are coupled ferromagnetically in **^16^L_S_4_***, the single electron on the oxyl ligand remains in a nonbonding orbital. Conversely, when they are coupled antiferromagnetically in **^8'^L_S_4_***, the single electron on the oxyl ligand engages in a π interaction with a t_2g_ orbital on Mn1, thereby stabilizing the system.

**Figure S4.** Spatial plots of the LUMO and LUMO+1 orbitals, along with their corresponding energies, for the **^14^H_S_4_** species.

**Figure S5.** Calculated key structures and their corresponding relative free energies (in kcal/mol) involved in O₂ evolution from the S_4_ state with a net charge of zero. In this system, O–O coupling occurs with an overall activation free energy of 12.4 kcal/mol, leading to O₂ formation from the S_4_ state with ΔG = -16.4 kcal/mol.

**Figure S6.** Calculated key structures and their corresponding relative free energies (in kcal/mol) involved in O₂ evolution from the S_4_ state, incorporating Y_z_ and His190. In this system, O–O coupling occurs with an overall activation free energy of 14.1 kcal/mol, leading to O₂ formation from the S_4_ state with ΔG = -16.1 kcal/mol.

**Figure S7.** Comparison of free energy profiles for proton transfer through the sequence O_x_H → W_2_ → W_3_ in the (a) HOS model and (b) LOS model. Since previous For a consistent comparison between these two models, ferromagnetic couplings were considered between all Mn atoms. The relative free energies are given in kcal/mol and selected bond distances in Å.

**Table S1.** Cartesian coordinates for all structures optimized in this study. Total potential (E), enthalpy (H) and Gibbs free energies (G) of structures optimized at the IEFPCM/B3LYP-D3/SDD,6-31G(d) level of theory along with the total potential energies calculated by IEFPCM/B3LYP-D3/SDD,6-311+G(2d,p)//IEFPCM/B3LYP-D3/SDD,6-31G(d) in a solvent with ε = 6.0.

In the Cartesian coordinates, a '-1' sign following an atom indicates it was fixed, while a '0' signifies it remained fully unconstrained during the optimization process.

**^6^H_S_4_**

E (IEFPCM/B3LYP-D3/SDD,6-31G(d)) = -5853.942847 au

H (IEFPCM/B3LYP-D3/SDD,6-31G(d)) = -5852.388217 au

G (IEFPCM/B3LYP-D3/SDD,6-31G(d)) = -5852.625232 au

E (IEFPCM/B3LYP-D3/SDD,6-311+G(2d,p)//IEFPCM/B3LYP-D3/SDD,6-31G(d)) = -5855.749438 au

Mn 0 1.45408200 1.89751200 -1.85913900

Mn 0 -0.99411800 -2.41450600 -0.68369900

Mn 0 -0.17601900 0.84266300 0.08709500

Mn 0 -2.71607300 -0.42888600 0.28541900

O 0 0.39017000 0.43178400 -1.57111700

O 0 -1.91993600 0.99606400 -0.50312100

O 0 -2.54302800 -1.50900000 -1.16663000

O 0 -0.94859300 -1.02626700 0.62783400

Ca 0 -1.79259200 0.11299100 -2.84552700

C -1 0.68824900 4.49709600 -5.66613200

H -1 0.75279300 5.44240300 -5.14772000

H -1 0.89797900 4.54727600 -6.72414000

H 0 1.55233400 3.93709400 -5.27685900

C 0 -0.56084800 3.68690700 -5.27977900

H 0 -0.75223600 2.90344900 -6.02487000

H 0 -1.47115800 4.29733500 -5.25738200

C 0 -0.52510600 2.91050500 -3.94411400

O 0 -1.54850500 2.26996100 -3.61420800

O 0 0.56603900 2.93910500 -3.26359600

C -1 -2.89825800 -6.71642400 -5.21754900

H -1 -1.93971400 -7.19327000 -5.35973500

H -1 -3.61704400 -6.86875200 -6.00909200

H 0 -3.33971000 -7.24162700 -4.35544400

C 0 -2.61300800 -5.27560600 -4.78517600

H 0 -3.52778600 -4.76207600 -4.46308700

H 0 -2.17317700 -4.68810700 -5.59895400

C 0 -1.61737400 -5.35701800 -3.61179100

H 0 -0.68597700 -5.82562000 -3.95927800

H 0 -2.01899000 -5.98294200 -2.81009900

C 0 -1.22745600 -4.01817700 -3.03303700

O 0 -0.88956700 -3.07970300 -3.76976400

O 0 -1.28371500 -3.97150800 -1.73036900

C -1 4.99576500 -4.60245000 1.21054000

H 0 5.76348600 -5.34870400 1.44573900

H 0 5.30068300 -4.07287200 0.30494000

C 0 4.87097500 -3.64361100 2.38894500

O 0 4.31605900 -3.99762600 3.44854100

C 0 3.65256100 -5.35588500 0.97549200

H 0 3.46577100 -6.00299200 1.84035300

H 0 3.75108500 -6.00015200 0.09812300

C 0 2.47206700 -4.44575800 0.78491600

N 0 2.17108400 -3.49192800 1.74554400

H 0 2.66327000 -3.41969600 2.63597600

C 0 1.53030500 -4.25943800 -0.20188500

H 0 1.38658200 -4.78670400 -1.13174700

C 0 1.14103200 -2.74838000 1.32858500

H 0 0.78746200 -1.86769700 1.82706600

N 0 0.70139600 -3.20300000 0.16048000

N 0 5.27895600 -2.38007700 2.16860000

H 0 5.62710500 -2.15477600 1.23378100

C -1 4.96556100 -1.28340000 3.08082300

H 0 5.85064200 -0.64098000 3.16047300

H 0 4.76061500 -1.71105600 4.06266000

C 0 3.75271100 -0.44674200 2.59492400

H 0 3.55863000 0.34189900 3.33246200

H 0 2.86894400 -1.08669900 2.59232300

C 0 3.93942200 0.17646600 1.19871800

H 0 4.43308800 -0.52092400 0.50988300

H 0 4.58963400 1.05774100 1.23617400

C 0 2.67628300 0.59349200 0.46431300

O 0 2.85433700 1.15202000 -0.66563000

O 0 1.53866400 0.33125600 0.96660100

C -1 -5.52251100 -3.82963100 2.23738100

H 0 -5.42161600 -3.07149200 3.02121500

H 0 -6.01954700 -4.69703200 2.68857500

C 0 -4.14837600 -4.26273700 1.72180000

H 0 -4.25661800 -4.99889900 0.91920000

H 0 -3.58881600 -4.76116100 2.52262000

C 0 -3.25283000 -3.16168300 1.17120700

O 0 -3.62760600 -1.95114300 1.26842200

O 0 -2.14331300 -3.53014800 0.67517300

C 0 -6.41930700 -3.30811000 1.11772600

O 0 -6.22857700 -3.59248700 -0.07354100

N 0 -7.47379800 -2.56335900 1.52089800

H 0 -7.53180700 -2.28334800 2.49054800

C -1 -8.45024000 -2.00163000 0.59281900

H 0 -9.37896100 -1.81191000 1.13078200

H 0 -8.62922400 -2.73082600 -0.20192200

C 0 -7.95981500 -0.65862400 0.00856200

O 0 -8.38961200 0.41649800 0.42379400

N 0 -7.02687400 -0.77639700 -0.97318700

H 0 -6.65372000 -1.69362200 -1.19239300

C 0 -6.38876000 0.36443300 -1.59383700

H 0 -6.56510700 0.37473000 -2.67301000

H 0 -6.83077000 1.26613800 -1.15801400

C 0 -4.87256700 0.40141000 -1.37999200

O 0 -4.12424700 0.70267200 -2.32189000

O 0 -4.48827600 0.11756400 -0.17078100

C -1 -3.47391600 3.83387900 4.87458100

H -1 -3.52521500 3.56143800 5.91839400

H -1 -4.27897100 4.47973900 4.55657700

H 0 -2.56481400 4.44335400 4.76762100

C 0 -3.41931200 2.67752000 3.86896000

H 0 -3.67262800 3.07854000 2.87919700

H 0 -4.17083700 1.91135100 4.08739000

C 0 -2.02569100 2.04636000 3.78149200

H 0 -1.85195000 1.38569200 4.63967000

H 0 -1.24792700 2.81404400 3.82219200

C 0 -1.81552000 1.27807300 2.49531000

O 0 -0.69975500 1.45640300 1.91409700

O 0 -2.74264500 0.51608700 2.07089700

C -1 -5.44704300 9.19972100 1.73900200

H -1 -5.33137000 8.81023100 2.73966300

H -1 -6.47646800 9.44275000 1.52087900

H 0 -4.87851100 10.13514900 1.67760500

C 0 -4.88622500 8.16451700 0.75056200

H 0 -3.79192500 8.14983100 0.85694000

H 0 -5.08819300 8.46608100 -0.28562300

C 0 -5.46064500 6.76130700 1.00063300

H 0 -6.54755600 6.78195900 0.85467800

H 0 -5.28782400 6.47082200 2.04596700

C 0 -4.89494700 5.66748600 0.09038900

H 0 -5.06820900 5.92581600 -0.96408900

H 0 -5.40452500 4.72007900 0.29597600

N 0 -3.46159100 5.47457000 0.33335800

H 0 -2.92664900 6.28805000 0.60778300

C 0 -2.74366700 4.41880700 -0.08854800

N 0 -3.31483200 3.43057800 -0.78631300

H 0 -2.83826400 2.52703900 -0.86719300

H 0 -4.21070700 3.54195800 -1.23515900

N 0 -1.43810800 4.35167600 0.18937100

H 0 -1.01286400 4.79151200 1.00198600

H 0 -0.86044800 3.59752700 -0.19866800

C -1 -1.11944000 -1.09677300 7.56234900

H -1 -0.12420900 -0.71322500 7.39258000

H -1 -1.31747400 -1.27897700 8.60826700

H 0 -1.85881700 -0.38374300 7.18344000

C 0 -1.28475600 -2.49209000 6.92587400

H 0 -2.25544900 -2.91172600 7.22327100

H 0 -0.52545200 -3.16544800 7.34319800

C 0 -1.20071300 -2.54030700 5.43768900

N 0 -1.02152900 -3.72952800 4.74059200

C 0 -1.32996800 -1.57682100 4.47674500

H 0 -1.48739000 -0.51952100 4.57048600

C 0 -1.04282300 -3.50480600 3.42268700

H 0 -0.93139500 -4.23562900 2.63886200

N 0 -1.23609200 -2.19747700 3.24711600

H 0 -1.25254800 -1.72945200 2.32628400

C -1 3.78516400 6.28073600 2.30772300

H -1 4.66482500 6.75758700 1.90124400

H -1 3.30970000 6.82100200 3.11298400

H 0 3.04171700 6.26928500 1.50109600

C 0 4.07907900 4.81270200 2.69461400

H 0 5.00008900 4.72061100 3.27764500

H 0 3.24448400 4.42738800 3.29238800

C 0 4.15938000 3.95644900 1.43266100

O 0 3.10556000 3.90643700 0.73304500

O 0 5.24649200 3.37632600 1.12359300

C -1 12.09980800 -2.47272200 -0.35865900

H -1 13.14931700 -2.22103900 -0.39849000

H -1 11.87696600 -3.37267700 -0.91257000

H 0 11.87523200 -2.71381000 0.69011600

C 0 11.20406000 -1.29925800 -0.82435800

H 0 11.73575000 -0.70230500 -1.57824100

H 0 10.31182100 -1.69396200 -1.32711200

C 0 10.76479100 -0.38927300 0.33762800

H 0 10.01674500 -0.91927000 0.94513900

H 0 11.62184700 -0.21790100 1.00304700

C 0 10.23839000 0.99176800 -0.08400500

H 0 9.99796900 1.58780000 0.80653800

H 0 11.04591300 1.52568400 -0.60210400

C 0 9.03467600 1.02940400 -1.02740600

H 0 8.90653100 2.04674700 -1.40570200

H 0 9.15928000 0.36880200 -1.88820800

N 0 7.72675400 0.67038600 -0.37516400

H 0 6.97142600 1.22918800 -0.83037800

H 0 7.44896900 -0.32277900 -0.47674000

H 0 7.68026700 0.95509100 0.62984000

Cl 0 5.91289900 -1.87425500 -1.06401500

O 0 2.29975900 1.02898200 -3.26988100

H 0 3.74195200 2.07413300 -3.64199900

O 0 0.45094400 2.43562900 -0.36944000

O 0 0.67174400 4.07641800 2.16145000

H 0 0.35488500 3.16474500 2.03719800

H 0 1.47240900 4.10657800 1.58873500

O 0 0.05223300 -0.25790700 -4.43206600

H 0 0.85429300 0.23792000 -4.12447000

H 0 0.26537700 -1.19439500 -4.26738200

O 0 -3.07577600 -1.39657100 -4.25406000

H 0 -3.81749100 -1.82353400 -3.77284500

H 0 -2.45340400 -2.12403000 -4.44845900

O 0 -4.72701800 -2.59131200 -2.27003800

H 0 -3.96344300 -2.27673500 -1.73327300

H 0 -5.17955700 -3.23226300 -1.69063200

O 0 2.54994300 3.55121100 -1.75905900

H 0 2.91664600 3.62220400 -0.81483200

H 0 3.57321600 -1.66399800 -1.82926200

O 0 -0.11955300 -1.71541500 -1.97523300

O 0 5.98883800 2.63045800 -1.30724800

H 0 5.53603100 2.87479500 -0.44314200

H 0 6.56281500 3.39266700 -1.49192500

O 0 4.34805400 2.84431000 -3.51497600

H 0 4.97862900 2.55903100 -2.81350700

H 0 3.30423100 3.44933500 -2.42978100

O 0 7.29247500 1.88178600 2.09913400

H 0 6.55335000 2.50730800 1.89274000

H 0 7.06502000 1.47475900 2.94794600

O 0 2.79568500 -1.54575500 -2.41193700

H 0 2.62190900 0.15381300 -2.92298700

H 0 1.99409500 -1.62913400 -1.86457300

H 0 -0.89427900 -4.64233300 5.16225200

**^6^H_P**

E (IEFPCM/B3LYP-D3/SDD,6-31G(d)) = -5853.964137 au
H (IEFPCM/B3LYP-D3/SDD,6-31G(d)) = -5852.408947 au
G (IEFPCM/B3LYP-D3/SDD,6-31G(d)) = -5852.647061 au
E (IEFPCM/B3LYP-D3/SDD,6-311+G(2d,p)//IEFPCM/B3LYP-D3/SDD,6-31G(d)) = -5855.772251 au

Mn 0 1.53422300 1.89664000 -1.98335500

Mn 0 -0.95011200 -2.41291800 -0.62838900

Mn 0 -0.23117500 0.70786300 0.13550200

Mn 0 -2.75096100 -0.46464800 0.27179600

O 0 0.07790500 -0.06883700 -1.56443000

O 0 -1.97696300 0.96930800 -0.44393800

O 0 -2.41997700 -1.47617200 -1.23638200

O 0 -1.03160000 -1.08708800 0.74531100

Ca 0 -2.01333800 0.22273500 -2.97987400

C -1 0.67831700 4.67228200 -5.55618100

H -1 0.74782200 5.60440200 -5.01503100

H -1 0.88258400 4.74801000 -6.61373700

H 0 1.52821400 4.08744100 -5.17811400

C 0 -0.59387800 3.87624800 -5.22155200

H 0 -0.82258200 3.16217000 -6.02389400

H 0 -1.48278800 4.51213900 -5.13562500

C 0 -0.53360300 3.01007500 -3.95318000

O 0 -1.56523100 2.37272100 -3.61819100

O 0 0.58108300 2.95938300 -3.33043000

C -1 -2.93220400 -6.54086400 -5.36438200

H -1 -1.97554900 -7.01620100 -5.52335800

H -1 -3.65550900 -6.67206900 -6.15559200

H 0 -3.36788200 -7.06401100 -4.49890100

C 0 -2.67054300 -5.07811800 -4.95299100

H 0 -3.60445000 -4.58070000 -4.66222600

H 0 -2.24543300 -4.50379100 -5.78419100

C 0 -1.69517700 -5.06504300 -3.76325400

H 0 -0.73241100 -5.50533000 -4.06339000

H 0 -2.07368900 -5.69494300 -2.95087400

C 0 -1.37160700 -3.70342400 -3.17288100

O 0 -1.43748600 -2.65247600 -3.81978900

O 0 -0.98977900 -3.77765200 -1.91796700

C -1 5.00055900 -4.60327000 1.07159500

H 0 5.81130000 -5.32332100 1.23363200

H 0 5.22896000 -4.03859100 0.16452800

C 0 4.91558900 -3.67797100 2.28285100

O 0 4.39856900 -4.06568400 3.35059800

C 0 3.67525600 -5.40303100 0.90516400

H 0 3.56391700 -6.06892100 1.76836200

H 0 3.74367100 -6.02936200 0.01203900

C 0 2.45740600 -4.53009300 0.79710200

N 0 2.17446100 -3.60382200 1.78958600

H 0 2.71107200 -3.53736000 2.65549400

C 0 1.47145400 -4.34915200 -0.14592900

H 0 1.29939600 -4.85514900 -1.08268400

C 0 1.11341900 -2.87397700 1.42994700

H 0 0.76955000 -2.00429700 1.95581700

N 0 0.63680800 -3.32031300 0.27334200

N 0 5.30944100 -2.40685600 2.08764100

H 0 5.66422300 -2.15895700 1.16024800

C -1 4.98804000 -1.33118100 3.02306700

H 0 5.85875800 -0.66882800 3.09619200

H 0 4.80966600 -1.77642600 4.00227900

C 0 3.75008100 -0.51873300 2.56168500

H 0 3.54881100 0.26233100 3.30538700

H 0 2.88031300 -1.17820600 2.56682300

C 0 3.91212600 0.11182300 1.16714900

H 0 4.44065200 -0.56269400 0.48219100

H 0 4.52782000 1.01913400 1.20715000

C 0 2.64626400 0.48011100 0.41229400

O 0 2.81479000 0.99289300 -0.73145500

O 0 1.50545600 0.20716100 0.92488200

C -1 -5.51030700 -3.83252200 2.17315800

H 0 -5.34945800 -3.07545700 2.94766900

H 0 -6.03082500 -4.67448000 2.64521800

C 0 -4.17616700 -4.32459700 1.61059200

H 0 -4.33511900 -5.05622600 0.81279700

H 0 -3.60598500 -4.83748800 2.39375200

C 0 -3.28621600 -3.23408600 1.03902600

O 0 -3.64908900 -2.02786600 1.17419100

O 0 -2.19702100 -3.60574300 0.49174300

C 0 -6.41522500 -3.27142900 1.07876300

O 0 -6.22731800 -3.50696400 -0.12466300

N 0 -7.47167400 -2.54765700 1.50688400

H 0 -7.54412700 -2.31383700 2.48782500

C -1 -8.44234600 -1.95809600 0.58966900

H 0 -9.37618800 -1.79351800 1.12749900

H 0 -8.61308300 -2.65963600 -0.23074500

C 0 -7.94879800 -0.59580000 0.06907000

O 0 -8.29433800 0.45972000 0.59575300

N 0 -7.10530600 -0.67500000 -0.99469200

H 0 -6.76834400 -1.58788200 -1.27682700

C 0 -6.44538200 0.48230200 -1.55319200

H 0 -6.62450300 0.55699300 -2.62918500

H 0 -6.86566800 1.36880700 -1.06726700

C 0 -4.93078500 0.47903500 -1.34186900

O 0 -4.19164600 0.89362700 -2.24903800

O 0 -4.53536300 0.04034400 -0.18886800

C -1 -3.42977400 3.75920300 4.98706300

H -1 -3.47621200 3.46127800 6.02411800

H -1 -4.23497000 4.41448300 4.68933200

H 0 -2.51687200 4.36309700 4.88418400

C 0 -3.40108600 2.62351300 3.95025000

H 0 -3.68419200 3.04804500 2.97852700

H 0 -4.14682800 1.85291300 4.17326400

C 0 -2.01238100 1.99119600 3.79878300

H 0 -1.80448600 1.31573500 4.63830900

H 0 -1.23281200 2.75793000 3.82188100

C 0 -1.84151200 1.23444800 2.49703000

O 0 -0.72550700 1.41863600 1.90244300

O 0 -2.77902700 0.48246300 2.08222100

C -1 -5.40674700 9.20494800 1.99494100

H -1 -5.28671900 8.79069800 2.98509100

H -1 -6.43673200 9.45556100 1.78834800

H 0 -4.84185100 10.14403900 1.95872800

C 0 -4.83022500 8.19697200 0.98147400

H 0 -3.73338500 8.22815100 1.05074900

H 0 -5.08090700 8.49305500 -0.04566100

C 0 -5.32757800 6.76633900 1.24226000

H 0 -6.42249100 6.74697900 1.17259100

H 0 -5.07352100 6.46591200 2.26807200

C 0 -4.79921800 5.70281600 0.27365000

H 0 -5.03743000 5.98637900 -0.76145600

H 0 -5.29331100 4.74888200 0.48502500

N 0 -3.35266300 5.49278600 0.41698300

H 0 -2.78286000 6.29236900 0.66169900

C 0 -2.69417100 4.41338000 -0.04499400

N 0 -3.34830300 3.44169500 -0.69514900

H 0 -2.89774300 2.53297800 -0.82060000

H 0 -4.24116100 3.59610700 -1.13754700

N 0 -1.37637200 4.29277500 0.14454500

H 0 -0.88082600 4.69343100 0.94146900

H 0 -0.87025900 3.49998900 -0.26765900

C -1 -1.07279400 -1.24131600 7.54018400

H -1 -0.07757000 -0.85592500 7.37459900

H -1 -1.26574000 -1.44875800 8.58234500

H 0 -1.81663800 -0.52481500 7.17761000

C 0 -1.24516900 -2.63400800 6.89781400

H 0 -2.19680600 -3.06788600 7.23413200

H 0 -0.46035900 -3.30002500 7.27892100

C 0 -1.22744200 -2.67294100 5.40937700

N 0 -1.11030500 -3.85989000 4.69627600

C 0 -1.36678600 -1.69660900 4.46338000

H 0 -1.48730100 -0.63515400 4.57137500

C 0 -1.17243000 -3.61992500 3.38168400

H 0 -1.08750900 -4.34436600 2.58819700

N 0 -1.33824700 -2.30667100 3.22649900

H 0 -1.34030200 -1.81336700 2.31328500

C -1 3.82142200 6.25229700 2.44251000

H -1 4.70005000 6.73704500 2.04320000

H -1 3.35148500 6.77364500 3.26333600

H 0 3.07480200 6.24481800 1.63849700

C 0 4.11968800 4.78196400 2.82797500

H 0 5.03855100 4.69867700 3.41591700

H 0 3.28610000 4.39965400 3.42972700

C 0 4.20697300 3.90165600 1.58138700

O 0 3.15652800 3.80527800 0.88360700

O 0 5.30621500 3.33628000 1.28106500

C -1 12.10123300 -2.45140600 -0.48258500

H -1 13.15110700 -2.20115800 -0.52180600

H -1 11.87335200 -3.33696300 -1.05726400

H 0 11.88154500 -2.71835000 0.56107200

C 0 11.21041200 -1.26059200 -0.90942600

H 0 11.72424700 -0.67078100 -1.68122500

H 0 10.29125200 -1.63534400 -1.37761700

C 0 10.83694000 -0.34946300 0.27477500

H 0 10.09248600 -0.86327200 0.90027000

H 0 11.71956000 -0.21244300 0.91430200

C 0 10.33996300 1.05253500 -0.11209800

H 0 10.14340000 1.64020700 0.79468100

H 0 11.14719300 1.57148400 -0.64572500

C 0 9.11049500 1.13952400 -1.01780400

H 0 8.98532500 2.17145500 -1.35600000

H 0 9.20317900 0.50813400 -1.90439700

N 0 7.82009700 0.76776800 -0.34105900

H 0 7.04531500 1.32808000 -0.76547100

H 0 7.54753800 -0.22593300 -0.45868600

H 0 7.79567200 1.02838900 0.67170600

Cl 0 6.11525600 -1.82145900 -1.07228200

O 0 2.28533900 0.94522200 -3.39542300

H 0 3.77773100 2.03153300 -3.68459200

O 0 0.34670500 2.19612200 -0.53886700

O 0 0.66932000 4.02336300 2.12012600

H 0 0.36770700 3.10470000 2.01135700

H 0 1.52849800 4.04066900 1.63294800

O 0 -0.08083900 -0.21503200 -4.40389900

H 0 0.77328000 0.21571800 -4.13378300

H 0 0.05124200 -1.17088300 -4.26955200

O 0 -3.57030400 -1.08469400 -4.34025400

H 0 -4.25781000 -1.47954100 -3.76103500

H 0 -2.99674700 -1.84783200 -4.55873300

O 0 -4.66444500 -2.60248800 -2.27579000

H 0 -3.84995200 -2.29745900 -1.81808800

H 0 -5.18386000 -3.05153000 -1.57973000

O 0 2.71081200 3.69193700 -1.72117400

H 0 3.05726500 3.68100500 -0.78579700

H 0 3.91225500 -1.57502000 -1.94353300

O 0 0.36876400 -1.44076200 -1.51384900

O 0 5.94565400 2.63067400 -1.19238900

H 0 5.53531000 2.85286700 -0.30155300

H 0 6.45140500 3.42842700 -1.42082800

O 0 4.45828600 2.72957500 -3.53942000

H 0 4.99471900 2.40451300 -2.78434000

H 0 3.46447000 3.55588200 -2.35697400

O 0 7.40445000 1.86853800 2.18822900

H 0 6.64449100 2.47676800 2.00807100

H 0 7.16627700 1.38017300 2.98992600

O 0 3.07731200 -1.55467900 -2.45784300

H 0 2.69046200 0.10726200 -3.04704800

H 0 2.34788000 -1.63030900 -1.81705200

H 0 -0.99614700 -4.77975400 5.10610300

**^6^H_S**

E (IEFPCM/B3LYP-D3/SDD,6-31G(d)) = -5853.954020 au
H (IEFPCM/B3LYP-D3/SDD,6-31G(d)) = -5852.398134 au
G (IEFPCM/B3LYP-D3/SDD,6-31G(d)) = -5852.638557 au
E (IEFPCM/B3LYP-D3/SDD,6-311+G(2d,p)//IEFPCM/B3LYP-D3/SDD,6-31G(d)) = -5855.762254 au

Mn 0 1.49925100 2.01292900 -1.87918800

Mn 0 -0.91951900 -2.49586600 -0.67476100

Mn 0 -0.26986800 0.72340100 0.20565500

Mn 0 -2.74833500 -0.54888100 0.21442700

O 0 0.24842200 0.09786300 -1.61194400

O 0 -1.95179000 0.92018800 -0.48554000

O 0 -2.40452700 -1.49753100 -1.30239900

O 0 -1.01436000 -1.10195500 0.70819300

Ca 0 -2.12522200 0.24452600 -3.07494000

C -1 0.68093600 4.65240600 -5.54431200

H -1 0.75979300 5.58250700 -5.00097500

H -1 0.88418500 4.72889800 -6.60200900

H 0 1.52581200 4.05786500 -5.16794500

C 0 -0.59716000 3.87179600 -5.19904300

H 0 -0.81080200 3.12160000 -5.97171200

H 0 -1.48694400 4.51177400 -5.15998900

C 0 -0.56409200 3.06517900 -3.88975700

O 0 -1.58237800 2.39107300 -3.58642700

O 0 0.50739200 3.10281500 -3.19402900

C -1 -3.00282400 -6.50123500 -5.39983700

H -1 -2.05079200 -6.98486100 -5.56154300

H -1 -3.72856400 -6.62388700 -6.19019000

H 0 -3.44103300 -7.00495300 -4.52489000

C 0 -2.71976900 -5.03424000 -5.02569300

H 0 -3.64465100 -4.51427400 -4.74574700

H 0 -2.28891200 -4.49012200 -5.87440900

C 0 -1.74387200 -5.00823300 -3.84364300

H 0 -0.80933200 -5.52305100 -4.11312800

H 0 -2.15370400 -5.56902600 -2.99545800

C 0 -1.33462700 -3.64671200 -3.31817600

O 0 -1.46315600 -2.59980400 -3.96887300

O 0 -0.79744000 -3.70648100 -2.12529700

C -1 4.92919000 -4.67853100 1.05349400

H 0 5.77109100 -5.36824800 1.19127100

H 0 5.11999400 -4.10525300 0.14223700

C 0 4.85463600 -3.75566000 2.26855400

O 0 4.35098000 -4.14723700 3.34110300

C 0 3.63134500 -5.51563900 0.91623600

H 0 3.53327000 -6.15965500 1.79684900

H 0 3.70502700 -6.16042800 0.03692300

C 0 2.42409700 -4.64004700 0.78984400

N 0 2.11769400 -3.73718300 1.79753700

H 0 2.62958500 -3.69554900 2.67999200

C 0 1.51977000 -4.37942300 -0.21019000

H 0 1.38776600 -4.84467800 -1.17410800

C 0 1.12192100 -2.94421100 1.39188200

H 0 0.76798300 -2.08655900 1.92915600

N 0 0.71155700 -3.32239000 0.18574800

N 0 5.25174900 -2.48627800 2.07241300

H 0 5.62749700 -2.23885600 1.15265700

C -1 4.94959400 -1.41132000 3.01305800

H 0 5.83218000 -0.76512000 3.09343500

H 0 4.76138900 -1.85871100 3.98957700

C 0 3.72337700 -0.58083800 2.55529000

H 0 3.51824500 0.18729200 3.31122500

H 0 2.85263400 -1.23899200 2.53922800

C 0 3.89155500 0.07739900 1.17393600

H 0 4.39716200 -0.59472300 0.46841200

H 0 4.52632800 0.97087600 1.22649300

C 0 2.61956800 0.49168000 0.45128000

O 0 2.77956900 1.04092900 -0.67994500

O 0 1.48561800 0.21161400 0.97022700

C -1 -5.57244100 -3.81490200 2.17407100

H 0 -5.37054200 -3.04188400 2.92344200

H 0 -6.12874400 -4.61450200 2.67896600

C 0 -4.27523200 -4.37527100 1.61641300

H 0 -4.47222000 -5.10904300 0.82892800

H 0 -3.72823900 -4.90651000 2.40415600

C 0 -3.32747500 -3.34636000 1.02692600

O 0 -3.64144100 -2.10970800 1.11836900

O 0 -2.26002200 -3.78505500 0.52077000

C 0 -6.47116000 -3.24186400 1.08088200

O 0 -6.29562900 -3.48180000 -0.12407800

N 0 -7.51494900 -2.50093700 1.51201900

H 0 -7.58127400 -2.26710100 2.49344800

C -1 -8.48983500 -1.90994500 0.59999100

H 0 -9.41387200 -1.72571200 1.14838700

H 0 -8.68118800 -2.61967600 -0.20897000

C 0 -7.98732600 -0.56257700 0.05199100

O 0 -8.33597100 0.50763500 0.54615800

N 0 -7.13195300 -0.67263900 -0.99940100

H 0 -6.79696600 -1.59577400 -1.25012100

C 0 -6.46076600 0.46727300 -1.57679900

H 0 -6.63054400 0.52064700 -2.65591800

H 0 -6.88196800 1.36749000 -1.11731800

C 0 -4.94772900 0.46922600 -1.35641800

O 0 -4.21739900 0.94762800 -2.24295900

O 0 -4.54264800 -0.02684500 -0.23568200

C -1 -3.41836300 3.75056200 5.00332100

H -1 -3.46584700 3.45049700 6.03971200

H -1 -4.21803700 4.41387900 4.70852100

H 0 -2.49468000 4.33675200 4.89559300

C 0 -3.43030300 2.63726100 3.93951900

H 0 -3.60853500 3.11858300 2.96882100

H 0 -4.26457000 1.94401000 4.09277700

C 0 -2.11030600 1.85704200 3.85097600

H 0 -2.07240700 1.11247800 4.65396400

H 0 -1.25530700 2.52487000 3.98556700

C 0 -1.92915000 1.15677400 2.51651400

O 0 -0.79950100 1.37938200 1.94264200

O 0 -2.84498800 0.42139500 2.05232000

C -1 -5.35047800 9.22148900 2.02787900

H -1 -5.23263600 8.80370500 3.01680600

H -1 -6.37846900 9.48197500 1.82358200

H 0 -4.77480200 10.15385900 1.99062900

C 0 -4.79194700 8.20845900 1.01015500

H 0 -3.69527700 8.21340800 1.08907100

H 0 -5.02616100 8.52010700 -0.01622800

C 0 -5.32621100 6.78747900 1.25240700

H 0 -6.41905400 6.79026500 1.15546000

H 0 -5.10266800 6.47773100 2.28251400

C 0 -4.79001800 5.71884600 0.29304600

H 0 -5.00123800 6.00566400 -0.74697100

H 0 -5.29568200 4.76804500 0.49109300

N 0 -3.34918100 5.50351400 0.47278000

H 0 -2.78708100 6.30392400 0.73176100

C 0 -2.67257500 4.42669300 0.03309500

N 0 -3.29984000 3.44700200 -0.63253400

H 0 -2.83400200 2.54610000 -0.76631400

H 0 -4.17344800 3.60103500 -1.11282800

N 0 -1.36127700 4.31878900 0.26565500

H 0 -0.89752400 4.73156800 1.07298100

H 0 -0.83128000 3.53378700 -0.13398100

C -1 -1.10290900 -1.27752500 7.54024700

H -1 -0.10448500 -0.90079800 7.37399300

H -1 -1.29606200 -1.48579000 8.58220500

H 0 -1.83798000 -0.55088000 7.17990400

C 0 -1.28406100 -2.66158800 6.88273400

H 0 -2.24779500 -3.08479700 7.19740300

H 0 -0.51652800 -3.34312300 7.27178800

C 0 -1.24253700 -2.69365000 5.39324400

N 0 -1.24124300 -3.88595400 4.67893700

C 0 -1.26605400 -1.70846200 4.44638400

H 0 -1.25271300 -0.64111300 4.55854600

C 0 -1.26814300 -3.64001700 3.36414000

H 0 -1.26107100 -4.36730300 2.56867900

N 0 -1.29662100 -2.31716100 3.20748400

H 0 -1.28922800 -1.82751400 2.28950200

C -1 3.85113500 6.18388000 2.45312900

H -1 4.73349700 6.66160100 2.05358600

H -1 3.38728200 6.70744200 3.27600300

H 0 3.10187700 6.16078300 1.65258400

C 0 4.16665900 4.72362100 2.87152400

H 0 5.09876100 4.66626800 3.44152400

H 0 3.34909000 4.35024500 3.49931600

C 0 4.23498300 3.82334300 1.64198800

O 0 3.15018000 3.65429400 1.01231000

O 0 5.34573000 3.32294900 1.27848200

C -1 12.04665600 -2.58751800 -0.50692700

H -1 13.09870100 -2.34673800 -0.54724000

H -1 11.80980000 -3.46953600 -1.08341900

H 0 11.82248500 -2.85090700 0.53660400

C 0 11.17312100 -1.38524900 -0.93530900

H 0 11.67205100 -0.83771000 -1.74719200

H 0 10.22410700 -1.74668400 -1.35187300

C 0 10.87959200 -0.42626900 0.23425400

H 0 10.12499300 -0.88304800 0.89073200

H 0 11.78445700 -0.32272400 0.84849900

C 0 10.45059100 0.99316700 -0.17122900

H 0 10.30864600 1.60736600 0.72824800

H 0 11.27224000 1.45717800 -0.73280300

C 0 9.20754200 1.13217600 -1.05119300

H 0 9.11985900 2.16782400 -1.39021900

H 0 9.25490300 0.49407400 -1.93678200

N 0 7.91834500 0.81401700 -0.34775800

H 0 7.14425600 1.36446700 -0.78418600

H 0 7.63417400 -0.18198700 -0.42462800

H 0 7.90481500 1.10972500 0.65706600

Cl 0 6.29455200 -1.83517800 -0.98294300

O 0 2.29516600 1.18868400 -3.34395800

H 0 3.71377000 2.36570600 -3.63128500

O 0 0.32684400 2.23783100 -0.40683800

O 0 0.67468200 3.94996600 2.26077400

H 0 0.32707900 3.05256800 2.12264600

H 0 1.53319500 3.93593900 1.77341800

O 0 -0.08153300 -0.08035100 -4.39437600

H 0 0.75293400 0.37232600 -4.10991300

H 0 0.09466400 -1.03398300 -4.30517600

O 0 -3.69910600 -1.13027400 -4.33013700

H 0 -4.33379000 -1.55893600 -3.71215800

H 0 -3.09846100 -1.86532700 -4.57599500

O 0 -4.64766600 -2.68115900 -2.25736100

H 0 -3.83135400 -2.35323200 -1.81546200

H 0 -5.18623600 -3.06470000 -1.53678500

O 0 2.62767600 3.79373600 -1.55202800

H 0 2.99451000 3.71507900 -0.62480900

H 0 4.38027600 -1.26929700 -2.14058100

O 0 0.74157600 -1.11435500 -1.76367500

O 0 6.00047700 2.65417200 -1.20719000

H 0 5.57829300 2.85118600 -0.31660400

H 0 6.52253400 3.45318600 -1.39250800

O 0 4.38117400 3.06600300 -3.44039600

H 0 4.96179200 2.67534000 -2.75008800

H 0 3.37335100 3.73289900 -2.21353800

O 0 7.47846700 1.90400600 2.18978500

H 0 6.71899400 2.50677000 1.98833800

H 0 7.15036800 1.30895600 2.88101200

O 0 3.52903500 -1.21507800 -2.62860400

H 0 2.81876500 0.39579000 -3.05491500

H 0 2.85026700 -1.44225800 -1.97369600

H 0 -1.22406500 -4.81294300 5.08828300

**^14^L_S_4_**

E (IEFPCM/B3LYP-D3/SDD,6-31G(d)) = -5854.301180 au
H (IEFPCM/B3LYP-D3/SDD,6-31G(d)) = -5852.744007 au
G (IEFPCM/B3LYP-D3/SDD,6-31G(d)) = -5852.979982 au
E (IEFPCM/B3LYP-D3/SDD,6-311+G(2d,p)//IEFPCM/B3LYP-D3/SDD,6-31G(d)) = -5856.124573 au

Mn 0 1.14159300 2.28645500 -1.83701800

Mn 0 -0.97999800 -2.42776800 -0.88272200

Mn 0 -0.14355800 0.85629000 0.13325900

Mn 0 -2.65908200 -0.52130000 0.33761900

O 0 0.24858300 0.68648700 -1.59162600

O 0 -1.94470900 1.00063300 -0.32425500

O 0 -2.56853400 -1.45342400 -1.20312400

O 0 -0.91184900 -1.08952400 0.59030200

Ca 0 -1.87683000 0.29701600 -2.71483500

C -1 0.56066800 4.55698600 -5.61336500

H -1 0.62547500 5.48958000 -5.07244900

H -1 0.75549600 4.63502900 -6.67253300

H 0 1.36935700 3.93211500 -5.21026300

C 0 -0.77032600 3.83438000 -5.34099800

H 0 -0.89808600 2.99427000 -6.03420100

H 0 -1.62920900 4.49731400 -5.51841600

C 0 -0.92587500 3.24267700 -3.92775400

O 0 -1.83562100 2.37355900 -3.77187000

O 0 -0.15098900 3.64156900 -3.00691400

C -1 -2.93811700 -6.69106500 -5.39621300

H -1 -1.97818800 -7.15695500 -5.56321200

H -1 -3.66657200 -6.82911600 -6.18151100

H 0 -3.35751000 -7.21829300 -4.52494400

C 0 -2.69458000 -5.24239900 -5.00862700

H 0 -3.61095400 -4.77663900 -4.62234300

H 0 -2.36912700 -4.63892100 -5.86245800

C 0 -1.60970200 -5.22515500 -3.92880000

H 0 -0.64089700 -5.50017800 -4.36987100

H 0 -1.82800200 -5.95883800 -3.14564300

C 0 -1.47310200 -3.85726300 -3.31020600

O 0 -1.50530500 -2.85698500 -4.03845200

O 0 -1.35965300 -3.89446100 -2.01511900

C -1 5.02783600 -4.67774400 0.97520200

H 0 5.80732800 -5.42033300 1.18367100

H 0 5.33494700 -4.11916500 0.08738900

C 0 4.89633900 -3.74872400 2.17928000

O 0 4.30443300 -4.12293000 3.21337000

C 0 3.69269200 -5.44061900 0.72246800

H 0 3.51687200 -6.10345500 1.57839300

H 0 3.80477600 -6.07292700 -0.16268800

C 0 2.50129900 -4.54211600 0.53734300

N 0 2.23632600 -3.55244100 1.46869000

H 0 2.72963300 -3.46579400 2.35359700

C 0 1.52238200 -4.38811600 -0.42152700

H 0 1.35130000 -4.94208600 -1.33170700

C 0 1.19176000 -2.82336700 1.05461800

H 0 0.87387100 -1.90932300 1.51590400

N 0 0.70741100 -3.31959100 -0.07273400

N 0 5.31921500 -2.48656500 2.00152600

H 0 5.88417200 -2.28030100 1.17165800

C -1 4.99927600 -1.40651900 2.92795400

H 0 5.88561400 -0.76506600 3.01272400

H 0 4.80991600 -1.84811900 3.90864300

C 0 3.79124500 -0.56937200 2.47136300

H 0 3.60193900 0.21053300 3.21999700

H 0 2.89977800 -1.19882700 2.45545500

C 0 4.00001400 0.06237200 1.09455700

H 0 4.44228700 -0.66698900 0.40322300

H 0 4.70535000 0.89993000 1.14531800

C 0 2.76094300 0.57596700 0.37020800

O 0 2.94777000 1.15806700 -0.72062900

O 0 1.61178900 0.31474800 0.88949000

C -1 -5.48068100 -4.01037900 2.16325400

H 0 -5.36217800 -3.25631300 2.94866700

H 0 -5.96871800 -4.88073300 2.62040500

C 0 -4.11835800 -4.43172100 1.61117200

H 0 -4.24518100 -5.15069200 0.79656400

H 0 -3.54882900 -4.94851000 2.39370700

C 0 -3.22265000 -3.31593600 1.07188600

O 0 -3.58808800 -2.10825200 1.24664500

O 0 -2.14750300 -3.67543300 0.52233400

C 0 -6.40914500 -3.48370600 1.07443400

O 0 -6.26241100 -3.77120000 -0.12129000

N 0 -7.45483400 -2.74026700 1.50906600

H 0 -7.47006600 -2.43904100 2.47412700

C -1 -8.44384400 -2.16430500 0.60458600

H 0 -9.39272200 -2.06297000 1.13277500

H 0 -8.56528900 -2.84682100 -0.24021800

C 0 -8.02087300 -0.76061000 0.12574200

O 0 -8.53653800 0.25344200 0.60005600

N 0 -7.05195400 -0.75015100 -0.82139600

H 0 -6.61209000 -1.61818500 -1.11157400

C 0 -6.44812600 0.47057300 -1.31778500

H 0 -6.65591900 0.60401200 -2.38350900

H 0 -6.89494900 1.30568800 -0.76974500

C 0 -4.92355900 0.49962800 -1.13524700

O 0 -4.22229800 0.93318700 -2.06585800

O 0 -4.50509500 0.07121100 0.00717000

C -1 -3.45160800 3.60052900 4.96287900

H -1 -3.48661100 3.30185300 6.00016800

H -1 -4.26560200 4.24797500 4.67201300

H 0 -2.54896900 4.22322200 4.86941500

C 0 -3.37512400 2.48136200 3.92901900

H 0 -3.48227100 2.93616600 2.93532200

H 0 -4.20535700 1.77394100 4.02924700

C 0 -2.03928500 1.73127400 3.98025000

H 0 -2.06327200 1.00495700 4.80095200

H 0 -1.21080300 2.41907500 4.17094200

C 0 -1.76852800 1.03142900 2.66418600

O 0 -0.65167800 1.25302900 2.11169700

O 0 -2.69044800 0.28474300 2.20380300

C -1 -5.50633500 9.02753200 1.98914800

H -1 -5.37413100 8.61418300 2.97812200

H -1 -6.54038700 9.26810100 1.79110700

H 0 -4.94785400 9.97069700 1.94797400

C 0 -4.95798900 8.03434900 0.95883300

H 0 -3.86575300 7.99767200 1.07499200

H 0 -5.14020900 8.39112200 -0.06333300

C 0 -5.56446200 6.63350800 1.13040000

H 0 -6.63519000 6.66693900 0.89505000

H 0 -5.47937300 6.31930400 2.17974800

C 0 -4.91480100 5.56171100 0.25272800

H 0 -5.00091800 5.83532500 -0.80932200

H 0 -5.42478300 4.60319900 0.39727100

N 0 -3.51220400 5.39335900 0.62941800

H 0 -3.02019300 6.21913600 0.94094700

C 0 -2.72444300 4.36982300 0.23848500

N 0 -3.19185600 3.39503200 -0.54087200

H 0 -2.70705100 2.48194900 -0.57075600

H 0 -4.04635100 3.48997400 -1.06745700

N 0 -1.45718100 4.32278200 0.64591000

H 0 -1.12620100 4.79054100 1.47989100

H 0 -0.80718600 3.61507800 0.24294800

C -1 -1.02487000 -1.37741400 7.49467800

H -1 -0.03486200 -0.98223800 7.32107900

H -1 -1.20721400 -1.58704800 8.53830800

H 0 -1.76934300 -0.65487300 7.14393000

C 0 -1.17731800 -2.74112400 6.78843100

H 0 -2.15273400 -3.17592900 7.04675500

H 0 -0.42410100 -3.43340200 7.18717800

C 0 -1.06478400 -2.71940900 5.29725300

N 0 -1.03339100 -3.88814900 4.54313900

C 0 -1.04012800 -1.70515200 4.38046900

H 0 -1.02103800 -0.64322300 4.53020200

C 0 -1.00039100 -3.59473400 3.23632300

H 0 -0.98100100 -4.29249600 2.41503900

N 0 -1.01720900 -2.26990100 3.11971300

H 0 -1.02504800 -1.76344500 2.19704300

C -1 3.75368400 6.16533400 2.35987000

H -1 4.62421300 6.65878900 1.95354900

H -1 3.28541400 6.68180500 3.18472100

H 0 2.99889900 6.12849400 1.56663000

C 0 4.12030700 4.71042400 2.74375400

H 0 5.06820100 4.66292000 3.28943800

H 0 3.32787600 4.29188600 3.37468200

C 0 4.17519100 3.86105500 1.47484300

O 0 3.08989600 3.77788300 0.84045100

O 0 5.27097000 3.33146300 1.10075200

C -1 12.09409000 -2.45592600 -0.63636400

H -1 13.14110500 -2.19538800 -0.68410200

H -1 11.87018800 -3.34350100 -1.20949000

H 0 11.87917700 -2.71890700 0.40914200

C 0 11.19629000 -1.27236500 -1.06221000

H 0 11.64004700 -0.76653600 -1.93214400

H 0 10.21832300 -1.65155500 -1.38062500

C 0 10.97888400 -0.26070900 0.07800900

H 0 10.27301200 -0.69564500 0.79892100

H 0 11.92234100 -0.11802800 0.62377600

C 0 10.49936100 1.13393600 -0.36106000

H 0 10.40773900 1.78539800 0.51897200

H 0 11.27141600 1.58568800 -0.99852700

C 0 9.19488000 1.21005800 -1.15664800

H 0 9.04364200 2.23161000 -1.51672900

H 0 9.20910800 0.54610400 -2.02467600

N 0 7.97748200 0.85401700 -0.35984700

H 0 7.13115500 1.23943900 -0.83236000

H 0 7.86265100 -0.18611400 -0.27333600

H 0 7.96110200 1.29060100 0.59196700

Cl 0 7.53781600 -2.23036400 -0.34138700

O 0 1.88160500 1.60408900 -3.48806200

H 0 3.37436100 2.44517000 -3.63608900

O 0 0.37728000 2.53988700 -0.09848900

O 0 0.80574900 3.77142600 2.53996900

H 0 0.37820400 2.92289500 2.32408800

H 0 1.50702900 3.82684700 1.85287800

O 0 -0.16564400 -0.12452600 -4.42271800

H 0 0.52226800 0.55090200 -4.19114400

H 0 0.09160100 -0.92915100 -3.92626400

O 0 -3.40612900 -0.97006500 -4.18860600

H 0 -4.12989300 -1.32791900 -3.63907400

H 0 -2.80085500 -1.74055900 -4.30146500

O 0 -4.81852800 -2.49146000 -2.16921900

H 0 -3.99154400 -2.17898100 -1.71824800

H 0 -5.20180900 -3.14303700 -1.55203600

O 0 2.39114700 3.90268100 -1.66445000

H 0 2.77802600 3.79830400 -0.74220700

H 0 2.79618900 -0.40252700 -1.80251000

O 0 -0.08469900 -1.67033100 -2.05534500

O 0 5.89093900 2.47153800 -1.35126900

H 0 5.45100500 2.71474100 -0.48301000

H 0 6.39881100 3.27041800 -1.57193200

O 0 4.14328300 3.06176600 -3.47232700

H 0 4.69194500 2.60756600 -2.79520600

H 0 3.13159500 3.75715800 -2.32648200

O 0 7.49809400 2.17595500 2.07348400

H 0 6.68591500 2.69339100 1.82515700

H 0 7.19063500 1.54859100 2.74576600

O 0 2.52909200 -1.06533900 -2.46500400

H 0 2.20193400 0.70259000 -3.25987500

H 0 1.58899600 -1.26009100 -2.21576600

H 0 -1.03075300 -4.82914000 4.91735900

**^16^L_S_4_^*^**

E (IEFPCM/B3LYP-D3/SDD,6-31G(d)) = -5854.276411 au

H (IEFPCM/B3LYP-D3/SDD,6-31G(d)) = -5852.725686 au

G (IEFPCM/B3LYP-D3/SDD,6-31G(d)) = -5852.966698 au

E (IEFPCM/B3LYP-D3/SDD,6-311+G(2d,p)//IEFPCM/B3LYP-D3/SDD,6-31G(d)) = -5856.099931 au

Mn 0 1.10145200 2.04'95800 -1.91307000

Mn 0 -1.05801300 -2.52967800 -0.82395300

Mn 0 -0.22348900 0.83472500 0.20538500

Mn 0 -2.69464400 -0.48080500 0.42327100

O 0 0.17958300 0.43216300 -1.49634400

O 0 -2.00324700 1.01095700 -0.30244400

O 0 -2.61463700 -1.45546500 -1.08845500

O 0 -0.96423600 -0.96639300 0.79491000

Ca 0 -1.93947300 0.18527600 -2.72247300

C -1 0.49662900 4.33602800 -5.77206000

H -1 0.54791700 5.29204300 -5.27213300

H -1 0.68924600 4.37103100 -6.83392500

H 0 1.31212000 3.74115600 -5.34089700

C 0 -0.82449800 3.60211400 -5.47132300

H 0 -0.92884300 2.72613300 -6.12384800

H 0 -1.69707900 4.23473800 -5.68509000

C 0 -0.97390100 3.06270400 -4.03631000

O 0 -1.94272800 2.27600600 -3.81072800

O 0 -0.11971100 3.41395700 -3.16880700

C -1 -2.83233600 -6.94323600 -5.06248600

H -1 -1.86566700 -7.40148200 -5.21070100

H -1 -3.55938300 -7.12607600 -5.83989700

H 0 -3.23060400 -7.38030900 -4.13666800

C 0 -2.52760000 -5.46080800 -4.84269100

H 0 -3.28422900 -4.98864100 -4.20249600

H 0 -2.51729900 -4.90342500 -5.78575000

C 0 -1.13438800 -5.36975400 -4.18986000

H 0 -0.36572900 -5.52033400 -4.95657300

H 0 -1.02631700 -6.14843000 -3.42807100

C 0 -0.97093800 -4.01713300 -3.54649700

O 0 -0.84603400 -3.01951800 -4.29742300

O 0 -1.07807200 -4.02417000 -2.27112200

C -1 5.10852800 -4.53665500 1.20296300

H 0 5.88074900 -5.26629000 1.47436700

H 0 5.44608100 -4.02129200 0.30037600

C 0 4.93441300 -3.55063900 2.35465400

O 0 4.30585300 -3.87259700 3.38449600

C 0 3.78295800 -5.31599200 0.94227000

H 0 3.58494600 -5.94127600 1.82144500

H 0 3.92681300 -5.98750800 0.09112100

C 0 2.58539100 -4.44271700 0.68043500

N 0 2.28092300 -3.41020100 1.55196000

H 0 2.75196800 -3.26832800 2.44172800

C 0 1.61805300 -4.36396900 -0.29910700

H 0 1.47624400 -4.97081400 -1.18043100

C 0 1.22378400 -2.73349500 1.08345400

H 0 0.87007000 -1.81101800 1.49722100

N 0 0.76894400 -3.29632200 -0.02659400

N 0 5.36821400 -2.29917900 2.13344100

H 0 5.96113100 -2.13166400 1.31426200

C -1 5.03263000 -1.18471100 3.01229500

H 0 5.89633100 -0.50786300 3.02986100

H 0 4.89489400 -1.57869100 4.02201800

C 0 3.78254800 -0.41741900 2.55861600

H 0 3.57657000 0.38312900 3.28026300

H 0 2.91554900 -1.08050200 2.58583100

C 0 3.95453200 0.16149300 1.15725400

H 0 4.44627000 -0.56647400 0.50019500

H 0 4.60620200 1.04331800 1.17177200

C 0 2.69326900 0.56537000 0.41187800

O 0 2.85982400 1.00029400 -0.75021500

O 0 1.56013700 0.38412000 0.99396600

C -1 -5.40769600 -3.97603500 2.37791000

H 0 -5.27891600 -3.18358900 3.12212700

H 0 -5.87528900 -4.83047800 2.88229600

C 0 -4.04867000 -4.41096300 1.80619000

H 0 -4.18483600 -5.19096600 1.05242700

H 0 -3.44005300 -4.85325700 2.60507400

C 0 -3.20705800 -3.30536800 1.16196900

O 0 -3.53716900 -2.10502400 1.38091900

O 0 -2.18791600 -3.68280400 0.49374700

C 0 -6.34634000 -3.50810200 1.26872500

O 0 -6.18733500 -3.83538700 0.08573800

N 0 -7.40301200 -2.76350800 1.67406000

H 0 -7.42333900 -2.42279600 2.62573500

C -1 -8.39978800 -2.24382800 0.74554400

H 0 -9.35042400 -2.13632800 1.26973600

H 0 -8.51021100 -2.96616200 -0.06706400

C 0 -8.00637800 -0.85665500 0.20135700

O 0 -8.52523200 0.16907200 0.64736900

N 0 -7.06678600 -0.87490200 -0.77363100

H 0 -6.60699700 -1.74557900 -1.02687700

C 0 -6.48830100 0.33162700 -1.33083500

H 0 -6.68781200 0.39897500 -2.40405000

H 0 -6.95937300 1.18448900 -0.83276000

C 0 -4.96833200 0.40465500 -1.13249700

O 0 -4.26361100 0.79672400 -2.08107000

O 0 -4.55683500 0.05477400 0.03823400

C -1 -3.49066900 3.77855400 4.84211900

H -1 -3.52018100 3.52459700 5.89142100

H -1 -4.31459000 4.40052000 4.52480300

H 0 -2.58960900 4.39547900 4.70981900

C 0 -3.43161100 2.61034700 3.87552800

H 0 -3.48772600 3.01623800 2.85692100

H 0 -4.29595800 1.94642800 3.98263100

C 0 -2.13644500 1.80279100 4.01496700

H 0 -2.25452700 1.05293400 4.80417500

H 0 -1.29062100 2.44637300 4.27306000

C 0 -1.85146800 1.13663500 2.69310500

O 0 -0.74565900 1.39663900 2.13030800

O 0 -2.77751900 0.39589500 2.23340500

C -1 -5.62965500 9.04024800 1.63951700

H -1 -5.49030000 8.67215200 2.64524600

H -1 -6.66739900 9.25648700 1.43291000

H 0 -5.09193700 9.99403900 1.56667500

C 0 -5.05406200 8.03081300 0.64260600

H 0 -3.96260100 8.01643200 0.77072200

H 0 -5.23364500 8.35932100 -0.38964400

C 0 -5.63491900 6.62471100 0.84229000

H 0 -6.71050500 6.63950800 0.62695000

H 0 -5.52452100 6.32353100 1.89301400

C 0 -4.98757000 5.55590800 -0.03958300

H 0 -5.10797600 5.81522600 -1.10191400

H 0 -5.47663700 4.59090900 0.12925400

N 0 -3.57291000 5.41038400 0.29844900

H 0 -3.07009000 6.24547700 0.56397900

C 0 -2.80996000 4.34679500 -0.02943300

N 0 -3.31239500 3.34401200 -0.75029700

H 0 -2.83739900 2.42889300 -0.73636500

H 0 -4.14001000 3.44379500 -1.31648100

N 0 -1.54641700 4.28750600 0.38853000

H 0 -1.20542500 4.81377300 1.18335600

H 0 -0.89186600 3.55493300 0.04008400

C -1 -0.98689400 -1.04811900 7.58316200

H -1 -0.00311000 -0.64603900 7.39102400

H -1 -1.16506400 -1.21506000 8.63518200

H 0 -1.73879500 -0.34629500 7.20612300

C 0 -1.11986300 -2.42919200 6.91191900

H 0 -2.09292000 -2.86935800 7.16917900

H 0 -0.36221000 -3.10642500 7.32835700

C 0 -0.99569000 -2.42124200 5.42176700

N 0 -1.06905200 -3.58686800 4.66782900

C 0 -0.86270600 -1.41332700 4.50520400

H 0 -0.72863800 -0.35823500 4.65630700

C 0 -0.99735800 -3.29183600 3.36116700

H 0 -1.02856100 -3.99269100 2.54231300

N 0 -0.88480800 -1.97379500 3.24297400

H 0 -0.90944200 -1.46733600 2.28775500

C -1 3.67272500 6.33590800 2.11891700

H -1 4.53534300 6.82429200 1.69021200

H -1 3.19752900 6.88054600 2.92138500

H 0 2.91789300 6.26356300 1.32732800

C 0 4.04319900 4.89698400 2.55553300

H 0 4.98825900 4.87261200 3.10761000

H 0 3.24935900 4.50196800 3.20007200

C 0 4.10930300 3.98528900 1.32882900

O 0 3.03451200 3.86559800 0.68267200

O 0 5.20976500 3.43207700 1.00258100

C -1 12.13901100 -2.28106600 -0.51465400

H -1 13.18194100 -2.00717700 -0.57531100

H -1 11.92793700 -3.19588900 -1.04845300

H 0 11.93121100 -2.50437600 0.54155700

C 0 11.22481000 -1.12656700 -0.98110800

H 0 11.65612800 -0.64938200 -1.87321500

H 0 10.24867600 -1.52868500 -1.27561600

C 0 11.00735700 -0.07606300 0.12308700

H 0 10.32210500 -0.49694000 0.87160700

H 0 11.95682600 0.10420000 0.64688800

C 0 10.49245100 1.29144900 -0.35806600

H 0 10.39768900 1.97113900 0.50004600

H 0 11.24486500 1.73763600 -1.02235600

C 0 9.17558300 1.30793500 -1.13548700

H 0 8.98639200 2.31488400 -1.51822300

H 0 9.19860100 0.62325000 -1.98706300

N 0 7.98221000 0.93314200 -0.31244800

H 0 7.11713700 1.26611100 -0.79287600

H 0 7.90932200 -0.10623200 -0.18504700

H 0 7.95404800 1.40579900 0.62097600

Cl 0 7.63822100 -2.16175600 -0.16837000

O 0 1.81357800 1.29474600 -3.52483400

H 0 3.34519000 2.07551200 -3.71310000

O 0 0.32453700 2.47667600 -0.23592600

O 0 0.69902500 3.95675700 2.32678300

H 0 0.28219300 3.08366300 2.21369900

H 0 1.40984400 3.94261900 1.64771800

O 0 -0.33597100 -0.12319400 -4.59806000

H 0 0.43518600 0.43353900 -4.31113300

H 0 -0.10824800 -1.04107800 -4.34765600

O 0 -3.11555400 -1.46524100 -4.10152700

H 0 -3.75497300 -1.97547900 -3.56118500

H 0 -2.35998000 -2.08021400 -4.26426100

O 0 -4.82490900 -2.53625000 -2.05694700

H 0 -4.04133200 -2.15301900 -1.57624000

H 0 -5.10075800 -3.28821900 -1.50207600

O 0 2.38223200 3.67155700 -1.84660300

H 0 2.75054600 3.66557100 -0.91306500

H 0 2.69578100 -0.65215500 -1.77269000

O 0 -0.21336600 -1.63458100 -2.21861800

O 0 5.83479600 2.39136700 -1.37576500

H 0 5.40773800 2.71223700 -0.52627200

H 0 6.28960400 3.18241600 -1.71025600

O 0 4.13383200 2.67303100 -3.58634100

H 0 4.65221000 2.26416100 -2.85999300

H 0 3.13037900 3.46928700 -2.48061800

O 0 7.43518100 2.34057300 2.06203600

H 0 6.62331000 2.83800300 1.77605600

H 0 7.12059300 1.73854100 2.75392900

O 0 2.47850000 -1.25808200 -2.50534600

H 0 2.12461700 0.39005400 -3.28036800

H 0 1.51891700 -1.45200100 -2.35392000

H 0 -1.15322100 -4.52434800 5.04132100

**^16^TS_1_**

E (IEFPCM/B3LYP-D3/SDD,6-31G(d)) = -5854.266986 au

H (IEFPCM/B3LYP-D3/SDD,6-31G(d)) = -5852.715863 au

G (IEFPCM/B3LYP-D3/SDD,6-31G(d)) = -5852.954583 au

E (IEFPCM/B3LYP-D3/SDD,6-311+G(2d,p)//IEFPCM/B3LYP-D3/SDD,6-31G(d)) = -5856.090957 au

Mn 0 1.13048200 1.86815100 -1.98667200

Mn 0 -1.00175600 -2.42021600 -0.81940700

Mn 0 -0.25544400 0.82658300 0.29409800

Mn 0 -2.72985100 -0.46213000 0.42896900

O 0 0.13748000 0.21046100 -1.45677600

O 0 -2.03334900 1.02460200 -0.28159600

O 0 -2.57975600 -1.42884700 -1.10119700

O 0 -1.01323600 -0.95769700 0.86906400

Ca 0 -1.98168700 0.20428800 -2.74413400

C -1 0.51386900 4.34131700 -5.78378700

H -1 0.56587100 5.29861900 -5.28640300

H -1 0.70711900 4.37326500 -6.84563400

H 0 1.32284000 3.73888300 -5.35267100

C 0 -0.81968600 3.62100700 -5.50323800

H 0 -0.96226800 2.79795000 -6.21545900

H 0 -1.68072100 4.28648300 -5.65081400

C 0 -0.95088500 2.98019100 -4.11143700

O 0 -1.97744400 2.27389900 -3.88479500

O 0 -0.01469800 3.17610600 -3.27976500

C -1 -2.82721800 -6.93253700 -5.04577900

H -1 -1.86094300 -7.39218300 -5.19221700

H -1 -3.55401800 -7.11671100 -5.82310700

H 0 -3.22717400 -7.37084500 -4.12102500

C 0 -2.53336800 -5.44752100 -4.82358200

H 0 -3.31484100 -4.97507200 -4.21368300

H 0 -2.49364500 -4.89566100 -5.76906700

C 0 -1.16572300 -5.33737900 -4.12374000

H 0 -0.37201100 -5.51667300 -4.85916800

H 0 -1.08471400 -6.09275900 -3.33523600

C 0 -1.00774100 -3.96423900 -3.51805200

O 0 -0.94394900 -2.98250900 -4.30028400

O 0 -1.04255300 -3.93744300 -2.24035700

C -1 5.11262400 -4.51736500 1.21765900

H 0 5.86371900 -5.26149000 1.50754800

H 0 5.47772100 -4.01055600 0.32089000

C 0 4.93172300 -3.52595600 2.36221600

O 0 4.30486700 -3.84550500 3.39412800

C 0 3.77715800 -5.27235600 0.92833600

H 0 3.56174000 -5.91220000 1.79285100

H 0 3.92107300 -5.92918400 0.06590500

C 0 2.58993600 -4.38221100 0.67374400

N 0 2.26693800 -3.38810100 1.58343300

H 0 2.73258700 -3.27539000 2.48124200

C 0 1.64454400 -4.25969700 -0.32258100

H 0 1.51798800 -4.82877200 -1.23067800

C 0 1.22095300 -2.69024100 1.12278600

H 0 0.85386700 -1.79054100 1.57292800

N 0 0.79131300 -3.20358900 -0.02162600

N 0 5.35841800 -2.27385300 2.13221400

H 0 5.95601500 -2.10949800 1.31562600

C -1 5.03919500 -1.16049300 3.01793300

H 0 5.90399700 -0.48470700 3.02011200

H 0 4.92190200 -1.55621800 4.02981500

C 0 3.78202900 -0.39280900 2.59373600

H 0 3.57808800 0.39283800 3.33182100

H 0 2.92080100 -1.06318500 2.61883300

C 0 3.93809200 0.20929100 1.20041200

H 0 4.47835900 -0.47985300 0.54077800

H 0 4.53928700 1.12664400 1.22949600

C 0 2.66046400 0.54829800 0.45450300

O 0 2.80299400 0.87982300 -0.74635400

O 0 1.54384100 0.43310000 1.07913200

C -1 -5.40367000 -3.94267200 2.38516000

H 0 -5.26879300 -3.14489900 3.12213000

H 0 -5.87115300 -4.79262300 2.89745900

C 0 -4.04769200 -4.38648800 1.80713800

H 0 -4.19268600 -5.16884500 1.05716300

H 0 -3.43713400 -4.82912300 2.60398300

C 0 -3.20882900 -3.28191900 1.15254600

O 0 -3.58163100 -2.08633400 1.34362300

O 0 -2.17162300 -3.64108600 0.50974600

C 0 -6.34642100 -3.48365300 1.27608900

O 0 -6.20694400 -3.84049000 0.09870700

N 0 -7.38788900 -2.71498200 1.67467600

H 0 -7.39575000 -2.35445700 2.61918400

C -1 -8.39304200 -2.21175500 0.74645600

H 0 -9.33932600 -2.09653300 1.27718800

H 0 -8.50989600 -2.94849600 -0.05209500

C 0 -8.00793000 -0.83523000 0.17415200

O 0 -8.52077400 0.19858000 0.60797600

N 0 -7.08305700 -0.87226600 -0.81427000

H 0 -6.61628700 -1.74544700 -1.04453500

C 0 -6.50194200 0.32386400 -1.38855300

H 0 -6.68626200 0.36840800 -2.46557600

H 0 -6.98267600 1.18560500 -0.91558000

C 0 -4.98602700 0.40633700 -1.17007500

O 0 -4.27428800 0.80855900 -2.10961600

O 0 -4.58572800 0.05467300 0.00255700

C -1 -3.47996700 3.81651800 4.82959900

H -1 -3.51033200 3.56541500 5.87956400

H -1 -4.30306100 4.43848400 4.51014800

H 0 -2.56254300 4.40017300 4.67209500

C 0 -3.49055400 2.62309300 3.89538300

H 0 -3.48444700 3.00470000 2.86594800

H 0 -4.41487200 2.04353400 3.99346700

C 0 -2.28035400 1.70313900 4.10150100

H 0 -2.52835200 0.91528400 4.81951200

H 0 -1.41428600 2.25883500 4.47272700

C 0 -1.94414200 1.11174400 2.75940600

O 0 -0.82928400 1.41249600 2.24164800

O 0 -2.85973800 0.39600300 2.23614600

C -1 -5.61168200 9.07180100 1.61166100

H -1 -5.47327500 8.70626700 2.61845400

H -1 -6.64908400 9.28856200 1.40388700

H 0 -5.07312200 10.02462500 1.53431900

C 0 -5.02826200 8.05070900 0.62815800

H 0 -3.93419600 8.06767400 0.73266100

H 0 -5.23974800 8.34343200 -0.40879900

C 0 -5.56233700 6.63503700 0.88360600

H 0 -6.64683100 6.61874400 0.71798500

H 0 -5.39751900 6.36118500 1.93471100

C 0 -4.93560900 5.55846800 -0.00354800

H 0 -5.10649700 5.79535600 -1.06427800

H 0 -5.40770700 4.59298200 0.20591300

N 0 -3.50409600 5.42813600 0.26973400

H 0 -2.99314500 6.27233900 0.48840100

C 0 -2.75338900 4.36961400 -0.10474200

N 0 -3.29111700 3.35959600 -0.78856800

H 0 -2.83918300 2.43149800 -0.74524600

H 0 -4.17851600 3.43527400 -1.25987600

N 0 -1.45693900 4.33048900 0.21009500

H 0 -1.08011400 4.80000900 1.02690000

H 0 -0.85007300 3.55945700 -0.14122300

C -1 -0.98275000 -1.00536500 7.58501800

H -1 0.00156000 -0.60482400 7.39235500

H -1 -1.16168400 -1.16929200 8.63738200

H 0 -1.73657200 -0.31001600 7.20119600

C 0 -1.11248900 -2.39719700 6.93548700

H 0 -2.08015100 -2.83915000 7.20982400

H 0 -0.34669400 -3.06274500 7.35596200

C 0 -1.00453000 -2.40977700 5.44574100

N 0 -1.08440000 -3.58380400 4.70624700

C 0 -0.88846700 -1.41113600 4.51707000

H 0 -0.75522100 -0.35368600 4.65491700

C 0 -1.03359600 -3.30047700 3.39517100

H 0 -1.07160000 -4.00857300 2.58291200

N 0 -0.92854600 -1.98387100 3.26156400

H 0 -0.96852900 -1.47797000 2.29266400

C -1 3.68761100 6.35910500 2.10357700

H -1 4.55097600 6.84543900 1.67404800

H -1 3.21253100 6.90639100 2.90431000

H 0 2.93273800 6.29792300 1.30981100

C 0 4.03771900 4.91233400 2.53140400

H 0 4.97847100 4.87429800 3.09000200

H 0 3.23490100 4.52464500 3.16911100

C 0 4.10645800 3.99886200 1.30407300

O 0 3.03036200 3.84188000 0.66771500

O 0 5.22015700 3.47503500 0.96993900

C -1 12.14641100 -2.27370200 -0.50204600

H -1 13.18966000 -2.00106000 -0.56285100

H -1 11.93468700 -3.18973700 -1.03350300

H 0 11.93846300 -2.49471800 0.55467600

C 0 11.23740700 -1.11583400 -0.96788000

H 0 11.66419500 -0.64769600 -1.86692400

H 0 10.25482200 -1.51098000 -1.25017800

C 0 11.04242000 -0.05779000 0.13336300

H 0 10.36041900 -0.46789200 0.89088700

H 0 11.99895600 0.11410000 0.64697000

C 0 10.53775800 1.31280800 -0.34898300

H 0 10.45645000 1.99702300 0.50688000

H 0 11.28893100 1.74893300 -1.02127900

C 0 9.21523600 1.33648700 -1.11567600

H 0 9.02757000 2.34396400 -1.49775700

H 0 9.22830200 0.65092900 -1.96679700

N 0 8.02677200 0.96713500 -0.28374800

H 0 7.15926500 1.29292700 -0.76615700

H 0 7.95389700 -0.07094800 -0.14810300

H 0 7.99766600 1.44909300 0.64426900

Cl 0 7.64563500 -2.12372200 -0.14766300

O 0 1.87636700 1.05916400 -3.54344500

H 0 3.40691500 1.87349900 -3.73084900

O 0 0.31763400 2.39184500 -0.38273700

O 0 0.65749800 3.99647400 2.24055300

H 0 0.25228000 3.11201000 2.19787000

H 0 1.39310700 3.94197700 1.58896500

O 0 -0.38945700 -0.17726300 -4.62934700

H 0 0.43295500 0.29597800 -4.33716400

H 0 -0.24714200 -1.11821300 -4.39177400

O 0 -3.19552700 -1.43576600 -4.09161600

H 0 -3.83485500 -1.93687300 -3.54309000

H 0 -2.45385900 -2.06570100 -4.26651800

O 0 -4.80107400 -2.56010000 -2.00906100

H 0 -4.00425400 -2.18268600 -1.54865700

H 0 -5.10018700 -3.28368300 -1.42813300

O 0 2.43011000 3.51768500 -1.89841000

H 0 2.76832300 3.54379700 -0.95805800

H 0 2.81661100 -0.82412600 -1.71315200

O 0 -0.11816600 -1.45353600 -2.17897900

O 0 5.86754000 2.37140500 -1.37072600

H 0 5.44933800 2.73153200 -0.53182500

H 0 6.29400600 3.15074100 -1.76440600

O 0 4.19094400 2.47805500 -3.61838000

H 0 4.70672200 2.09814100 -2.87613500

H 0 3.19253300 3.30600600 -2.50748100

O 0 7.43715900 2.39568200 2.07126600

H 0 6.63073600 2.88934300 1.76533900

H 0 7.10700300 1.79381500 2.75604100

O 0 2.57946000 -1.43664300 -2.43417500

H 0 2.19728500 0.16422600 -3.27570100

H 0 1.60642700 -1.56975600 -2.29326000

H 0 -1.16070400 -4.51724300 5.09110100

**^8^L_S_4_**

E (IEFPCM/B3LYP-D3/SDD,6-31G(d)) = -5854.301193 au

H (IEFPCM/B3LYP-D3/SDD,6-31G(d)) = -5852.746722 au

G (IEFPCM/B3LYP-D3/SDD,6-31G(d)) = -5852.985951 au

E (IEFPCM/B3LYP-D3/SDD,6-311+G(2d,p)//IEFPCM/B3LYP-D3/SDD,6-31G(d)) = -5856.125903 au

Mn 0 1.11255200 2.26484600 -1.83414400

Mn 0 -0.91235300 -2.43519900 -0.89300000

Mn 0 -0.16614000 0.84300800 0.13696100

Mn 0 -2.65306400 -0.54470500 0.31814200

O 0 0.20645800 0.66114100 -1.58705700

O 0 -1.96736900 1.00070300 -0.30916300

O 0 -2.49935900 -1.44631300 -1.23819100

O 0 -0.90232400 -1.09559700 0.58408900

Ca 0 -1.95548900 0.38621600 -2.71710700

C -1 0.55340100 4.57182800 -5.62778500

H -1 0.61432300 5.50451600 -5.08657800

H -1 0.74830400 4.65094500 -6.68685900

H 0 1.35847200 3.94488300 -5.22277000

C 0 -0.78137700 3.85464600 -5.36694600

H 0 -0.90639600 3.01455600 -6.06068600

H 0 -1.63716300 4.51917000 -5.55217500

C 0 -0.94911500 3.26468700 -3.95684700

O 0 -1.89158500 2.42946700 -3.79839300

O 0 -0.15243900 3.62681300 -3.04199900

C -1 -2.90094400 -6.69003500 -5.41512100

H -1 -1.93911900 -7.15207700 -5.58190400

H -1 -3.62856200 -6.83074100 -6.20072400

H 0 -3.31964600 -7.22018300 -4.54521100

C 0 -2.66726700 -5.23978900 -5.02493200

H 0 -3.59219200 -4.77490700 -4.65902800

H 0 -2.32613400 -4.63700300 -5.87324200

C 0 -1.60604100 -5.22100100 -3.92334700

H 0 -0.62783800 -5.49934600 -4.34164100

H 0 -1.84062100 -5.95485300 -3.14489700

C 0 -1.47180200 -3.85736400 -3.29469200

O 0 -1.53267000 -2.84646800 -4.00457100

O 0 -1.31760400 -3.90836800 -2.00456900

C -1 5.05467100 -4.64705300 0.95976900

H 0 5.85398400 -5.37377000 1.14830200

H 0 5.32840800 -4.08260500 0.06498000

C 0 4.93373200 -3.72055100 2.16735500

O 0 4.36670500 -4.10368400 3.21207800

C 0 3.72808600 -5.43434400 0.74264700

H 0 3.58209200 -6.09398800 1.60645900

H 0 3.82965900 -6.06990100 -0.14145800

C 0 2.52283200 -4.55176800 0.57699200

N 0 2.25962600 -3.56211100 1.50928600

H 0 2.76279300 -3.46899300 2.38786500

C 0 1.53825800 -4.40294500 -0.37602400

H 0 1.36511000 -4.95550600 -1.28657600

C 0 1.21109200 -2.83569800 1.09886800

H 0 0.89589900 -1.91885400 1.55670500

N 0 0.72350500 -3.33720500 -0.02396100

N 0 5.33380000 -2.45203500 1.98229200

H 0 5.89133000 -2.24099400 1.14877100

C -1 5.01246200 -1.37652700 2.91344500

H 0 5.89343400 -0.72680900 2.99197300

H 0 4.83625100 -1.82176600 3.89491900

C 0 3.79353500 -0.55025500 2.46854700

H 0 3.60189900 0.22615800 3.22018700

H 0 2.90841100 -1.18838600 2.45627000

C 0 3.98865100 0.08558100 1.09235500

H 0 4.43190900 -0.63926800 0.39678400

H 0 4.68762400 0.92854600 1.13977300

C 0 2.74168900 0.58887500 0.37631800

O 0 2.92127600 1.17832500 -0.71229800

O 0 1.59680500 0.31352500 0.89486600

C -1 -5.45683500 -4.02159900 2.14418500

H 0 -5.33308500 -3.26396600 2.92524300

H 0 -5.94781100 -4.88745300 2.60674300

C 0 -4.09870200 -4.45020000 1.58880900

H 0 -4.23052300 -5.17017300 0.77577700

H 0 -3.52824200 -4.96741800 2.37013100

C 0 -3.20428700 -3.33606900 1.04478100

O 0 -3.58459200 -2.12955400 1.20188600

O 0 -2.12144600 -3.68947700 0.51145500

C 0 -6.38554400 -3.49524800 1.05461800

O 0 -6.23547000 -3.77738800 -0.14204000

N 0 -7.43484700 -2.75733600 1.48952200

H 0 -7.45445700 -2.46029700 2.45579300

C -1 -8.42671100 -2.18681200 0.58496600

H 0 -9.37882300 -2.10061700 1.11029500

H 0 -8.53729500 -2.86596700 -0.26397300

C 0 -8.01987400 -0.77706500 0.11603100

O 0 -8.54192600 0.22946100 0.59906600

N 0 -7.05724300 -0.75322300 -0.83669900

H 0 -6.60548800 -1.61567900 -1.12473200

C 0 -6.45996000 0.47498200 -1.31901000

H 0 -6.67216700 0.62357500 -2.38189500

H 0 -6.90567500 1.30224400 -0.75812500

C 0 -4.93656600 0.50247400 -1.14168700

O 0 -4.24572200 0.97723400 -2.06215400

O 0 -4.50739000 0.03051400 -0.02396600

C -1 -3.45889800 3.59647100 4.94672400

H -1 -3.49309600 3.29736100 5.98391400

H -1 -4.27534100 4.24077600 4.65574700

H 0 -2.55496300 4.21637600 4.84908700

C 0 -3.39051500 2.47325700 3.91522900

H 0 -3.47509900 2.92891200 2.92014100

H 0 -4.23724000 1.78433600 4.00672800

C 0 -2.07307100 1.69163300 3.98478500

H 0 -2.13192300 0.95571400 4.79522100

H 0 -1.23371100 2.35893100 4.19926700

C 0 -1.79228600 0.99886900 2.66645100

O 0 -0.67042500 1.22232600 2.12379700

O 0 -2.70876200 0.25251000 2.19528400

C -1 -5.53399600 9.01615800 1.97379600

H -1 -5.40051700 8.60305100 2.96270000

H -1 -6.56892000 9.25269300 1.77544700

H 0 -4.97941900 9.96178700 1.93406700

C 0 -4.97823800 8.02481100 0.94712000

H 0 -3.88810900 7.97555300 1.07817100

H 0 -5.14088700 8.38718200 -0.07627600

C 0 -5.60180900 6.63015300 1.10217600

H 0 -6.66037400 6.66842500 0.81821700

H 0 -5.56766000 6.32155600 2.15616400

C 0 -4.91852400 5.55042100 0.26308200

H 0 -4.92593400 5.83429600 -0.79972700

H 0 -5.45918400 4.60306000 0.36416400

N 0 -3.54826800 5.34495900 0.73302700

H 0 -3.07056300 6.14309300 1.12788600

C 0 -2.76056700 4.31729600 0.35236200

N 0 -3.20309800 3.40353700 -0.51204900

H 0 -2.74440300 2.47784200 -0.54507800

H 0 -4.03319900 3.54134400 -1.06632000

N 0 -1.53686300 4.18855000 0.86119900

H 0 -1.22043200 4.69835900 1.67456300

H 0 -0.84606500 3.53135000 0.43635000

C -1 -1.01340900 -1.37256200 7.47798400

H -1 -0.02490800 -0.97342300 7.30485800

H -1 -1.19530000 -1.58321600 8.52148700

H 0 -1.76127600 -0.65469900 7.12488200

C 0 -1.15915900 -2.73954400 6.77800900

H 0 -2.13084100 -3.17936700 7.04185500

H 0 -0.40016600 -3.42566900 7.17664200

C 0 -1.05386400 -2.72202000 5.28736100

N 0 -1.04702300 -3.89264200 4.53615300

C 0 -1.01604000 -1.70984200 4.36886900

H 0 -0.97160400 -0.64810800 4.51612700

C 0 -1.01609900 -3.60250900 3.22871100

H 0 -1.00994500 -4.30126600 2.40810100

N 0 -1.01223500 -2.27780300 3.10956200

H 0 -1.01641800 -1.77242900 2.18634200

C -1 3.73713700 6.19050300 2.34707200

H -1 4.60585400 6.68751400 1.94120900

H -1 3.26652800 6.70488100 3.17190000

H 0 2.98256500 6.14857300 1.55388500

C 0 4.11193300 4.73741400 2.73245500

H 0 5.06450000 4.69568200 3.27051700

H 0 3.32689200 4.31693500 3.37128800

C 0 4.15988800 3.88235500 1.46671900

O 0 3.06892700 3.78794200 0.84397100

O 0 5.25594800 3.35857100 1.08474600

C -1 12.11266600 -2.39684000 -0.64859000

H -1 13.15865900 -2.13214900 -0.69587200

H -1 11.89248400 -3.28512900 -1.22205100

H 0 11.89692000 -2.65919100 0.39684100

C 0 11.20990200 -1.21830700 -1.07897700

H 0 11.65257200 -0.71324100 -1.94994400

H 0 10.23426500 -1.60343400 -1.39733500

C 0 10.98466600 -0.20374400 0.05679600

H 0 10.28218500 -0.64134600 0.77919900

H 0 11.92690500 -0.05072600 0.60192800

C 0 10.49306400 1.18483800 -0.38852100

H 0 10.39698300 1.83967100 0.48853100

H 0 11.26071300 1.63996400 -1.02893900

C 0 9.18701300 1.24684300 -1.18311900

H 0 9.02764100 2.26549700 -1.54787100

H 0 9.20550100 0.57896600 -2.04803800

N 0 7.97310700 0.88543400 -0.38328000

H 0 7.12318100 1.26435900 -0.85447400

H 0 7.86569600 -0.15527400 -0.29301700

H 0 7.95608100 1.32505300 0.56707200

Cl 0 7.56951100 -2.20498700 -0.34570700

O 0 1.85831700 1.58217300 -3.47465200

H 0 3.34051100 2.44265000 -3.63653000

O 0 0.33706900 2.53504600 -0.10031500

O 0 0.83369500 3.67908100 2.60243000

H 0 0.40520800 2.83034300 2.38858800

H 0 1.50413800 3.76187800 1.88842800

O 0 -0.21848200 -0.17935600 -4.32636300

H 0 0.47862900 0.49516200 -4.12836900

H 0 0.00913000 -0.95060900 -3.75637300

O 0 -3.41368900 -0.93965000 -4.18807900

H 0 -4.13888800 -1.30961700 -3.64800500

H 0 -2.79555100 -1.70254900 -4.28783100

O 0 -4.77600500 -2.48393600 -2.17250900

H 0 -3.93671000 -2.17966300 -1.73961400

H 0 -5.16186600 -3.12093100 -1.54173900

O 0 2.35376000 3.88848200 -1.65981000

H 0 2.74553700 3.78677600 -0.73954100

H 0 2.89342300 -0.36466300 -1.80169800

O 0 0.07188100 -1.75660800 -2.04318400

O 0 5.86931600 2.48751500 -1.36605800

H 0 5.43131500 2.73169500 -0.49727000

H 0 6.37025400 3.28923100 -1.59224500

O 0 4.10495000 3.06583400 -3.47727400

H 0 4.66215600 2.61484800 -2.80503000

H 0 3.09249100 3.74901200 -2.32500200

O 0 7.49379300 2.21507200 2.04636800

H 0 6.67829300 2.72835400 1.79974400

H 0 7.19094300 1.58650800 2.71961600

O 0 2.64501100 -1.02619900 -2.47284400

H 0 2.20025600 0.68644700 -3.24898800

H 0 1.72087100 -1.27471400 -2.21185400

H 0 -1.05971700 -4.83233200 4.91334300

**^2^L_S_4_**

E (IEFPCM/B3LYP-D3/SDD,6-31G(d)) = -5854.303767 au

H (IEFPCM/B3LYP-D3/SDD,6-31G(d)) = -5852.749759 au

G (IEFPCM/B3LYP-D3/SDD,6-31G(d)) = -5852.988803 au

E (IEFPCM/B3LYP-D3/SDD,6-311+G(2d,p)//IEFPCM/B3LYP-D3/SDD,6-31G(d)) = -5856.128479 au

Mn 0 1.13522900 2.29243200 -1.83466800

Mn 0 -0.98080900 -2.42380800 -0.88702700

Mn 0 -0.15181600 0.85993500 0.13327700

Mn 0 -2.67172400 -0.51905200 0.34113900

O 0 0.25568100 0.69336600 -1.57693200

O 0 -1.95305900 0.99885100 -0.32013900

O 0 -2.57551100 -1.46165800 -1.19508000

O 0 -0.92096600 -1.08320000 0.58437700

Ca 0 -1.86411900 0.27424000 -2.71115600

C -1 0.56203900 4.51149800 -5.66132400

H -1 0.62687100 5.44845500 -5.12800300

H -1 0.75660700 4.58097100 -6.72113600

H 0 1.37287100 3.89251700 -5.25451400

C 0 -0.76593100 3.79017800 -5.37179400

H 0 -0.89761400 2.94030900 -6.05200600

H 0 -1.62794100 4.44859300 -5.55047900

C 0 -0.90597700 3.21730500 -3.94905800

O 0 -1.81671800 2.35567200 -3.76611100

O 0 -0.11212700 3.62583100 -3.04729700

C -1 -2.93543300 -6.73480700 -5.35213300

H -1 -1.97549100 -7.20193000 -5.51557700

H -1 -3.66405900 -6.87930200 -6.13611200

H 0 -3.35457600 -7.25055700 -4.47410800

C 0 -2.68809100 -5.28143100 -4.98499600

H 0 -3.60258300 -4.80820000 -4.60392400

H 0 -2.36379600 -4.69078600 -5.84822900

C 0 -1.59956200 -5.25107700 -3.90875200

H 0 -0.63231000 -5.53089300 -4.35004700

H 0 -1.81531000 -5.97480700 -3.11580200

C 0 -1.46335200 -3.87504300 -3.30861300

O 0 -1.48828300 -2.88586100 -4.05210600

O 0 -1.36246900 -3.89428200 -2.01182500

C -1 5.03181000 -4.66901000 1.00084500

H 0 5.80393800 -5.41513100 1.22328500

H 0 5.35055600 -4.12101100 0.11059200

C 0 4.89578200 -3.72810800 2.19471300

O 0 4.29862900 -4.09165300 3.22964400

C 0 3.69304300 -5.42556500 0.74394700

H 0 3.50903300 -6.08280900 1.60250400

H 0 3.80727500 -6.06362400 -0.13681800

C 0 2.50526100 -4.52406200 0.54683300

N 0 2.23926400 -3.52617500 1.46907200

H 0 2.73007900 -3.43267900 2.35460900

C 0 1.52795800 -4.37723900 -0.41506200

H 0 1.35796400 -4.93915600 -1.32058100

C 0 1.19641000 -2.79961600 1.04685800

H 0 0.87938400 -1.88092700 1.49915100

N 0 0.71324900 -3.30468500 -0.07709800

N 0 5.31966600 -2.46786100 2.00644200

H 0 5.89306700 -2.27033500 1.18018400

C -1 5.00334700 -1.38206000 2.92701300

H 0 5.88943900 -0.73913700 3.00218600

H 0 4.82069700 -1.81723800 3.91194200

C 0 3.79200100 -0.54789800 2.47489600

H 0 3.60480500 0.23253600 3.22349500

H 0 2.90094100 -1.17805800 2.46362800

C 0 3.99543200 0.08220600 1.09708000

H 0 4.43731100 -0.64757400 0.40599700

H 0 4.69938800 0.92125100 1.14436500

C 0 2.75383800 0.59196000 0.37499700

O 0 2.93896400 1.16492500 -0.72085900

O 0 1.60682100 0.33634600 0.90149000

C -1 -5.47650000 -3.99319000 2.18595400

H 0 -5.35404700 -3.22949700 2.96121900

H 0 -5.96492600 -4.85686900 2.65529100

C 0 -4.11676900 -4.42377800 1.63452200

H 0 -4.24726400 -5.14883900 0.82592100

H 0 -3.54687800 -4.93572900 2.41996100

C 0 -3.22178200 -3.31209700 1.08532800

O 0 -3.59191100 -2.10467300 1.25399600

O 0 -2.14662000 -3.67312600 0.53725500

C 0 -6.40750000 -3.47943600 1.09276400

O 0 -6.26733200 -3.78620100 -0.09881400

N 0 -7.44824000 -2.72500600 1.52099100

H 0 -7.45567200 -2.40645800 2.48055900

C -1 -8.44024000 -2.16014300 0.61307500

H 0 -9.38775400 -2.05311600 1.14268300

H 0 -8.56343800 -2.85294000 -0.22307400

C 0 -8.02112800 -0.76170500 0.11485800

O 0 -8.54356200 0.25672500 0.57250300

N 0 -7.05008500 -0.76049100 -0.82984400

H 0 -6.60389400 -1.62969800 -1.10738500

C 0 -6.44851200 0.45553900 -1.34089900

H 0 -6.65316800 0.57378000 -2.40905400

H 0 -6.89916700 1.29690600 -0.80567100

C 0 -4.92479000 0.49113800 -1.15334500

O 0 -4.22011200 0.91855700 -2.08402100

O 0 -4.51061400 0.07333200 -0.00507800

C -1 -3.44761400 3.64039400 4.92328600

H -1 -3.48233700 3.35013500 5.96297100

H -1 -4.26174900 4.28537000 4.62737400

H 0 -2.54251500 4.25810000 4.82139200

C 0 -3.37940800 2.50850900 3.90328500

H 0 -3.46954500 2.95274600 2.90379600

H 0 -4.22191700 1.81604800 4.00516500

C 0 -2.05700800 1.73657800 3.97644600

H 0 -2.10639100 1.00989900 4.79583900

H 0 -1.22039500 2.41101500 4.17868500

C 0 -1.78070700 1.03032600 2.66545900

O 0 -0.65498700 1.23129400 2.12356700

O 0 -2.70808100 0.29348500 2.20044900

C -1 -5.50365600 9.04287800 1.90613500

H -1 -5.37117000 8.63757700 2.89839700

H -1 -6.53778200 9.28171900 1.70639700

H 0 -4.94432300 9.98526300 1.85801600

C 0 -4.95433200 8.04148300 0.88440900

H 0 -3.86642100 7.97414600 1.02586600

H 0 -5.10085400 8.40816900 -0.13992700

C 0 -5.60250400 6.65626600 1.02865200

H 0 -6.65751600 6.71376300 0.73493800

H 0 -5.58376400 6.34330200 2.08174100

C 0 -4.93020800 5.56669700 0.19130000

H 0 -4.91981800 5.85553200 -0.87005500

H 0 -5.48955900 4.62906100 0.28132300

N 0 -3.56986600 5.33472000 0.67651900

H 0 -3.08768600 6.12148500 1.08826000

C 0 -2.79019000 4.29622800 0.30925100

N 0 -3.22354300 3.38745100 -0.56485900

H 0 -2.75804700 2.46440300 -0.60421300

H 0 -4.04040900 3.53486700 -1.13604300

N 0 -1.58141400 4.15056500 0.85076600

H 0 -1.26614700 4.68764300 1.64661300

H 0 -0.87651900 3.51439400 0.42296700

C -1 -1.01971500 -1.31658800 7.49478900

H -1 -0.02979300 -0.92272400 7.31775400

H -1 -1.20178800 -1.51777300 8.54012800

H 0 -1.76389300 -0.59664300 7.13797500

C 0 -1.17011200 -2.68404300 6.79651200

H 0 -2.14654000 -3.11725200 7.05360000

H 0 -0.41839400 -3.37447600 7.20142700

C 0 -1.05318700 -2.67020400 5.30557400

N 0 -1.04890800 -3.84340800 4.55789800

C 0 -1.00099900 -1.66153600 4.38350300

H 0 -0.94884000 -0.59955900 4.52715600

C 0 -1.00686900 -3.55792400 3.24968400

H 0 -1.00419000 -4.25991500 2.43173200

N 0 -0.99181400 -2.23386400 3.12568100

H 0 -1.00110200 -1.73436300 2.19875800

C -1 3.75677200 6.18480000 2.29784500

H -1 4.62714800 6.67504000 1.88732800

H -1 3.28864000 6.70789100 3.11859300

H 0 3.00189100 6.12707900 1.50601900

C 0 4.13613900 4.74129100 2.71714000

H 0 5.08642800 4.71832500 3.26033100

H 0 3.34990400 4.33270300 3.36205800

C 0 4.19347100 3.85978400 1.47182500

O 0 3.09870400 3.72212100 0.86367400

O 0 5.29936700 3.35969900 1.08653400

C -1 12.09743200 -2.45955400 -0.63037200

H -1 13.14440500 -2.19929600 -0.68047100

H -1 11.87349200 -3.35177300 -1.19622900

H 0 11.88067600 -2.71151800 0.41736800

C 0 11.20155400 -1.27980000 -1.07077300

H 0 11.64418500 -0.78839600 -1.94956600

H 0 10.22144900 -1.66103500 -1.37986600

C 0 10.99051600 -0.25083800 0.05461100

H 0 10.28743500 -0.67446900 0.78465300

H 0 11.93651800 -0.10021900 0.59382900

C 0 10.50870300 1.13670100 -0.40383200

H 0 10.42200200 1.80204500 0.46624100

H 0 11.27668100 1.57846100 -1.05312500

C 0 9.19913100 1.19908900 -1.19228900

H 0 9.04262000 2.21555800 -1.56429400

H 0 9.20978100 0.52422900 -2.05189400

N 0 7.98820800 0.84985500 -0.38261100

H 0 7.13785000 1.23023900 -0.85150800

H 0 7.87639700 -0.18933800 -0.28240000

H 0 7.97949500 1.29721200 0.56434700

Cl 0 7.57268200 -2.24024300 -0.30615700

O 0 1.89078600 1.59430100 -3.47732200

H 0 3.36725100 2.45815800 -3.62578300

O 0 0.33781500 2.54361800 -0.11296000

O 0 0.83297900 3.69761900 2.58533500

H 0 0.40911600 2.84386800 2.38250800

H 0 1.52211100 3.76018700 1.88711500

O 0 -0.14793700 -0.12757300 -4.42509700

H 0 0.54077400 0.54456900 -4.18437500

H 0 0.10841700 -0.93800800 -3.93828300

O 0 -3.38469100 -0.99395400 -4.18372800

H 0 -4.10905900 -1.35057300 -3.63427400

H 0 -2.77815800 -1.76420100 -4.29505200

O 0 -4.82828000 -2.49916200 -2.14988600

H 0 -3.99848900 -2.18730000 -1.70276700

H 0 -5.20012100 -3.16276900 -1.53868500

O 0 2.38914000 3.91482900 -1.63982500

H 0 2.77901400 3.78972600 -0.72253200

H 0 2.78196700 -0.41025800 -1.78197000

O 0 -0.09873700 -1.66111500 -2.06812600

O 0 5.89862000 2.47000000 -1.36054700

H 0 5.46748500 2.72049700 -0.48997900

H 0 6.41003000 3.26453100 -1.58863200

O 0 4.13289500 3.07907100 -3.46077300

H 0 4.68721600 2.62125900 -2.79075600

H 0 3.12604800 3.77096900 -2.30632800

O 0 7.53307500 2.19731100 2.04080000

H 0 6.72171300 2.71772200 1.79704100

H 0 7.22674400 1.57160100 2.71517700

O 0 2.52897500 -1.08253900 -2.44039100

H 0 2.21915000 0.69843000 -3.24179800

H 0 1.58468200 -1.27547800 -2.20633000

H 0 -1.06967600 -4.78191100 4.93771100

**^2'^L_S_4_**

E (IEFPCM/B3LYP-D3/SDD,6-31G(d)) = -5854.305055 au

H (IEFPCM/B3LYP-D3/SDD,6-31G(d)) = -5852.750968 au

G (IEFPCM/B3LYP-D3/SDD,6-31G(d)) = -5852.989354 au

E (IEFPCM/B3LYP-D3/SDD,6-311+G(2d,p)//IEFPCM/B3LYP-D3/SDD,6-31G(d)) = -5856.129766 au

Mn 0 1.13076600 2.28119100 -1.83242800

Mn 0 -0.97405000 -2.41857400 -0.88591000

Mn 0 -0.15881300 0.85605100 0.13684000

Mn 0 -2.67059000 -0.51745800 0.33932100

O 0 0.24774200 0.67844800 -1.56892000

O 0 -1.96063800 1.00067500 -0.32712600

O 0 -2.57054600 -1.46183500 -1.19529700

O 0 -0.91908000 -1.08221600 0.58945600

Ca 0 -1.86812000 0.26893300 -2.71871700

C -1 0.55948500 4.50954400 -5.66319300

H -1 0.62382000 5.44691000 -5.13053000

H -1 0.75373100 4.57842300 -6.72310200

H 0 1.37077900 3.89117900 -5.25638900

C 0 -0.76810900 3.78705100 -5.37412200

H 0 -0.89911000 2.93799100 -6.05552200

H 0 -1.63050700 4.44507500 -5.55228400

C 0 -0.90881800 3.21181800 -3.95232000

O 0 -1.82118400 2.35130200 -3.77103400

O 0 -0.11421200 3.61670200 -3.04990600

C -1 -2.93027800 -6.73891600 -5.34539700

H -1 -1.97006300 -7.20549900 -5.50877000

H -1 -3.65900900 -6.88444200 -6.12908800

H 0 -3.34898000 -7.25483700 -4.46724500

C 0 -2.68469100 -5.28510300 -4.97845400

H 0 -3.60065800 -4.81195000 -4.60078700

H 0 -2.35777600 -4.69507200 -5.84114700

C 0 -1.60004700 -5.25335600 -3.89857600

H 0 -0.63193900 -5.53784700 -4.33511000

H 0 -1.82044500 -5.97345100 -3.10358900

C 0 -1.46053100 -3.87574000 -3.30241700

O 0 -1.48652200 -2.88794200 -4.04768100

O 0 -1.35453300 -3.89258000 -2.00601100

C -1 5.03720500 -4.66336700 1.00410000

H 0 5.80938200 -5.40910300 1.22770700

H 0 5.35650800 -4.11602000 0.11366300

C 0 4.90008800 -3.72161800 2.19710000

O 0 4.30287800 -4.08468400 3.23219600

C 0 3.69887700 -5.42042600 0.74666600

H 0 3.51445600 -6.07744800 1.60529500

H 0 3.81381500 -6.05867100 -0.13387100

C 0 2.51124500 -4.51902800 0.54880300

N 0 2.24380800 -3.52245200 1.47215100

H 0 2.73405900 -3.42961000 2.35812600

C 0 1.53601800 -4.37030900 -0.41477800

H 0 1.36750000 -4.93085000 -1.32142800

C 0 1.20198100 -2.79497900 1.04909500

H 0 0.88410600 -1.87711400 1.50239200

N 0 0.72105400 -3.29794600 -0.07681600

N 0 5.32303900 -2.46125100 2.00772000

H 0 5.89725600 -2.26448100 1.18185000

C -1 5.00701100 -1.37511900 2.92802500

H 0 5.89224200 -0.73064700 3.00015100

H 0 4.82783000 -1.80967300 3.91388900

C 0 3.79331300 -0.54353000 2.47832900

H 0 3.60641600 0.23721100 3.22668000

H 0 2.90327100 -1.17517300 2.46955100

C 0 3.99345800 0.08542800 1.09969200

H 0 4.43686300 -0.64382800 0.40907500

H 0 4.69518100 0.92644900 1.14540500

C 0 2.74976700 0.59091000 0.37858000

O 0 2.93341800 1.15763000 -0.72130000

O 0 1.60431700 0.33991000 0.90910400

C -1 -5.47125400 -3.99386100 2.19147000

H 0 -5.34937500 -3.23016600 2.96682700

H 0 -5.95875500 -4.85815200 2.66062100

C 0 -4.11113200 -4.42285100 1.63915000

H 0 -4.24139900 -5.14908700 0.83156000

H 0 -3.53993800 -4.93325400 2.42462800

C 0 -3.21785000 -3.31067100 1.08749000

O 0 -3.58994500 -2.10358700 1.25418300

O 0 -2.14239600 -3.67106700 0.53938400

C 0 -6.40260400 -3.48060000 1.09826000

O 0 -6.26250500 -3.78756200 -0.09332100

N 0 -7.44375400 -2.72674700 1.52626100

H 0 -7.45170700 -2.40809300 2.48578100

C -1 -8.43664300 -2.16390100 0.61810500

H 0 -9.38387600 -2.05686600 1.14824900

H 0 -8.56001000 -2.85807800 -0.21686800

C 0 -8.01899300 -0.76611700 0.11757800

O 0 -8.53897900 0.25292100 0.57655900

N 0 -7.05189600 -0.76632800 -0.83116000

H 0 -6.60590000 -1.63608100 -1.10709000

C 0 -6.45005400 0.44880500 -1.34367300

H 0 -6.65476500 0.56587500 -2.41193600

H 0 -6.90030200 1.29098300 -0.80937200

C 0 -4.92636900 0.48395100 -1.15628300

O 0 -4.22204500 0.91197400 -2.08693700

O 0 -4.51260800 0.06477500 -0.00840300

C -1 -3.44683800 3.64296900 4.92305000

H -1 -3.48109500 3.35339800 5.96294300

H -1 -4.26148700 4.28719000 4.62690800

H 0 -2.54144600 4.25987200 4.81963200

C 0 -3.38169400 2.51102400 3.90306700

H 0 -3.46697500 2.95675100 2.90374700

H 0 -4.22858500 1.82349000 4.00217400

C 0 -2.06432200 1.73046200 3.97876900

H 0 -2.12334900 0.99829300 4.79244400

H 0 -1.22442100 2.39825900 4.18945400

C 0 -1.78780100 1.03235800 2.66384000

O 0 -0.66476900 1.24115400 2.11954700

O 0 -2.71368200 0.29579600 2.19545900

C -1 -5.50732400 9.04199100 1.90273500

H -1 -5.37430700 8.63745900 2.89523900

H -1 -6.54166400 9.27999400 1.70310100

H 0 -4.94848200 9.98459600 1.85352600

C 0 -4.95839300 8.03970500 0.88178700

H 0 -3.87017400 7.97388800 1.02146700

H 0 -5.10725600 8.40433600 -0.14294900

C 0 -5.60484500 6.65416500 1.03007400

H 0 -6.66107300 6.71055200 0.74050800

H 0 -5.58164500 6.34246800 2.08345400

C 0 -4.93519400 5.56426400 0.19124500

H 0 -4.93070000 5.85144200 -0.87062600

H 0 -5.49277000 4.62600400 0.28570900

N 0 -3.57204600 5.33514800 0.66985200

H 0 -3.08887300 6.12344100 1.07750400

C 0 -2.79203700 4.29856000 0.29754800

N 0 -3.22943300 3.38881700 -0.57378500

H 0 -2.76495700 2.46559400 -0.61325300

H 0 -4.05146900 3.53366000 -1.13816600

N 0 -1.57868700 4.15739800 0.82908300

H 0 -1.26195800 4.68922000 1.62801000

H 0 -0.87707800 3.51513400 0.40359800

C -1 -1.01491300 -1.31060400 7.49731600

H -1 -0.02530300 -0.91619000 7.31975500

H -1 -1.19657800 -1.51119600 8.54284000

H 0 -1.75951000 -0.59118600 7.14025000

C 0 -1.16451400 -2.67820000 6.79922200

H 0 -2.14057200 -3.11207300 7.05659200

H 0 -0.41225400 -3.36811300 7.20404600

C 0 -1.04820700 -2.66484200 5.30814400

N 0 -1.04794100 -3.83851300 4.56122200

C 0 -0.99358600 -1.65692900 4.38525100

H 0 -0.93689300 -0.59501000 4.52808800

C 0 -1.00622200 -3.55399300 3.25278100

H 0 -1.00616700 -4.25653800 2.43529200

N 0 -0.98747300 -2.23010900 3.12769600

H 0 -0.99730900 -1.73148200 2.19959500

C -1 3.75514100 6.19046100 2.29400100

H -1 4.62507900 6.68101000 1.88292300

H -1 3.28686700 6.71379700 3.11451200

H 0 3.00006600 6.13227600 1.50236900

C 0 4.13465200 4.74702200 2.71336000

H 0 5.08509000 4.72404500 3.25629800

H 0 3.34860500 4.33871900 3.35872800

C 0 4.19145700 3.86451000 1.46858600

O 0 3.09670200 3.72684100 0.86055100

O 0 5.29707800 3.36324200 1.08386100

C -1 12.10090400 -2.45023900 -0.63046000

H -1 13.14768800 -2.18930500 -0.68100800

H -1 11.87742400 -3.34299600 -1.19564800

H 0 11.88456000 -2.70163800 0.41751400

C 0 11.20385000 -1.27165200 -1.07150100

H 0 11.64550500 -0.78073200 -1.95104700

H 0 10.22387800 -1.65407500 -1.37957200

C 0 10.99251400 -0.24178100 0.05293900

H 0 10.29123700 -0.66582500 0.78448900

H 0 11.93896700 -0.08880400 0.59071600

C 0 10.50755000 1.14441200 -0.40632100

H 0 10.42072100 1.81039700 0.46324800

H 0 11.27394400 1.58694200 -1.05696500

C 0 9.19699600 1.20391400 -1.19335500

H 0 9.03836900 2.21985000 -1.56591900

H 0 9.20785100 0.52842800 -2.05246900

N 0 7.98746000 0.85322900 -0.38224500

H 0 7.13589600 1.23078800 -0.85128800

H 0 7.87799600 -0.18601700 -0.28057300

H 0 7.97820700 1.30219000 0.56394400

Cl 0 7.57877400 -2.23793000 -0.30181100

O 0 1.88340200 1.58117100 -3.47725900

H 0 3.35973000 2.44461100 -3.62775100

O 0 0.33813100 2.54060700 -0.11373400

O 0 0.82715400 3.70650100 2.57839900

H 0 0.40170800 2.85371300 2.37509100

H 0 1.51795200 3.76678800 1.88161000

O 0 -0.15105300 -0.12899000 -4.43111900

H 0 0.53918100 0.54194400 -4.18849600

H 0 0.10526500 -0.94113700 -3.94734200

O 0 -3.39075400 -1.00324800 -4.18634200

H 0 -4.11343100 -1.36024800 -3.63484700

H 0 -2.78368300 -1.77276000 -4.29823800

O 0 -4.82165600 -2.50738400 -2.14730700

H 0 -3.99281300 -2.19256800 -1.70070900

H 0 -5.19541800 -3.16616500 -1.53201600

O 0 2.38487300 3.90544300 -1.64339800

H 0 2.77566600 3.78415200 -0.72610100

H 0 2.78629300 -0.41785400 -1.78105900

O 0 -0.09737600 -1.65476700 -2.07006300

O 0 5.89365700 2.46683500 -1.36189600

H 0 5.46402900 2.72012800 -0.49144200

H 0 6.40300000 3.26133800 -1.59461200

O 0 4.12636600 3.06491900 -3.46475300

H 0 4.68002500 2.60874200 -2.79318400

H 0 3.12088600 3.76006900 -2.31032200

O 0 7.53142000 2.20365000 2.03949200

H 0 6.71968300 2.72327500 1.79511500

H 0 7.22550300 1.57871300 2.71478100

O 0 2.53238500 -1.08660500 -2.44291900

H 0 2.21498500 0.68648400 -3.24118600

H 0 1.58797900 -1.28001100 -2.20934700

H 0 -1.07111800 -4.77669500 4.94169900

**^6^L_S_4_**

E (IEFPCM/B3LYP-D3/SDD,6-31G(d)) = -5854.304329 au

H (IEFPCM/B3LYP-D3/SDD,6-31G(d)) = -5852.750242 au

G (IEFPCM/B3LYP-D3/SDD,6-31G(d)) = -5852.990113 au

E (IEFPCM/B3LYP-D3/SDD,6-311+G(2d,p)//IEFPCM/B3LYP-D3/SDD,6-31G(d)) = -5856.129095 au

Mn 0 1.13555500 2.28954500 -1.83512500

Mn 0 -0.98639700 -2.42672300 -0.87886300

Mn 0 -0.15370700 0.86047000 0.13395800

Mn 0 -2.66721500 -0.51870800 0.33581900

O 0 0.24970800 0.69409700 -1.57643900

O 0 -1.95637500 0.99892400 -0.33025100

O 0 -2.57693200 -1.46208600 -1.19962100

O 0 -0.91667600 -1.08393300 0.58956900

Ca 0 -1.86465500 0.27016400 -2.71861900

C -1 0.56207700 4.51274000 -5.66071000

H -1 0.62675300 5.44970800 -5.12738900

H -1 0.75663300 4.58224500 -6.72052200

H 0 1.37280100 3.89363500 -5.25392300

C 0 -0.76607500 3.79108200 -5.37248600

H 0 -0.89796100 2.94285700 -6.05470100

H 0 -1.62788300 4.45012100 -5.54979800

C 0 -0.90676300 3.21496000 -3.95118300

O 0 -1.81786500 2.35309600 -3.77064100

O 0 -0.11347300 3.62133600 -3.04803900

C -1 -2.93352400 -6.73414700 -5.35151900

H -1 -1.97350500 -7.20111000 -5.51496300

H -1 -3.66212600 -6.87876300 -6.13549800

H 0 -3.35258000 -7.25037100 -4.47372300

C 0 -2.68604400 -5.28088700 -4.98312000

H 0 -3.60049900 -4.80804200 -4.60153400

H 0 -2.36198600 -4.68944800 -5.84592300

C 0 -1.59740200 -5.25147500 -3.90684500

H 0 -0.62999800 -5.53017600 -4.34855100

H 0 -1.81264400 -5.97692500 -3.11530600

C 0 -1.46126700 -3.87674500 -3.30313600

O 0 -1.48923500 -2.88524900 -4.04362400

O 0 -1.35611700 -3.89964200 -2.00693400

C -1 5.03337500 -4.66702400 1.00145900

H 0 5.80736700 -5.41167200 1.22243200

H 0 5.34961700 -4.11843000 0.11068900

C 0 4.89799700 -3.72634600 2.19560700

O 0 4.30151400 -4.09009100 3.23078100

C 0 3.69560600 -5.42611300 0.74701100

H 0 3.51377800 -6.08268500 1.60655500

H 0 3.80993200 -6.06503900 -0.13312600

C 0 2.50638900 -4.52643300 0.55022600

N 0 2.24362300 -3.52455500 1.46898700

H 0 2.73518400 -3.42866600 2.35367400

C 0 1.52523800 -4.38407500 -0.40850900

H 0 1.35195400 -4.94993400 -1.31096600

C 0 1.19875300 -2.80032300 1.04759900

H 0 0.88341800 -1.87968400 1.49717300

N 0 0.71143800 -3.31032100 -0.07231300

N 0 5.32075200 -2.46569600 2.00689300

H 0 5.89512600 -2.26836800 1.18129800

C -1 5.00436500 -1.38007900 2.92762700

H 0 5.89035600 -0.73700500 3.00282600

H 0 4.82204300 -1.81547900 3.91252000

C 0 3.79283900 -0.54589500 2.47613100

H 0 3.60616700 0.23462400 3.22477100

H 0 2.90161500 -1.17584100 2.46553000

C 0 3.99552800 0.08394300 1.09812100

H 0 4.43739700 -0.64586800 0.40709600

H 0 4.69927000 0.92320600 1.14494900

C 0 2.75336000 0.59288300 0.37655500

O 0 2.93775100 1.16195900 -0.72174700

O 0 1.60692700 0.34059100 0.90503300

C -1 -5.47504700 -3.99295200 2.18656800

H 0 -5.35264800 -3.22871800 2.96131300

H 0 -5.96296000 -4.85650800 2.65665100

C 0 -4.11556900 -4.42312000 1.63487100

H 0 -4.24587000 -5.14926700 0.82722100

H 0 -3.54462600 -4.93353800 2.42052800

C 0 -3.22275000 -3.31111700 1.08374700

O 0 -3.59101400 -2.10378100 1.25208100

O 0 -2.14892300 -3.67255700 0.53229100

C 0 -6.40665000 -3.48012900 1.09333100

O 0 -6.26669600 -3.78761900 -0.09806500

N 0 -7.44741000 -2.72577200 1.52158500

H 0 -7.45440500 -2.40664800 2.48097200

C -1 -8.43909200 -2.16039800 0.61368900

H 0 -9.38721200 -2.05505700 1.14260000

H 0 -8.56091900 -2.85205400 -0.22357700

C 0 -8.02032600 -0.76097400 0.11848400

O 0 -8.54070800 0.25657300 0.58039200

N 0 -7.05173100 -0.75803900 -0.82863700

H 0 -6.60602400 -1.62671100 -1.10877200

C 0 -6.44878400 0.45876700 -1.33607000

H 0 -6.65544800 0.58180100 -2.40329500

H 0 -6.89624600 1.29895600 -0.79634100

C 0 -4.92458900 0.48917600 -1.15156100

O 0 -4.22043100 0.91416600 -2.08379800

O 0 -4.51020700 0.06910900 -0.00422700

C -1 -3.44743100 3.64096900 4.92390000

H -1 -3.48210600 3.35070400 5.96358500

H -1 -4.26167400 4.28581000 4.62798800

H 0 -2.54201400 4.25805200 4.82150700

C 0 -3.38142000 2.50983800 3.90274300

H 0 -3.46971000 2.95601900 2.90387400

H 0 -4.22636100 1.82006900 4.00292300

C 0 -2.06185000 1.73272900 3.97529800

H 0 -2.11626100 1.00182600 4.79045700

H 0 -1.22316300 2.40305600 4.18273600

C 0 -1.78617600 1.03246100 2.66106200

O 0 -0.66265300 1.24011000 2.11672100

O 0 -2.71183700 0.29524900 2.19387900

C -1 -5.50437200 9.04311100 1.90674900

H -1 -5.37181900 8.63783200 2.89901100

H -1 -6.53853800 9.28178000 1.70701100

H 0 -4.94500600 9.98543200 1.85819200

C 0 -4.95548500 8.04086600 0.88562400

H 0 -3.86718600 7.97550900 1.02494400

H 0 -5.10483700 8.40527400 -0.13911500

C 0 -5.60120200 6.65499300 1.03435900

H 0 -6.65750700 6.71071300 0.74495900

H 0 -5.57768900 6.34358900 2.08781900

C 0 -4.93123000 5.56508300 0.19572400

H 0 -4.92679500 5.85208600 -0.86619600

H 0 -5.48855300 4.62670200 0.29040200

N 0 -3.56795600 5.33634800 0.67433200

H 0 -3.08504500 6.12493400 1.08175000

C 0 -2.78725300 4.30053200 0.30130900

N 0 -3.22435500 3.39071000 -0.57008600

H 0 -2.75880100 2.46834300 -0.61201200

H 0 -4.04656700 3.53525400 -1.13431400

N 0 -1.57353200 4.16033100 0.83220100

H 0 -1.25742700 4.69102300 1.63213400

H 0 -0.87184800 3.51829300 0.40674900

C -1 -1.01870800 -1.31560900 7.49540300

H -1 -0.02885100 -0.92158000 7.31836800

H -1 -1.20074700 -1.51682400 8.54074200

H 0 -1.76268900 -0.59524500 7.13891100

C 0 -1.16939600 -2.68222900 6.79569000

H 0 -2.14604200 -3.11535200 7.05208500

H 0 -0.41804900 -3.37345600 7.19998100

C 0 -1.05221800 -2.66700400 5.30467900

N 0 -1.05249900 -3.83968200 4.55620800

C 0 -0.99541100 -1.65797500 4.38313200

H 0 -0.93807200 -0.59628900 4.52738000

C 0 -1.00879500 -3.55334500 3.24815800

H 0 -1.00812200 -4.25494400 2.42984900

N 0 -0.98827300 -2.22937300 3.12481600

H 0 -0.99601500 -1.72923700 2.19675400

C -1 3.75653100 6.18657400 2.29845900

H -1 4.62682600 6.67695800 1.88794200

H -1 3.28831200 6.70958700 3.11920700

H 0 3.00161500 6.12963700 1.50660100

C 0 4.13536300 4.74250000 2.71604800

H 0 5.08543200 4.71844900 3.25957700

H 0 3.34877400 4.33330500 3.36016000

C 0 4.19293900 3.86235800 1.46964200

O 0 3.09881600 3.72708400 0.85985600

O 0 5.29851300 3.36071700 1.08536200

C -1 12.09862900 -2.45639300 -0.62975800

H -1 13.14555900 -2.19596100 -0.67985700

H -1 11.87483800 -3.34864900 -1.19561500

H 0 11.88153600 -2.70805600 0.41795300

C 0 11.20290400 -1.27674300 -1.07102500

H 0 11.64636800 -0.78521400 -1.94933700

H 0 10.22317800 -1.65812500 -1.38115100

C 0 10.99057100 -0.24797800 0.05422300

H 0 10.28717200 -0.67218800 0.78358000

H 0 11.93612500 -0.09684500 0.59408400

C 0 10.50800500 1.13922900 -0.40431700

H 0 10.42133600 1.80470100 0.46566200

H 0 11.27541000 1.58128400 -1.05408700

C 0 9.19796300 1.20051800 -1.19201300

H 0 9.04009200 2.21690700 -1.56367300

H 0 9.20874000 0.52584400 -2.05177100

N 0 7.98797400 0.84985200 -0.38159100

H 0 7.13687700 1.22800100 -0.85085200

H 0 7.87825500 -0.18941100 -0.27990100

H 0 7.97859100 1.29852500 0.56475200

Cl 0 7.57989200 -2.24147300 -0.29867400

O 0 1.89023700 1.58780300 -3.47721300

H 0 3.36780100 2.44950000 -3.62736700

O 0 0.34264800 2.54300200 -0.11234500

O 0 0.83052000 3.70460200 2.58000500

H 0 0.40481100 2.85192800 2.37671600

H 0 1.52023900 3.76540400 1.88220300

O 0 -0.14465900 -0.13177100 -4.42629300

H 0 0.54357100 0.54124800 -4.18550700

H 0 0.11289100 -0.94173200 -3.93965800

O 0 -3.38922000 -0.99732100 -4.18875900

H 0 -4.11430800 -1.35051000 -3.63811200

H 0 -2.78432700 -1.76917900 -4.29711400

O 0 -4.83359600 -2.49753700 -2.14954600

H 0 -4.00389100 -2.18562000 -1.70284300

H 0 -5.20464600 -3.16170600 -1.53839200

O 0 2.39091100 3.91043300 -1.64438900

H 0 2.78062600 3.78801400 -0.72656900

H 0 2.77834600 -0.41514100 -1.77758200

O 0 -0.10436000 -1.65940900 -2.05703700

O 0 5.89753600 2.46787700 -1.36059200

H 0 5.46697300 2.71999000 -0.49017500

H 0 6.40885000 3.26191300 -1.59056100

O 0 4.13376200 3.07042600 -3.46398800

H 0 4.68788100 2.61447000 -2.79256700

H 0 3.12769100 3.76487800 -2.31062600

O 0 7.53195300 2.19885700 2.04082700

H 0 6.72043000 2.71895500 1.79689800

H 0 7.22580400 1.57313300 2.71527400

O 0 2.52294000 -1.08895400 -2.43352200

H 0 2.21762300 0.69218400 -3.23971700

H 0 1.57851600 -1.27946500 -2.19775700

H 0 -1.07714700 -4.77829400 4.93552000

**^8'^L_S_4_**

E (IEFPCM/B3LYP-D3/SDD,6-31G(d)) = -5854.305043 au

H (IEFPCM/B3LYP-D3/SDD,6-31G(d)) = -5852.751010 au

G (IEFPCM/B3LYP-D3/SDD,6-31G(d)) = -5852.990208 au

E (IEFPCM/B3LYP-D3/SDD,6-311+G(2d,p)//IEFPCM/B3LYP-D3/SDD,6-31G(d)) = -5856.129787 au

Mn 0 1.12900300 2.29423300 -1.82803000

Mn 0 -0.98985200 -2.42039500 -0.88143800

Mn 0 -0.15350600 0.85329900 0.13463400

Mn 0 -2.67474700 -0.52185300 0.33850100

O 0 0.26261800 0.68400700 -1.57062200

O 0 -1.95367400 0.99049100 -0.32920800

O 0 -2.58523900 -1.46995900 -1.19579700

O 0 -0.92172600 -1.08178800 0.59152800

Ca 0 -1.85592600 0.26217900 -2.71452800

C -1 0.56199200 4.49837000 -5.67216200

H -1 0.62752500 5.43654200 -5.14106700

H -1 0.75577700 4.56532100 -6.73228000

H 0 1.37434900 3.88154400 -5.26484100

C 0 -0.76424900 3.77674800 -5.37579600

H 0 -0.89542800 2.92176800 -6.04972200

H 0 -1.62774900 4.43240800 -5.55748200

C 0 -0.90064200 3.21342400 -3.94850100

O 0 -1.80722500 2.34891300 -3.75912400

O 0 -0.10808600 3.63211200 -3.05005200

C -1 -2.93880800 -6.74607200 -5.33421300

H -1 -1.97913900 -7.21387900 -5.49730100

H -1 -3.66807500 -6.89215400 -6.11730200

H 0 -3.35735800 -7.25985400 -4.45475300

C 0 -2.68803300 -5.29235100 -4.96925600

H 0 -3.60021300 -4.81677000 -4.58544800

H 0 -2.36526000 -4.70364800 -5.83436600

C 0 -1.59516800 -5.26237700 -3.89676600

H 0 -0.63138600 -5.54960100 -4.34081200

H 0 -1.81203800 -5.98088500 -3.09941800

C 0 -1.44909500 -3.88339800 -3.30462800

O 0 -1.46413600 -2.89899500 -4.05510900

O 0 -1.35219600 -3.89563600 -2.00746600

C -1 5.03390900 -4.66807300 1.00790800

H 0 5.80337700 -5.41570100 1.23439700

H 0 5.35596800 -4.12348300 0.11673400

C 0 4.89742200 -3.72367900 2.19871800

O 0 4.29912800 -4.08378100 3.23409700

C 0 3.69353500 -5.42200000 0.74987600

H 0 3.50617500 -6.07624600 1.61002100

H 0 3.80840700 -6.06307400 -0.12862000

C 0 2.50768300 -4.51873400 0.54764700

N 0 2.24470900 -3.51529900 1.46467200

H 0 2.73450500 -3.41915500 2.35032600

C 0 1.52882100 -4.37531600 -0.41340400

H 0 1.35629100 -4.94236400 -1.31528200

C 0 1.20214600 -2.78966900 1.04017900

H 0 0.88713400 -1.86874500 1.48899800

N 0 0.71614100 -3.29951100 -0.08052200

N 0 5.32203700 -2.46407200 2.00708400

H 0 5.89337500 -2.26832000 1.17899900

C -1 5.00795000 -1.37665200 2.92646200

H 0 5.89447400 -0.73387000 2.99829700

H 0 4.82762900 -1.81021600 3.91252500

C 0 3.79581400 -0.54229200 2.47632700

H 0 3.61024200 0.23837600 3.22510200

H 0 2.90440300 -1.17197700 2.46736000

C 0 3.99635600 0.08759000 1.09782400

H 0 4.44014800 -0.64119000 0.40692800

H 0 4.69805300 0.92859900 1.14412000

C 0 2.75282200 0.59395700 0.37640300

O 0 2.93521500 1.16231400 -0.72224200

O 0 1.60701600 0.34073800 0.90716400

C -1 -5.47328300 -3.98615600 2.19941400

H 0 -5.34703300 -3.21940000 2.97101600

H 0 -5.96304400 -4.84651500 2.67342800

C 0 -4.11580500 -4.42255100 1.64638500

H 0 -4.24989400 -5.15118100 0.84160400

H 0 -3.54473700 -4.93173900 2.43277900

C 0 -3.22176100 -3.31392500 1.08974700

O 0 -3.58870100 -2.10602300 1.25910300

O 0 -2.15058300 -3.67693800 0.53430600

C 0 -6.40500800 -3.47440900 1.10589400

O 0 -6.26700300 -3.78524800 -0.08492000

N 0 -7.44421300 -2.71734100 1.53299000

H 0 -7.44998700 -2.39540300 2.49143600

C -1 -8.43763600 -2.15582000 0.62453200

H 0 -9.38338500 -2.04393000 1.15627100

H 0 -8.56462400 -2.85319000 -0.20724500

C 0 -8.01728600 -0.76107200 0.11706000

O 0 -8.53473400 0.26108800 0.57191600

N 0 -7.05134400 -0.76746500 -0.83301600

H 0 -6.60809700 -1.63957100 -1.10595900

C 0 -6.44769200 0.44405600 -1.35223800

H 0 -6.64967900 0.55362500 -2.42180800

H 0 -6.89935200 1.28989200 -0.82497100

C 0 -4.92435200 0.48149500 -1.16114800

O 0 -4.21755300 0.90629600 -2.09123300

O 0 -4.51337300 0.06784300 -0.00990200

C -1 -3.43990100 3.65311400 4.91747900

H -1 -3.47392700 3.36528000 5.95786100

H -1 -4.25405700 4.29766100 4.62068800

H 0 -2.53302400 4.26722100 4.81111300

C 0 -3.38049100 2.51802000 3.90024400

H 0 -3.46417800 2.96136900 2.89997200

H 0 -4.23088200 1.83518100 4.00211000

C 0 -2.06701200 1.73142400 3.97637700

H 0 -2.12942000 0.99937500 4.78996100

H 0 -1.22429700 2.39553900 4.18748800

C 0 -1.79129000 1.03030300 2.66250300

O 0 -0.66338900 1.22807500 2.12407500

O 0 -2.72153500 0.30011700 2.19232800

C -1 -5.49652100 9.04923500 1.88935600

H -1 -5.36341100 8.64619600 2.88245500

H -1 -6.53072300 9.28794200 1.68984800

H 0 -4.93702800 9.99144900 1.83935500

C 0 -4.94768000 8.04681200 0.86797100

H 0 -3.86064600 7.97413000 1.01367800

H 0 -5.08830100 8.41674200 -0.15605100

C 0 -5.60268300 6.66396700 1.00515300

H 0 -6.65651300 6.72777400 0.70843400

H 0 -5.58871800 6.34689100 2.05708500

C 0 -4.93331100 5.57391100 0.16535200

H 0 -4.91693100 5.86726800 -0.89463000

H 0 -5.49836600 4.63910900 0.24879900

N 0 -3.57649000 5.33161100 0.65490300

H 0 -3.09150700 6.11283700 1.07370500

C 0 -2.80479500 4.28277100 0.30129700

N 0 -3.23756600 3.37423900 -0.57349400

H 0 -2.77132800 2.45186700 -0.61390400

H 0 -4.03991800 3.53146800 -1.16236000

N 0 -1.60593200 4.12353200 0.86169200

H 0 -1.28930800 4.67607900 1.64627300

H 0 -0.89492800 3.49573400 0.43157900

C -1 -1.01162300 -1.29865900 7.49864000

H -1 -0.02171000 -0.90552200 7.31994100

H -1 -1.19296500 -1.49735900 8.54458100

H 0 -1.75522800 -0.57836400 7.14122500

C 0 -1.16297700 -2.66569700 6.80020300

H 0 -2.14024700 -3.09792100 7.05569800

H 0 -0.41257500 -3.35739000 7.20549300

C 0 -1.04423900 -2.65095000 5.30922100

N 0 -1.05268300 -3.82351900 4.56060800

C 0 -0.97886700 -1.64216200 4.38783000

H 0 -0.91121700 -0.58092700 4.53207800

C 0 -1.00598800 -3.53731800 3.25265300

H 0 -1.01053600 -4.23888700 2.43426400

N 0 -0.97557100 -2.21360200 3.12939300

H 0 -0.98500500 -1.71450700 2.20060400

C -1 3.76329500 6.18912400 2.28067600

H -1 4.63351500 6.67813200 1.86836200

H -1 3.29595200 6.71426800 3.10056200

H 0 3.00786300 6.12632100 1.48972000

C 0 4.14419200 4.74882900 2.71088400

H 0 5.09552900 4.73175400 3.25246600

H 0 3.35949400 4.34555900 3.36096700

C 0 4.19949100 3.85713100 1.47339300

O 0 3.10082000 3.70151400 0.87678200

O 0 5.30791000 3.36766100 1.08145700

C -1 12.09899000 -2.46466100 -0.63379100

H -1 13.14600800 -2.20485400 -0.68528800

H -1 11.87433900 -3.35811900 -1.19740500

H 0 11.88432000 -2.71571500 0.41465900

C 0 11.20196900 -1.28526500 -1.07254800

H 0 11.64356300 -0.79238900 -1.95100700

H 0 10.22188500 -1.66667300 -1.38147800

C 0 10.99108200 -0.25784600 0.05448100

H 0 10.28826000 -0.68234800 0.78443300

H 0 11.93725900 -0.10826400 0.59369300

C 0 10.51002500 1.13072000 -0.40169800

H 0 10.42292300 1.79439700 0.46960900

H 0 11.27894300 1.57328500 -1.04933300

C 0 9.20143200 1.19588500 -1.19157000

H 0 9.04730200 2.21297200 -1.56287800

H 0 9.21196500 0.52176400 -2.05175000

N 0 7.98873900 0.84817800 -0.38387500

H 0 7.13966800 1.23109000 -0.85323300

H 0 7.87407800 -0.19099200 -0.28613200

H 0 7.98002500 1.29380600 0.56384000

Cl 0 7.56309000 -2.23955100 -0.32010700

O 0 1.88766800 1.60194700 -3.47401700

H 0 3.35660900 2.47675600 -3.61814500

O 0 0.32878300 2.54153300 -0.11064400

O 0 0.82721300 3.69302700 2.58862900

H 0 0.40148700 2.84044700 2.38489300

H 0 1.52136000 3.75109800 1.89504700

O 0 -0.13926200 -0.11720300 -4.43568200

H 0 0.54796400 0.55526600 -4.18953600

H 0 0.12396800 -0.93272200 -3.96292400

O 0 -3.37332300 -1.01575100 -4.18139600

H 0 -4.09780400 -1.37298500 -3.63265500

H 0 -2.76425300 -1.78407200 -4.29173400

O 0 -4.82896500 -2.52033200 -2.15174400

H 0 -4.00326600 -2.20327900 -1.70103300

H 0 -5.20491100 -3.17748300 -1.53609200

O 0 2.37629600 3.92219800 -1.62175300

H 0 2.76858200 3.78899500 -0.70695700

H 0 2.77355900 -0.41614700 -1.78711200

O 0 -0.12376400 -1.63913100 -2.06239200

O 0 5.90081400 2.47048200 -1.36412200

H 0 5.47383100 2.72438000 -0.49242000

H 0 6.41430100 3.26264100 -1.59583200

O 0 4.12002800 3.09967700 -3.44930400

H 0 4.67790700 2.63751500 -2.78528700

H 0 3.11258100 3.78484200 -2.29046600

O 0 7.53465000 2.19528800 2.04027200

H 0 6.72632800 2.71932400 1.79428600

H 0 7.22401300 1.57209200 2.71502800

O 0 2.51468000 -1.08430900 -2.44758900

H 0 2.21973000 0.70757100 -3.23925500

H 0 1.56978200 -1.26941600 -2.21136100

H 0 -1.08374300 -4.76215500 4.93943200

**^2^L_S_4_^*^**

E (IEFPCM/B3LYP-D3/SDD,6-31G(d)) = -5854.276692 au

H (IEFPCM/B3LYP-D3/SDD,6-31G(d)) = -5852.723283 au

G (IEFPCM/B3LYP-D3/SDD,6-31G(d)) = -5852.962114 au

E (IEFPCM/B3LYP-D3/SDD,6-311+G(2d,p)//IEFPCM/B3LYP-D3/SDD,6-31G(d)) = -5856.101339 au

Mn 0 1.09352300 2.04077500 -1.93753900

Mn 0 -1.04967200 -2.53266800 -0.79848200

Mn 0 -0.23032700 0.84609900 0.18562800

Mn 0 -2.70319200 -0.47597400 0.41927000

O 0 0.18535500 0.43722300 -1.50037600

O 0 -2.00860400 1.01040900 -0.31785200

O 0 -2.60022700 -1.47307800 -1.08335400

O 0 -0.97294700 -0.95282500 0.79505700

Ca 0 -1.92931100 0.14033000 -2.73768200

C -1 0.50523800 4.27767500 -5.82831000

H -1 0.55760000 5.23906200 -5.33890600

H -1 0.69855700 4.30070800 -6.89037500

H 0 1.31495400 3.68016000 -5.39046700

C 0 -0.82425500 3.56218500 -5.51984600

H 0 -0.95664600 2.69911100 -6.18344700

H 0 -1.68703900 4.21538800 -5.71125900

C 0 -0.96174300 3.01152300 -4.08794300

O 0 -1.91550300 2.20787800 -3.86445100

O 0 -0.11300200 3.37713900 -3.21941500

C -1 -2.84044500 -6.98829700 -4.99670200

H -1 -1.87434800 -7.44953600 -5.13925600

H -1 -3.56727800 -7.17864000 -5.77251100

H 0 -3.24061900 -7.41906900 -4.06861400

C 0 -2.52248700 -5.50775200 -4.77850900

H 0 -3.27351300 -5.02570300 -4.13900300

H 0 -2.50205900 -4.95015700 -5.72123600

C 0 -1.12860000 -5.43713600 -4.12328600

H 0 -0.36287200 -5.61294700 -4.88761400

H 0 -1.03860100 -6.21002600 -3.35317500

C 0 -0.93355800 -4.08117600 -3.49503600

O 0 -0.77996300 -3.09760000 -4.26027500

O 0 -1.04493300 -4.06729700 -2.22136800

C -1 5.10004200 -4.52429600 1.24686800

H 0 5.86659800 -5.25494200 1.53099200

H 0 5.44543800 -4.02078100 0.34049900

C 0 4.92414500 -3.52450300 2.38598600

O 0 4.29413600 -3.83419900 3.41881400

C 0 3.77199200 -5.30039500 0.98610900

H 0 3.56813200 -5.91948400 1.86836800

H 0 3.91686500 -5.97804100 0.14002000

C 0 2.57727600 -4.42574500 0.71452800

N 0 2.27657600 -3.38393200 1.57649300

H 0 2.74873700 -3.23543200 2.46473500

C 0 1.60815500 -4.35519300 -0.26416900

H 0 1.46066800 -4.96928700 -1.13932400

C 0 1.22020600 -2.70966800 1.10456300

H 0 0.86928500 -1.78173300 1.50885700

N 0 0.76229500 -3.28341600 0.00113100

N 0 5.35532500 -2.27523900 2.14843700

H 0 5.95975800 -2.11969600 1.33516200

C -1 5.02787500 -1.15252900 3.01914100

H 0 5.89498300 -0.47984800 3.02823000

H 0 4.89193500 -1.53724900 4.03269200

C 0 3.77899000 -0.38154500 2.56624000

H 0 3.57781800 0.41961200 3.28845600

H 0 2.90963400 -1.04155200 2.59647600

C 0 3.94866400 0.19685300 1.16389900

H 0 4.44890600 -0.52818000 0.51007200

H 0 4.59271600 1.08431400 1.17755100

C 0 2.68584200 0.58771700 0.41223800

O 0 2.85417900 1.00754400 -0.75557600

O 0 1.55244500 0.41112100 0.99461000

C -1 -5.41608000 -3.93566100 2.40895600

H 0 -5.28441500 -3.13523800 3.14397500

H 0 -5.88951200 -4.78106800 2.92318000

C 0 -4.05858500 -4.38489600 1.84631400

H 0 -4.19608500 -5.17503400 1.10340500

H 0 -3.45243000 -4.81768200 2.65228100

C 0 -3.21237700 -3.29135000 1.18899600

O 0 -3.54734100 -2.08768400 1.38585200

O 0 -2.18814700 -3.67856200 0.53646900

C 0 -6.35109000 -3.47695100 1.29362600

O 0 -6.19376100 -3.81959900 0.11458300

N 0 -7.40532100 -2.72357500 1.68923200

H 0 -7.42458200 -2.37059400 2.63639500

C -1 -8.40466600 -2.21723300 0.75573700

H 0 -9.35014300 -2.08943400 1.28459900

H 0 -8.52756400 -2.95758700 -0.03889200

C 0 -8.00543600 -0.84695000 0.17386600

O 0 -8.52246700 0.19234900 0.58929900

N 0 -7.06382700 -0.89514600 -0.79839300

H 0 -6.60948000 -1.77516400 -1.02643000

C 0 -6.48243900 0.29372800 -1.38902700

H 0 -6.67285000 0.32682400 -2.46538400

H 0 -6.96025000 1.16099300 -0.92312500

C 0 -4.96449700 0.38048600 -1.18008400

O 0 -4.25615200 0.76527500 -2.12926000

O 0 -4.55984900 0.05198300 -0.00096700

C -1 -3.48936900 3.84282500 4.78885200

H -1 -3.51989100 3.60047800 5.84086700

H -1 -4.31219600 4.46244300 4.46419000

H 0 -2.58533400 4.45324800 4.64791300

C 0 -3.43970800 2.66260000 3.83552000

H 0 -3.48570800 3.05838800 2.81237900

H 0 -4.31302000 2.01109900 3.94595900

C 0 -2.15693900 1.83799100 3.99167000

H 0 -2.29224900 1.09520600 4.78486800

H 0 -1.30501300 2.47229400 4.25278800

C 0 -1.86864400 1.15896400 2.67666000

O 0 -0.76055800 1.41055300 2.11648300

O 0 -2.79579400 0.41549000 2.22062500

C -1 -5.61879100 9.07200900 1.52717400

H -1 -5.48058400 8.71480800 2.53698200

H -1 -6.65609400 9.28745100 1.31754600

H 0 -5.07182200 10.01805900 1.43285300

C 0 -5.05905900 8.02740000 0.55607900

H 0 -3.96686200 8.00283700 0.67635600

H 0 -5.24097100 8.32329800 -0.48539000

C 0 -5.65872500 6.63629000 0.80739900

H 0 -6.72907700 6.65095600 0.56848900

H 0 -5.57517400 6.38303700 1.87327800

C 0 -5.00347300 5.51983600 -0.00598000

H 0 -5.07578000 5.74139000 -1.08124300

H 0 -5.52180900 4.57296200 0.18018900

N 0 -3.60573300 5.36104500 0.39485300

H 0 -3.11687400 6.19230000 0.69694700

C 0 -2.82018100 4.32738100 0.02360800

N 0 -3.29651000 3.34863500 -0.74621800

H 0 -2.82274200 2.43032100 -0.73967600

H 0 -4.16911100 3.42995000 -1.24344400

N 0 -1.55526000 4.27744300 0.43941800

H 0 -1.21977900 4.78730200 1.24696400

H 0 -0.89507800 3.56204000 0.07052300

C -1 -0.99424800 -0.95697100 7.58443900

H -1 -0.00976600 -0.55844500 7.38850600

H -1 -1.17330400 -1.11206300 8.63812100

H 0 -1.74504700 -0.25842800 7.19930200

C 0 -1.12883400 -2.34525100 6.92919200

H 0 -2.10300700 -2.78045300 7.19060600

H 0 -0.37288500 -3.01886500 7.35453700

C 0 -1.00361000 -2.35570100 5.43915600

N 0 -1.08175600 -3.53097600 4.70082200

C 0 -0.86627800 -1.36049400 4.50930000

H 0 -0.72722400 -0.30385500 4.64528200

C 0 -1.00907300 -3.25399600 3.39040600

H 0 -1.04426700 -3.96559600 2.58108800

N 0 -0.89106900 -1.93799700 3.25471100

H 0 -0.91632500 -1.44282100 2.29435500

C -1 3.67937900 6.35974500 2.04216900

H -1 4.54296400 6.84213900 1.60865600

H -1 3.20447800 6.91386700 2.83829300

H 0 2.92429000 6.27316600 1.25204800

C 0 4.05203000 4.92951200 2.50838500

H 0 4.99935700 4.91967100 3.05706500

H 0 3.26062200 4.54850900 3.16409100

C 0 4.11377600 3.99233600 1.30195500

O 0 3.02644700 3.82262800 0.68873000

O 0 5.22283300 3.46938600 0.95511000

C -1 12.13482700 -2.29786000 -0.49105300

H -1 13.17818800 -2.02615500 -0.55406600

H -1 11.92275900 -3.21820000 -1.01488200

H 0 11.92363900 -2.50730800 0.56718400

C 0 11.22534600 -1.14689900 -0.97658700

H 0 11.66842300 -0.67501400 -1.86576100

H 0 10.25393700 -1.55167600 -1.28212600

C 0 10.99220100 -0.08942800 0.11768600

H 0 10.29490500 -0.50629700 0.85704500

H 0 11.93408800 0.09397800 0.65387200

C 0 10.48357000 1.27462000 -0.37946500

H 0 10.39230200 1.96509000 0.47041600

H 0 11.23684400 1.70983500 -1.04999800

C 0 9.16468700 1.28515800 -1.15348100

H 0 8.97759600 2.28662100 -1.55134300

H 0 9.18155800 0.58661000 -1.99390100

N 0 7.97430200 0.92768600 -0.31847100

H 0 7.10695000 1.24776700 -0.80286100

H 0 7.90635100 -0.10878100 -0.16580200

H 0 7.94962100 1.42173500 0.60404000

Cl 0 7.67241400 -2.16502200 -0.09427900

O 0 1.82201300 1.27645800 -3.53421900

H 0 3.34116400 2.09061200 -3.73031400

O 0 0.30610700 2.47974000 -0.27247700

O 0 0.71338400 3.92758200 2.34532600

H 0 0.29188600 3.05623100 2.23398700

H 0 1.42111800 3.91330400 1.66257100

O 0 -0.30729800 -0.20413700 -4.60390400

H 0 0.45110400 0.37027800 -4.32183400

H 0 -0.07443900 -1.11021100 -4.31737200

O 0 -3.06768000 -1.55732900 -4.09246500

H 0 -3.70619200 -2.06605400 -3.54987700

H 0 -2.30345000 -2.16435700 -4.24208600

O 0 -4.79617300 -2.59010400 -2.04753500

H 0 -4.01906700 -2.19014900 -1.56988400

H 0 -5.08501500 -3.31813700 -1.46799500

O 0 2.37494700 3.68262000 -1.84834800

H 0 2.74271600 3.65745300 -0.91596500

H 0 2.69001100 -0.65631600 -1.75054800

O 0 -0.20478000 -1.65942100 -2.21347400

O 0 5.82862200 2.37427600 -1.40397600

H 0 5.40288200 2.70992900 -0.55958100

H 0 6.30287800 3.15449700 -1.73730900

O 0 4.12267400 2.69684000 -3.60485500

H 0 4.64960100 2.28735400 -2.88479000

H 0 3.12115900 3.48520100 -2.48509000

O 0 7.44233800 2.38381600 2.02913900

H 0 6.63505200 2.88316800 1.73336100

H 0 7.11945800 1.79322000 2.72710800

O 0 2.48329600 -1.27361800 -2.47664500

H 0 2.14137900 0.37828100 -3.27652200

H 0 1.52240200 -1.46998400 -2.33284800

H 0 -1.17017500 -4.46264200 5.08747100

**^2'^L_S_4_^*^**

E (IEFPCM/B3LYP-D3/SDD,6-31G(d)) = -5854.292563 au

H (IEFPCM/B3LYP-D3/SDD,6-31G(d)) = -5852.739237 au

G (IEFPCM/B3LYP-D3/SDD,6-31G(d)) = -5852.975724 au

E (IEFPCM/B3LYP-D3/SDD,6-311+G(2d,p)//IEFPCM/B3LYP-D3/SDD,6-31G(d)) = -5856.116592 au

Mn 0 1.09219800 2.07427300 -1.91320400

Mn 0 -1.06885300 -2.55006400 -0.87954500

Mn 0 -0.22249000 0.82624100 0.17782900

Mn 0 -2.70373100 -0.48234200 0.40115800

O 0 0.18736800 0.46001600 -1.52784800

O 0 -1.99643700 1.01381800 -0.31863200

O 0 -2.65495800 -1.44086400 -1.10898600

O 0 -0.96734900 -0.97170400 0.74781300

Ca 0 -1.95317000 0.20969000 -2.71560300

C -1 0.51748100 4.31932100 -5.80746200

H -1 0.56838000 5.27710600 -5.31089700

H -1 0.71192300 4.35040600 -6.86911800

H 0 1.32580600 3.71811300 -5.37230300

C 0 -0.81294500 3.60120500 -5.51154300

H 0 -0.93348600 2.73535000 -6.17390500

H 0 -1.67478600 4.25102300 -5.71826300

C 0 -0.97219900 3.05545600 -4.07978500

O 0 -1.93213900 2.25136600 -3.87469700

O 0 -0.14166700 3.41933200 -3.19559100

C -1 -2.81813600 -6.95572600 -5.06294600

H -1 -1.85143700 -7.41496300 -5.20786200

H -1 -3.54394600 -7.14101700 -5.84093300

H 0 -3.22004600 -7.40351500 -4.14331300

C 0 -2.50527900 -5.48263200 -4.80820000

H 0 -3.28678400 -5.00547100 -4.20312400

H 0 -2.42829400 -4.91128700 -5.73987100

C 0 -1.14844700 -5.44397600 -4.07361200

H 0 -0.33796100 -5.56836400 -4.79994500

H 0 -1.09589900 -6.25818200 -3.34424800

C 0 -1.00340100 -4.13490400 -3.35103800

O 0 -0.77823000 -3.10429200 -4.03098200

O 0 -1.22425400 -4.18944400 -2.08953900

C -1 5.11320900 -4.53037700 1.20732100

H 0 5.89353000 -5.25158200 1.47876500

H 0 5.44360700 -4.01586300 0.30151300

C 0 4.93664000 -3.54037900 2.35562500

O 0 4.30904700 -3.85949700 3.38673900

C 0 3.79428400 -5.31979700 0.95423500

H 0 3.59743100 -5.93718700 1.83926500

H 0 3.94200800 -5.99882400 0.10964400

C 0 2.59836800 -4.44998700 0.68117700

N 0 2.28237100 -3.41752500 1.54873600

H 0 2.74471500 -3.27318000 2.44242400

C 0 1.64785800 -4.36748600 -0.31381800

H 0 1.52024500 -4.97824500 -1.19496300

C 0 1.23356800 -2.73886000 1.06122400

H 0 0.87297700 -1.81626900 1.46871300

N 0 0.79635500 -3.29943300 -0.05550900

N 0 5.37004100 -2.28956100 2.12966000

H 0 5.97140700 -2.12715400 1.31578800

C -1 5.03585000 -1.17190200 3.00443800

H 0 5.90071100 -0.49649200 3.02083900

H 0 4.89579700 -1.56171700 4.01545200

C 0 3.78607000 -0.40483800 2.54680300

H 0 3.57731600 0.39396500 3.26953300

H 0 2.92006900 -1.06959700 2.57018000

C 0 3.95894500 0.17773200 1.14636600

H 0 4.45865900 -0.54648700 0.49100900

H 0 4.60410300 1.06429900 1.16337700

C 0 2.69654200 0.57180600 0.39532600

O 0 2.86352700 1.01619200 -0.76398900

O 0 1.56268400 0.37264800 0.96831000

C -1 -5.40472900 -3.96051200 2.36231400

H 0 -5.28257500 -3.16482600 3.10415800

H 0 -5.87489900 -4.81270700 2.86862800

C 0 -4.04284400 -4.39482400 1.80459700

H 0 -4.17036500 -5.17985100 1.05454800

H 0 -3.43730300 -4.82906300 2.61021300

C 0 -3.20415200 -3.29027800 1.15817000

O 0 -3.55370000 -2.08948900 1.35535200

O 0 -2.17576500 -3.66557900 0.51099000

C 0 -6.34097600 -3.50089000 1.24812500

O 0 -6.18590200 -3.84375400 0.06932600

N 0 -7.39540300 -2.74877500 1.64799300

H 0 -7.41026900 -2.39618100 2.59545100

C -1 -8.39320000 -2.23277000 0.71862100

H 0 -9.34763200 -2.13776000 1.23862700

H 0 -8.49212700 -2.95146200 -0.09861500

C 0 -8.01009500 -0.83855500 0.18586100

O 0 -8.53595500 0.17931900 0.64206500

N 0 -7.07010900 -0.84067100 -0.78850100

H 0 -6.60379600 -1.70458900 -1.05509700

C 0 -6.49895200 0.37604200 -1.33172300

H 0 -6.70660100 0.45963600 -2.40229500

H 0 -6.96800200 1.21992500 -0.81678900

C 0 -4.97729600 0.44599700 -1.14176400

O 0 -4.27766700 0.83349200 -2.09751600

O 0 -4.56111400 0.09839900 0.02680900

C -1 -3.48815500 3.80198800 4.80184200

H -1 -3.51957700 3.55182700 5.85200100

H -1 -4.31123400 4.42320000 4.48088500

H 0 -2.58704500 4.41925700 4.67039400

C 0 -3.42369300 2.63086500 3.83844800

H 0 -3.47331900 3.03421300 2.81844100

H 0 -4.28774600 1.96623800 3.94252200

C 0 -2.12996000 1.82358600 3.98962000

H 0 -2.24857900 1.08841800 4.79289000

H 0 -1.28380600 2.47085300 4.23726400

C 0 -1.84412000 1.13268000 2.67948600

O 0 -0.73168100 1.37261800 2.12090200

O 0 -2.77187600 0.39116600 2.22624900

C -1 -5.61914200 9.05313100 1.57666900

H -1 -5.48167800 8.68859500 2.58395400

H -1 -6.65642700 9.26911900 1.36751600

H 0 -5.07367400 10.00087500 1.49041900

C 0 -5.05545800 8.01647100 0.60023100

H 0 -3.96327900 7.99467700 0.72103000

H 0 -5.23732500 8.31680900 -0.43994300

C 0 -5.65080600 6.62255500 0.84460700

H 0 -6.71884800 6.63183900 0.59542400

H 0 -5.57632000 6.36847900 1.91096900

C 0 -4.98091300 5.51134200 0.03716800

H 0 -5.03965800 5.73592100 -1.03834300

H 0 -5.49846600 4.56213800 0.21351900

N 0 -3.58807600 5.35569600 0.45651200

H 0 -3.10614300 6.18605900 0.77183400

C 0 -2.79638100 4.32744100 0.08255300

N 0 -3.26405400 3.35898900 -0.70480800

H 0 -2.79563800 2.43709000 -0.69362800

H 0 -4.13592900 3.44179800 -1.20283100

N 0 -1.53635200 4.26922400 0.51158100

H 0 -1.20833300 4.77502500 1.32431000

H 0 -0.87226700 3.55605000 0.13858300

C -1 -0.99138300 -1.01597300 7.56451600

H -1 -0.00707900 -0.61505800 7.37260800

H -1 -1.17142400 -1.17903900 8.61682400

H 0 -1.74144000 -0.31339200 7.18505400

C 0 -1.12467900 -2.39677600 6.89259000

H 0 -2.09987600 -2.83434100 7.14621500

H 0 -0.37050400 -3.07546400 7.31303800

C 0 -0.99484300 -2.39403400 5.40186200

N 0 -1.06657200 -3.56517600 4.65562800

C 0 -0.85920600 -1.39291100 4.47802500

H 0 -0.72453700 -0.33681600 4.62097400

C 0 -0.99173500 -3.28033000 3.34707800

H 0 -1.02362800 -3.98636500 2.53277300

N 0 -0.87873300 -1.96303500 3.21937500

H 0 -0.90339700 -1.46516200 2.26252700

C -1 3.68110200 6.34609900 2.08166200

H -1 4.54468300 6.83252300 1.65267200

H -1 3.20480300 6.89385100 2.88135200

H 0 2.92667700 6.26980100 1.28995600

C 0 4.05259600 4.90907000 2.52516700

H 0 5.00018800 4.88770600 3.07308100

H 0 3.26072200 4.51671200 3.17358900

C 0 4.11228000 3.99455500 1.30089900

O 0 3.02846900 3.85901900 0.67324300

O 0 5.21478000 3.45671300 0.95646600

C -1 12.14769500 -2.28434400 -0.50642100

H -1 13.19086000 -2.01117200 -0.56628700

H -1 11.93708900 -3.20098300 -1.03728100

H 0 11.93564200 -2.50208000 0.54993200

C 0 11.23638700 -1.13123500 -0.98470800

H 0 11.68364700 -0.64699400 -1.86510500

H 0 10.26895700 -1.53657500 -1.30199200

C 0 10.98986000 -0.08750100 0.11988700

H 0 10.28797300 -0.51577300 0.84829000

H 0 11.92654800 0.09349600 0.66590700

C 0 10.47982600 1.28033600 -0.36554300

H 0 10.38126400 1.96123800 0.49121200

H 0 11.23592900 1.72512100 -1.02657200

C 0 9.16564300 1.29592200 -1.14764000

H 0 8.98029000 2.30040200 -1.53864800

H 0 9.18840400 0.60407300 -1.99346000

N 0 7.97016500 0.93096200 -0.32307400

H 0 7.10560600 1.25359100 -0.81046600

H 0 7.90323900 -0.10687400 -0.17868200

H 0 7.93951300 1.41769100 0.60335800

Cl 0 7.68066500 -2.16420000 -0.12653600

O 0 1.81413100 1.32438000 -3.53315200

H 0 3.32681100 2.13389400 -3.73030800

O 0 0.32159800 2.48332200 -0.22932600

O 0 0.75143100 3.87293900 2.38892000

H 0 0.32264500 3.00742300 2.25896600

H 0 1.43221300 3.88272900 1.68003200

O 0 -0.30181100 -0.15975600 -4.54670200

H 0 0.44576600 0.44351200 -4.28701200

H 0 -0.01791000 -1.05131400 -4.27179300

O 0 -3.07495500 -1.51349600 -4.08329700

H 0 -3.74062100 -1.99141400 -3.54379000

H 0 -2.31863400 -2.13698900 -4.17257600

O 0 -4.86103500 -2.51019600 -2.06375900

H 0 -4.07763400 -2.12288100 -1.58095400

H 0 -5.12642500 -3.26994000 -1.51395900

O 0 2.35472400 3.70194400 -1.84452700

H 0 2.73210600 3.68700900 -0.91393900

H 0 2.67543300 -0.62484600 -1.76932400

O 0 -0.28136700 -1.69530900 -2.16565400

O 0 5.82729200 2.38532700 -1.41297900

H 0 5.39858600 2.71220800 -0.56666600

H 0 6.30492800 3.16840500 -1.73469700

O 0 4.10701800 2.74288500 -3.60284800

H 0 4.63872300 2.32982100 -2.88807700

H 0 3.09897900 3.51405000 -2.48797600

O 0 7.42961700 2.36863200 2.03291100

H 0 6.62169200 2.86785700 1.73774800

H 0 7.10717600 1.77506200 2.72853600

O 0 2.45468500 -1.26169800 -2.47506400

H 0 2.12924600 0.42504200 -3.28382700

H 0 1.50425100 -1.45502500 -2.30311100

H 0 -1.15271000 -4.49952000 5.03620600

**^8^L_S_4_^*^**

E (IEFPCM/B3LYP-D3/SDD,6-31G(d)) = -5854.291126 au

H (IEFPCM/B3LYP-D3/SDD,6-31G(d)) = -5852.737857 au

G (IEFPCM/B3LYP-D3/SDD,6-31G(d)) = -5852.975842 au

E (IEFPCM/B3LYP-D3/SDD,6-311+G(2d,p)//IEFPCM/B3LYP-D3/SDD,6-31G(d)) = -5856.115222 au

Mn 0 1.08840600 2.06891100 -1.91018600

Mn 0 -1.05973500 -2.54873600 -0.87598700

Mn 0 -0.22711800 0.82661600 0.18369600

Mn 0 -2.70595500 -0.47958900 0.40203800

O 0 0.17959700 0.45468100 -1.52088200

O 0 -2.00337300 1.01724600 -0.31766300

O 0 -2.64396100 -1.43584700 -1.10682200

O 0 -0.96214100 -0.96989000 0.74960300

Ca 0 -1.95280800 0.20656300 -2.71570800

C -1 0.51513900 4.31777200 -5.80906300

H -1 0.56576600 5.27574400 -5.31282900

H -1 0.70943900 4.34856500 -6.87075300

H 0 1.32366300 3.71703200 -5.37364300

C 0 -0.81514700 3.59895700 -5.51366400

H 0 -0.93575700 2.73415700 -6.17738000

H 0 -1.67715000 4.24901500 -5.71904700

C 0 -0.97398100 3.05068400 -4.08284000

O 0 -1.93393600 2.24614800 -3.87902000

O 0 -0.14370700 3.41383200 -3.19824000

C -1 -2.81645700 -6.95818200 -5.06030400

H -1 -1.84961600 -7.41713200 -5.20518400

H -1 -3.54229900 -7.14399100 -5.83813800

H 0 -3.21823600 -7.40629900 -4.14074000

C 0 -2.50354400 -5.48547100 -4.80480400

H 0 -3.28584400 -5.00778200 -4.20118200

H 0 -2.42387900 -4.91401200 -5.73619000

C 0 -1.14834200 -5.44893100 -4.06738700

H 0 -0.33668000 -5.57656000 -4.79184700

H 0 -1.09949900 -6.26198900 -3.33645300

C 0 -1.00180000 -4.13903100 -3.34715700

O 0 -0.76971900 -3.11106600 -4.02900900

O 0 -1.22841600 -4.18921900 -2.08664500

C -1 5.11481800 -4.52794000 1.20815700

H 0 5.89377900 -5.25001800 1.48108100

H 0 5.44700900 -4.01424900 0.30255300

C 0 4.93754800 -3.53738900 2.35570300

O 0 4.31029600 -3.85642600 3.38708600

C 0 3.79525300 -5.31611100 0.95367700

H 0 3.59756800 -5.93420600 1.83800300

H 0 3.94261900 -5.99437500 0.10842500

C 0 2.59989700 -4.44525700 0.68138900

N 0 2.28459300 -3.41345400 1.55005400

H 0 2.74728700 -3.27013900 2.44378600

C 0 1.64921400 -4.36161800 -0.31325600

H 0 1.52083300 -4.97127900 -1.19499800

C 0 1.23618000 -2.73367000 1.06396500

H 0 0.87576300 -1.81134700 1.47226700

N 0 0.79846200 -3.29326100 -0.05311200

N 0 5.36931100 -2.28626000 2.12850900

H 0 5.97054400 -2.12397300 1.31450000

C -1 5.03651100 -1.16888200 3.00414300

H 0 5.90148600 -0.49356700 3.01931400

H 0 4.89839000 -1.55919800 4.01523200

C 0 3.78627400 -0.40129300 2.54948500

H 0 3.57884200 0.39692100 3.27322300

H 0 2.92008800 -1.06573900 2.57382600

C 0 3.95751000 0.18223100 1.14935800

H 0 4.45833100 -0.54051200 0.49327200

H 0 4.60103600 1.06996800 1.16657300

C 0 2.69395900 0.57427200 0.39961800

O 0 2.85948700 1.01281800 -0.76236800

O 0 1.56119200 0.37957200 0.97562700

C -1 -5.40317500 -3.96134700 2.36425800

H 0 -5.28197000 -3.16684900 3.10755200

H 0 -5.87349200 -4.81459400 2.86865600

C 0 -4.04054100 -4.39424000 1.80674000

H 0 -4.16725700 -5.17864300 1.05586500

H 0 -3.43525900 -4.82911800 2.61222400

C 0 -3.20129800 -3.28889200 1.16180700

O 0 -3.55317100 -2.08853700 1.35818800

O 0 -2.17075500 -3.66344000 0.51775500

C 0 -6.33842800 -3.50009800 1.24995400

O 0 -6.18235600 -3.84104800 0.07070000

N 0 -7.39376500 -2.74933600 1.64986900

H 0 -7.41027700 -2.39833900 2.59786800

C -1 -8.39245100 -2.23520500 0.72034900

H 0 -9.34603600 -2.13836100 1.24154800

H 0 -8.49303100 -2.95603900 -0.09482600

C 0 -8.00969100 -0.84278700 0.18294700

O 0 -8.53411200 0.17674500 0.63706400

N 0 -7.07239400 -0.84857900 -0.79405600

H 0 -6.60499300 -1.71339300 -1.05543400

C 0 -6.50014300 0.36597900 -1.34058400

H 0 -6.70390200 0.44459700 -2.41224800

H 0 -6.97175600 1.21193100 -0.83138000

C 0 -4.97939700 0.43875000 -1.14549500

O 0 -4.27775500 0.83182600 -2.09740500

O 0 -4.56623500 0.08717100 0.02312100

C -1 -3.48900400 3.80264800 4.80091200

H -1 -3.52020900 3.55283300 5.85116000

H -1 -4.31233900 4.42346400 4.47984600

H 0 -2.58758200 4.41912400 4.66831900

C 0 -3.42659500 2.63056500 3.83859600

H 0 -3.47425700 3.03322200 2.81821100

H 0 -4.29269000 1.96851600 3.94246700

C 0 -2.13521000 1.81944200 3.99178300

H 0 -2.25885300 1.08096900 4.79109500

H 0 -1.28830900 2.46360200 4.24490700

C 0 -1.84741100 1.13422700 2.67953600

O 0 -0.73816600 1.38222000 2.11864800

O 0 -2.77182300 0.38960700 2.22436500

C -1 -5.62222000 9.05195200 1.57422000

H -1 -5.48450400 8.68780600 2.58161100

H -1 -6.65960600 9.26750800 1.36512200

H 0 -5.07740500 10.00010500 1.48804300

C 0 -5.05822400 8.01623800 0.59730200

H 0 -3.96603500 7.99480600 0.71798000

H 0 -5.24036100 8.31719800 -0.44265000

C 0 -5.65314900 6.62215400 0.84093900

H 0 -6.72148700 6.63155400 0.59302800

H 0 -5.57728400 6.36691000 1.90692500

C 0 -4.98391900 5.51230600 0.03134100

H 0 -5.04508400 5.73771600 -1.04386100

H 0 -5.50014900 4.56245300 0.20802200

N 0 -3.59011700 5.35780400 0.44773600

H 0 -3.10810800 6.18819900 0.76282700

C 0 -2.79899400 4.32952100 0.07292200

N 0 -3.26866400 3.36147100 -0.71394900

H 0 -2.80203600 2.43924700 -0.70209100

H 0 -4.14110900 3.44470700 -1.21086600

N 0 -1.53849300 4.27092200 0.50005600

H 0 -1.20941400 4.77696600 1.31229800

H 0 -0.87482700 3.55613400 0.12847300

C -1 -0.99021200 -1.01350400 7.56491300

H -1 -0.00607100 -0.61231100 7.37274700

H -1 -1.17006600 -1.17627500 8.61729900

H 0 -1.74045000 -0.31108300 7.18548900

C 0 -1.12313500 -2.39411600 6.89269800

H 0 -2.09830700 -2.83190500 7.14605900

H 0 -0.36893600 -3.07280200 7.31311000

C 0 -0.99303800 -2.39122900 5.40190900

N 0 -1.06465100 -3.56257200 4.65608300

C 0 -0.85743500 -1.39046400 4.47757800

H 0 -0.72237100 -0.33439400 4.62049200

C 0 -0.98991500 -3.27800900 3.34742400

H 0 -1.02219000 -3.98446800 2.53352600

N 0 -0.87717700 -1.96077100 3.21888500

H 0 -0.90230100 -1.46288800 2.26009200

C -1 3.67902900 6.34833200 2.07898100

H -1 4.54238800 6.83491200 1.64971900

H -1 3.20263800 6.89619000 2.87854400

H 0 2.92452800 6.27144400 1.28744200

C 0 4.05106300 4.91160800 2.52274800

H 0 4.99873700 4.89054400 3.07052600

H 0 3.25943100 4.51919200 3.17144600

C 0 4.11072700 3.99673400 1.29872400

O 0 3.02723900 3.86207600 0.67040500

O 0 5.21294900 3.45759200 0.95535200

C -1 12.14830900 -2.28003900 -0.50721900

H -1 13.19137100 -2.00652400 -0.56730700

H -1 11.93795700 -3.19693200 -1.03774100

H 0 11.93655900 -2.49749100 0.54927700

C 0 11.23623300 -1.12772300 -0.98579100

H 0 11.68227900 -0.64449900 -1.86735900

H 0 10.26855800 -1.53377400 -1.30140200

C 0 10.99068600 -0.08252500 0.11763600

H 0 10.28994400 -0.50989800 0.84765700

H 0 11.92799300 0.09961700 0.66221600

C 0 10.47956300 1.28445600 -0.36911800

H 0 10.38131900 1.96635700 0.48688000

H 0 11.23500800 1.72877100 -1.03121500

C 0 9.16482500 1.29867700 -1.15033600

H 0 8.97873300 2.30277000 -1.54199300

H 0 9.18734200 0.60620700 -1.99565500

N 0 7.97005100 0.93384000 -0.32471300

H 0 7.10492100 1.25551100 -0.81183100

H 0 7.90341000 -0.10391200 -0.17971400

H 0 7.93968000 1.42137300 0.60130100

Cl 0 7.68121000 -2.16145100 -0.12615000

O 0 1.81035700 1.31750500 -3.53001100

H 0 3.32253100 2.12675700 -3.72956000

O 0 0.32057100 2.48317700 -0.22927500

O 0 0.74734900 3.88266700 2.38304100

H 0 0.31811100 3.01733400 2.25388800

H 0 1.42967800 3.89001000 1.67559200

O 0 -0.30037500 -0.16608100 -4.54481800

H 0 0.44681900 0.43771500 -4.28435400

H 0 -0.01617100 -1.05726800 -4.26900500

O 0 -3.06838800 -1.52225500 -4.08176300

H 0 -3.73178400 -2.00167500 -3.54052900

H 0 -2.31000700 -2.14310500 -4.17128300

O 0 -4.84765500 -2.51252000 -2.06151600

H 0 -4.06551200 -2.12085000 -1.57967100

H 0 -5.11138900 -3.27103500 -1.50935400

O 0 2.35102900 3.69756500 -1.84647600

H 0 2.72919100 3.68516900 -0.91625700

H 0 2.67887700 -0.62652700 -1.76485800

O 0 -0.26907100 -1.70502200 -2.16684700

O 0 5.82430900 2.38462700 -1.41370000

H 0 5.39643300 2.71235200 -0.56734300

H 0 6.30017200 3.16779000 -1.73779500

O 0 4.10309700 2.73560300 -3.60334400

H 0 4.63437800 2.32390800 -2.88751700

H 0 3.09486200 3.50871800 -2.49005700

O 0 7.42948200 2.37223800 2.03091500

H 0 6.62064500 2.87035400 1.73633900

H 0 7.10839300 1.77803800 2.72662100

O 0 2.46248200 -1.26321000 -2.47225700

H 0 2.12862500 0.41940200 -3.27985400

H 0 1.51286700 -1.46265900 -2.30229100

H 0 -1.15101500 -4.49682700 5.03682100

**^8'^L_S_4_^*^**

E (IEFPCM/B3LYP-D3/SDD,6-31G(d)) = -5854.295844 au

H (IEFPCM/B3LYP-D3/SDD,6-31G(d)) = -5852.742353 au

G (IEFPCM/B3LYP-D3/SDD,6-31G(d)) = -5852.981161 au

E (IEFPCM/B3LYP-D3/SDD,6-311+G(2d,p)//IEFPCM/B3LYP-D3/SDD,6-31G(d)) = -5856.119827 au

Mn 0 1.09544300 2.06802000 -1.92222100

Mn 0 -1.07309800 -2.55397300 -0.87463200

Mn 0 -0.22230000 0.83010000 0.16919000

Mn 0 -2.70451900 -0.47852200 0.40069300

O 0 0.19072300 0.46564000 -1.52534000

O 0 -1.99900500 1.01389500 -0.32659800

O 0 -2.65780000 -1.44431100 -1.10582100

O 0 -0.96912200 -0.96686000 0.74906800

Ca 0 -1.95648100 0.19835100 -2.71518900

C -1 0.51880600 4.30796800 -5.81589200

H -1 0.57086600 5.26662200 -5.32112500

H -1 0.71305200 4.33685700 -6.87764400

H 0 1.32768000 3.70745900 -5.38066800

C 0 -0.81113400 3.59136400 -5.51500500

H 0 -0.93626300 2.72624800 -6.17744600

H 0 -1.67339500 4.24234400 -5.71603900

C 0 -0.96314300 3.04308100 -4.08360700

O 0 -1.92293200 2.24136300 -3.87371900

O 0 -0.12494300 3.40425300 -3.20425500

C -1 -2.82906500 -6.96198700 -5.04960700

H -1 -1.86290400 -7.42255900 -5.19387100

H -1 -3.55524700 -7.14793200 -5.82709000

H 0 -3.23126000 -7.40789000 -4.12916700

C 0 -2.51434600 -5.48891900 -4.79702200

H 0 -3.29449200 -5.01033600 -4.19132500

H 0 -2.43789600 -4.91859600 -5.72935500

C 0 -1.15651800 -5.45144700 -4.06429600

H 0 -0.34713500 -5.57716500 -4.79159700

H 0 -1.10399400 -6.26510900 -3.33435200

C 0 -1.00898000 -4.14209600 -3.34277000

O 0 -0.78261600 -3.11270600 -4.02416000

O 0 -1.22915500 -4.19533900 -2.08119400

C -1 5.10629900 -4.53367300 1.21442600

H 0 5.88611100 -5.25511700 1.48664100

H 0 5.43616900 -4.02145900 0.30710200

C 0 4.93170800 -3.54109700 2.36074100

O 0 4.30578500 -3.85807400 3.39348400

C 0 3.78604500 -5.32207500 0.96511300

H 0 3.58963900 -5.93660200 1.85221300

H 0 3.93157200 -6.00369200 0.12222400

C 0 2.59108000 -4.45130400 0.69101800

N 0 2.27864000 -3.41402100 1.55412800

H 0 2.74171900 -3.26686000 2.44685700

C 0 1.63885600 -4.37187400 -0.30255100

H 0 1.50845600 -4.98657100 -1.18054100

C 0 1.23018600 -2.73563700 1.06530500

H 0 0.87172400 -1.81042200 1.46896800

N 0 0.78993600 -3.30097100 -0.04779800

N 0 5.36450800 -2.29066600 2.13123500

H 0 5.96444800 -2.12975000 1.31601300

C -1 5.03302500 -1.17176400 3.00528300

H 0 5.89974700 -0.49869700 3.02222200

H 0 4.89124500 -1.56098600 4.01626000

C 0 3.78539600 -0.40109900 2.54715500

H 0 3.57744000 0.39683100 3.27104700

H 0 2.91803400 -1.06410200 2.56896100

C 0 3.95959900 0.18358200 1.14744000

H 0 4.46544500 -0.53684300 0.49274300

H 0 4.59972400 1.07384100 1.16697200

C 0 2.69681600 0.57162100 0.39319600

O 0 2.86502500 1.00375000 -0.77118300

O 0 1.56355200 0.38050300 0.96888400

C -1 -5.41075500 -3.95006600 2.37060000

H 0 -5.28745500 -3.15305100 3.11085100

H 0 -5.88222000 -4.80050500 2.87868200

C 0 -4.04949900 -4.38741500 1.81389400

H 0 -4.17799200 -5.17363700 1.06524300

H 0 -3.44477800 -4.82108400 2.62046100

C 0 -3.20880400 -3.28522600 1.16593100

O 0 -3.55562000 -2.08357200 1.36164100

O 0 -2.18122600 -3.66391800 0.51915500

C 0 -6.34658200 -3.49129300 1.25573900

O 0 -6.19243300 -3.83669000 0.07755500

N 0 -7.39989400 -2.73694100 1.65440700

H 0 -7.41398000 -2.38227000 2.60109400

C -1 -8.39767600 -2.22210500 0.72432300

H 0 -9.35138600 -2.12379500 1.24502900

H 0 -8.49871600 -2.94324600 -0.09050400

C 0 -8.01282100 -0.83029200 0.18663300

O 0 -8.53653600 0.18995400 0.64002700

N 0 -7.07402100 -0.83726500 -0.78885400

H 0 -6.60886200 -1.70282400 -1.05202600

C 0 -6.50083400 0.37658900 -1.33628000

H 0 -6.70754600 0.45616300 -2.40733100

H 0 -6.96929600 1.22306800 -0.82509600

C 0 -4.97920100 0.44542700 -1.14535500

O 0 -4.27888300 0.83037900 -2.10188800

O 0 -4.56389600 0.09964900 0.02379700

C -1 -3.48510800 3.81486000 4.79521800

H -1 -3.51657800 3.56669500 5.84584800

H -1 -4.30757100 4.43637800 4.47327600

H 0 -2.58343800 4.43100500 4.66235000

C 0 -3.42246300 2.64212600 3.83360200

H 0 -3.47546300 3.04373700 2.81307000

H 0 -4.28594300 1.97729100 3.94125800

C 0 -2.12761600 1.83617900 3.98196800

H 0 -2.24344800 1.10174800 4.78627800

H 0 -1.28144700 2.48448100 4.22686200

C 0 -1.84415400 1.14402900 2.67180500

O 0 -0.73375200 1.38445100 2.10992800

O 0 -2.77255400 0.40117600 2.22187800

C -1 -5.61100700 9.06231600 1.56069900

H -1 -5.47372700 8.69950900 2.56863300

H -1 -6.64810000 9.27905500 1.35136500

H 0 -5.06402100 10.00889700 1.47178900

C 0 -5.04949300 8.02165800 0.58702100

H 0 -3.95715100 7.99937700 0.70625800

H 0 -5.23270700 8.31821800 -0.45399400

C 0 -5.64564000 6.62910700 0.83770900

H 0 -6.71414800 6.63842900 0.59057900

H 0 -5.56940400 6.37900400 1.90489200

C 0 -4.97862300 5.51394600 0.03327200

H 0 -5.03899400 5.73460500 -1.04294600

H 0 -5.49711600 4.56609400 0.21419800

N 0 -3.58504200 5.35831600 0.45039500

H 0 -3.10278800 6.18900200 0.76448000

C 0 -2.79333300 4.33129000 0.07335900

N 0 -3.26162300 3.36191300 -0.71245500

H 0 -2.79428600 2.43899800 -0.69935100

H 0 -4.13871400 3.44124800 -1.20191300

N 0 -1.53088600 4.27689000 0.49654800

H 0 -1.20091000 4.77847400 1.31122900

H 0 -0.87018600 3.56273300 0.12371200

C -1 -0.99304700 -1.00068100 7.56635000

H -1 -0.00834400 -0.60120900 7.37348300

H -1 -1.17304100 -1.16158300 8.61900000

H 0 -1.74216900 -0.29752800 7.18603700

C 0 -1.12829900 -2.38190200 6.89577800

H 0 -2.10415600 -2.81781500 7.14972300

H 0 -0.37514500 -3.06131100 7.31689000

C 0 -0.99819100 -2.38098300 5.40493400

N 0 -1.07058500 -3.55323300 4.66047500

C 0 -0.86143000 -1.38147400 4.47944200

H 0 -0.72610500 -0.32523800 4.62074900

C 0 -0.99508200 -3.27043500 3.35148200

H 0 -1.02769500 -3.97780300 2.53835400

N 0 -0.88092300 -1.95347900 3.22158800

H 0 -0.90526800 -1.45699000 2.26326700

C -1 3.68635800 6.34598800 2.06875900

H -1 4.55038300 6.83065900 1.63867600

H -1 3.21083500 6.89575600 2.86752600

H 0 2.93199900 6.26362500 1.27757200

C 0 4.06040900 4.91317600 2.52563200

H 0 5.00666100 4.90079900 3.07613100

H 0 3.26807600 4.52530200 3.17608800

C 0 4.12629800 3.98735900 1.31078200

O 0 3.04034000 3.82416300 0.69357700

O 0 5.23639900 3.46840000 0.96127100

C -1 12.14288400 -2.29859400 -0.50501200

H -1 13.18633600 -2.02668400 -0.56561100

H -1 11.93115400 -3.21599000 -1.03411400

H 0 11.93064000 -2.51372000 0.55184600

C 0 11.23323200 -1.14540900 -0.98569800

H 0 11.67832600 -0.66740000 -1.87058900

H 0 10.26319500 -1.54945400 -1.29663600

C 0 10.99502800 -0.09498100 0.11429200

H 0 10.29477200 -0.51671900 0.84804800

H 0 11.93458300 0.08554600 0.65551900

C 0 10.48789400 1.27199400 -0.37639100

H 0 10.39361700 1.95727200 0.47734400

H 0 11.24359400 1.71128800 -1.04153800

C 0 9.17177800 1.28764000 -1.15516400

H 0 8.98732700 2.29129700 -1.54870600

H 0 9.19106600 0.59326400 -1.99900200

N 0 7.97776800 0.92747900 -0.32643900

H 0 7.11282300 1.25320000 -0.81121100

H 0 7.90709100 -0.10992000 -0.18108700

H 0 7.95184200 1.41515200 0.59959400

Cl 0 7.67506200 -2.16690100 -0.12516700

O 0 1.82140400 1.31506200 -3.53998300

H 0 3.33562400 2.12965100 -3.72535800

O 0 0.32020600 2.47845700 -0.24920300

O 0 0.74748100 3.88036500 2.38068200

H 0 0.31945000 3.01372500 2.25476400

H 0 1.44203100 3.87995400 1.68485000

O 0 -0.30064200 -0.17285900 -4.54436900

H 0 0.44787000 0.43002300 -4.28727900

H 0 -0.01779600 -1.06382700 -4.26655900

O 0 -3.08039400 -1.52364700 -4.08195600

H 0 -3.74542500 -2.00003500 -3.54009800

H 0 -2.32454100 -2.14752700 -4.17134300

O 0 -4.86411100 -2.51237600 -2.05963100

H 0 -4.08108200 -2.12431800 -1.57661600

H 0 -5.13070300 -3.27052300 -1.50823900

O 0 2.36745900 3.70480900 -1.83289900

H 0 2.74324300 3.67565400 -0.90327500

H 0 2.66923600 -0.63526400 -1.76936900

O 0 -0.28464400 -1.70315300 -2.16092000

O 0 5.83790100 2.38996300 -1.40718500

H 0 5.41692600 2.72211700 -0.55882500

H 0 6.31457300 3.16994000 -1.73761400

O 0 4.11613600 2.73714800 -3.59494700

H 0 4.64405100 2.32359600 -2.87780600

H 0 3.11080800 3.51559000 -2.47611900

O 0 7.44860900 2.36606600 2.03184100

H 0 6.64321700 2.87029600 1.73902400

H 0 7.12270700 1.76978000 2.72355400

O 0 2.44968900 -1.27224300 -2.47557700

H 0 2.13757700 0.41649500 -3.28872400

H 0 1.49909300 -1.46637500 -2.30488500

H 0 -1.15775100 -4.48695100 5.04234900

**^6^L_S_4_^*^**

E (IEFPCM/B3LYP-D3/SDD,6-31G(d)) = -5854.300952 au

H (IEFPCM/B3LYP-D3/SDD,6-31G(d)) = -5852.745964 au

G (IEFPCM/B3LYP-D3/SDD,6-31G(d)) = -5852.983761 au

E (IEFPCM/B3LYP-D3/SDD,6-311+G(2d,p)//IEFPCM/B3LYP-D3/SDD,6-31G(d)) = -5856.125473 au

Mn 0 1.09424100 2.05010300 -1.91042000

Mn 0 -1.06743800 -2.59107300 -0.91349900

Mn 0 -0.23173400 0.82796700 0.18059800

Mn 0 -2.70168800 -0.48172500 0.40968100

O 0 0.17174000 0.44557800 -1.50853200

O 0 -2.00982400 1.01238800 -0.32280000

O 0 -2.63558500 -1.44358200 -1.10288700

O 0 -0.96818700 -0.95448800 0.77907600

Ca 0 -1.98109200 0.23816500 -2.72254500

C -1 0.51881800 4.28779800 -5.82123500

H -1 0.57069700 5.24818200 -5.32965300

H -1 0.71327600 4.31326000 -6.88301800

H 0 1.32398800 3.68560200 -5.38185200

C 0 -0.81489400 3.57965600 -5.52188400

H 0 -0.93908700 2.70802900 -6.17609300

H 0 -1.67373600 4.23095900 -5.73539200

C 0 -0.97742800 3.04670500 -4.08672000

O 0 -1.96111700 2.27450500 -3.86993900

O 0 -0.12697000 3.38612700 -3.21219200

C -1 -2.82800400 -6.97971600 -5.01770400

H -1 -1.86184100 -7.44077400 -5.16024800

H -1 -3.55411100 -7.16842800 -5.79463900

H 0 -3.22817900 -7.40923700 -4.08933000

C 0 -2.50900900 -5.50062800 -4.80000000

H 0 -3.25711700 -5.02065400 -4.15660400

H 0 -2.49227200 -4.94159100 -5.74202300

C 0 -1.11042100 -5.43864500 -4.14758700

H 0 -0.33988400 -5.52972900 -4.92013900

H 0 -0.99648800 -6.26038800 -3.43396100

C 0 -0.97174800 -4.13851800 -3.41137800

O 0 -0.75095500 -3.09585400 -4.07254300

O 0 -1.20707000 -4.21492000 -2.15142400

C -1 5.10602200 -4.52966300 1.23947000

H 0 5.89938800 -5.23699000 1.51029700

H 0 5.42206600 -4.01879800 0.32632500

C 0 4.92784400 -3.53146900 2.38074900

O 0 4.29891500 -3.84330700 3.41327800

C 0 3.79696900 -5.33784900 1.00531300

H 0 3.60752300 -5.93993200 1.90231700

H 0 3.94863200 -6.03066700 0.17273100

C 0 2.59719800 -4.48009500 0.71763000

N 0 2.27224300 -3.43660100 1.56879700

H 0 2.72899800 -3.27874300 2.46306300

C 0 1.65798800 -4.41158400 -0.28823100

H 0 1.54093800 -5.03283600 -1.16344500

C 0 1.22843100 -2.76454000 1.06133500

H 0 0.85848800 -1.83780300 1.45133800

N 0 0.80467300 -3.34069400 -0.05268600

N 0 5.36509300 -2.28322500 2.14888000

H 0 5.96423200 -2.12401900 1.33264000

C -1 5.03212200 -1.16182000 3.01905400

H 0 5.89480400 -0.48354700 3.02765100

H 0 4.89809700 -1.54642100 4.03290000

C 0 3.77742900 -0.40242100 2.56293800

H 0 3.56111200 0.39258200 3.28749500

H 0 2.91649100 -1.07428800 2.58279300

C 0 3.94865000 0.18431300 1.16409800

H 0 4.46584700 -0.52902500 0.51069800

H 0 4.57799100 1.08273300 1.18488300

C 0 2.68406200 0.55715000 0.40694100

O 0 2.84976800 0.96981400 -0.76508400

O 0 1.55201400 0.37837900 0.98770800

C -1 -5.41128400 -3.94327700 2.39194900

H 0 -5.27979700 -3.14123500 3.12536800

H 0 -5.88870800 -4.78563600 2.90779500

C 0 -4.05571200 -4.39449500 1.83405900

H 0 -4.19125200 -5.19081600 1.09759400

H 0 -3.44829500 -4.81851800 2.64376100

C 0 -3.21288500 -3.30477000 1.16704500

O 0 -3.54075400 -2.09793200 1.36776500

O 0 -2.20182600 -3.70222600 0.50599000

C 0 -6.34460400 -3.48377400 1.27518400

O 0 -6.18588500 -3.82495900 0.09626100

N 0 -7.40034300 -2.73189800 1.67175400

H 0 -7.41977400 -2.38079500 2.61964300

C -1 -8.39807400 -2.22108300 0.73938300

H 0 -9.35105200 -2.11913200 1.26076900

H 0 -8.50124900 -2.94701800 -0.07098900

C 0 -8.01342300 -0.83409900 0.19229900

O 0 -8.53389300 0.19029600 0.63980300

N 0 -7.07909100 -0.85040100 -0.78728400

H 0 -6.61310100 -1.71865700 -1.03879400

C 0 -6.50374400 0.35846000 -1.34141100

H 0 -6.70461500 0.42963200 -2.41412100

H 0 -6.97541800 1.20917800 -0.84019600

C 0 -4.98404200 0.43111500 -1.14298200

O 0 -4.28473300 0.83233600 -2.09446700

O 0 -4.56975600 0.07389600 0.02187000

C -1 -3.48682700 3.82994600 4.79086700

H -1 -3.51841600 3.58532100 5.84228000

H -1 -4.30925100 4.45032100 4.46664000

H 0 -2.58347600 4.44241200 4.65360800

C 0 -3.43152600 2.65228000 3.83483200

H 0 -3.47936600 3.05027700 2.81260600

H 0 -4.30138600 1.99595000 3.94347700

C 0 -2.14479400 1.83353700 3.98833300

H 0 -2.27318400 1.09257100 4.78447600

H 0 -1.29463500 2.47243800 4.24407500

C 0 -1.86060700 1.15079300 2.67403600

O 0 -0.75339200 1.40144600 2.10978300

O 0 -2.78760800 0.40751100 2.22133300

C -1 -5.61268600 9.06630100 1.53838500

H -1 -5.47552400 8.70692600 2.54754500

H -1 -6.64975100 9.28226600 1.32812800

H 0 -5.06690600 10.01350300 1.44779200

C 0 -5.05006700 8.02543400 0.56663500

H 0 -3.95755900 8.00637400 0.68466900

H 0 -5.23552700 8.31923500 -0.47477500

C 0 -5.64259400 6.63262200 0.82198300

H 0 -6.71206600 6.63937500 0.57884600

H 0 -5.56149800 6.38463600 1.88927000

C 0 -4.97592600 5.51835400 0.01688900

H 0 -5.04602900 5.73392100 -1.05979000

H 0 -5.48732800 4.56834100 0.20583800

N 0 -3.57848500 5.37173400 0.42364200

H 0 -3.09340500 6.20735700 0.71954000

C 0 -2.79178400 4.33633900 0.06050100

N 0 -3.26911000 3.35572300 -0.70499900

H 0 -2.80298600 2.43267700 -0.68413500

H 0 -4.13812000 3.43691600 -1.20838500

N 0 -1.52751500 4.28397300 0.47917000

H 0 -1.19479500 4.79357900 1.28771300

H 0 -0.86887200 3.56476900 0.11220600

C -1 -0.99473000 -0.97601000 7.57848500

H -1 -0.01002400 -0.57709900 7.38448200

H -1 -1.17487500 -1.13340800 8.63166200

H 0 -1.74303000 -0.27258500 7.19693800

C 0 -1.13113200 -2.35670600 6.90746400

H 0 -2.10812900 -2.79120600 7.15928500

H 0 -0.37980000 -3.03779600 7.32925700

C 0 -0.99838000 -2.35388400 5.41657300

N 0 -1.09105800 -3.52307700 4.66949800

C 0 -0.83977800 -1.35524700 4.49310100

H 0 -0.68503200 -0.30151500 4.63613900

C 0 -1.00662000 -3.23834600 3.36109900

H 0 -1.05035800 -3.94332900 2.54614600

N 0 -0.86606600 -1.92412700 3.23386900

H 0 -0.89052500 -1.42657400 2.26957500

C -1 3.68483900 6.35261100 2.05706100

H -1 4.54892500 6.83595200 1.62553700

H -1 3.20918400 6.90504700 2.85392800

H 0 2.93052900 6.26890000 1.26594800

C 0 4.05893300 4.92060800 2.51547400

H 0 5.00274000 4.90958200 3.07015500

H 0 3.26407800 4.53056200 3.16137500

C 0 4.13295200 3.99895600 1.29811500

O 0 3.04882000 3.83064800 0.67889300

O 0 5.24793500 3.49068600 0.94786300

C -1 12.14265800 -2.29969300 -0.48623300

H -1 13.18611100 -2.02788500 -0.54763500

H -1 11.93114100 -3.21887500 -1.01234400

H 0 11.92905300 -2.51008100 0.57123300

C 0 11.23459300 -1.14782400 -0.97404800

H 0 11.68303200 -0.67367400 -1.85935100

H 0 10.26525500 -1.55219200 -1.28691000

C 0 10.99289400 -0.09272900 0.12079800

H 0 10.28469400 -0.50877700 0.85010300

H 0 11.92903500 0.08511700 0.66879100

C 0 10.49669000 1.27527500 -0.37825500

H 0 10.40001800 1.96406800 0.47237600

H 0 11.25981500 1.70801500 -1.03924000

C 0 9.18669100 1.29606200 -1.16723500

H 0 9.01255700 2.29877400 -1.56773100

H 0 9.20752100 0.59672000 -2.00693700

N 0 7.98335600 0.94981400 -0.34583500

H 0 7.12524900 1.28377900 -0.83642400

H 0 7.90221900 -0.08640700 -0.19686000

H 0 7.95692700 1.43891600 0.57961900

Cl 0 7.66660600 -2.14300300 -0.11431600

O 0 1.81960100 1.28587000 -3.52772600

H 0 3.32946100 2.10454700 -3.72703300

O 0 0.32175800 2.47365400 -0.24571300

O 0 0.75378800 3.88595500 2.36990600

H 0 0.31755300 3.02320800 2.24646800

H 0 1.44627300 3.87888400 1.67232000

O 0 -0.31701900 -0.15139500 -4.53297600

H 0 0.44525600 0.43461000 -4.27225200

H 0 -0.04878200 -1.05081900 -4.26689500

O 0 -3.06520400 -1.51853800 -4.07019300

H 0 -3.72207300 -2.00591300 -3.52823900

H 0 -2.30093000 -2.13143800 -4.16455600

O 0 -4.83552800 -2.52655800 -2.04909000

H 0 -4.05469000 -2.13258400 -1.56692000

H 0 -5.10721900 -3.27591900 -1.48850100

O 0 2.36355900 3.68186500 -1.84181700

H 0 2.74663400 3.66577000 -0.91466100

H 0 2.67796400 -0.66160300 -1.75718700

O 0 -0.27162200 -1.70834700 -2.17073200

O 0 5.85852000 2.43934200 -1.43180700

H 0 5.44905100 2.77324200 -0.57838000

H 0 6.32851300 3.21751700 -1.77512000

O 0 4.10392500 2.72356600 -3.61176400

H 0 4.64853900 2.32641800 -2.89817200

H 0 3.10290600 3.49538000 -2.49108200

O 0 7.45942800 2.38387900 2.01657000

H 0 6.65421600 2.88973800 1.72652500

H 0 7.13223800 1.78022100 2.70130900

O 0 2.46124700 -1.29882700 -2.46430400

H 0 2.13710800 0.38827700 -3.27582100

H 0 1.50940100 -1.49120600 -2.29986000

H 0 -1.19617600 -4.45566300 5.04948600

**^8^TS_1_**

E (IEFPCM/B3LYP-D3/SDD,6-31G(d)) = -5854.274688 au

H (IEFPCM/B3LYP-D3/SDD,6-31G(d)) = -5852.723681 au

G (IEFPCM/B3LYP-D3/SDD,6-31G(d)) = -5852.962244 au

E (IEFPCM/B3LYP-D3/SDD,6-311+G(2d,p)//IEFPCM/B3LYP-D3/SDD,6-31G(d)) = -5856.098227 au

Mn 0 1.12947600 1.85506100 -1.99736500

Mn 0 -0.99637600 -2.42664400 -0.84298800

Mn 0 -0.27593200 0.82236300 0.30248100

Mn 0 -2.74586700 -0.46634300 0.41914000

O 0 0.11297300 0.18061800 -1.47150500

O 0 -2.04886600 1.03133200 -0.27672000

O 0 -2.59552700 -1.41248000 -1.11237600

O 0 -1.01921000 -0.95986000 0.85076300

Ca 0 -2.01586200 0.22677300 -2.73401600

C -1 0.51383500 4.34239000 -5.79171900

H -1 0.56429700 5.30023000 -5.29521400

H -1 0.70662600 4.37373300 -6.85366800

H 0 1.32820300 3.74649400 -5.35993000

C 0 -0.81373300 3.61638700 -5.50244600

H 0 -0.95208600 2.78573300 -6.20668400

H 0 -1.68029400 4.27378500 -5.65201800

C 0 -0.93571300 2.98087500 -4.10789200

O 0 -1.97097500 2.29327200 -3.86451200

O 0 0.01857400 3.15790600 -3.29258000

C -1 -2.80662800 -6.93680600 -5.04233000

H -1 -1.83958000 -7.39483900 -5.18872200

H -1 -3.53338800 -7.12298800 -5.81921700

H 0 -3.20529600 -7.37686300 -4.11785400

C 0 -2.51251700 -5.45250700 -4.81516600

H 0 -3.29241400 -4.98312000 -4.20118800

H 0 -2.47503600 -4.89619400 -5.75828200

C 0 -1.14192500 -5.34715300 -4.11652600

H 0 -0.34849700 -5.49836300 -4.85789800

H 0 -1.05289900 -6.12124200 -3.34734600

C 0 -1.00443400 -3.98972000 -3.47502300

O 0 -0.89485500 -2.99086900 -4.23069800

O 0 -1.11318700 -3.98774400 -2.20048900

C -1 5.13121000 -4.50169300 1.21592500

H 0 5.89017500 -5.23931600 1.50214500

H 0 5.48816700 -3.99240700 0.31719600

C 0 4.94876800 -3.51032000 2.36101800

O 0 4.32228300 -3.82999700 3.39299500

C 0 3.80087800 -5.26657500 0.93410600

H 0 3.59117600 -5.90419600 1.80165600

H 0 3.94648300 -5.92608800 0.07392900

C 0 2.60978200 -4.38303400 0.67781000

N 0 2.28123500 -3.38710700 1.58334900

H 0 2.74517000 -3.27009000 2.48137100

C 0 1.66623600 -4.26747800 -0.32075700

H 0 1.54515500 -4.84196800 -1.22637600

C 0 1.23288300 -2.69511600 1.11695700

H 0 0.85955200 -1.79615900 1.56356900

N 0 0.80834600 -3.21357700 -0.02617500

N 0 5.37431700 -2.25794300 2.13053400

H 0 5.98105600 -2.09529200 1.32026400

C -1 5.05240500 -1.14334500 3.01321500

H 0 5.91335100 -0.46277300 3.00857800

H 0 4.93992500 -1.53386900 4.02762100

C 0 3.78814300 -0.38388800 2.58959100

H 0 3.58840600 0.40961300 3.32033500

H 0 2.92881800 -1.05635400 2.63024100

C 0 3.92826300 0.20370600 1.18775700

H 0 4.45399400 -0.49713500 0.52840700

H 0 4.53656700 1.11681900 1.20017700

C 0 2.64265200 0.54601400 0.45497600

O 0 2.77558300 0.87034700 -0.75108100

O 0 1.53208400 0.44205300 1.08807700

C -1 -5.38566200 -3.94492300 2.38690100

H 0 -5.24780100 -3.14718500 3.12325900

H 0 -5.85533100 -4.79311600 2.90018500

C 0 -4.03166600 -4.39190500 1.80717900

H 0 -4.17790400 -5.18109500 1.06482800

H 0 -3.41633800 -4.82563500 2.60529600

C 0 -3.20158400 -3.28880700 1.13882300

O 0 -3.58331700 -2.09475800 1.32674500

O 0 -2.16717400 -3.64628500 0.49272500

C 0 -6.32693200 -3.48413300 1.27790100

O 0 -6.18627000 -3.83894200 0.10021100

N 0 -7.37111900 -2.71836300 1.67657100

H 0 -7.38048500 -2.35711700 2.62074400

C -1 -8.37877100 -2.22087300 0.74777900

H 0 -9.32652500 -2.11406100 1.27796200

H 0 -8.48901300 -2.95749600 -0.05173100

C 0 -8.00357700 -0.84076600 0.17959800

O 0 -8.50677300 0.18982700 0.63208000

N 0 -7.09943600 -0.87078800 -0.82808900

H 0 -6.62734200 -1.73994100 -1.06392100

C 0 -6.52344500 0.33035200 -1.39626500

H 0 -6.70935900 0.37998800 -2.47265600

H 0 -7.00641000 1.18802600 -0.91815500

C 0 -5.00790800 0.41648000 -1.17899100

O 0 -4.30078800 0.83151700 -2.11797300

O 0 -4.60403200 0.05543100 -0.01156400

C -1 -3.47503600 3.81991500 4.82364800

H -1 -3.50455200 3.56970100 5.87384900

H -1 -4.29937300 4.44010900 4.50395200

H 0 -2.55419400 4.39498000 4.65747700

C 0 -3.50584400 2.61780600 3.89951600

H 0 -3.49165100 2.98808200 2.86607500

H 0 -4.44293100 2.06006300 4.00332600

C 0 -2.31587400 1.67274400 4.11662000

H 0 -2.58907900 0.87989000 4.81976500

H 0 -1.44733400 2.20994700 4.50895300

C 0 -1.96766900 1.09224000 2.77249100

O 0 -0.85341700 1.40619700 2.26172200

O 0 -2.87394700 0.37070600 2.24054900

C -1 -5.61744600 9.06845400 1.60180600

H -1 -5.47799900 8.70407400 2.60887400

H -1 -6.65531600 9.28315600 1.39423200

H 0 -5.07974300 10.02154100 1.52215700

C 0 -5.03659100 8.04490700 0.62055000

H 0 -3.94228600 8.06171500 0.72194400

H 0 -5.25141100 8.33302600 -0.41702800

C 0 -5.57166000 6.63192300 0.88591600

H 0 -6.65721800 6.61692600 0.72710200

H 0 -5.40071200 6.36307300 1.93732700

C 0 -4.95210100 5.55081800 0.00076300

H 0 -5.13428600 5.77893900 -1.06007100

H 0 -5.41999400 4.58613000 0.22256600

N 0 -3.51785600 5.42504000 0.26036100

H 0 -3.00336700 6.27207100 0.45847700

C 0 -2.77475700 4.36064000 -0.11238200

N 0 -3.32762400 3.34460300 -0.77359900

H 0 -2.87125800 2.41823200 -0.73256900

H 0 -4.21553500 3.42054100 -1.24363200

N 0 -1.47382600 4.32025300 0.18362600

H 0 -1.08518100 4.79329300 0.99309100

H 0 -0.87303300 3.54474100 -0.16891100

C -1 -0.96808400 -0.99497900 7.58244400

H -1 0.01542900 -0.59283700 7.38904800

H -1 -1.14632400 -1.15828400 8.63502200

H 0 -1.72174000 -0.29839000 7.20027000

C 0 -1.09872600 -2.38430700 6.92817900

H 0 -2.06675500 -2.82636100 7.20103700

H 0 -0.33347700 -3.05205300 7.34627500

C 0 -0.99186000 -2.39285000 5.43773300

N 0 -1.08108100 -3.56562000 4.69711600

C 0 -0.87013500 -1.39408500 4.50962000

H 0 -0.72837900 -0.33765900 4.64800000

C 0 -1.03081700 -3.28158900 3.38631300

H 0 -1.07437800 -3.98877300 2.57353400

N 0 -0.91673500 -1.96571300 3.25326000

H 0 -0.96126900 -1.46308100 2.28252200

C -1 3.68691100 6.37297900 2.09262700

H -1 4.54923500 6.86048400 1.66233400

H -1 3.21114700 6.92012600 2.89304900

H 0 2.93147000 6.30999000 1.29924000

C 0 4.03732100 4.92675800 2.52394300

H 0 4.97740300 4.89119000 3.08384700

H 0 3.23411600 4.54135200 3.16260700

C 0 4.10797100 4.00528500 1.30232200

O 0 3.03230700 3.83580900 0.66868700

O 0 5.22385700 3.48454800 0.96987900

C -1 12.16028700 -2.24689100 -0.50846100

H -1 13.20302000 -1.97242200 -0.56990600

H -1 11.95001600 -3.16378400 -1.03901600

H 0 11.95047300 -2.46501700 0.54834400

C 0 11.25186300 -1.09091000 -0.98155400

H 0 11.68690800 -0.61995300 -1.87519700

H 0 10.27400700 -1.48872300 -1.27543500

C 0 11.04173000 -0.03516800 0.11897000

H 0 10.35120000 -0.44889700 0.86646400

H 0 11.99198600 0.13818900 0.64358600

C 0 10.53810200 1.33475100 -0.36617100

H 0 10.45928000 2.02124100 0.48814300

H 0 11.28783300 1.76866600 -1.04149200

C 0 9.21259300 1.35628600 -1.12772900

H 0 9.02266700 2.36226600 -1.51265600

H 0 9.22156200 0.66721800 -1.97614300

N 0 8.02923700 0.98957800 -0.28746700

H 0 7.15754900 1.30789000 -0.76738800

H 0 7.96356800 -0.04756500 -0.14109800

H 0 8.00364400 1.47817200 0.63729000

Cl 0 7.69762700 -2.10494500 -0.09963200

O 0 1.87409500 1.03467500 -3.55334700

H 0 3.40345700 1.85109600 -3.74370100

O 0 0.30084600 2.38086500 -0.41506200

O 0 0.63962900 3.99832300 2.21622100

H 0 0.23446100 3.11345200 2.18650600

H 0 1.37739000 3.93577900 1.56804200

O 0 -0.40824200 -0.19943500 -4.58367500

H 0 0.42310200 0.27104200 -4.31129400

H 0 -0.26224900 -1.14319200 -4.35958300

O 0 -3.17111100 -1.46452400 -4.08497100

H 0 -3.81095000 -1.96695800 -3.53722100

H 0 -2.41632400 -2.08441300 -4.23367300

O 0 -4.81473300 -2.53628900 -2.02270600

H 0 -4.01871600 -2.15274200 -1.56144400

H 0 -5.09660300 -3.27193800 -1.44859800

O 0 2.44257700 3.50413000 -1.90085400

H 0 2.77763300 3.53173600 -0.96014800

H 0 2.84795700 -0.80806600 -1.70612800

O 0 -0.11182300 -1.45419800 -2.09153500

O 0 5.85188300 2.36944400 -1.36904700

H 0 5.44269300 2.73568000 -0.52805700

H 0 6.26722600 3.14742900 -1.77708600

O 0 4.19376900 2.44708300 -3.63108500

H 0 4.70038400 2.06622100 -2.88356300

H 0 3.20431500 3.28863600 -2.50743400

O 0 7.44844300 2.42064800 2.06789500

H 0 6.63949400 2.91098100 1.76325500

H 0 7.12040200 1.81400500 2.74953000

O 0 2.60684000 -1.44278100 -2.40733200

H 0 2.20189700 0.14454900 -3.28149900

H 0 1.64315500 -1.58626800 -2.24300000

H 0 -1.16312200 -4.49845900 5.08190600

**^2'^TS_1_**

E (IEFPCM/B3LYP-D3/SDD,6-31G(d)) = -5854.277167 au

H (IEFPCM/B3LYP-D3/SDD,6-31G(d)) = -5852.725878 au

G (IEFPCM/B3LYP-D3/SDD,6-31G(d)) = -5852.962905 au

E (IEFPCM/B3LYP-D3/SDD,6-311+G(2d,p)//IEFPCM/B3LYP-D3/SDD,6-31G(d)) = -5856.100619 au

Mn 0 1.13226600 1.85309600 -2.00188800

Mn 0 -1.01134300 -2.43625900 -0.84530500

Mn 0 -0.27684700 0.81894800 0.29339100

Mn 0 -2.74466400 -0.46623400 0.41709800

O 0 0.11577900 0.18095900 -1.47297800

O 0 -2.04659400 1.02970400 -0.27770400

O 0 -2.61079800 -1.41408300 -1.11312700

O 0 -1.02212900 -0.95489000 0.84728800

Ca 0 -2.01904900 0.22498000 -2.73333700

C -1 0.51629600 4.32941500 -5.80343400

H -1 0.56672900 5.28840600 -5.30915200

H -1 0.70918700 4.35828600 -6.86543500

H 0 1.33062500 3.73458100 -5.37012700

C 0 -0.81095400 3.60463200 -5.51051500

H 0 -0.94986700 2.77079900 -6.21083700

H 0 -1.67782400 4.26116100 -5.66185000

C 0 -0.93051200 2.97523900 -4.11304000

O 0 -1.96631900 2.29088800 -3.86334200

O 0 0.02714400 3.15381300 -3.30181700

C -1 -2.80442700 -6.94795200 -5.02813000

H -1 -1.83737400 -7.40634100 -5.17336600

H -1 -3.53111800 -7.13592900 -5.80464900

H 0 -3.20302300 -7.38493800 -4.10222400

C 0 -2.51096100 -5.46268400 -4.80672900

H 0 -3.28938800 -4.99237600 -4.19167100

H 0 -2.47749400 -4.90932100 -5.75172600

C 0 -1.13804900 -5.35298800 -4.11303800

H 0 -0.34646100 -5.50072100 -4.85695100

H 0 -1.04366800 -6.12674000 -3.34422400

C 0 -1.00538200 -3.99519600 -3.47201900

O 0 -0.89780900 -2.99582600 -4.22653600

O 0 -1.11773300 -3.99424300 -2.19724900

C -1 5.13286400 -4.49842600 1.22519000

H 0 5.89494300 -5.23301300 1.51108900

H 0 5.48594700 -3.99035900 0.32424600

C 0 4.95088800 -3.50457300 2.36839900

O 0 4.32452100 -3.82179100 3.40112000

C 0 3.80399800 -5.26761800 0.94935500

H 0 3.59707400 -5.90124500 1.82051800

H 0 3.94980000 -5.93125600 0.09235300

C 0 2.61106000 -4.38762300 0.68982400

N 0 2.28206000 -3.38639200 1.58916900

H 0 2.74593700 -3.26344100 2.48623000

C 0 1.66624000 -4.27932100 -0.30845100

H 0 1.54635300 -4.85988900 -1.21045600

C 0 1.23194700 -2.69900600 1.11848300

H 0 0.85852100 -1.79742700 1.55962100

N 0 0.80697200 -3.22472900 -0.02088800

N 0 5.37756300 -2.25294100 2.13565100

H 0 5.98375600 -2.09210800 1.32465800

C -1 5.05394800 -1.13590600 3.01465900

H 0 5.91431600 -0.45460900 3.00881700

H 0 4.94049600 -1.52328900 4.03014400

C 0 3.78940900 -0.37905800 2.58671700

H 0 3.58833100 0.41706100 3.31423300

H 0 2.93054100 -1.05205100 2.62889600

C 0 3.93003500 0.20353300 1.18274600

H 0 4.45582500 -0.49967900 0.52595100

H 0 4.53822400 1.11673100 1.19217400

C 0 2.64421600 0.54326800 0.44869000

O 0 2.77785500 0.86789200 -0.75758300

O 0 1.53363300 0.43686500 1.08027700

C -1 -5.38410700 -3.93875800 2.39388100

H 0 -5.24550300 -3.13792900 3.12668600

H 0 -5.85207300 -4.78531400 2.91137000

C 0 -4.03074400 -4.38708500 1.81345400

H 0 -4.17770600 -5.17943200 1.07466200

H 0 -3.41321900 -4.81673100 2.61204900

C 0 -3.20465400 -3.28511200 1.13900000

O 0 -3.58634200 -2.09135800 1.32368300

O 0 -2.17170100 -3.64349600 0.48924100

C 0 -6.32718300 -3.48346700 1.28411300

O 0 -6.18816900 -3.84456100 0.10818900

N 0 -7.37035200 -2.71517700 1.68065200

H 0 -7.37744800 -2.34876400 2.62287900

C -1 -8.37703300 -2.21847400 0.75047400

H 0 -9.32654300 -2.11513600 1.27828200

H 0 -8.48305300 -2.95379000 -0.05071100

C 0 -8.00312800 -0.83602900 0.18716200

O 0 -8.50523300 0.19248000 0.64566100

N 0 -7.10112400 -0.86138200 -0.82248000

H 0 -6.62891200 -1.72908600 -1.06404000

C 0 -6.52593200 0.34263700 -1.38602800

H 0 -6.71582600 0.39834400 -2.46144200

H 0 -7.00617200 1.19793700 -0.90104200

C 0 -5.00931400 0.42442700 -1.17324700

O 0 -4.30315200 0.83248000 -2.11647600

O 0 -4.60372900 0.06681600 -0.00590400

C -1 -3.47357900 3.83169400 4.81274500

H -1 -3.50319800 3.58392200 5.86352200

H -1 -4.29787500 4.45115600 4.49153000

H 0 -2.55388300 4.40916800 4.64773900

C 0 -3.49810200 2.62967500 3.88876700

H 0 -3.48294300 3.00000600 2.85531500

H 0 -4.43286600 2.06780300 3.99054900

C 0 -2.30457400 1.69048500 4.10859700

H 0 -2.57223000 0.90408000 4.82118400

H 0 -1.43573200 2.23394200 4.49159100

C 0 -1.96096000 1.09684000 2.76885500

O 0 -0.84505000 1.40257500 2.25523700

O 0 -2.86941300 0.37307900 2.24516500

C -1 -5.61559700 9.07276200 1.57850600

H -1 -5.47625200 8.71072200 2.58643200

H -1 -6.65344400 9.28699800 1.37033500

H 0 -5.07628000 10.02443200 1.49434700

C 0 -5.03792500 8.04209300 0.60262700

H 0 -3.94337300 8.05757200 0.70151600

H 0 -5.25406900 8.32293800 -0.43662900

C 0 -5.57584500 6.63221400 0.87966500

H 0 -6.66079900 6.61708800 0.71711300

H 0 -5.40927600 6.37328800 1.93429300

C 0 -4.95419700 5.54167700 0.00799800

H 0 -5.12812000 5.76196500 -1.05592100

H 0 -5.42692900 4.58029700 0.23438700

N 0 -3.52209600 5.41421700 0.27905000

H 0 -3.00966600 6.26054500 0.48550600

C 0 -2.77568600 4.35531600 -0.10298400

N 0 -3.32476900 3.34408900 -0.77448800

H 0 -2.86780000 2.41788300 -0.73654500

H 0 -4.21874300 3.41800000 -1.23307100

N 0 -1.47457700 4.31601500 0.19251800

H 0 -1.08626600 4.78397300 1.00511100

H 0 -0.87322100 3.54358300 -0.16522500

C -1 -0.96696700 -0.97681500 7.58296600

H -1 0.01657100 -0.57513900 7.38872700

H -1 -1.14530800 -1.13766800 8.63590300

H 0 -1.72052500 -0.28096000 7.19925000

C 0 -1.09772400 -2.36742700 6.93137100

H 0 -2.06598500 -2.80882100 7.20441300

H 0 -0.33276400 -3.03464500 7.35083700

C 0 -0.99020600 -2.37783900 5.44102400

N 0 -1.08231900 -3.55076800 4.70093000

C 0 -0.86509400 -1.37980800 4.51268500

H 0 -0.72076500 -0.32365300 4.65038200

C 0 -1.03051400 -3.26773600 3.39004300

H 0 -1.07495000 -3.97510700 2.57744700

N 0 -0.91243900 -1.95221900 3.25682600

H 0 -0.95578600 -1.45111500 2.28825000

C -1 3.68866800 6.37828000 2.07646600

H -1 4.55104000 6.86476800 1.64512200

H -1 3.21283700 6.92729500 2.87556900

H 0 2.93334400 6.31226500 1.28326500

C 0 4.03937800 4.93369800 2.51342400

H 0 4.97972200 4.90062300 3.07304300

H 0 3.23651000 4.55093000 3.15410100

C 0 4.10950600 4.00707400 1.29581400

O 0 3.03325700 3.83356300 0.66423300

O 0 5.22571000 3.48642400 0.96424300

C -1 12.16214200 -2.24775700 -0.50377500

H -1 13.20488500 -1.97345000 -0.56576000

H -1 11.95190500 -3.16587800 -1.03221500

H 0 11.95228800 -2.46352400 0.55351300

C 0 11.25406000 -1.09250100 -0.97920900

H 0 11.68883900 -0.62384200 -1.87418600

H 0 10.27576700 -1.49042200 -1.27151700

C 0 11.04541200 -0.03421500 0.11914400

H 0 10.35567800 -0.44612100 0.86838900

H 0 11.99629900 0.14005800 0.64231200

C 0 10.54152700 1.33469700 -0.36845900

H 0 10.46365100 2.02306100 0.48442800

H 0 11.29061400 1.76705000 -1.04549500

C 0 9.21528300 1.35454600 -1.12871800

H 0 9.02471200 2.35975300 -1.51533900

H 0 9.22364000 0.66390200 -1.97585900

N 0 8.03281400 0.98901200 -0.28673300

H 0 7.16070700 1.30639500 -0.76661000

H 0 7.96730300 -0.04789300 -0.13872000

H 0 8.00767100 1.47923700 0.63712200

Cl 0 7.69945000 -2.10530600 -0.09663200

O 0 1.87451900 1.02900800 -3.55639900

H 0 3.40723000 1.84143500 -3.74535800

O 0 0.30022400 2.38001700 -0.42163700

O 0 0.64200400 3.98915000 2.21822500

H 0 0.23745200 3.10376600 2.18861400

H 0 1.37757100 3.92869000 1.56749600

O 0 -0.41103100 -0.20381400 -4.58267400

H 0 0.42089300 0.26671200 -4.31230000

H 0 -0.26444000 -1.14758900 -4.35973600

O 0 -3.17546400 -1.46837100 -4.08186800

H 0 -3.81657100 -1.96842500 -3.53356300

H 0 -2.42141800 -2.08938900 -4.22858200

O 0 -4.83173700 -2.53515000 -2.01864000

H 0 -4.03636100 -2.15157800 -1.55649000

H 0 -5.11177800 -3.27276600 -1.44599200

O 0 2.44838300 3.50170000 -1.90589300

H 0 2.78174900 3.52939800 -0.96455100

H 0 2.83470000 -0.81730800 -1.70743100

O 0 -0.13080900 -1.46345700 -2.08934200

O 0 5.85515100 2.36526800 -1.37046200

H 0 5.44559800 2.73390200 -0.53064700

H 0 6.26933200 3.14232100 -1.78143200

O 0 4.19868500 2.43573800 -3.63294500

H 0 4.70343300 2.05563800 -2.88380600

H 0 3.21052400 3.28301800 -2.51066800

O 0 7.45045500 2.42523700 2.06533700

H 0 6.64168700 2.91476300 1.75902300

H 0 7.12202200 1.82053300 2.74850500

O 0 2.59179600 -1.45173800 -2.40824700

H 0 2.19795700 0.13769900 -3.28309400

H 0 1.62872900 -1.59472400 -2.24208800

H 0 -1.16664100 -4.48315500 5.08630500

**^8'^TS_1_**

E (IEFPCM/B3LYP-D3/SDD,6-31G(d)) = -5854.277032 au

H (IEFPCM/B3LYP-D3/SDD,6-31G(d)) = -5852.725498 au

G (IEFPCM/B3LYP-D3/SDD,6-31G(d)) = -5852.963854 au

E (IEFPCM/B3LYP-D3/SDD,6-311+G(2d,p)//IEFPCM/B3LYP-D3/SDD,6-31G(d)) = -5856.100216 au

Mn 0 1.16750500 1.82703900 -2.02327800

Mn 0 -1.02213500 -2.44750700 -0.83316900

Mn 0 -0.28563700 0.82932800 0.27609300

Mn 0 -2.75076200 -0.45637800 0.42168300

O 0 0.09540300 0.15561300 -1.47228600

O 0 -2.05290800 1.04044500 -0.27835500

O 0 -2.62229500 -1.41196100 -1.10340400

O 0 -1.03110300 -0.94560600 0.85224800

Ca 0 -2.02081600 0.19727500 -2.72789000

C -1 0.52117300 4.29571200 -5.83884800

H -1 0.57291500 5.25785400 -5.35086500

H -1 0.71470500 4.31726800 -6.90090600

H 0 1.34154600 3.70998300 -5.40491700

C 0 -0.79634400 3.56685000 -5.51971100

H 0 -0.94878000 2.73252100 -6.21683000

H 0 -1.67053100 4.21831200 -5.64571400

C 0 -0.87479200 2.93070500 -4.12245700

O 0 -1.91408000 2.27465900 -3.82919900

O 0 0.12924500 3.07839700 -3.35863100

C -1 -2.81863400 -6.97080700 -4.99118000

H -1 -1.85225900 -7.43173800 -5.13284200

H -1 -3.54520000 -7.16269500 -5.76685900

H 0 -3.21834300 -7.39997900 -4.06215000

C 0 -2.52227200 -5.48497300 -4.78200000

H 0 -3.29871900 -5.00838800 -4.16931400

H 0 -2.48928900 -4.93892000 -5.73124700

C 0 -1.14802200 -5.37256000 -4.09144400

H 0 -0.35745900 -5.52245300 -4.83591600

H 0 -1.05213300 -6.14273300 -3.31924700

C 0 -1.02015800 -4.01155900 -3.45768800

O 0 -0.91245700 -3.01731300 -4.21918400

O 0 -1.14130500 -4.00386900 -2.18384500

C -1 5.11920000 -4.49324700 1.25039700

H 0 5.88705900 -5.22238900 1.53497600

H 0 5.46327500 -3.98964600 0.34348100

C 0 4.94198800 -3.49248400 2.38874600

O 0 4.31765300 -3.80273600 3.42467300

C 0 3.79175500 -5.26933400 0.99096800

H 0 3.59075800 -5.89270000 1.87085400

H 0 3.93454400 -5.94281700 0.14115400

C 0 2.59727100 -4.39359900 0.72618000

N 0 2.27265800 -3.37922600 1.61226000

H 0 2.73748400 -3.24618300 2.50719900

C 0 1.65004400 -4.29757300 -0.27082400

H 0 1.52833700 -4.88967300 -1.16511000

C 0 1.22219800 -2.69666000 1.13442000

H 0 0.85011000 -1.78893300 1.56437400

N 0 0.79355200 -3.23771200 0.00389800

N 0 5.37048200 -2.24280100 2.14830000

H 0 5.97481500 -2.08736900 1.33486300

C -1 5.04484500 -1.11888600 3.01763500

H 0 5.90624600 -0.43892500 3.01173700

H 0 4.92332800 -1.49802900 4.03513200

C 0 3.78276200 -0.36291600 2.57517500

H 0 3.58260400 0.44291000 3.29218400

H 0 2.92202200 -1.03302600 2.62541900

C 0 3.92184000 0.20403100 1.16323800

H 0 4.43890800 -0.50949000 0.51049800

H 0 4.53635600 1.11299900 1.16146500

C 0 2.63237800 0.54736800 0.43580100

O 0 2.76159100 0.86099700 -0.77749800

O 0 1.52687100 0.45423100 1.07219500

C -1 -5.39748400 -3.90853500 2.40937900

H 0 -5.25583700 -3.10175800 3.13500700

H 0 -5.86933900 -4.74826000 2.93454300

C 0 -4.04626200 -4.36730500 1.83401000

H 0 -4.19554200 -5.16416400 1.10057500

H 0 -3.43090700 -4.79333700 2.63622200

C 0 -3.21681700 -3.27226000 1.15374200

O 0 -3.59503900 -2.07667200 1.33266900

O 0 -2.18518200 -3.63805700 0.50619000

C 0 -6.33916200 -3.45970400 1.29588900

O 0 -6.20123600 -3.83073800 0.12296600

N 0 -7.38056400 -2.68581500 1.68649600

H 0 -7.38605000 -2.31144900 2.62560900

C -1 -8.38664100 -2.19417700 0.75297100

H 0 -9.33606200 -2.08587800 1.27995500

H 0 -8.49346900 -2.93484600 -0.04317200

C 0 -8.01081400 -0.81610700 0.17997800

O 0 -8.51455700 0.21589400 0.62890500

N 0 -7.10547100 -0.84909300 -0.82638600

H 0 -6.63241600 -1.71850100 -1.06038900

C 0 -6.52707200 0.35055500 -1.39613300

H 0 -6.71221400 0.39841600 -2.47276100

H 0 -7.00925000 1.20962200 -0.91978400

C 0 -5.01110100 0.43344700 -1.17707600

O 0 -4.30119800 0.83831600 -2.11943500

O 0 -4.61046300 0.08034800 -0.00710100

C -1 -3.47545800 3.87451800 4.77810100

H -1 -3.50607400 3.63372200 5.83047100

H -1 -4.29854800 4.49321000 4.45234300

H 0 -2.55366100 4.44726000 4.60878400

C 0 -3.50459500 2.66453000 3.86510400

H 0 -3.47794000 3.02515000 2.82838600

H 0 -4.44589200 2.11328700 3.96485600

C 0 -2.32221000 1.71573000 4.10370300

H 0 -2.60404700 0.93485600 4.81696900

H 0 -1.45312300 2.25410600 4.49345600

C 0 -1.97047200 1.11481600 2.76939100

O 0 -0.85413800 1.41819900 2.25790500

O 0 -2.87757300 0.38720800 2.24665500

C -1 -5.60699400 9.09769600 1.50819200

H -1 -5.46881200 8.74207400 2.51856000

H -1 -6.64436900 9.31226900 1.29802400

H 0 -5.06427400 10.04639100 1.41493000

C 0 -5.03106500 8.05225300 0.54522900

H 0 -3.93581500 8.07267600 0.63569800

H 0 -5.25508500 8.31336600 -0.49744000

C 0 -5.56185600 6.64499400 0.85107000

H 0 -6.64729600 6.62241900 0.69310800

H 0 -5.39105300 6.40675000 1.90995900

C 0 -4.94063000 5.53891400 -0.00135400

H 0 -5.10719500 5.74663300 -1.06899700

H 0 -5.42110900 4.58352000 0.23505300

N 0 -3.50996200 5.40644000 0.27858000

H 0 -3.00011000 6.24873600 0.50745500

C 0 -2.76000500 4.36005100 -0.13079100

N 0 -3.30441700 3.36333900 -0.82830500

H 0 -2.85878100 2.43236800 -0.78362100

H 0 -4.22044900 3.42966700 -1.24219600

N 0 -1.45593900 4.32145000 0.15436000

H 0 -1.06507800 4.76393600 0.98079500

H 0 -0.86046000 3.55140400 -0.21543700

C -1 -0.97835400 -0.91976900 7.58136100

H -1 0.00595600 -0.52100500 7.38504200

H -1 -1.15755000 -1.07338900 8.63523300

H 0 -1.73045700 -0.22539700 7.19214500

C 0 -1.11100300 -2.31468900 6.93965200

H 0 -2.08038700 -2.75233900 7.21479500

H 0 -0.34773800 -2.98032600 7.36475300

C 0 -1.00183100 -2.33501700 5.44970700

N 0 -1.09714500 -3.51213900 4.71689900

C 0 -0.87218600 -1.34317200 4.51537100

H 0 -0.72516500 -0.28654100 4.64678300

C 0 -1.04280800 -3.23689300 3.40427700

H 0 -1.08902100 -3.94926700 2.59615500

N 0 -0.91982400 -1.92279200 3.26293300

H 0 -0.96295300 -1.42659400 2.28851500

C -1 3.69252100 6.39120000 2.02919400

H -1 4.55593800 6.87341300 1.59514700

H -1 3.21715300 6.94625100 2.82439300

H 0 2.93810200 6.30998700 1.23703100

C 0 4.04716900 4.95595200 2.49575200

H 0 4.98933500 4.93808800 3.05296800

H 0 3.24767600 4.58715000 3.14877000

C 0 4.11496800 4.00167500 1.30077100

O 0 3.03600600 3.80342000 0.68123800

O 0 5.23386900 3.48407400 0.97237700

C -1 12.15315700 -2.26562000 -0.48934500

H -1 13.19638600 -1.99344800 -0.55254100

H -1 11.94169700 -3.18685300 -1.01184700

H 0 11.94302500 -2.47462100 0.56931300

C 0 11.24878500 -1.11041600 -0.96996600

H 0 11.67801300 -0.65502600 -1.87441800

H 0 10.26480300 -1.50466700 -1.24782800

C 0 11.06032200 -0.03873100 0.11886300

H 0 10.37968600 -0.43843900 0.88300600

H 0 12.01916800 0.13660000 0.62692900

C 0 10.55606600 1.32685000 -0.37705200

H 0 10.48897800 2.02374000 0.46976800

H 0 11.29939100 1.75021800 -1.06599000

C 0 9.22251000 1.34235500 -1.12405800

H 0 9.02686500 2.34578100 -1.51280900

H 0 9.22340300 0.64845200 -1.96857200

N 0 8.04859900 0.97828500 -0.26975700

H 0 7.17240600 1.29440300 -0.74344000

H 0 7.98283200 -0.05817100 -0.11990900

H 0 8.02967600 1.47123800 0.65242800

Cl 0 7.68828400 -2.11413300 -0.08879800

O 0 1.91056200 0.98040700 -3.55095300

H 0 3.45116900 1.80291000 -3.73906300

O 0 0.30743700 2.37153500 -0.48383700

O 0 0.62531900 3.99149400 2.20449000

H 0 0.22440600 3.10354200 2.19374600

H 0 1.37270700 3.91904300 1.56843500

O 0 -0.40620400 -0.23933300 -4.57444000

H 0 0.43108400 0.21836000 -4.30376100

H 0 -0.27444000 -1.18507900 -4.34804200

O 0 -3.18653600 -1.48816300 -4.07746800

H 0 -3.82794400 -1.98361200 -3.52482100

H 0 -2.43470300 -2.11226200 -4.22333500

O 0 -4.84082300 -2.53150600 -2.00747500

H 0 -4.04532400 -2.14598300 -1.54550600

H 0 -5.12032600 -3.26843800 -1.43378500

O 0 2.50225100 3.51395800 -1.91103300

H 0 2.82055900 3.52679100 -0.96619800

H 0 2.83794200 -0.87058200 -1.68556600

O 0 -0.13104900 -1.48889900 -2.07407000

O 0 5.86838100 2.34483600 -1.34656700

H 0 5.45785700 2.72207800 -0.51050300

H 0 6.27969700 3.11817700 -1.76732700

O 0 4.24808100 2.38749600 -3.62811800

H 0 4.73678400 2.01298500 -2.86599500

H 0 3.26630700 3.27873100 -2.50318300

O 0 7.46158500 2.42453400 2.07600200

H 0 6.65411000 2.91385900 1.76700700

H 0 7.13132700 1.82333500 2.76136200

O 0 2.58505500 -1.49846400 -2.38772100

H 0 2.22819900 0.09032200 -3.26424600

H 0 1.61906200 -1.62909600 -2.22136700

H 0 -1.18505900 -4.44178100 5.10793900

**^8^L_P**

E (IEFPCM/B3LYP-D3/SDD,6-31G(d)) = -5854.305274 au

H (IEFPCM/B3LYP-D3/SDD,6-31G(d)) = -5852.750372 au

G (IEFPCM/B3LYP-D3/SDD,6-31G(d)) = -5852.990499 au

E (IEFPCM/B3LYP-D3/SDD,6-311+G(2d,p)//IEFPCM/B3LYP-D3/SDD,6-31G(d)) = -5856.130284 au

Mn 0 1.15499100 1.98766400 -1.91949500

Mn 0 -0.78320800 -2.29213100 -0.89883800

Mn 0 -0.24150600 0.76524400 0.30963300

Mn 0 -2.73713900 -0.57591700 0.28197600

O 0 0.04490200 0.35183400 -1.79073900

O 0 -2.08780500 0.96018100 -0.28994100

O 0 -2.41567500 -1.43129800 -1.31491000

O 0 -0.96193300 -1.09707800 0.67432200

Ca 0 -2.13329200 0.53119700 -2.80637300

C -1 0.53079100 4.65322800 -5.53170200

H -1 0.58799400 5.58171600 -4.98292300

H -1 0.72192300 4.74229300 -6.59067400

H 0 1.32007900 4.01131000 -5.12351000

C 0 -0.82139000 3.95546900 -5.32123400

H 0 -0.95815200 3.16228200 -6.06838000

H 0 -1.66446900 4.64305000 -5.46954700

C 0 -1.00461900 3.26472900 -3.96635400

O 0 -2.08303100 2.61419500 -3.79683600

O 0 -0.07517700 3.34895700 -3.11586200

C -1 -2.85753700 -6.62993800 -5.40239100

H -1 -1.89357500 -7.08496600 -5.57597700

H -1 -3.58677600 -6.76833300 -6.18689900

H 0 -3.27017200 -7.14566400 -4.52193500

C 0 -2.61941300 -5.16018200 -5.04551100

H 0 -3.54755500 -4.67862300 -4.71259700

H 0 -2.26241300 -4.59410400 -5.91331100

C 0 -1.58028900 -5.09268000 -3.92746900

H 0 -0.62765900 -5.52276300 -4.27214100

H 0 -1.89471800 -5.70030600 -3.07128100

C 0 -1.27686500 -3.69673400 -3.42100800

O 0 -1.44540300 -2.69718100 -4.13156000

O 0 -0.80251400 -3.69718500 -2.21048300

C -1 5.10596400 -4.59381300 0.96484100

H 0 5.90339700 -5.32109800 1.16084200

H 0 5.39735200 -4.02003400 0.08153800

C 0 4.96657700 -3.67945400 2.17954300

O 0 4.39818800 -4.07602100 3.21905400

C 0 3.78732100 -5.37418800 0.70979200

H 0 3.61915700 -6.05317800 1.55392100

H 0 3.89935600 -5.98601000 -0.18939600

C 0 2.60448900 -4.46834700 0.54862500

N 0 2.27167800 -3.58382500 1.56406500

H 0 2.74422600 -3.56448100 2.46672700

C 0 1.72920400 -4.18046300 -0.47144100

H 0 1.62112200 -4.62845200 -1.44655300

C 0 1.29064300 -2.77935500 1.14113500

H 0 0.93013100 -1.92531500 1.67785700

N 0 0.91262100 -3.12899100 -0.08059500

N 0 5.36381700 -2.40957700 2.00552700

H 0 5.93638100 -2.19543700 1.18285800

C -1 5.05090000 -1.33996600 2.94585600

H 0 5.92863500 -0.68339900 3.00458400

H 0 4.90248100 -1.78795100 3.93109000

C 0 3.81540400 -0.53174500 2.53070400

H 0 3.62458300 0.24116900 3.28566700

H 0 2.94359600 -1.18764400 2.53953600

C 0 3.97014100 0.10287700 1.14875800

H 0 4.47594900 -0.58586800 0.46107200

H 0 4.59718400 1.00213900 1.18999800

C 0 2.68049800 0.49380800 0.44469700

O 0 2.79736400 0.92865800 -0.72798700

O 0 1.57547500 0.31543200 1.06666600

C -1 -5.40524000 -4.03945200 2.18673600

H 0 -5.21835600 -3.26708100 2.93994500

H 0 -5.93131800 -4.86313800 2.68667600

C 0 -4.09249100 -4.54638000 1.59670300

H 0 -4.28402700 -5.28955200 0.81686900

H 0 -3.50929900 -5.05620200 2.37336700

C 0 -3.19414500 -3.47076800 0.98033700

O 0 -3.58270000 -2.26016000 1.09648300

O 0 -2.12287100 -3.85464300 0.44967500

C 0 -6.33172500 -3.48639900 1.10680200

O 0 -6.19117400 -3.74861200 -0.09703000

N 0 -7.37372800 -2.74472300 1.55024000

H 0 -7.40153100 -2.46509400 2.52123600

C -1 -8.39055400 -2.20907000 0.65201400

H 0 -9.32247000 -2.09479100 1.20796300

H 0 -8.53775000 -2.92821900 -0.15785900

C 0 -8.00390100 -0.83169300 0.09829400

O 0 -8.46134000 0.20659600 0.57714900

N 0 -7.13790800 -0.87691600 -0.94354100

H 0 -6.69915600 -1.76357000 -1.16841000

C 0 -6.53416200 0.31224200 -1.49617700

H 0 -6.68131800 0.35791600 -2.57903400

H 0 -7.03072000 1.17772900 -1.04681300

C 0 -5.02843500 0.41241600 -1.23663500

O 0 -4.34784300 1.01897700 -2.08634300

O 0 -4.60570800 -0.13438400 -0.15473600

C -1 -3.44275500 3.56652500 5.04658200

H -1 -3.47198100 3.25859600 6.08134200

H -1 -4.26382400 4.20846500 4.76350000

H 0 -2.51083300 4.12845000 4.90307500

C 0 -3.52690200 2.42802400 4.02654400

H 0 -3.44550800 2.88180000 3.02967800

H 0 -4.51571000 1.95610700 4.05687200

C 0 -2.44316500 1.34913800 4.18009900

H 0 -2.81626400 0.52438700 4.79642500

H 0 -1.54910800 1.76502200 4.65539000

C 0 -2.05194300 0.84163300 2.80626000

O 0 -0.90799600 1.15569400 2.37674000

O 0 -2.92864700 0.15133700 2.18166800

C -1 -5.55850400 8.99860000 2.12536800

H -1 -5.41954700 8.57806000 3.11037600

H -1 -6.59539600 9.23074800 1.93218000

H 0 -5.01237500 9.94943900 2.09278400

C 0 -4.98778700 8.01559700 1.09577400

H 0 -3.89243100 8.02719300 1.18616300

H 0 -5.21412200 8.34360800 0.07261500

C 0 -5.52020700 6.59325600 1.31423600

H 0 -6.60807400 6.58540500 1.17029000

H 0 -5.33540300 6.28587900 2.35270700

C 0 -4.91689700 5.54179800 0.38311200

H 0 -5.11231400 5.80728000 -0.66667900

H 0 -5.38764900 4.57275200 0.57710000

N 0 -3.47991000 5.39492400 0.61659800

H 0 -2.94990900 6.23522500 0.80283500

C 0 -2.75816800 4.32572800 0.21204500

N 0 -3.34382200 3.32252100 -0.44043400

H 0 -2.88601800 2.39101600 -0.46102200

H 0 -4.21364800 3.43520200 -0.93737000

N 0 -1.45168900 4.26495900 0.47861400

H 0 -1.04625000 4.70276900 1.29918500

H 0 -0.85740900 3.50908800 0.06981900

C -1 -0.96061500 -1.40908700 7.52876900

H -1 0.02501100 -1.00277100 7.35594100

H -1 -1.13802400 -1.62947500 8.57103200

H 0 -1.71105900 -0.68830300 7.18719200

C 0 -1.10544400 -2.76220300 6.80582800

H 0 -2.07088900 -3.21374800 7.07251600

H 0 -0.33726800 -3.45244700 7.17969100

C 0 -1.02383600 -2.71130300 5.31421800

N 0 -1.11393900 -3.86401000 4.54123600

C 0 -0.93044800 -1.68416000 4.41478200

H 0 -0.79776900 -0.62979000 4.57611000

C 0 -1.09137800 -3.54908300 3.23922600

H 0 -1.15140500 -4.22744600 2.40245200

N 0 -0.99659100 -2.22685600 3.14443000

H 0 -1.01368900 -1.71225000 2.21762200

C -1 3.72997100 6.22396300 2.44655900

H -1 4.59452100 6.72938500 2.04218800

H -1 3.25896600 6.72870700 3.27709200

H 0 2.97174400 6.21547700 1.65311900

C 0 4.06737300 4.75048500 2.78951500

H 0 5.01881900 4.67149400 3.32536800

H 0 3.27314900 4.33947700 3.42317700

C 0 4.09962100 3.90474500 1.51243900

O 0 3.00000300 3.75717500 0.91497000

O 0 5.20973500 3.43155600 1.10034000

C -1 12.14574200 -2.28923200 -0.64637200

H -1 13.19003100 -2.01807300 -0.69466000

H -1 11.92892500 -3.17398400 -1.22654500

H 0 11.93490200 -2.56265500 0.39715300

C 0 11.23781100 -1.10870300 -1.06131400

H 0 11.68492800 -0.58216900 -1.91715400

H 0 10.26766200 -1.49293500 -1.39671600

C 0 10.99747200 -0.12119400 0.09529400

H 0 10.28742000 -0.57850100 0.79749200

H 0 11.93339200 0.02091100 0.65395300

C 0 10.50556100 1.27596800 -0.32066000

H 0 10.40826100 1.91200600 0.57007400

H 0 11.27173200 1.74613800 -0.95186000

C 0 9.19812300 1.34935400 -1.11087500

H 0 9.03075300 2.37463000 -1.45287500

H 0 9.21788100 0.70001000 -1.98983300

N 0 7.98985800 0.96267100 -0.31495500

H 0 7.13503300 1.32478000 -0.79020300

H 0 7.90375100 -0.07997100 -0.22327500

H 0 7.95784000 1.40224800 0.63457400

Cl 0 7.64401900 -2.13315200 -0.26267000

O 0 1.96673500 1.43156700 -3.55047400

H 0 3.40649500 2.37017700 -3.67142300

O 0 0.28691800 2.36393600 -0.30874300

O 0 0.63277400 3.73671900 2.50340700

H 0 0.20626800 2.86813500 2.39408300

H 0 1.36725000 3.72327000 1.84920100

O 0 -0.36999500 0.08803200 -4.54661400

H 0 0.36974000 0.71959000 -4.37247100

H 0 -0.09426200 -0.67581700 -3.98179000

O 0 -3.50268200 -0.95570600 -4.19558400

H 0 -4.15363800 -1.40590000 -3.61915900

H 0 -2.83368700 -1.65944800 -4.36719900

O 0 -4.58445700 -2.69478400 -2.21425500

H 0 -3.76908000 -2.31556300 -1.79325100

H 0 -5.04905300 -3.14157900 -1.48125600

O 0 2.34322300 3.65991600 -1.64983700

H 0 2.68889900 3.61130700 -0.71173300

H 0 3.33335400 -0.49990700 -1.92801800

O 0 0.39825100 -0.99839200 -2.21080100

O 0 5.87471300 2.52284700 -1.31920500

H 0 5.44306800 2.80494900 -0.45760400

H 0 6.35764700 3.31927400 -1.59715300

O 0 4.15276600 3.00836400 -3.48835000

H 0 4.71472600 2.55795700 -2.82205800

H 0 3.11317100 3.59244200 -2.28476400

O 0 7.42943600 2.27837100 2.10792500

H 0 6.62385600 2.79497800 1.83824500

H 0 7.10013100 1.64137300 2.76066400

O 0 3.01833300 -1.07164100 -2.65249400

H 0 2.37226000 0.54640400 -3.39474800

H 0 2.04642100 -1.14270200 -2.43293000

H 0 -1.18384100 -4.80759300 4.90163300

**^8'^L_P**

E (IEFPCM/B3LYP-D3/SDD,6-31G(d)) = -5854.297488 au

H (IEFPCM/B3LYP-D3/SDD,6-31G(d)) = -5852.745091 au

G (IEFPCM/B3LYP-D3/SDD,6-31G(d)) = -5852.986679 au

E (IEFPCM/B3LYP-D3/SDD,6-311+G(2d,p)//IEFPCM/B3LYP-D3/SDD,6-31G(d)) = -5856.120893 au

Mn 0 1.22057200 1.74779800 -2.05350200

Mn 0 -0.93495400 -2.28504300 -0.66841800

Mn 0 -0.31120800 0.76981500 0.25963600

Mn 0 -2.92810000 -0.41591200 0.37169900

O 0 -0.05129900 0.03405500 -1.74687400

O 0 -2.12293800 1.02296300 -0.32878800

O 0 -2.43512400 -1.51595800 -1.25573700

O 0 -0.87597300 -1.07172000 0.73279800

Ca 0 -2.22444400 0.33386600 -2.76079800

C -1 0.52523500 4.48976300 -5.74487500

H -1 0.59469400 5.43991400 -5.23604200

H -1 0.70602100 4.53306700 -6.80851400

H 0 1.35352600 3.89323800 -5.34087400

C 0 -0.78219600 3.74801600 -5.42036500

H 0 -0.97720000 2.97657000 -6.17765800

H 0 -1.65607600 4.41040900 -5.44571200

C 0 -0.80842900 2.98833400 -4.08408200

O 0 -1.87731200 2.39431900 -3.77336100

O 0 0.25910000 2.97561400 -3.39788300

C -1 -2.94142900 -6.75237400 -5.10937000

H -1 -1.98256500 -7.22148700 -5.27343400

H -1 -3.67971200 -6.91794600 -5.88003200

H 0 -3.35196200 -7.25090200 -4.21701100

C 0 -2.68412100 -5.28282500 -4.76242100

H 0 -3.61123500 -4.77355300 -4.47031800

H 0 -2.27635800 -4.73668400 -5.62023200

C 0 -1.68744200 -5.22903200 -3.60023700

H 0 -0.74974400 -5.72662600 -3.89219700

H 0 -2.07168100 -5.78229000 -2.73624700

C 0 -1.31212600 -3.83187300 -3.14191600

O 0 -1.33085900 -2.88285400 -3.94656800

O 0 -0.95824100 -3.78075100 -1.89949600

C -1 5.10178700 -4.51187100 1.08672700

H 0 5.91308600 -5.22505700 1.27661500

H 0 5.34927200 -3.97162700 0.16951800

C 0 5.00565900 -3.54983700 2.26883400

O 0 4.47516500 -3.90379700 3.34238900

C 0 3.77971400 -5.31688200 0.91828500

H 0 3.65732200 -5.95847000 1.79921500

H 0 3.86845900 -5.97044200 0.04607000

C 0 2.56878500 -4.44298400 0.76099100

N 0 2.30385400 -3.46786300 1.70888800

H 0 2.81906700 -3.37749500 2.58174800

C 0 1.59305600 -4.27192400 -0.19632800

H 0 1.41813300 -4.81216400 -1.11383000

C 0 1.26690500 -2.72670100 1.29980700

H 0 0.94315000 -1.82405300 1.77619100

N 0 0.78409100 -3.20176900 0.16209200

N 0 5.39203800 -2.28567200 2.03116800

H 0 5.93447900 -2.10252900 1.18125700

C -1 5.09028900 -1.17777300 2.93026600

H 0 5.96272400 -0.51180800 2.94303500

H 0 4.95976100 -1.58115900 3.93662900

C 0 3.83740200 -0.39429800 2.49931400

H 0 3.66210700 0.41611700 3.21765200

H 0 2.96879600 -1.05127300 2.56726800

C 0 3.94431800 0.17240300 1.07965500

H 0 4.45299300 -0.53510900 0.41424400

H 0 4.54849400 1.08813000 1.06410200

C 0 2.62549300 0.50126600 0.39380500

O 0 2.70604800 0.78716100 -0.83994700

O 0 1.54833600 0.43065300 1.05926400

C -1 -5.39201300 -3.82799800 2.38989100

H 0 -5.21134300 -3.03958000 3.12646400

H 0 -5.89773000 -4.65413000 2.90598500

C 0 -4.07166700 -4.33198000 1.79518400

H 0 -4.26057100 -5.10150400 1.04043400

H 0 -3.46128200 -4.80238000 2.57453400

C 0 -3.24135600 -3.23514700 1.13080900

O 0 -3.71575700 -2.06259200 1.19650300

O 0 -2.14110200 -3.56751800 0.59499400

C 0 -6.33342800 -3.32510700 1.29708100

O 0 -6.25952500 -3.72258500 0.12302900

N 0 -7.30848700 -2.48016000 1.69737000

H 0 -7.27629200 -2.09711700 2.63204100

C -1 -8.38001300 -2.04096500 0.80997200

H 0 -9.25024300 -1.79015900 1.41819500

H 0 -8.63057900 -2.87370900 0.14671600

C 0 -8.01398600 -0.79320500 -0.01144000

O 0 -8.43495000 0.32236000 0.29572600

N 0 -7.23787800 -1.04691600 -1.09450800

H 0 -6.79084100 -1.95659800 -1.14109500

C 0 -6.62735300 0.01661900 -1.86120700

H 0 -6.57409800 -0.26612500 -2.91556300

H 0 -7.26057600 0.90455900 -1.77868300

C 0 -5.20803600 0.37977200 -1.39549000

O 0 -4.47099400 0.95292700 -2.23684000

O 0 -4.89585000 0.07152400 -0.20224000

C -1 -3.34618700 3.87587000 4.90901100

H -1 -3.36688100 3.61167200 5.95599400

H -1 -4.16557500 4.51157100 4.60757200

H 0 -2.41667900 4.43603400 4.74079400

C 0 -3.41314900 2.69094100 3.95797100

H 0 -3.34736600 3.08363400 2.93434400

H 0 -4.38492500 2.18879300 4.02210200

C 0 -2.29245500 1.66794400 4.17967300

H 0 -2.61671400 0.90431500 4.89509900

H 0 -1.39099000 2.15241300 4.56774500

C 0 -1.97572500 1.04475000 2.83998900

O 0 -0.84526700 1.29723100 2.33208100

O 0 -2.89832900 0.34513100 2.31242100

C -1 -5.45352500 9.19688700 1.78439400

H -1 -5.30736100 8.81683700 2.78469600

H -1 -6.49069000 9.42854400 1.59208700

H 0 -4.89744000 10.13831900 1.69998100

C 0 -4.89769300 8.15006600 0.80387000

H 0 -3.80012500 8.17800500 0.85819900

H 0 -5.15971200 8.40321300 -0.23192300

C 0 -5.40266500 6.73800200 1.13618400

H 0 -6.49638800 6.71549200 1.04849200

H 0 -5.16548800 6.49788000 2.18184500

C 0 -4.84665100 5.62550200 0.24492700

H 0 -5.07019500 5.84135900 -0.81076200

H 0 -5.33703200 4.68144500 0.50462400

N 0 -3.40539500 5.44780700 0.43789500

H 0 -2.85273100 6.27946300 0.59775300

C 0 -2.72013000 4.36987300 -0.01287200

N 0 -3.33970300 3.37722200 -0.64643100

H 0 -2.90537700 2.42781300 -0.62655500

H 0 -4.28352300 3.46145300 -0.98994000

N 0 -1.39542600 4.30087700 0.17086500

H 0 -0.94557700 4.69727000 0.99225600

H 0 -0.85433800 3.51111600 -0.23856100

C -1 -0.87379900 -1.01019200 7.57222100

H -1 0.11283900 -0.61886700 7.37264300

H -1 -1.04196600 -1.18550100 8.62452400

H 0 -1.62153300 -0.29729100 7.20872800

C 0 -1.04003400 -2.39103900 6.90818800

H 0 -2.00564200 -2.82113100 7.20805100

H 0 -0.27325000 -3.07188100 7.30190900

C 0 -0.97900300 -2.40861000 5.41428000

N 0 -1.08873100 -3.59571300 4.69884800

C 0 -0.88976800 -1.42707800 4.46412400

H 0 -0.74285800 -0.36856800 4.57525400

C 0 -1.07963600 -3.33987100 3.38282400

H 0 -1.13499000 -4.06105500 2.58395200

N 0 -0.97869400 -2.02528200 3.22079900

H 0 -0.97475900 -1.55667800 2.24927600

C -1 3.81788600 6.36839800 2.12810300

H -1 4.68176300 6.84997500 1.69426700

H -1 3.35909200 6.91092600 2.94147200

H 0 3.05305800 6.32752900 1.34180300

C 0 4.13929800 4.91093800 2.54724400

H 0 5.08289400 4.85307900 3.09937600

H 0 3.33455800 4.54460900 3.19557900

C 0 4.18026500 3.97987100 1.33046400

O 0 3.09798200 3.80730200 0.70974100

O 0 5.29100900 3.44965100 0.99089400

C -1 12.14066000 -2.32956900 -0.69018200

H -1 13.18628700 -2.06851600 -0.76026600

H -1 11.91158500 -3.23613500 -1.23060600

H 0 11.94639800 -2.56576300 0.36607100

C 0 11.24063800 -1.15130600 -1.11879000

H 0 11.65728000 -0.67264100 -2.01698300

H 0 10.24644800 -1.52530900 -1.38927100

C 0 11.08621600 -0.11238200 0.00716400

H 0 10.40939500 -0.52466900 0.76846400

H 0 12.05483500 0.02860100 0.50725100

C 0 10.60174100 1.27800700 -0.43637500

H 0 10.55671800 1.94666800 0.43430100

H 0 11.34575700 1.71084900 -1.11863200

C 0 9.26129600 1.34330000 -1.16788900

H 0 9.07985000 2.36324000 -1.51874800

H 0 9.24161300 0.67987100 -2.03636700

N 0 8.08966100 0.96884500 -0.31561000

H 0 7.21286700 1.31174900 -0.77152200

H 0 8.00390200 -0.07104100 -0.20611100

H 0 8.08418000 1.43003000 0.62251000

Cl 0 7.60267600 -2.10744800 -0.32287000

O 0 1.97432000 0.89526100 -3.58632100

H 0 3.50189500 1.72944900 -3.75631900

O 0 0.27940100 2.28319300 -0.57959800

O 0 0.66153300 3.87279200 2.23227300

H 0 0.26397900 2.98250500 2.21139600

H 0 1.41694400 3.81930100 1.60502300

O 0 -0.53364200 -0.29604300 -4.50237700

H 0 0.31669800 0.13356000 -4.23968000

H 0 -0.46421000 -1.22722800 -4.19907500

O 0 -3.54584100 -1.26898400 -4.12072700

H 0 -4.16918600 -1.71365600 -3.50803000

H 0 -2.84913600 -1.94934200 -4.25845100

O 0 -4.53975900 -2.90778700 -2.05682700

H 0 -3.72940900 -2.47825700 -1.66447300

H 0 -5.04337300 -3.24660700 -1.29310700

O 0 2.58168400 3.45011100 -1.91102100

H 0 2.89277200 3.47809900 -0.96714900

H 0 3.18133600 -0.85964400 -1.75166400

O 0 0.20360100 -1.36496300 -1.80370000

O 0 5.88714900 2.32997700 -1.33708400

H 0 5.49602300 2.71281100 -0.49293600

H 0 6.26079300 3.10445000 -1.78960700

O 0 4.31161000 2.29854200 -3.64520100

H 0 4.78340200 1.92224700 -2.87480600

H 0 3.34786000 3.20948300 -2.49447800

O 0 7.51578900 2.35424200 2.07183400

H 0 6.71189100 2.85473700 1.77186400

H 0 7.17954600 1.73829600 2.74096600

O 0 2.87276700 -1.55828100 -2.35875500

H 0 2.31856600 0.01662000 -3.30967900

H 0 1.93105500 -1.65334300 -2.08887600

H 0 -1.15998100 -4.52061300 5.10427200

**^8^TS_2_**

E (IEFPCM/B3LYP-D3/SDD,6-31G(d)) = -5854.291157 au

H (IEFPCM/B3LYP-D3/SDD,6-31G(d)) = -5852.740910 au

G (IEFPCM/B3LYP-D3/SDD,6-31G(d)) = -5852.981334 au

E (IEFPCM/B3LYP-D3/SDD,6-311+G(2d,p)//IEFPCM/B3LYP-D3/SDD,6-31G(d)) = -5856.116736 au

Mn 0 1.26560500 1.86182000 -1.99261800

Mn 0 -0.87067900 -2.31483100 -0.69706200

Mn 0 -0.31498100 0.76788400 0.28397200

Mn 0 -2.89785800 -0.49283100 0.31081400

O 0 0.01105200 0.08299000 -1.82702000

O 0 -2.07900900 0.96002000 -0.41459500

O 0 -2.40735700 -1.52030300 -1.28721000

O 0 -0.82377300 -1.07024300 0.73148300

Ca 0 -2.29979000 0.34826600 -2.77560400

C -1 0.53808800 4.47206200 -5.74538400

H -1 0.61203500 5.42201000 -5.23680600

H -1 0.72027000 4.51402500 -6.80883800

H 0 1.37345500 3.87931600 -5.34818600

C 0 -0.75507800 3.72173300 -5.38744500

H 0 -0.93501500 2.91242100 -6.10842600

H 0 -1.64303100 4.36315700 -5.43646700

C 0 -0.76561500 3.01667000 -4.01925200

O 0 -1.83069500 2.45213200 -3.65798700

O 0 0.32172300 3.01472200 -3.35755200

C -1 -2.98894900 -6.75127600 -5.10976400

H -1 -2.03241200 -7.22553400 -5.27260800

H -1 -3.72725400 -6.91319800 -5.88117900

H 0 -3.40612400 -7.23312300 -4.21176700

C 0 -2.73456200 -5.27107200 -4.79830000

H 0 -3.67515700 -4.72956600 -4.63626600

H 0 -2.22547800 -4.77827500 -5.63436800

C 0 -1.87620000 -5.16961700 -3.53832100

H 0 -0.99814300 -5.82837700 -3.61847600

H 0 -2.43361000 -5.52634000 -2.66413400

C 0 -1.34275100 -3.78997800 -3.19970000

O 0 -1.39219500 -2.85946100 -4.02525200

O 0 -0.81581600 -3.72977500 -2.01996900

C -1 5.05924300 -4.55133000 1.09440000

H 0 5.88994800 -5.24586200 1.27110900

H 0 5.28304200 -4.00258400 0.17589000

C 0 4.96379700 -3.59008200 2.27828200

O 0 4.43376700 -3.94247600 3.35274000

C 0 3.75359800 -5.37895300 0.93810200

H 0 3.63328700 -6.00574600 1.82955200

H 0 3.85029400 -6.04427900 0.07587800

C 0 2.54882200 -4.50418900 0.76308800

N 0 2.26270900 -3.54930300 1.72650100

H 0 2.76184100 -3.47741400 2.61117000

C 0 1.64015100 -4.27038400 -0.24309000

H 0 1.49931700 -4.77734900 -1.18493100

C 0 1.28231500 -2.75713300 1.28107400

H 0 0.94983400 -1.86410900 1.76836600

N 0 0.85098600 -3.18123300 0.10172200

N 0 5.35698500 -2.32814500 2.04039600

H 0 5.91145600 -2.15036500 1.19688700

C -1 5.06343100 -1.21656200 2.93675800

H 0 5.94265300 -0.56078200 2.95651600

H 0 4.91677900 -1.61672700 3.94240500

C 0 3.81991000 -0.42046300 2.49354300

H 0 3.65391600 0.40151800 3.20099600

H 0 2.94490400 -1.06878300 2.56896100

C 0 3.92617800 0.12618100 1.06543600

H 0 4.34590500 -0.63433300 0.39529600

H 0 4.60015600 0.99006300 1.01533900

C 0 2.61192200 0.53944900 0.41744400

O 0 2.69295300 0.91458700 -0.79692300

O 0 1.53760000 0.42602100 1.07219000

C -1 -5.43220200 -3.81125900 2.38577200

H 0 -5.22349400 -3.01043000 3.10160400

H 0 -5.96376300 -4.60543400 2.92613500

C 0 -4.13323800 -4.36643600 1.80111100

H 0 -4.34548200 -5.14496500 1.06199900

H 0 -3.53629100 -4.83970000 2.58968700

C 0 -3.26149200 -3.31353000 1.11845800

O 0 -3.69744200 -2.11751000 1.16330400

O 0 -2.17559000 -3.69007600 0.60148500

C 0 -6.36510600 -3.30087200 1.28959800

O 0 -6.28901800 -3.69309400 0.11365900

N 0 -7.33956700 -2.45424300 1.68970100

H 0 -7.30990200 -2.07689500 2.62675000

C -1 -8.40892800 -2.00893900 0.80194100

H 0 -9.26858200 -1.72933000 1.41242000

H 0 -8.68230100 -2.84705600 0.15428100

C 0 -8.02212700 -0.78236700 -0.04437100

O 0 -8.41667500 0.34734800 0.24534100

N 0 -7.26255800 -1.07193800 -1.13061600

H 0 -6.82752700 -1.98856300 -1.15196700

C 0 -6.63430000 -0.03319500 -1.91713200

H 0 -6.53374600 -0.36551300 -2.95374300

H 0 -7.28019500 0.84906500 -1.90705400

C 0 -5.23762400 0.37380300 -1.41687500

O 0 -4.51718600 1.00718500 -2.23656600

O 0 -4.91793300 0.03698300 -0.23853800

C -1 -3.34824700 3.88252900 4.90444900

H -1 -3.37149500 3.61881700 5.95150200

H -1 -4.16391400 4.52246600 4.60188600

H 0 -2.40896800 4.41957100 4.72091500

C 0 -3.46921000 2.69222100 3.95579700

H 0 -3.42138300 3.08349800 2.93075300

H 0 -4.45743500 2.22685500 4.04677000

C 0 -2.38183300 1.62306400 4.14100300

H 0 -2.73854800 0.83011900 4.80685200

H 0 -1.47551400 2.06078400 4.57120200

C 0 -2.04598200 1.05523000 2.77883600

O 0 -0.91078500 1.34543200 2.29785000

O 0 -2.95040600 0.36693600 2.20803900

C -1 -5.42385100 9.21355400 1.77565400

H -1 -5.28080700 8.83309000 2.77624900

H -1 -6.45955900 9.45065000 1.58212400

H 0 -4.85596400 10.14655200 1.68216100

C 0 -4.88935900 8.14475000 0.80543500

H 0 -3.79097400 8.16814700 0.83989900

H 0 -5.16975800 8.37518900 -0.23079300

C 0 -5.39636300 6.74428200 1.18357700

H 0 -6.49179200 6.72639200 1.11795300

H 0 -5.13984500 6.53259400 2.23077700

C 0 -4.86330000 5.60166400 0.31690000

H 0 -5.12190300 5.77240800 -0.73899400

H 0 -5.33710300 4.66547700 0.62967400

N 0 -3.41587200 5.44345900 0.47039800

H 0 -2.86573400 6.28976100 0.53146200

C 0 -2.73494200 4.34497900 0.06418100

N 0 -3.36891500 3.29152700 -0.43963900

H 0 -2.87982800 2.36700000 -0.48569200

H 0 -4.30736700 3.34523500 -0.80497400

N 0 -1.39914500 4.32373400 0.17855900

H 0 -0.92969500 4.76819400 0.96311400

H 0 -0.85641000 3.52237300 -0.20462300

C -1 -0.90477900 -1.01565000 7.57208700

H -1 0.08414300 -0.62964400 7.37345900

H -1 -1.07503100 -1.18969100 8.62426600

H 0 -1.64525300 -0.29257500 7.21285200

C 0 -1.08308700 -2.38482500 6.89106100

H 0 -2.05473700 -2.80901600 7.17965500

H 0 -0.32552900 -3.07931200 7.27913600

C 0 -1.01243000 -2.38385400 5.39648100

N 0 -1.14504200 -3.56377600 4.67379000

C 0 -0.88552400 -1.40030300 4.45112200

H 0 -0.71925900 -0.34440800 4.56732700

C 0 -1.11219700 -3.30213200 3.35928700

H 0 -1.18657600 -4.01821700 2.55677400

N 0 -0.97379900 -1.99050200 3.20262500

H 0 -0.94563500 -1.52271100 2.21843100

C -1 3.83202000 6.33597600 2.13055600

H -1 4.69891800 6.81280200 1.69750500

H -1 3.37522100 6.88122200 2.94323100

H 0 3.06818700 6.27906000 1.34486200

C 0 4.16598300 4.88829200 2.57803500

H 0 5.10907500 4.85258500 3.13286000

H 0 3.36306900 4.52770100 3.23153500

C 0 4.21784700 3.93825100 1.37867400

O 0 3.13015300 3.72594100 0.77830300

O 0 5.33634700 3.43612800 1.02571200

C -1 12.11155900 -2.40708200 -0.67552400

H -1 13.15863400 -2.15161300 -0.74454600

H -1 11.87826500 -3.31261100 -1.21588300

H 0 11.90517100 -2.63157200 0.38050200

C 0 11.22310200 -1.23090600 -1.13223400

H 0 11.63700300 -0.79000200 -2.05094600

H 0 10.22188000 -1.60167600 -1.37975200

C 0 11.09255300 -0.15094600 -0.04397300

H 0 10.43103200 -0.53226800 0.74629900

H 0 12.07110900 0.00918800 0.43038300

C 0 10.59825600 1.22071000 -0.53285900

H 0 10.56207500 1.92147600 0.31262700

H 0 11.33195300 1.62952400 -1.24059200

C 0 9.24845600 1.25625800 -1.24955000

H 0 9.05964700 2.26353500 -1.63160700

H 0 9.21913400 0.56450300 -2.09520600

N 0 8.08957600 0.90876900 -0.36823400

H 0 7.20938200 1.25380500 -0.81015700

H 0 7.99811000 -0.12818400 -0.23193900

H 0 8.10956500 1.39234100 0.55901600

Cl 0 7.61679000 -2.16938900 -0.25739900

O 0 2.04912900 1.00588800 -3.52499500

H 0 3.52912800 1.90959500 -3.72658800

O 0 0.28081500 2.30781000 -0.53252900

O 0 0.67542200 3.90444500 2.23544600

H 0 0.24467500 3.03122800 2.19438400

H 0 1.44404100 3.82591200 1.62669100

O 0 -0.60101100 -0.24496400 -4.53049500

H 0 0.24145200 0.17081900 -4.24054900

H 0 -0.53197700 -1.18736800 -4.25751100

O 0 -3.62728500 -1.27153400 -4.11053300

H 0 -4.21269700 -1.74062900 -3.47573500

H 0 -2.92845900 -1.93982700 -4.29185500

O 0 -4.49683500 -2.95081100 -2.04872400

H 0 -3.69052800 -2.50045100 -1.66710200

H 0 -5.02369200 -3.22553700 -1.27528300

O 0 2.58894400 3.59534400 -1.83408600

H 0 2.92634300 3.56707300 -0.89822800

H 0 3.33407600 -0.66092800 -1.68346100

O 0 0.36083600 -1.21903500 -1.99301500

O 0 5.92951400 2.39353600 -1.33941600

H 0 5.53520400 2.71974900 -0.47304500

H 0 6.37579800 3.18117100 -1.69280000

O 0 4.30424300 2.52697300 -3.62814300

H 0 4.82473000 2.16152600 -2.88254900

H 0 3.33702500 3.38467800 -2.45445600

O 0 7.60325700 2.32593300 2.01440500

H 0 6.78729600 2.82817100 1.75541500

H 0 7.30275200 1.71416800 2.70398600

O 0 3.13970900 -1.56060000 -2.00381800

H 0 2.37699400 0.12490000 -3.26457700

H 0 2.18947800 -1.65375000 -1.78844700

H 0 -1.24743200 -4.48779200 5.07420300

**^8^L_S**

E (IEFPCM/B3LYP-D3/SDD,6-31G(d)) = -5854.296311 au

H (IEFPCM/B3LYP-D3/SDD,6-31G(d)) = -5852.743554 au

G (IEFPCM/B3LYP-D3/SDD,6-31G(d)) = -5852.985832 au

E (IEFPCM/B3LYP-D3/SDD,6-311+G(2d,p)//IEFPCM/B3LYP-D3/SDD,6-31G(d)) = -5856.121692 au

Mn 0 1.26187800 1.87881000 -1.98974700

Mn 0 -0.85518200 -2.34337000 -0.69531600

Mn 0 -0.31046300 0.76094300 0.29289300

Mn 0 -2.89696000 -0.50302800 0.31622100

O 0 0.03316500 0.10472200 -1.85821300

O 0 -2.07394400 0.95380600 -0.40141000

O 0 -2.39139100 -1.52972300 -1.28455800

O 0 -0.81206300 -1.07966700 0.73473700

Ca 0 -2.30035600 0.36656500 -2.78168900

C -1 0.53703100 4.47521300 -5.73218500

H -1 0.61099600 5.42451200 -5.22240000

H -1 0.71986100 4.51841700 -6.79547800

H 0 1.37089700 3.88200500 -5.33372700

C 0 -0.76017000 3.73224700 -5.38113500

H 0 -0.94110500 2.92267500 -6.10131000

H 0 -1.64374800 4.37928800 -5.43766600

C 0 -0.78583500 3.03052000 -4.01254800

O 0 -1.85198200 2.44944300 -3.68078700

O 0 0.28308500 3.04897800 -3.32174500

C -1 -2.99417000 -6.74770100 -5.11240400

H -1 -2.03769700 -7.22208200 -5.27526000

H -1 -3.73207100 -6.90842800 -5.88445600

H 0 -3.40844300 -7.22551100 -4.21130500

C 0 -2.72229800 -5.27034400 -4.80397700

H 0 -3.64511600 -4.74129200 -4.53553500

H 0 -2.30316500 -4.75720200 -5.67701200

C 0 -1.73565100 -5.19099600 -3.63870600

H 0 -0.81977100 -5.75062900 -3.88447600

H 0 -2.15182200 -5.67467700 -2.74815200

C 0 -1.28746900 -3.79730100 -3.24026700

O 0 -1.35806000 -2.84897500 -4.04490500

O 0 -0.80273000 -3.74571600 -2.04352700

C -1 5.05106900 -4.55807300 1.09923400

H 0 5.89535600 -5.23932800 1.26497300

H 0 5.25529400 -4.00941300 0.17616100

C 0 4.96246900 -3.59866800 2.28516800

O 0 4.44708200 -3.95661800 3.36461600

C 0 3.75825200 -5.40475300 0.96150500

H 0 3.64964300 -6.02510300 1.85872900

H 0 3.85536600 -6.07490300 0.10312600

C 0 2.55252200 -4.53548800 0.78546300

N 0 2.26546200 -3.57751300 1.74567700

H 0 2.75908600 -3.50669900 2.63333400

C 0 1.65410700 -4.29918900 -0.22807000

H 0 1.51558300 -4.80780000 -1.16938500

C 0 1.29331400 -2.78053100 1.29116300

H 0 0.95711300 -1.88558400 1.77259600

N 0 0.87047100 -3.20464700 0.10864500

N 0 5.35037100 -2.33503100 2.04684300

H 0 5.88189300 -2.15049400 1.19026500

C -1 5.05528700 -1.22556600 2.94567600

H 0 5.93675300 -0.57283900 2.97811300

H 0 4.89613500 -1.63038100 3.94701100

C 0 3.81741400 -0.42423900 2.49157800

H 0 3.64115500 0.38948400 3.20600100

H 0 2.94293500 -1.07466900 2.54999600

C 0 3.93443500 0.14448400 1.07005500

H 0 4.39841900 -0.58676400 0.39658000

H 0 4.57908100 1.03181000 1.04657500

C 0 2.61611900 0.53265300 0.40903800

O 0 2.69144200 0.89235500 -0.81183000

O 0 1.54483000 0.42402700 1.07095200

C -1 -5.44089200 -3.81603400 2.38527200

H 0 -5.22455800 -3.01188300 3.09530900

H 0 -5.98504600 -4.59772500 2.93180700

C 0 -4.15207200 -4.38937800 1.81026000

H 0 -4.36970100 -5.16996300 1.07493900

H 0 -3.56098000 -4.86295000 2.60311300

C 0 -3.26861300 -3.35086500 1.12611000

O 0 -3.68793900 -2.14688900 1.15874100

O 0 -2.18824300 -3.75396800 0.62361400

C 0 -6.36900300 -3.30137800 1.28647700

O 0 -6.29012700 -3.68899000 0.10933700

N 0 -7.34523200 -2.45640700 1.68780700

H 0 -7.31718600 -2.08277300 2.62638800

C -1 -8.41606600 -2.01077100 0.80187800

H 0 -9.27698200 -1.73663100 1.41309000

H 0 -8.68646900 -2.84704500 0.15055400

C 0 -8.03193900 -0.78073900 -0.03903300

O 0 -8.43360100 0.34652100 0.25069700

N 0 -7.26390600 -1.06507300 -1.12068400

H 0 -6.82651600 -1.98081200 -1.14154800

C 0 -6.63451800 -0.02211400 -1.89995000

H 0 -6.54296300 -0.34134800 -2.94146900

H 0 -7.27511400 0.86374200 -1.87369000

C 0 -5.23316500 0.37044700 -1.40313600

O 0 -4.51059400 0.99975500 -2.22384800

O 0 -4.91251100 0.02996700 -0.22599700

C -1 -3.35584000 3.87395900 4.91460500

H -1 -3.37979900 3.60897300 5.96132100

H -1 -4.17111100 4.51454200 4.61234100

H 0 -2.41766700 4.41524700 4.73691900

C 0 -3.46522300 2.68770000 3.95858300

H 0 -3.40397100 3.08554300 2.93664500

H 0 -4.45388500 2.22053300 4.03439200

C 0 -2.37894500 1.61869100 4.15057500

H 0 -2.73461100 0.83222600 4.82471600

H 0 -1.47188800 2.06047000 4.57511500

C 0 -2.04287200 1.03801700 2.79308500

O 0 -0.90913200 1.33128700 2.30940800

O 0 -2.94060000 0.33477200 2.23057500

C -1 -5.42778200 9.20951200 1.79110600

H -1 -5.28546200 8.82777500 2.79131900

H -1 -6.46329500 9.44719400 1.59725000

H 0 -4.86078600 10.14340200 1.70038800

C 0 -4.88764500 8.14484100 0.81829100

H 0 -3.78961400 8.16331500 0.86625100

H 0 -5.15401300 8.38655300 -0.21908700

C 0 -5.40382100 6.74155300 1.17497100

H 0 -6.49812900 6.72746200 1.09242400

H 0 -5.16400000 6.51836100 2.22374900

C 0 -4.86177200 5.60495200 0.30402700

H 0 -5.10100300 5.79042100 -0.75390600

H 0 -5.34705300 4.66813700 0.59699700

N 0 -3.41796300 5.43446600 0.48021500

H 0 -2.86462300 6.27593900 0.57193400

C 0 -2.73321700 4.34005500 0.06848600

N 0 -3.35664500 3.29992200 -0.47617600

H 0 -2.87588000 2.37113100 -0.50698900

H 0 -4.29304100 3.35849400 -0.84570900

N 0 -1.40156600 4.30720800 0.21541400

H 0 -0.94388100 4.74308200 1.01141000

H 0 -0.85326500 3.51278800 -0.17511300

C -1 -0.91561300 -1.02830900 7.57769800

H -1 0.07355700 -0.64239400 7.38013000

H -1 -1.08655000 -1.20357900 8.62956100

H 0 -1.65440500 -0.30272300 7.21984300

C 0 -1.09663500 -2.39427100 6.89120300

H 0 -2.07195800 -2.81413300 7.17372600

H 0 -0.34482000 -3.09402100 7.28106100

C 0 -1.01817600 -2.39017400 5.39655500

N 0 -1.15594100 -3.56844800 4.67195200

C 0 -0.88014200 -1.40682200 4.45230500

H 0 -0.70669100 -0.35222900 4.57045000

C 0 -1.11530200 -3.30552400 3.35767800

H 0 -1.19596500 -4.01959700 2.55362500

N 0 -0.96613800 -1.99505800 3.20236600

H 0 -0.93409000 -1.52664800 2.21457900

C -1 3.82690500 6.32837000 2.14799000

H -1 4.69422200 6.80543400 1.71603800

H -1 3.36980600 6.87277600 2.96106000

H 0 3.06334300 6.27251900 1.36235800

C 0 4.16202700 4.88062000 2.59387200

H 0 5.10822300 4.84287000 3.14321300

H 0 3.36263200 4.51914900 3.25124900

C 0 4.20470700 3.93525700 1.39159100

O 0 3.11423100 3.73991500 0.79092200

O 0 5.31673000 3.42079600 1.03549600

C -1 12.10516000 -2.41404500 -0.66386800

H -1 13.15236300 -2.15884600 -0.73195500

H -1 11.87188300 -3.31883400 -1.20547300

H 0 11.90495500 -2.64685600 0.39191900

C 0 11.21269600 -1.23313300 -1.10193600

H 0 11.63729000 -0.76050200 -1.99961700

H 0 10.21887300 -1.60433400 -1.37818300

C 0 11.05345800 -0.18741300 0.01687800

H 0 10.36529400 -0.59042900 0.77290800

H 0 12.01747200 -0.05006800 0.52673400

C 0 10.58422200 1.20361400 -0.44119300

H 0 10.53431700 1.87882400 0.42417000

H 0 11.33919600 1.62581000 -1.11801900

C 0 9.25230000 1.27488900 -1.18805300

H 0 9.08746300 2.29232800 -1.55393800

H 0 9.23262700 0.60028800 -2.04781900

N 0 8.06839900 0.92751000 -0.34056200

H 0 7.20178200 1.28928300 -0.79764300

H 0 7.96022000 -0.11059000 -0.22834100

H 0 8.07429800 1.39253200 0.59650300

Cl 0 7.53628100 -2.13759500 -0.33074200

O 0 2.05078600 1.07575400 -3.53732200

H 0 3.50939000 1.99783700 -3.73976000

O 0 0.28524300 2.30538000 -0.51755200

O 0 0.66691900 3.88315500 2.27384800

H 0 0.23759100 3.00930400 2.22732200

H 0 1.42931800 3.81346500 1.65696400

O 0 -0.60575400 -0.21872900 -4.54904100

H 0 0.23432500 0.20589100 -4.26578500

H 0 -0.52253900 -1.15890500 -4.27287400

O 0 -3.60542300 -1.26720700 -4.10873900

H 0 -4.18849400 -1.74008800 -3.47407000

H 0 -2.89925500 -1.92907000 -4.28615500

O 0 -4.48343800 -2.94164700 -2.05501200

H 0 -3.67012000 -2.50049300 -1.67336800

H 0 -5.00941200 -3.21583100 -1.28096200

O 0 2.56906900 3.60475000 -1.80961400

H 0 2.90661700 3.57652300 -0.87344000

H 0 3.40570200 -0.64190200 -1.76324500

O 0 0.42929300 -1.16540100 -2.07015100

O 0 5.91063300 2.39608600 -1.34009600

H 0 5.51324600 2.73336900 -0.47972700

H 0 6.35067300 3.18098600 -1.70746700

O 0 4.29286200 2.60355600 -3.62409800

H 0 4.80767100 2.20549100 -2.89179000

H 0 3.32175500 3.41931900 -2.43254900

O 0 7.58990000 2.34829700 2.04168400

H 0 6.77020700 2.84075800 1.77411400

H 0 7.29316100 1.74775500 2.74277200

O 0 3.17076700 -1.39194500 -2.34059600

H 0 2.43424900 0.20676800 -3.29564400

H 0 2.23572600 -1.54385500 -2.09299800

H 0 -1.26783900 -4.49197700 5.07083700

**^10^L_F**

E (IEFPCM/B3LYP-D3/SDD,6-31G(d)) = -5703.972069 au

H (IEFPCM/B3LYP-D3/SDD,6-31G(d)) = -5702.430449 au

G (IEFPCM/B3LYP-D3/SDD,6-31G(d)) = -5702.670251 au

E (IEFPCM/B3LYP-D3/SDD,6-311+G(2d,p)//IEFPCM/B3LYP-D3/SDD,6-31G(d)) = -5705.746756 au

Mn 0 1.21838400 2.17844900 -1.94884200

Mn 0 -0.87439700 -2.34307700 -0.75643300

Mn 0 -0.30689300 0.73371500 0.42845400

Mn 0 -2.87644400 -0.57312300 0.28702400

O 0 -2.04597500 0.91506500 -0.36270800

O 0 -2.35781900 -1.47965400 -1.37259800

O 0 -0.81221800 -1.17746500 0.69521000

Ca 0 -2.50281200 0.63840100 -2.69664100

C -1 0.43843600 4.68400600 -5.59894900

H -1 0.50090900 5.61724000 -5.05885300

H -1 0.60516700 4.76568300 -6.66249000

H 0 1.26766100 4.07692300 -5.21367700

C 0 -0.86973100 3.94082900 -5.28442400

H 0 -0.98622200 3.07329700 -5.94785500

H 0 -1.75410300 4.56726400 -5.45920100

C 0 -0.95261000 3.38076200 -3.85622400

O 0 -2.00277200 2.74241200 -3.53317800

O 0 0.03919500 3.56785100 -3.09521800

C -1 -2.85835300 -6.62312200 -5.30214400

H -1 -1.89506100 -7.07228400 -5.49376700

H -1 -3.60397300 -6.77351700 -6.06886900

H 0 -3.25169300 -7.13060000 -4.40787000

C 0 -2.63713700 -5.13124800 -4.97315800

H 0 -3.59392200 -4.62558900 -4.79296600

H 0 -2.17244700 -4.62294100 -5.82589700

C 0 -1.74420200 -4.95107300 -3.73604500

H 0 -0.85253100 -5.59211600 -3.80586300

H 0 -2.26346500 -5.26791200 -2.82272200

C 0 -1.22436200 -3.53956800 -3.49460600

O 0 -1.40604500 -2.63753300 -4.32819900

O 0 -0.52876800 -3.38729900 -2.40002800

C -1 5.23034300 -4.47444500 0.86720700

H 0 6.05073800 -5.18105300 1.04300200

H 0 5.48785100 -3.89784800 -0.02434800

C 0 5.10840800 -3.55375000 2.08127400

O 0 4.54404700 -3.93659200 3.12843300

C 0 3.92803300 -5.29476700 0.63855400

H 0 3.79650800 -5.97628900 1.48744400

H 0 4.04722700 -5.90745200 -0.25918000

C 0 2.70684000 -4.43413700 0.49670900

N 0 2.38736300 -3.54770700 1.51306200

H 0 2.89376500 -3.50696600 2.39743400

C 0 1.77365400 -4.19206400 -0.48509500

H 0 1.64485300 -4.65607300 -1.45009800

C 0 1.36284800 -2.78408700 1.12674200

H 0 0.99627300 -1.93701300 1.66809700

N 0 0.93668400 -3.15799700 -0.07459900

N 0 5.52647100 -2.29122400 1.90418900

H 0 6.08906000 -2.08823800 1.07233800

C -1 5.19459300 -1.20485200 2.82268700

H 0 6.02021100 -0.48350800 2.78423700

H 0 5.14339000 -1.61041500 3.83587600

C 0 3.87524000 -0.51044100 2.45377200

H 0 3.69731300 0.30972300 3.15905900

H 0 3.05472700 -1.21639000 2.59957700

C 0 3.86663600 0.01797000 1.01389900

H 0 4.39093700 -0.66066600 0.33384000

H 0 4.39534600 0.97761500 0.95927700

C 0 2.48900600 0.23950500 0.41542600

O 0 2.37613900 0.28699100 -0.83959500

O 0 1.50430500 0.36192000 1.22125200

C -1 -5.25465100 -3.98960800 2.32138100

H 0 -5.01963400 -3.19779000 3.03874000

H 0 -5.77455000 -4.78745100 2.86741000

C 0 -3.98125900 -4.54130300 1.68443700

H 0 -4.21896700 -5.32187700 0.95551800

H 0 -3.34656000 -5.00827500 2.44645000

C 0 -3.16131300 -3.47392700 0.96616000

O 0 -3.60450700 -2.28611000 1.05162800

O 0 -2.10255000 -3.83495800 0.37733900

C 0 -6.21448600 -3.45923800 1.25467700

O 0 -6.15578500 -3.81871500 0.06735800

N 0 -7.18905200 -2.63231000 1.69368400

H 0 -7.15092700 -2.28192200 2.64087300

C -1 -8.28796500 -2.19462100 0.83968200

H 0 -9.14417500 -1.96453900 1.47588600

H 0 -8.54773800 -3.02299800 0.17433100

C 0 -7.96831600 -0.93489400 0.02367800

O 0 -8.35566000 0.17842400 0.37698500

N 0 -7.27599800 -1.17486300 -1.11943400

H 0 -6.81309500 -2.07535900 -1.18718800

C 0 -6.70084800 -0.08583300 -1.87448900

H 0 -6.57656200 -0.38469600 -2.91903900

H 0 -7.38899400 0.76307400 -1.84967100

C 0 -5.33243800 0.37801600 -1.35572700

O 0 -4.70641800 1.18527100 -2.10180200

O 0 -4.92293100 -0.08829500 -0.25542500

C -1 -3.28715500 3.65466600 5.07490200

H -1 -3.29119800 3.35450800 6.11240300

H -1 -4.11979700 4.28745900 4.80520000

H 0 -2.34907100 4.18417800 4.87366000

C 0 -3.48201700 2.48808300 4.09392800

H 0 -3.39746400 2.89997600 3.07958500

H 0 -4.50826900 2.10930600 4.16898000

C 0 -2.50098200 1.31301400 4.24677600

H 0 -2.94622600 0.50946900 4.84332100

H 0 -1.58152500 1.64595700 4.73835400

C 0 -2.14333900 0.81015500 2.86173100

O 0 -0.98862500 1.09446100 2.44157700

O 0 -3.04365200 0.17957600 2.21279500

C -1 -5.51040800 9.04603600 2.15840200

H -1 -5.34620600 8.63456200 3.14339400

H -1 -6.55326700 9.26851600 1.98680200

H 0 -4.97268300 10.00115000 2.11008500

C 0 -4.95962600 8.07142600 1.10286500

H 0 -3.86240400 8.07436000 1.16514900

H 0 -5.21560400 8.42230400 0.09349800

C 0 -5.48076600 6.64128200 1.30102900

H 0 -6.57912800 6.65126400 1.29686200

H 0 -5.17014600 6.27143000 2.28762800

C 0 -5.03211700 5.63459500 0.23378800

H 0 -5.40143000 5.93981400 -0.75518200

H 0 -5.46712100 4.65921500 0.46326900

N 0 -3.57835800 5.47577300 0.19268000

H 0 -3.02303800 6.31533700 0.10138800

C 0 -2.91508200 4.30718700 0.04763700

N 0 -3.57928400 3.15374200 -0.12424200

H 0 -3.00878200 2.28509500 -0.17182500

H 0 -4.42725100 3.12661400 -0.67441800

N 0 -1.58791700 4.28011400 0.12408100

H 0 -1.04491200 5.01850900 0.55551900

H 0 -1.03169200 3.42504200 -0.11732300

C -1 -0.71151800 -1.28174700 7.54032500

H -1 0.26673100 -0.86948200 7.34223400

H -1 -0.86388600 -1.49494300 8.58815500

H 0 -1.46946400 -0.55997700 7.21680800

C 0 -0.88063400 -2.63134400 6.81604300

H 0 -1.83466600 -3.08457600 7.11911700

H 0 -0.09857100 -3.32246200 7.15886200

C 0 -0.85593500 -2.58475900 5.31974300

N 0 -0.99292000 -3.74552900 4.56576200

C 0 -0.77189400 -1.57134900 4.40125400

H 0 -0.62172200 -0.51560700 4.53967500

C 0 -1.00259600 -3.44388400 3.25890500

H 0 -1.08041000 -4.13724200 2.43669400

N 0 -0.88593600 -2.12690600 3.13922100

H 0 -0.88760400 -1.63872500 2.16484700

C -1 3.80344800 6.34420800 2.29228500

H -1 4.65542600 6.85302800 1.86452200

H -1 3.34841100 6.85268000 3.12928800

H 0 3.03790300 6.35246000 1.50894000

C 0 4.16796200 4.87455200 2.57620400

H 0 5.02731400 4.77974800 3.24794600

H 0 3.31485900 4.37177400 3.05165600

C 0 4.44235300 4.09514100 1.28094100

O 0 3.69313100 4.34443700 0.29778300

O 0 5.37237300 3.22529900 1.25409600

C -1 12.21392500 -2.12990700 -0.92088800

H -1 13.25476900 -1.85123500 -0.99495900

H -1 11.99108500 -3.02101600 -1.48892100

H 0 12.03588100 -2.40377500 0.12905700

C 0 11.29007400 -0.95448400 -1.30029100

H 0 11.67905100 -0.44691700 -2.19504900

H 0 10.29653500 -1.33890000 -1.55726200

C 0 11.14722500 0.05260700 -0.14562400

H 0 10.52869200 -0.40597600 0.63860700

H 0 12.13239800 0.22673400 0.30970500

C 0 10.57495600 1.42666900 -0.53135100

H 0 10.53871900 2.07095000 0.35784400

H 0 11.26018300 1.91348100 -1.23825500

C 0 9.19615400 1.43428400 -1.19102700

H 0 8.92675200 2.45399600 -1.48073800

H 0 9.17394300 0.81206000 -2.08948400

N 0 8.10410700 0.93820200 -0.29770600

H 0 7.17680000 1.22892100 -0.69189600

H 0 8.09875000 -0.10786100 -0.23279700

H 0 8.12720500 1.35490000 0.66104600

Cl 0 7.78253500 -2.16066900 -0.41925100

O 0 1.57587000 0.95926500 -3.56539200

H 0 3.25277100 1.32239400 -3.71096000

O 0 0.21359500 2.29380000 -0.23721300

O 0 1.06206400 4.34972900 1.29275600

H 0 0.78959900 3.51050300 0.84102700

H 0 1.95919700 4.49370200 0.92795300

O 0 -0.78513100 0.02992200 -4.13531400

H 0 0.14151800 0.43812100 -4.01856600

H 0 -0.70138100 -0.94631600 -4.20109700

O 0 -3.72577700 -1.05006800 -4.05028300

H 0 -4.24819200 -1.62443000 -3.44640000

H 0 -2.99822700 -1.64455400 -4.33411000

O 0 -4.43363900 -2.95677900 -2.13949200

H 0 -3.63543900 -2.49368600 -1.75977500

H 0 -4.94768700 -3.24604300 -1.36225000

O 0 2.96264400 3.47204800 -2.09736600

H 0 3.37896700 3.68898900 -1.22100800

H 0 2.12667100 -1.33145300 -1.87988900

O 0 5.71235100 2.05870500 -1.13298700

H 0 5.49981800 2.54060100 -0.27782600

H 0 5.88831300 2.75802100 -1.78240900

O 0 4.20673100 1.59790100 -3.56638700

H 0 4.46540100 1.13861400 -2.74698000

H 0 3.60108300 2.92444700 -2.62748200

O 0 7.68723600 2.18952800 2.19601300

H 0 6.83525400 2.65380100 1.99131800

H 0 7.46342000 1.55817800 2.89680400

O 0 2.01714200 -1.77610800 -2.74379000

H 0 1.79305600 0.04398500 -3.29601500

H 0 1.20118400 -2.30397900 -2.66578900

H 0 -1.06851700 -4.68234500 4.94179300

**^8′′^L_S_4_^*^**

E (IEFPCM/B3LYP-D3/SDD,6-31G(d)) = -5854.303945 au

H (IEFPCM/B3LYP-D3/SDD,6-31G(d)) = -5852.750187 au

G (IEFPCM/B3LYP-D3/SDD,6-31G(d)) = -5852.988559 au

E (IEFPCM/B3LYP-D3/SDD,6-311+G(2d,p)//IEFPCM/B3LYP-D3/SDD,6-31G(d)) = -5856.127617 au

Mn 0 1.10703200 2.09465300 -1.94820700

Mn 0 -1.06572100 -2.60253400 -0.88576500

Mn 0 -0.21324300 0.89283200 0.15221300

Mn 0 -2.77494300 -0.45411900 0.35638100

O 0 0.20560800 0.49160200 -1.52559800

O 0 -2.01215200 1.00958600 -0.36648000

O 0 -2.64822900 -1.44393300 -1.11690900

O 0 -0.97013100 -0.97349100 0.72626900

Ca 0 -1.93279700 0.17077000 -2.74358900

C -1 0.51119600 4.28263100 -5.81856200

H -1 0.56137900 5.24350400 -5.32792500

H -1 0.70440100 4.30747300 -6.88060600

H 0 1.31917600 3.68336500 -5.37999500

C 0 -0.82116100 3.56971500 -5.52155200

H 0 -0.94733700 2.70374200 -6.18255000

H 0 -1.68105800 4.22299600 -5.72513600

C 0 -0.97471900 3.02651600 -4.09082100

O 0 -1.92314000 2.21169600 -3.88035700

O 0 -0.14649400 3.40446800 -3.20815300

C -1 -2.80856800 -6.99205300 -5.00126800

H -1 -1.84142300 -7.45089000 -5.14446500

H -1 -3.53500600 -7.18306600 -5.77728100

H 0 -3.20780300 -7.43084100 -4.07649400

C 0 -2.49672400 -5.51414900 -4.76366600

H 0 -3.26305200 -5.03888100 -4.13829100

H 0 -2.45063400 -4.94800600 -5.70053900

C 0 -1.11775900 -5.46276400 -4.06952400

H 0 -0.32670000 -5.57118100 -4.81875000

H 0 -1.03509300 -6.27964000 -3.34632300

C 0 -0.96886800 -4.16001800 -3.33862300

O 0 -0.72553500 -3.12340200 -4.00142700

O 0 -1.20800500 -4.22240300 -2.07894300

C -1 5.12659300 -4.51786000 1.24504400

H 0 5.90509900 -5.23616300 1.52972500

H 0 5.47025800 -4.00644400 0.34221000

C 0 4.94079500 -3.52244000 2.38703700

O 0 4.31114800 -3.83610800 3.41788000

C 0 3.81763200 -5.31540400 0.96713100

H 0 3.61180000 -5.94200400 1.84374500

H 0 3.98504200 -5.98646400 0.11989100

C 0 2.61757800 -4.45401000 0.68818500

N 0 2.27710900 -3.45447800 1.58326400

H 0 2.72673300 -3.33009900 2.48734400

C 0 1.67974500 -4.35135700 -0.31656100

H 0 1.57038300 -4.93624200 -1.21744300

C 0 1.22793000 -2.77395600 1.10643400

H 0 0.83694700 -1.90448700 1.58984600

N 0 0.80889800 -3.30119900 -0.03439600

N 0 5.38283500 -2.27449300 2.15785300

H 0 5.98197800 -2.11742000 1.34169200

C -1 5.04678500 -1.14855000 3.02165700

H 0 5.90446000 -0.46431100 3.02159200

H 0 4.91450400 -1.52532900 4.03852100

C 0 3.78079200 -0.40123300 2.56011200

H 0 3.56452100 0.40198600 3.27546800

H 0 2.92475700 -1.07876700 2.59383900

C 0 3.94204900 0.17040100 1.15281900

H 0 4.41806300 -0.56889500 0.49625200

H 0 4.60786900 1.04202000 1.15652800

C 0 2.68735700 0.59650100 0.40241300

O 0 2.86791200 1.03834900 -0.75549300

O 0 1.54359600 0.42803600 0.97021900

C -1 -5.39078700 -3.95486600 2.40844800

H 0 -5.28513800 -3.16490200 3.15900800

H 0 -5.85102100 -4.81898100 2.90302400

C 0 -4.01590900 -4.36469600 1.85884700

H 0 -4.12120600 -5.17983800 1.13798300

H 0 -3.39476400 -4.74776600 2.67784900

C 0 -3.20419000 -3.26545200 1.16881500

O 0 -3.57118200 -2.06309700 1.33347300

O 0 -2.18692200 -3.64935900 0.50964800

C 0 -6.32332400 -3.49487700 1.29161000

O 0 -6.16138900 -3.82959100 0.11081700

N 0 -7.38137100 -2.74616400 1.68849800

H 0 -7.40594400 -2.40136900 2.63854000

C -1 -8.38340300 -2.24117800 0.75760100

H 0 -9.33000700 -2.12229400 1.28654300

H 0 -8.50105200 -2.97701200 -0.04196300

C 0 -7.99266900 -0.86385600 0.18572800

O 0 -8.49648900 0.17141500 0.62492000

N 0 -7.07227000 -0.89978300 -0.80943900

H 0 -6.61821200 -1.77541000 -1.05550200

C 0 -6.51262900 0.29814000 -1.40159900

H 0 -6.71788300 0.33680400 -2.47502400

H 0 -6.99077500 1.15787300 -0.92223000

C 0 -4.99399000 0.39925700 -1.21920000

O 0 -4.29017100 0.77351100 -2.16977800

O 0 -4.57599600 0.08414400 -0.03260600

C -1 -3.48180400 3.82495600 4.79824500

H -1 -3.51171100 3.58118700 5.84995000

H -1 -4.30607000 4.44310100 4.47442500

H 0 -2.57613600 4.43197400 4.65385200

C 0 -3.44073400 2.63863200 3.84396700

H 0 -3.50343900 3.03763200 2.82294500

H 0 -4.31661100 1.99244000 3.96682000

C 0 -2.15894800 1.80131700 3.96901300

H 0 -2.28081400 1.03344600 4.73991200

H 0 -1.30103700 2.42487400 4.23670300

C 0 -1.87889400 1.14281400 2.64066300

O 0 -0.76578700 1.38758200 2.08325900

O 0 -2.81428700 0.42394300 2.16364900

C -1 -5.62340900 9.05342800 1.54341000

H -1 -5.48432600 8.69524700 2.55274900

H -1 -6.66121600 9.26675800 1.33411400

H 0 -5.07008200 9.99408800 1.43881700

C 0 -5.07997100 7.98880600 0.58345400

H 0 -3.98812800 7.94476400 0.70040400

H 0 -5.25843500 8.27160400 -0.46209100

C 0 -5.70764300 6.61310600 0.85757800

H 0 -6.77090500 6.63584800 0.59071000

H 0 -5.65780000 6.38852900 1.93213600

C 0 -5.04840300 5.46404800 0.09566000

H 0 -5.06622200 5.66846000 -0.98496400

H 0 -5.59888000 4.53357600 0.27539300

N 0 -3.67112400 5.28471900 0.55908400

H 0 -3.21511600 6.09070600 0.96413500

C 0 -2.85002600 4.30606400 0.12590400

N 0 -3.28127400 3.37961600 -0.73404800

H 0 -2.80636400 2.46157000 -0.74472100

H 0 -4.19531500 3.43479300 -1.15488400

N 0 -1.58981300 4.25801400 0.55430000

H 0 -1.26285200 4.74201400 1.38078100

H 0 -0.91613800 3.58009900 0.15124600

C -1 -0.97551300 -0.97269000 7.58752400

H -1 0.00803900 -0.57165400 7.39205000

H -1 -1.15415300 -1.12954800 8.64101500

H 0 -1.72456800 -0.27359400 7.19978100

C 0 -1.12603900 -2.36428000 6.95081200

H 0 -2.11056500 -2.77694800 7.21391100

H 0 -0.38751200 -3.04392200 7.39976500

C 0 -0.97541200 -2.37604000 5.46792000

N 0 -1.10228700 -3.53311200 4.71836700

C 0 -0.73304400 -1.38789100 4.54510300

H 0 -0.54041100 -0.34154300 4.71999800

C 0 -0.94190300 -3.21430800 3.40898000

H 0 -0.98331900 -3.93308900 2.60495900

N 0 -0.72633100 -1.91762000 3.27070800

H 0 -0.88602400 -1.21716900 1.70283600

C -1 3.68099200 6.36185700 2.05441900

H -1 4.54344300 6.84679000 1.62148100

H -1 3.20486500 6.91386400 2.85128100

H 0 2.92544200 6.28523000 1.26353900

C 0 4.04532400 4.92452900 2.50315800

H 0 4.99662500 4.89829000 3.04418600

H 0 3.25550000 4.54217600 3.16010600

C 0 4.08845300 4.00135200 1.28497600

O 0 2.99812100 3.86901600 0.66556300

O 0 5.18371100 3.45340300 0.93610000

C -1 12.15615000 -2.27304800 -0.49038700

H -1 13.19888200 -1.99886700 -0.55310600

H -1 11.94616700 -3.19319700 -1.01538800

H 0 11.94639600 -2.48564100 0.56750100

C 0 11.24174100 -1.12382600 -0.97263900

H 0 11.68902400 -0.63942800 -1.85293700

H 0 10.27632200 -1.53298700 -1.29118300

C 0 10.98908600 -0.07899500 0.12974400

H 0 10.28640300 -0.50807000 0.85687600

H 0 11.92374500 0.10547900 0.67802700

C 0 10.47685600 1.28692000 -0.35895900

H 0 10.37479900 1.96897100 0.49647200

H 0 11.23350500 1.73240100 -1.01889300

C 0 9.16453300 1.29879100 -1.14427600

H 0 8.97948800 2.30176300 -1.53930400

H 0 9.18980700 0.60383900 -1.98748500

N 0 7.96724100 0.93567400 -0.32139200

H 0 7.10381400 1.25236500 -0.81337400

H 0 7.90243900 -0.10191300 -0.17202600

H 0 7.93155900 1.42765800 0.60208700

Cl 0 7.68401600 -2.15654000 -0.11909700

O 0 1.83436700 1.32530300 -3.54428700

H 0 3.35565600 2.15410300 -3.75057600

O 0 0.30371300 2.51957300 -0.27116200

O 0 0.72013500 3.88782700 2.38401100

H 0 0.31693000 3.00661100 2.28118700

H 0 1.40835200 3.89700200 1.68194600

O 0 -0.26684400 -0.18691400 -4.54724000

H 0 0.47605600 0.42660400 -4.29929000

H 0 0.03805600 -1.07576300 -4.28791800

O 0 -3.05069300 -1.55813900 -4.09959100

H 0 -3.71872200 -2.04243900 -3.56888700

H 0 -2.29671200 -2.18077400 -4.19838900

O 0 -4.83205100 -2.57409700 -2.08060900

H 0 -4.05852700 -2.17535000 -1.59638900

H 0 -5.10870700 -3.31609100 -1.51216300

O 0 2.36969400 3.72869900 -1.86285800

H 0 2.73447200 3.71378800 -0.92570900

H 0 2.69043900 -0.61917400 -1.75790300

O 0 -0.27391100 -1.73096400 -2.15214600

O 0 5.82675900 2.39128800 -1.42682200

H 0 5.38231400 2.70775800 -0.58487200

H 0 6.31487000 3.17717200 -1.72540000

O 0 4.12586300 2.77089500 -3.61764200

H 0 4.66414100 2.35814400 -2.90685800

H 0 3.12049800 3.54054200 -2.49913600

O 0 7.40988300 2.39015100 2.01884500

H 0 6.59733400 2.87970800 1.72092500

H 0 7.09717000 1.80893200 2.72915100

O 0 2.46224700 -1.24171200 -2.47380600

H 0 2.15408600 0.42938500 -3.28304400

H 0 1.51719300 -1.44829100 -2.29131400

H 0 -1.26962700 -4.46025200 5.08562700

**^8′′^TS_1_**

E (IEFPCM/B3LYP-D3/SDD,6-31G(d)) = -5854.284654 au

H (IEFPCM/B3LYP-D3/SDD,6-31G(d)) = -5852.732156 au

G (IEFPCM/B3LYP-D3/SDD,6-31G(d)) = -5852.971029 au

E (IEFPCM/B3LYP-D3/SDD,6-311+G(2d,p)//IEFPCM/B3LYP-D3/SDD,6-31G(d)) = -5856.10734 au

Mn 0 1.20682900 1.80946600 -2.04646200

Mn 0 -1.03828400 -2.47985900 -0.84806800

Mn 0 -0.27121300 0.88045400 0.25812000

Mn 0 -2.82071300 -0.41739200 0.38718300

O 0 0.10284700 0.13146500 -1.45351600

O 0 -2.05716100 1.05146600 -0.30352200

O 0 -2.64392100 -1.38578800 -1.10771900

O 0 -1.04470900 -0.95634300 0.82742900

Ca 0 -2.00773700 0.18939700 -2.74258800

C -1 0.52318500 4.29240300 -5.82791600

H -1 0.57589400 5.25477400 -5.34048800

H -1 0.71666200 4.31316300 -6.88999900

H 0 1.34463400 3.70719400 -5.39650900

C 0 -0.79282500 3.56383000 -5.50716300

H 0 -0.95383600 2.73771200 -6.21232700

H 0 -1.66752900 4.21734200 -5.61516100

C 0 -0.85664600 2.90941800 -4.11979500

O 0 -1.90237100 2.27327300 -3.81086600

O 0 0.17125800 3.02135500 -3.37966100

C -1 -2.82749600 -6.97038200 -4.97356000

H -1 -1.86157900 -7.43233200 -5.11502700

H -1 -3.55430300 -7.16200900 -5.74907600

H 0 -3.22707500 -7.39563800 -4.04291600

C 0 -2.52990000 -5.48388400 -4.77198000

H 0 -3.29891400 -5.00669700 -4.15057400

H 0 -2.50880600 -4.94027700 -5.72299000

C 0 -1.14663600 -5.36844300 -4.09878800

H 0 -0.36422100 -5.51272600 -4.85239500

H 0 -1.03932800 -6.13789300 -3.32772400

C 0 -1.02365900 -4.00841000 -3.46690800

O 0 -0.90288800 -3.01256200 -4.22149700

O 0 -1.17022100 -4.00234400 -2.19289000

C -1 5.11318700 -4.49695400 1.26603400

H 0 5.87750300 -5.22566000 1.56201400

H 0 5.47563400 -3.99021700 0.36773600

C 0 4.92506500 -3.49732900 2.40389700

O 0 4.29450300 -3.80678600 3.43587700

C 0 3.79793900 -5.27693000 0.96753000

H 0 3.58528500 -5.92282600 1.82836200

H 0 3.96325300 -5.92943600 0.10555500

C 0 2.60029100 -4.40647300 0.70715300

N 0 2.22938700 -3.46021200 1.64769300

H 0 2.68034000 -3.36288300 2.55555000

C 0 1.68182200 -4.26203600 -0.30990200

H 0 1.59753700 -4.79914100 -1.24239000

C 0 1.17952800 -2.76931000 1.18683400

H 0 0.75499700 -1.94028100 1.71224900

N 0 0.79150600 -3.24060000 0.01023900

N 0 5.37165000 -2.25191500 2.17115100

H 0 5.97663300 -2.09881500 1.35808100

C -1 5.04223200 -1.12151000 3.03134400

H 0 5.89754000 -0.43485500 3.01812700

H 0 4.91948400 -1.49117800 4.05211700

C 0 3.76817600 -0.38549200 2.57651400

H 0 3.54381400 0.41456200 3.29260200

H 0 2.92274600 -1.07604300 2.61430900

C 0 3.90968900 0.18862800 1.16627300

H 0 4.42583900 -0.52307700 0.51071000

H 0 4.52783100 1.09560900 1.17019700

C 0 2.62700500 0.54853900 0.43276700

O 0 2.76448400 0.85556700 -0.78213300

O 0 1.51541300 0.48266500 1.06321500

C -1 -5.40284200 -3.90137300 2.42542900

H 0 -5.27644400 -3.10183200 3.16194600

H 0 -5.86506400 -4.75431000 2.93722500

C 0 -4.03953700 -4.33477000 1.85537000

H 0 -4.16859800 -5.15123200 1.13983800

H 0 -3.41206700 -4.72333800 2.66636400

C 0 -3.23098400 -3.24036500 1.14883100

O 0 -3.62052500 -2.04385000 1.31046200

O 0 -2.21061400 -3.61162500 0.48686800

C 0 -6.34209000 -3.44924200 1.31102800

O 0 -6.19881400 -3.81100000 0.13554000

N 0 -7.38489700 -2.67708000 1.70170300

H 0 -7.39752400 -2.31245700 2.64454400

C -1 -8.39045200 -2.18506100 0.76825200

H 0 -9.33703900 -2.06590400 1.29769000

H 0 -8.50579700 -2.92949400 -0.02332900

C 0 -8.00861100 -0.81229000 0.18345000

O 0 -8.50424000 0.22603800 0.62495000

N 0 -7.10696600 -0.85581200 -0.82807000

H 0 -6.64215700 -1.72951400 -1.06165500

C 0 -6.54266400 0.33841500 -1.42148200

H 0 -6.73258200 0.36770800 -2.49790600

H 0 -7.02908100 1.20134400 -0.95632900

C 0 -5.02772600 0.44281400 -1.21746400

O 0 -4.31612200 0.83526500 -2.15688600

O 0 -4.62175300 0.11193800 -0.03372200

C -1 -3.47309200 3.88116700 4.78955800

H -1 -3.50386700 3.64100200 5.84206700

H -1 -4.29560600 4.50047200 4.46350400

H 0 -2.55104300 4.45334900 4.61936400

C 0 -3.50183600 2.66690000 3.87587400

H 0 -3.50004900 3.03086700 2.83977000

H 0 -4.43585500 2.10629000 3.99214600

C 0 -2.30570400 1.72377100 4.08181800

H 0 -2.55746500 0.93266600 4.79472200

H 0 -1.43084900 2.26516400 4.45359100

C 0 -1.97323600 1.12510500 2.74152900

O 0 -0.85608000 1.41605600 2.22143000

O 0 -2.89314200 0.41689000 2.21254900

C -1 -5.59979400 9.10453800 1.51681000

H -1 -5.46188200 8.74936000 2.52737100

H -1 -6.63697400 9.31999800 1.30659300

H 0 -5.05408700 10.05096500 1.42000900

C 0 -5.02935200 8.05242600 0.55734500

H 0 -3.93360900 8.07105300 0.64215500

H 0 -5.25752400 8.30678700 -0.48604800

C 0 -5.56230600 6.64819100 0.87518900

H 0 -6.64799200 6.62606300 0.71988200

H 0 -5.39005800 6.41739200 1.93551800

C 0 -4.94524700 5.53478100 0.02958300

H 0 -5.10919900 5.73912900 -1.03894600

H 0 -5.42909100 4.58170900 0.26970500

N 0 -3.51418700 5.40163300 0.31020800

H 0 -3.01221400 6.23577900 0.58254300

C 0 -2.75620900 4.37646000 -0.13053300

N 0 -3.29047700 3.39783100 -0.86357600

H 0 -2.84851900 2.46608000 -0.82672800

H 0 -4.22089500 3.46083800 -1.24509400

N 0 -1.45050700 4.34138200 0.14987400

H 0 -1.05801300 4.75430700 0.99162400

H 0 -0.85792100 3.58511300 -0.24059400

C -1 -0.98044200 -0.91393600 7.59538300

H -1 0.00423900 -0.51624100 7.39876700

H -1 -1.15971200 -1.06677900 8.64935700

H 0 -1.73266600 -0.22499300 7.19729200

C 0 -1.12952900 -2.32371900 6.99690400

H 0 -2.10400500 -2.73823300 7.29265400

H 0 -0.37486000 -2.98385900 7.44801800

C 0 -1.01397100 -2.36915300 5.51452700

N 0 -1.14759500 -3.53893400 4.78655900

C 0 -0.81091100 -1.39102800 4.57381700

H 0 -0.62875900 -0.33977300 4.72870000

C 0 -1.03028300 -3.23721900 3.46885200

H 0 -1.07896700 -3.96534500 2.67363100

N 0 -0.83518500 -1.93991400 3.30971900

H 0 -0.99319100 -1.23255800 1.80178900

C -1 3.69712800 6.38931500 2.03870100

H -1 4.56098300 6.87044200 1.60431500

H -1 3.22235700 6.94528400 2.83361400

H 0 2.94253100 6.31283300 1.24596900

C 0 4.04484500 4.95130300 2.50147900

H 0 4.98648400 4.92696900 3.05918500

H 0 3.24351600 4.58512300 3.15385100

C 0 4.10909800 3.99690800 1.30607600

O 0 3.03175600 3.80932500 0.67864400

O 0 5.22350500 3.46570800 0.98574000

C -1 12.14917800 -2.27715200 -0.47548400

H -1 13.19266600 -2.00602900 -0.53891000

H -1 11.93678500 -3.19847800 -0.99744400

H 0 11.93788500 -2.48405600 0.58331400

C 0 11.24819500 -1.12084500 -0.95895800

H 0 11.67483300 -0.67423000 -1.86903400

H 0 10.26055000 -1.51168300 -1.22841700

C 0 11.07241700 -0.04035000 0.12318700

H 0 10.39502900 -0.43061000 0.89510400

H 0 12.03544500 0.13381400 0.62366500

C 0 10.57193600 1.32393500 -0.38003900

H 0 10.51152200 2.02723800 0.46196500

H 0 11.31380000 1.73908100 -1.07549400

C 0 9.23495900 1.33919400 -1.12080500

H 0 9.04101100 2.34050300 -1.51583500

H 0 9.22890700 0.63900600 -1.96008400

N 0 8.06454600 0.98565000 -0.25744700

H 0 7.18671800 1.29810200 -0.72980100

H 0 7.99660800 -0.04932400 -0.09874900

H 0 8.05142700 1.48970700 0.65893800

Cl 0 7.68347700 -2.10328100 -0.07826400

O 0 1.94974100 0.93824900 -3.54577700

H 0 3.50441600 1.76875300 -3.74050100

O 0 0.31670900 2.38412900 -0.52116900

O 0 0.61593500 4.00260000 2.19671900

H 0 0.23513600 3.10594100 2.21407500

H 0 1.37225000 3.92736600 1.57102000

O 0 -0.38646300 -0.24419000 -4.56986300

H 0 0.46083300 0.19822200 -4.30625000

H 0 -0.26230200 -1.19591700 -4.36555600

O 0 -3.18494400 -1.48641100 -4.08512900

H 0 -3.83309000 -1.98279300 -3.54110200

H 0 -2.44046100 -2.11555600 -4.24092100

O 0 -4.85028100 -2.53298600 -2.02187300

H 0 -4.05858900 -2.14709400 -1.55719100

H 0 -5.13079500 -3.26994500 -1.44829700

O 0 2.54490300 3.50932100 -1.92774400

H 0 2.85056000 3.53015300 -0.97866200

H 0 2.84594700 -0.93796300 -1.66329700

O 0 -0.14833300 -1.50644500 -2.06465400

O 0 5.87864200 2.34465000 -1.33335800

H 0 5.45957000 2.71263200 -0.49720300

H 0 6.29228000 3.12334800 -1.74194900

O 0 4.29384800 2.36007400 -3.62975100

H 0 4.78091700 1.99729300 -2.86057900

H 0 3.31457800 3.26824500 -2.50964400

O 0 7.50346600 2.48025200 2.06250200

H 0 6.67639000 2.94229100 1.76478000

H 0 7.21041300 1.89832400 2.78059800

O 0 2.57362500 -1.54047200 -2.37910100

H 0 2.25769000 0.04736200 -3.24632800

H 0 1.60871500 -1.66158800 -2.20533500

H 0 -1.29307400 -4.46198300 5.17279800

**^8′′^L_P**

E (IEFPCM/B3LYP-D3/SDD,6-31G(d)) = -5854.305288 au

H (IEFPCM/B3LYP-D3/SDD,6-31G(d)) = -5852.752621 au

G (IEFPCM/B3LYP-D3/SDD,6-31G(d)) = -5852.990372 au

E (IEFPCM/B3LYP-D3/SDD,6-311+G(2d,p)//IEFPCM/B3LYP-D3/SDD,6-31G(d)) = -5856.129272 au

Mn 0 1.21889200 1.70864000 -2.06771300

Mn 0 -0.98505300 -2.43430100 -0.79852100

Mn 0 -0.28782800 0.88600300 0.30497100

Mn 0 -2.82864500 -0.40036000 0.35548000

O 0 -0.05909400 0.04440800 -1.66794300

O 0 -2.11388400 1.06182700 -0.31752200

O 0 -2.56614500 -1.38347400 -1.14818200

O 0 -1.04792200 -0.91446500 0.85412100

Ca 0 -2.13113100 0.24861200 -2.82050500

C -1 0.51395700 4.34868000 -5.78994200

H -1 0.56470900 5.30800300 -5.29633700

H -1 0.70596400 4.37686600 -6.85212100

H 0 1.33511900 3.75679200 -5.36545200

C 0 -0.80185100 3.61580400 -5.48249800

H 0 -0.97268000 2.81885600 -6.21920000

H 0 -1.67468700 4.27571800 -5.55664100

C 0 -0.86368500 2.90050500 -4.12599200

O 0 -1.95052500 2.33881700 -3.80998200

O 0 0.19518800 2.88221200 -3.42730300

C -1 -2.80507300 -6.92847900 -5.00443200

H -1 -1.83809700 -7.38687400 -5.15016700

H -1 -3.53238900 -7.11703600 -5.78022300

H 0 -3.20301200 -7.36606300 -4.07851600

C 0 -2.51860900 -5.43964000 -4.78380100

H 0 -3.30361800 -4.97051800 -4.17565800

H 0 -2.48239900 -4.88940500 -5.73054900

C 0 -1.15205700 -5.32217100 -4.08213800

H 0 -0.35746800 -5.50022100 -4.81746900

H 0 -1.06653000 -6.07853700 -3.29539600

C 0 -0.98010900 -3.95350300 -3.47086400

O 0 -0.97754700 -2.95888700 -4.23973500

O 0 -0.92080500 -3.93555200 -2.19421300

C -1 5.13717500 -4.47408500 1.24068500

H 0 5.91081500 -5.20150800 1.51456000

H 0 5.47931800 -3.95617800 0.34083400

C 0 4.96248500 -3.48688000 2.39209000

O 0 4.35120000 -3.81104100 3.43125700

C 0 3.81976300 -5.25722300 0.96166200

H 0 3.62865900 -5.91393500 1.81926100

H 0 3.96901300 -5.89865000 0.08853100

C 0 2.61312600 -4.38969100 0.73630300

N 0 2.24884600 -3.46103600 1.69758800

H 0 2.71532800 -3.37347900 2.59887400

C 0 1.68180500 -4.23474700 -0.26683900

H 0 1.58256900 -4.75469600 -1.20714200

C 0 1.19025700 -2.76726300 1.26131400

H 0 0.76345400 -1.94953100 1.80368500

N 0 0.79227100 -3.22337400 0.08216500

N 0 5.39494800 -2.23598400 2.16336200

H 0 5.98934200 -2.07261200 1.34448600

C -1 5.05942800 -1.11038900 3.02799400

H 0 5.91331000 -0.42177100 3.02158800

H 0 4.93285700 -1.48473000 4.04647500

C 0 3.78559600 -0.37768500 2.56906600

H 0 3.56652200 0.43259200 3.27511600

H 0 2.94017700 -1.06676500 2.62404200

C 0 3.90848100 0.17720500 1.14741700

H 0 4.43145900 -0.53289400 0.49630300

H 0 4.50775900 1.09700900 1.13367200

C 0 2.60338400 0.50155300 0.43855300

O 0 2.69223900 0.74382700 -0.79969800

O 0 1.51746200 0.47877800 1.10573000

C -1 -5.37887700 -3.91462100 2.41772700

H 0 -5.24525300 -3.11812300 3.15599300

H 0 -5.83919700 -4.76948000 2.92787200

C 0 -4.02039500 -4.34751100 1.83203500

H 0 -4.15852100 -5.15951900 1.11289400

H 0 -3.38603300 -4.74297200 2.63378200

C 0 -3.22085600 -3.24590800 1.12401400

O 0 -3.63031000 -2.05772200 1.26589800

O 0 -2.18451700 -3.60580500 0.47499400

C 0 -6.32301200 -3.45650400 1.30956900

O 0 -6.19266500 -3.82406900 0.13345800

N 0 -7.35548200 -2.67378000 1.70271400

H 0 -7.36387200 -2.30875800 2.64540100

C -1 -8.37332500 -2.19569900 0.77566700

H 0 -9.31477300 -2.07898500 1.31512400

H 0 -8.49347800 -2.94873000 -0.00694700

C 0 -8.01093400 -0.82878000 0.17308900

O 0 -8.49869900 0.21286400 0.61453500

N 0 -7.13579700 -0.88280900 -0.86071100

H 0 -6.66501500 -1.75680000 -1.07772500

C 0 -6.57739100 0.30644500 -1.46489000

H 0 -6.76286100 0.32292600 -2.54229500

H 0 -7.07058700 1.17235900 -1.01248500

C 0 -5.06564200 0.42250800 -1.25650600

O 0 -4.36842400 0.86050000 -2.18868400

O 0 -4.64858500 0.05367000 -0.09211300

C -1 -3.46706700 3.85760700 4.82986900

H -1 -3.49579100 3.61052600 5.88083500

H -1 -4.29168700 4.47678100 4.50892900

H 0 -2.53241500 4.39782900 4.63486000

C 0 -3.57606400 2.63156300 3.93361900

H 0 -3.55905700 2.97886000 2.89215300

H 0 -4.54924500 2.14509800 4.06500900

C 0 -2.45217100 1.60297100 4.14766900

H 0 -2.79178500 0.77888200 4.78188200

H 0 -1.57544900 2.06543800 4.61083700

C 0 -2.06695400 1.08953900 2.78587300

O 0 -0.94512300 1.42690300 2.31775100

O 0 -2.96614500 0.40071600 2.18862700

C -1 -5.61225600 9.09633800 1.59394400

H -1 -5.47203900 8.73497600 2.60199300

H -1 -6.65029500 9.31034100 1.38649400

H 0 -5.07431700 10.04854800 1.50840700

C 0 -5.02515900 8.06135800 0.62446100

H 0 -3.92931800 8.10879800 0.69590400

H 0 -5.27390700 8.31278900 -0.41501300

C 0 -5.51198000 6.64116900 0.94333600

H 0 -6.60230100 6.59589600 0.82957600

H 0 -5.29380500 6.40112900 1.99308300

C 0 -4.90782200 5.55157900 0.05772700

H 0 -5.11684600 5.77007500 -1.00013400

H 0 -5.37014300 4.58872000 0.30067500

N 0 -3.46497200 5.42718700 0.27774100

H 0 -2.95324700 6.26144100 0.53116000

C 0 -2.72656100 4.39319200 -0.17746600

N 0 -3.28877300 3.41760000 -0.89362500

H 0 -2.87034300 2.47400600 -0.83852400

H 0 -4.22737700 3.49201600 -1.25270500

N 0 -1.41268900 4.34692900 0.06761300

H 0 -1.00389400 4.73136500 0.91690100

H 0 -0.84210700 3.57638200 -0.33045700

C -1 -0.95771100 -0.94883700 7.60118700

H -1 0.02562700 -0.54719900 7.40586800

H -1 -1.13516400 -1.10901200 8.65437900

H 0 -1.71339500 -0.26124100 7.20760900

C 0 -1.10375200 -2.35751800 6.99978400

H 0 -2.06959400 -2.78178400 7.30987700

H 0 -0.33599800 -3.01297500 7.43527100

C 0 -1.01394800 -2.39296800 5.51626000

N 0 -1.13939600 -3.55947900 4.78208700

C 0 -0.84987200 -1.40339100 4.58033400

H 0 -0.68715600 -0.34978500 4.73999500

C 0 -1.05577900 -3.24539800 3.46502100

H 0 -1.09924700 -3.96857400 2.66529100

N 0 -0.88995600 -1.94293500 3.31294300

H 0 -1.01665900 -1.21055500 1.82559900

C -1 3.69267100 6.40304700 2.08596900

H -1 4.55464100 6.88933000 1.65358700

H -1 3.21745300 6.95254600 2.88510200

H 0 2.93695400 6.34394300 1.29189000

C 0 4.02593700 4.95329900 2.52157000

H 0 4.96549600 4.90927500 3.08158500

H 0 3.21949300 4.58490300 3.16655700

C 0 4.08664100 4.00946900 1.31465600

O 0 3.01350200 3.83052600 0.67750400

O 0 5.20017400 3.47164900 1.00028000

C -1 12.16480600 -2.22390800 -0.49559700

H -1 13.20747100 -1.94954500 -0.55862900

H -1 11.95421600 -3.14239700 -1.02325700

H 0 11.95591700 -2.43857100 0.56215500

C 0 11.26031800 -1.06611700 -0.96895300

H 0 11.68187600 -0.61487500 -1.87910800

H 0 10.27136400 -1.45614000 -1.23501600

C 0 11.09023800 0.00907400 0.11948100

H 0 10.41727200 -0.38483300 0.89345400

H 0 12.05599100 0.18052300 0.61563200

C 0 10.58765900 1.37624300 -0.37382700

H 0 10.53219600 2.07496100 0.47231700

H 0 11.32629500 1.79477200 -1.07069400

C 0 9.24710000 1.39673400 -1.10804600

H 0 9.05147700 2.40084100 -1.49509100

H 0 9.23732200 0.70238200 -1.95216700

N 0 8.08049100 1.03659300 -0.24245200

H 0 7.19886700 1.34538500 -0.71141300

H 0 8.01564700 0.00089200 -0.08855700

H 0 8.06650800 1.53717600 0.67551800

Cl 0 7.68939800 -2.05404100 -0.10098500

O 0 1.99124000 0.85258300 -3.56553800

H 0 3.52777200 1.69375300 -3.75011700

O 0 0.30481500 2.32377700 -0.58907300

O 0 0.57683200 4.00965500 2.17964200

H 0 0.19585900 3.11487400 2.23853000

H 0 1.34036700 3.91353200 1.56552300

O 0 -0.44352500 -0.26615200 -4.55007700

H 0 0.41419100 0.14938900 -4.28290300

H 0 -0.35045200 -1.22318000 -4.33969000

O 0 -3.27988300 -1.47904200 -4.12476700

H 0 -3.91182800 -1.97817600 -3.56450900

H 0 -2.53438800 -2.10538600 -4.28798400

O 0 -4.79249200 -2.57841700 -2.00248800

H 0 -3.99384100 -2.18725100 -1.55737600

H 0 -5.10855200 -3.26596000 -1.38652700

O 0 2.54678900 3.39940700 -1.93903200

H 0 2.83599200 3.46383700 -0.98785700

H 0 3.07695100 -0.98517100 -1.70599500

O 0 0.08321000 -1.36486400 -1.92244300

O 0 5.86087000 2.34381400 -1.30888400

H 0 5.44528400 2.72230300 -0.47491200

H 0 6.23900900 3.12283600 -1.74991700

O 0 4.32122100 2.28093100 -3.63287300

H 0 4.79592300 1.91772200 -2.85734700

H 0 3.33150900 3.17587600 -2.50639700

O 0 7.49424700 2.51908900 2.08076500

H 0 6.66170700 2.96926200 1.78036600

H 0 7.20631400 1.93135100 2.79618400

O 0 2.74922000 -1.60448700 -2.38372100

H 0 2.32552300 -0.03054800 -3.27824000

H 0 1.79170800 -1.67568600 -2.15207800

H 0 -1.25902200 -4.48816900 5.16364600

**^8′^L_S_4_** (for the case with a net charge of zero on the OEC)

E (IEFPCM/B3LYP-D3/SDD,6-31G(d)) = -5393.917470 au

H (IEFPCM/B3LYP-D3/SDD,6-31G(d)) = -5392.364161 au

G (IEFPCM/B3LYP-D3/SDD,6-31G(d)) = -5392.596444 au

E (IEFPCM/B3LYP-D3/SDD,6-311+G(2d,p)//IEFPCM/B3LYP-D3/SDD,6-31G(d)) = -5395.699252 au

Mn 0 1.31214200 2.18305100 -1.88946500

Mn 0 -0.84874800 -2.46320500 -0.86967700

Mn 0 0.01225700 0.80332000 0.10112300

Mn 0 -2.51267100 -0.50974000 0.31589700

O 0 0.38217700 0.60381500 -1.61435100

O 0 -1.78250400 1.00571300 -0.33228200

O 0 -2.39211000 -1.43052900 -1.23741100

O 0 -0.77279800 -1.10600600 0.58620100

Ca 0 -1.82568100 0.38477800 -2.73563300

C -1 0.77041000 4.55359800 -5.65131000

H -1 0.87337400 5.48115300 -5.10765300

H -1 0.95082200 4.63033100 -6.71312400

H 0 1.55925800 3.89703100 -5.26139000

C 0 -0.58248100 3.88081800 -5.36753700

H 0 -0.76618300 3.06894900 -6.08120600

H 0 -1.41756800 4.58331600 -5.49779100

C 0 -0.71433800 3.25388900 -3.97133300

O 0 -1.66094100 2.42844100 -3.80076500

O 0 0.12105600 3.58253400 -3.07587900

C -1 -3.08011800 -6.57875900 -5.42472400

H -1 -2.13824200 -7.07406900 -5.60895900

H -1 -3.82493600 -6.69000700 -6.19884800

H 0 -3.50465700 -7.10783700 -4.55652000

C 0 -2.79176000 -5.14390000 -5.00864500

H 0 -3.69673200 -4.65347000 -4.62710300

H 0 -2.43276200 -4.53769600 -5.84711700

C 0 -1.72622700 -5.18648100 -3.91140600

H 0 -0.76461200 -5.50922600 -4.33702000

H 0 -1.99198400 -5.91859300 -3.14141200

C 0 -1.51206700 -3.84318400 -3.25880100

O 0 -1.56586200 -2.80927000 -3.93459800

O 0 -1.28793700 -3.93149300 -1.98017500

C -1 5.04568300 -4.84854100 0.82687500

H 0 5.85731000 -5.57706300 0.93975600

H 0 5.22441400 -4.28987100 -0.09751700

C 0 5.04494000 -3.93572300 2.04333200

O 0 4.63871600 -4.33711200 3.14377200

C 0 3.70141400 -5.62675600 0.72800800

H 0 3.60651900 -6.24876700 1.62562500

H 0 3.73493800 -6.29623700 -0.13547500

C 0 2.51599500 -4.71609900 0.60184800

N 0 2.32129400 -3.70023400 1.52536600

H 0 2.83460600 -3.60957800 2.39433900

C 0 1.52058200 -4.53423400 -0.33208700

H 0 1.30293300 -5.09228200 -1.22969400

C 0 1.29327300 -2.93321500 1.12359500

H 0 1.02036400 -2.00037500 1.57807200

N 0 0.76254600 -3.42868900 0.01917200

N 0 5.41012400 -2.65082000 1.82678900

H 0 5.64353600 -2.38377100 0.88039700

C -1 5.15200300 -1.58713600 2.79332100

H 0 6.05493500 -0.96836800 2.87912000

H 0 4.98797900 -2.07054500 3.75738300

C 0 3.95319200 -0.70848700 2.40559100

H 0 3.78239200 0.02364300 3.20393000

H 0 3.05387000 -1.32252400 2.35540700

C 0 4.16147100 0.01074300 1.07269300

H 0 4.64779000 -0.64648400 0.33988100

H 0 4.82630700 0.87365200 1.18657500

C 0 2.91527500 0.49968400 0.34293000

O 0 3.10726900 1.06063900 -0.75882300

O 0 1.77295500 0.24152400 0.86695600

C -1 -5.41619100 -3.85386400 2.18539700

H 0 -5.24325400 -3.10416500 2.96469100

H 0 -5.94090700 -4.69520600 2.65599600

C 0 -4.08997600 -4.34707900 1.60841200

H 0 -4.26800400 -5.05236200 0.79109100

H 0 -3.53499500 -4.89868000 2.37727700

C 0 -3.15299400 -3.27233100 1.06235000

O 0 -3.47906800 -2.05178900 1.22463100

O 0 -2.08748800 -3.66695700 0.52088600

C 0 -6.33719900 -3.28036300 1.11341400

O 0 -6.20785700 -3.54783000 -0.08963000

N 0 -7.35668800 -2.51505700 1.56856900

H 0 -7.36341900 -2.23815000 2.54095800

C -1 -8.34351400 -1.90757400 0.68189900

H 0 -9.27725800 -1.77510500 1.22976800

H 0 -8.50580800 -2.58649800 -0.15902000

C 0 -7.88463000 -0.52116700 0.19201600

O 0 -8.34628100 0.51270000 0.67699500

N 0 -6.94476700 -0.55097200 -0.78434100

H 0 -6.54366700 -1.43921300 -1.06597100

C 0 -6.29925900 0.64196900 -1.28990100

H 0 -6.51772900 0.78745500 -2.35197100

H 0 -6.70030200 1.49564800 -0.73460200

C 0 -4.77479900 0.60634700 -1.13117300

O 0 -4.07642000 1.06123500 -2.05562900

O 0 -4.34998100 0.10840200 -0.02277500

C -1 -3.10223800 3.67583700 4.98364400

H -1 -3.13028200 3.37361800 6.02011700

H -1 -3.89975400 4.35010500 4.70849400

H 0 -2.17762200 4.26182500 4.87185000

C 0 -3.09748200 2.55697600 3.94264200

H 0 -3.18641300 3.02424400 2.95357900

H 0 -3.96821600 1.90072800 4.04784700

C 0 -1.81009600 1.72471600 3.97373300

H 0 -1.87602100 0.98761000 4.78256100

H 0 -0.94137400 2.35890200 4.17098000

C 0 -1.58186200 1.02610100 2.64698100

O 0 -0.45911400 1.20773500 2.08886600

O 0 -2.53264200 0.31619200 2.18744500

C -1 -5.03032800 9.17897300 2.06554500

H -1 -4.89568000 8.75706300 3.05056600

H -1 -6.05922100 9.45312000 1.88504300

H 0 -4.44172200 10.10331100 2.01865700

C 0 -4.52853900 8.16995700 1.02642500

H 0 -3.43905000 8.07882000 1.14081500

H 0 -4.69369600 8.54269400 0.00712100

C 0 -5.20184200 6.79869800 1.18608200

H 0 -6.26626500 6.88300700 0.93566900

H 0 -5.14791400 6.47740700 2.23540000

C 0 -4.59244400 5.69935500 0.31440200

H 0 -4.62765300 5.99061900 -0.74556000

H 0 -5.16521700 4.77302300 0.42977200

N 0 -3.21452500 5.43463100 0.72932200

H 0 -2.68377400 6.21284100 1.09524900

C 0 -2.49868100 4.35123000 0.36733400

N 0 -3.01757700 3.42810100 -0.44459300

H 0 -2.57295200 2.49694500 -0.50184300

H 0 -3.81708100 3.61333700 -1.02977500

N 0 -1.27197100 4.16963200 0.85559300

H 0 -0.90421000 4.69824600 1.63465700

H 0 -0.61404400 3.49648600 0.41334700

C -1 -0.79510100 -1.38821600 7.45578200

H -1 0.20409000 -1.02382600 7.26802200

H -1 -0.96746300 -1.59679700 8.50131700

H 0 -1.52340200 -0.64397000 7.11719300

C 0 -1.00549000 -2.75023200 6.76419200

H 0 -1.98691400 -3.15286200 7.05020400

H 0 -0.26456900 -3.46338300 7.14921900

C 0 -0.93178400 -2.74001400 5.27310000

N 0 -0.97363600 -3.91148300 4.52537400

C 0 -0.88463200 -1.73062700 4.35212100

H 0 -0.81191100 -0.66991000 4.49567000

C 0 -0.96224800 -3.62479700 3.21701900

H 0 -0.99232600 -4.32549500 2.39879100

N 0 -0.92259600 -2.30064100 3.09480800

H 0 -0.91895700 -1.79292000 2.17264400

C -1 4.13878300 6.02295100 2.27647000

H -1 5.01800700 6.49043600 1.85833400

H -1 3.70028000 6.55018100 3.11082100

H 0 3.37040300 6.00989700 1.49562100

C 0 4.46418400 4.55797500 2.65931600

H 0 5.41948300 4.48244100 3.18865300

H 0 3.66967600 4.16806200 3.30581700

C 0 4.46736400 3.70100100 1.39689700

O 0 3.36945600 3.62964200 0.78805900

O 0 5.54684500 3.14464900 1.00143200

C -1 12.15255600 -2.84442200 -0.88818600

H -1 13.20643300 -2.61699100 -0.95156800

H -1 11.89151800 -3.72178800 -1.46132100

H 0 11.96381700 -3.12438200 0.15885700

C 0 11.28496800 -1.61732400 -1.24548400

H 0 11.74010300 -1.06588200 -2.07951800

H 0 10.30130700 -1.94962600 -1.60349100

C 0 11.10363800 -0.67811500 -0.03689100

H 0 10.43711300 -1.16059000 0.69349400

H 0 12.06852500 -0.57010800 0.47609700

C 0 10.60672900 0.74132400 -0.35823000

H 0 10.55800200 1.33351600 0.56491600

H 0 11.34593700 1.23550100 -1.00183300

C 0 9.26988400 0.86505300 -1.08641700

H 0 9.08881600 1.90760000 -1.35838500

H 0 9.24105800 0.26832500 -2.00105900

N 0 8.07928800 0.45923800 -0.25841700

H 0 7.21193600 0.80792500 -0.73948500

H 0 8.02552800 -0.55258800 -0.12115100

H 0 8.05007700 0.93288200 0.68770500

O 0 2.03729700 1.44613100 -3.52876700

H 0 3.56069600 2.20660900 -3.67164500

O 0 0.55234800 2.47158100 -0.16216300

O 0 1.12656000 3.60867400 2.54866700

H 0 0.66713400 2.77430800 2.34192700

H 0 1.79527400 3.66855700 1.83214400

O 0 -0.09826500 -0.28001400 -4.31952800

H 0 0.61330700 0.38432100 -4.14472200

H 0 0.10545700 -1.03423800 -3.71725000

O 0 -3.38025100 -0.85611800 -4.18469500

H 0 -4.11540000 -1.18157000 -3.62927600

H 0 -2.81223100 -1.65452600 -4.29235400

O 0 -4.70050200 -2.40363800 -2.17976600

H 0 -3.84928500 -2.12844400 -1.75401400

H 0 -5.13599000 -2.96796000 -1.51299900

O 0 2.65170800 3.75163500 -1.72587500

H 0 3.02765500 3.64385000 -0.80225300

H 0 2.97058600 -0.46953600 -1.79661000

O 0 0.17347900 -1.82364400 -2.01054600

O 0 6.08767900 2.02288100 -1.34888000

H 0 5.67114000 2.40359900 -0.51560800

H 0 6.62989000 2.75846600 -1.68178300

O 0 4.38353100 2.74408500 -3.48669700

H 0 4.85446300 2.23585600 -2.79249900

H 0 3.39089500 3.54526800 -2.36971100

O 0 7.64756600 1.87919600 2.04688600

H 0 6.87673000 2.45180400 1.77240400

H 0 7.33976900 1.39960200 2.83120200

O 0 2.77051600 -1.19936300 -2.41137700

H 0 2.34550500 0.54416300 -3.29198800

H 0 1.82993700 -1.42294300 -2.18524500

H 0 -1.01079300 -4.84944000 4.90560900

**^8′′^TS_1_** (for the case with a net charge of zero on the OEC)

E (IEFPCM/B3LYP-D3/SDD,6-31G(d)) = -5393.895283 au

H (IEFPCM/B3LYP-D3/SDD,6-31G(d)) = -5392.34584 au

G (IEFPCM/B3LYP-D3/SDD,6-31G(d)) = -5392.577538 au

E (IEFPCM/B3LYP-D3/SDD,6-311+G(2d,p)//IEFPCM/B3LYP-D3/SDD,6-31G(d)) = -5395.676277 au

Mn 0 1.38829500 1.67713000 -2.09661500

Mn 0 -0.99882800 -2.49139200 -0.82256200

Mn 0 -0.10171900 0.85237500 0.23669000

Mn 0 -2.69323700 -0.34747000 0.39240500

O 0 0.23776200 0.06291500 -1.46442900

O 0 -1.87615600 1.08767400 -0.31597200

O 0 -2.55916600 -1.33305700 -1.09476300

O 0 -0.93267200 -0.95326300 0.82585100

Ca 0 -1.89167400 0.20301000 -2.74872000

C -1 0.77956500 4.20327500 -5.88052600

H -1 0.87554900 5.16509400 -5.39864000

H -1 0.96416500 4.21098600 -6.94436200

H 0 1.58302500 3.58897300 -5.45568300

C 0 -0.55620300 3.52576900 -5.53449800

H 0 -0.76595700 2.70935700 -6.23815400

H 0 -1.40734000 4.21262600 -5.61969900

C 0 -0.61352100 2.86945500 -4.14814800

O 0 -1.67541900 2.27602100 -3.81604400

O 0 0.43682900 2.93548700 -3.43100700

C -1 -3.01624800 -6.91023300 -4.93763800

H -1 -2.07112400 -7.41161600 -5.08515900

H -1 -3.75715700 -7.07605500 -5.70572100

H 0 -3.42587400 -7.32164400 -4.00482200

C 0 -2.65052600 -5.44055500 -4.72722800

H 0 -3.40323100 -4.92609600 -4.11564300

H 0 -2.58575100 -4.89602500 -5.67554100

C 0 -1.27352300 -5.40625700 -4.03293800

H 0 -0.49245200 -5.60836800 -4.77542000

H 0 -1.22544700 -6.17664100 -3.25696500

C 0 -1.05469600 -4.05507700 -3.40786800

O 0 -0.91639300 -3.06715800 -4.16892700

O 0 -1.12858500 -4.04313200 -2.12674600

C -1 5.07378900 -4.72998800 1.21887500

H 0 5.84595800 -5.46984200 1.46088200

H 0 5.36545100 -4.24036800 0.28355500

C 0 4.99211800 -3.73196700 2.36263800

O 0 4.48381900 -4.03924000 3.45063900

C 0 3.72510200 -5.48230600 1.02159500

H 0 3.54062800 -6.08145900 1.92127400

H 0 3.82512700 -6.17587200 0.18245900

C 0 2.55786400 -4.57054800 0.77850300

N 0 2.25973200 -3.57945200 1.70107500

H 0 2.70675700 -3.48773100 2.60731400

C 0 1.63476800 -4.39561200 -0.22825600

H 0 1.50355100 -4.94909200 -1.14570800

C 0 1.24041500 -2.84047800 1.23500700

H 0 0.86520100 -1.97875100 1.74655100

N 0 0.81144900 -3.31672900 0.07672300

N 0 5.42965400 -2.48024800 2.09063200

H 0 5.75658000 -2.29087600 1.15402200

C -1 5.15534000 -1.34584700 2.96699200

H 0 6.03528100 -0.69026000 2.97120600

H 0 5.03215900 -1.74573100 3.97478500

C 0 3.90555200 -0.55388400 2.54625400

H 0 3.70845200 0.22081100 3.29619500

H 0 3.03948200 -1.21772100 2.55827100

C 0 4.06130800 0.07959600 1.16192900

H 0 4.61483400 -0.57718100 0.48111400

H 0 4.64459300 1.00631700 1.21846600

C 0 2.78314100 0.42109500 0.41246200

O 0 2.92438600 0.66988100 -0.81554900

O 0 1.67664100 0.40261100 1.04946500

C -1 -5.39868100 -3.70328000 2.46747600

H 0 -5.22944900 -2.90371700 3.19534400

H 0 -5.89024000 -4.53122100 2.99276600

C 0 -4.06321300 -4.19950600 1.88568200

H 0 -4.23633200 -5.00810100 1.17033900

H 0 -3.44761100 -4.61812200 2.69059500

C 0 -3.21211500 -3.14321100 1.17368200

O 0 -3.54863900 -1.93058400 1.33079100

O 0 -2.20981500 -3.56051500 0.50989500

C 0 -6.33211300 -3.22124800 1.36098200

O 0 -6.21475400 -3.59599500 0.18631000

N 0 -7.33825100 -2.40539800 1.75624200

H 0 -7.32830700 -2.03617100 2.69736300

C -1 -8.32918100 -1.87548800 0.82762400

H 0 -9.26541300 -1.71246000 1.36359800

H 0 -8.48307700 -2.61794300 0.04091200

C 0 -7.88966300 -0.52415000 0.23481300

O 0 -8.32536000 0.53815500 0.68151400

N 0 -7.00692600 -0.61389100 -0.79063900

H 0 -6.58778400 -1.50957000 -1.02589800

C 0 -6.39437900 0.55074700 -1.39378000

H 0 -6.59103200 0.58389600 -2.46892100

H 0 -6.83786000 1.43662100 -0.92864300

C 0 -4.87527500 0.58852600 -1.20264900

O 0 -4.15463600 0.94424700 -2.14961200

O 0 -4.47234200 0.24493200 -0.02121600

C -1 -3.13445000 4.00617800 4.77366200

H -1 -3.16545200 3.77260800 5.82764800

H -1 -3.93413100 4.65668900 4.45160100

H 0 -2.19192200 4.54118200 4.59426100

C 0 -3.21753900 2.79255200 3.86213400

H 0 -3.19978900 3.15566800 2.82577300

H 0 -4.17509000 2.27341600 3.97856500

C 0 -2.06462900 1.79678800 4.06794000

H 0 -2.34905400 1.02202000 4.78638500

H 0 -1.16394900 2.29995000 4.43164400

C 0 -1.76856000 1.17748500 2.72903000

O 0 -0.64607000 1.42426000 2.19558000

O 0 -2.71770600 0.50009500 2.21269800

C -1 -5.07741300 9.29533600 1.49234500

H -1 -4.94490400 8.93981000 2.50350700

H -1 -6.10687400 9.55160000 1.29012100

H 0 -4.49744800 10.22066600 1.38954600

C 0 -4.55163600 8.22386700 0.52862400

H 0 -3.45530300 8.20368500 0.60643700

H 0 -4.77728800 8.48869700 -0.51272400

C 0 -5.12998400 6.83755100 0.84522700

H 0 -6.21715000 6.85357400 0.70006700

H 0 -4.95574300 6.59501600 1.90258000

C 0 -4.56030900 5.70812700 -0.01317500

H 0 -4.72666700 5.92450300 -1.07881900

H 0 -5.07475600 4.77112800 0.22596000

N 0 -3.13169800 5.52172400 0.25202000

H 0 -2.59503800 6.33681200 0.51645300

C 0 -2.42032000 4.45797200 -0.17165600

N 0 -2.99526700 3.49297500 -0.89202800

H 0 -2.59594300 2.54327600 -0.84434500

H 0 -3.91824700 3.59486000 -1.28324900

N 0 -1.11705800 4.36811500 0.11282300

H 0 -0.70965200 4.77558300 0.94920000

H 0 -0.55830100 3.58621000 -0.27316900

C -1 -0.81272300 -0.87217500 7.58261800

H -1 0.18543500 -0.51565700 7.37527200

H -1 -0.98854500 -1.01247500 8.63891400

H 0 -1.54070700 -0.15642300 7.18719300

C 0 -1.02529800 -2.27967800 6.99872800

H 0 -2.01183500 -2.65172800 7.30994500

H 0 -0.29261000 -2.96634700 7.44644100

C 0 -0.93137200 -2.34215600 5.51624400

N 0 -1.12314700 -3.51086800 4.80045500

C 0 -0.70093300 -1.38046800 4.56515000

H 0 -0.47554600 -0.33610500 4.70942900

C 0 -1.01293500 -3.22475500 3.47878100

H 0 -1.10192800 -3.95658900 2.69068500

N 0 -0.76636800 -1.93768500 3.30631500

H 0 -0.89069500 -1.23018700 1.80148200

C -1 4.10635500 6.20835400 1.94678100

H -1 4.98502800 6.65196400 1.50230200

H -1 3.66163300 6.78697800 2.74290500

H 0 3.34298600 6.15134800 1.16102900

C 0 4.40695500 4.76573300 2.42581500

H 0 5.34289300 4.72049700 2.99181000

H 0 3.58958000 4.43190000 3.07585400

C 0 4.45042200 3.78952900 1.24897800

O 0 3.37696900 3.62024600 0.61308400

O 0 5.55115700 3.20671200 0.95873900

C -1 12.17789000 -2.80554500 -0.59602800

H -1 13.23088000 -2.57722500 -0.67004200

H -1 11.92370900 -3.72005300 -1.11125700

H 0 11.97911000 -3.00459500 0.46737700

C 0 11.33494300 -1.59835400 -1.05043200

H 0 11.71911000 -1.21232700 -2.00474800

H 0 10.30140200 -1.91619400 -1.24461200

C 0 11.34808600 -0.47842800 0.00777000

H 0 10.75303600 -0.79879800 0.87629700

H 0 12.37186300 -0.36125500 0.38696300

C 0 10.88647300 0.90722600 -0.47511900

H 0 10.95767200 1.62737500 0.35030100

H 0 11.57497800 1.26015400 -1.25343000

C 0 9.48680700 0.99065700 -1.07861300

H 0 9.28679000 2.00284800 -1.43799800

H 0 9.36268400 0.30273900 -1.91854500

N 0 8.38916900 0.68242600 -0.09936200

H 0 7.45984900 0.90458700 -0.54912500

H 0 8.40927000 -0.29529500 0.19813100

H 0 8.39699100 1.29996900 0.75746800

O 0 2.08196500 0.73874800 -3.58679400

H 0 3.71852000 1.35741100 -3.69531800

O 0 0.55408800 2.31721500 -0.57108600

O 0 0.96632700 3.92069700 2.14796300

H 0 0.53341600 3.04828700 2.17188900

H 0 1.70510400 3.80432500 1.50978200

O 0 -0.31634300 -0.33615100 -4.57131500

H 0 0.55348300 0.07340900 -4.33191100

H 0 -0.22975100 -1.28924100 -4.35226800

O 0 -3.14170800 -1.44200500 -4.06316700

H 0 -3.81372400 -1.90124400 -3.51520500

H 0 -2.43391300 -2.11052800 -4.21847600

O 0 -4.82156300 -2.40394900 -1.97937000

H 0 -4.01434800 -2.04536200 -1.52238300

H 0 -5.14300100 -3.10936600 -1.38727000

O 0 2.87703800 3.26243600 -1.99849900

H 0 3.14820100 3.30612400 -1.04044300

H 0 2.87995700 -1.06621100 -1.65128700

O 0 -0.06639100 -1.57417700 -2.04885800

O 0 6.13314200 1.76696400 -1.17978200

H 0 5.75409900 2.29868800 -0.41032500

H 0 6.46239600 2.44493600 -1.79407100

O 0 4.59242200 1.78773700 -3.49721600

H 0 4.89353100 1.35887400 -2.67214800

H 0 3.64678400 2.90613800 -2.51420100

O 0 7.89903400 2.39774400 1.99997600

H 0 7.04384600 2.81697700 1.70835500

H 0 7.68096000 1.94844900 2.83133800

O 0 2.64305700 -1.72737300 -2.32855800

H 0 2.32834200 -0.16588200 -3.27552600

H 0 1.67051500 -1.83272800 -2.18440500

H 0 -1.31246200 -4.42139500 5.19751200

**^10^L_F** (for the case with a net charge of zero on the OEC)

E (IEFPCM/B3LYP-D3/SDD,6-31G(d)) = -5243.58936 au

H (IEFPCM/B3LYP-D3/SDD,6-31G(d)) = -5242.049487 au

G (IEFPCM/B3LYP-D3/SDD,6-31G(d)) = -5242.283752 au

E (IEFPCM/B3LYP-D3/SDD,6-311+G(2d,p)//IEFPCM/B3LYP-D3/SDD,6-31G(d)) = -5245.322121 au

Mn 0 1.34091000 2.09031700 -2.07491000

Mn 0 -0.81678700 -2.38721600 -0.71778000

Mn 0 -0.13295800 0.69020100 0.38916800

Mn 0 -2.74254500 -0.50596300 0.27548700

O 0 -1.84808700 0.92810100 -0.42155700

O 0 -2.25632800 -1.47637700 -1.35818400

O 0 -0.69518000 -1.18627600 0.69856700

Ca 0 -2.36914000 0.62346600 -2.74328600

C -1 0.70017400 4.55442100 -5.72510100

H -1 0.80767400 5.49261800 -5.20092300

H -1 0.85521600 4.61280000 -6.79193900

H 0 1.50184700 3.91050800 -5.34234300

C 0 -0.64445000 3.88521400 -5.39800700

H 0 -0.82832300 3.03774800 -6.07116800

H 0 -1.49104100 4.56810800 -5.54663400

C 0 -0.73567100 3.31460300 -3.97835900

O 0 -1.78437400 2.67988900 -3.65781800

O 0 0.25970900 3.49424200 -3.21234700

C -1 -3.04451200 -6.60411600 -5.20313100

H -1 -2.10284000 -7.09479600 -5.40032200

H -1 -3.80619500 -6.73608100 -5.95735800

H 0 -3.44731300 -7.08811700 -4.29985500

C 0 -2.76047800 -5.12180900 -4.88638800

H 0 -3.69486900 -4.57039300 -4.72553300

H 0 -2.25851700 -4.64404900 -5.73555600

C 0 -1.88229600 -4.97820700 -3.63409700

H 0 -1.04792800 -5.69501900 -3.66000300

H 0 -2.44906900 -5.21719600 -2.72550600

C 0 -1.25043400 -3.61150600 -3.42154600

O 0 -1.36838100 -2.70917700 -4.26492800

O 0 -0.53195100 -3.49636700 -2.33398100

C -1 5.20930700 -4.68913300 0.82281500

H 0 6.03321800 -5.39994600 0.95965700

H 0 5.39485000 -4.14463600 -0.10890300

C 0 5.18501700 -3.75134000 2.01879200

O 0 4.77080500 -4.12832000 3.12582600

C 0 3.88461400 -5.49522000 0.70585500

H 0 3.78957500 -6.12715700 1.59625700

H 0 3.94108200 -6.15523700 -0.16348400

C 0 2.68770100 -4.60233600 0.58172500

N 0 2.43143200 -3.66794000 1.57517100

H 0 2.93541400 -3.62080300 2.45593700

C 0 1.74562400 -4.35091600 -0.38792700

H 0 1.57613000 -4.84137300 -1.33345500

C 0 1.42535600 -2.87620200 1.18334600

H 0 1.09626600 -2.00278900 1.70857900

N 0 0.96219900 -3.27075000 0.00368400

N 0 5.55649900 -2.47554800 1.77531500

H 0 5.80604600 -2.23237000 0.82728000

C -1 5.33242300 -1.39087200 2.72589900

H 0 6.19691400 -0.71634700 2.68217800

H 0 5.30825500 -1.83797300 3.72119100

C 0 4.04486800 -0.61109700 2.45278400

H 0 3.93001000 0.15945200 3.22218900

H 0 3.19395000 -1.28472100 2.57260000

C 0 4.02054800 0.02673000 1.05278000

H 0 4.64623300 -0.51894300 0.33968800

H 0 4.42236300 1.04702600 1.08881800

C 0 2.63297600 0.09450200 0.45049100

O 0 2.49592500 -0.05098500 -0.79716200

O 0 1.65984100 0.29666800 1.24269700

C -1 -5.22639300 -3.75803200 2.40837200

H 0 -4.94021500 -2.96220400 3.10235400

H 0 -5.77553800 -4.51532800 2.98278100

C 0 -3.99509400 -4.38867700 1.76491500

H 0 -4.28569500 -5.16115700 1.04647800

H 0 -3.37797200 -4.88353200 2.52355700

C 0 -3.12928700 -3.37612700 1.02583100

O 0 -3.51871200 -2.16849400 1.08513200

O 0 -2.08666100 -3.79532800 0.44559600

C 0 -6.17932000 -3.20544100 1.34578400

O 0 -6.14832700 -3.58000400 0.16202500

N 0 -7.11749700 -2.33918600 1.78721800

H 0 -7.05858300 -1.98389800 2.73150100

C -1 -8.20537400 -1.86496400 0.93855200

H 0 -9.04345300 -1.58942700 1.58071300

H 0 -8.50889900 -2.68856800 0.28621600

C 0 -7.83429900 -0.62941300 0.10697300

O 0 -8.13395300 0.50764400 0.46835000

N 0 -7.19687200 -0.91729500 -1.05784400

H 0 -6.78950200 -1.84396100 -1.12899100

C 0 -6.57946600 0.13079500 -1.83730700

H 0 -6.49867900 -0.18336800 -2.88178900

H 0 -7.21615900 1.01856700 -1.80437400

C 0 -5.17436100 0.51753600 -1.35703800

O 0 -4.52203400 1.29199100 -2.11511000

O 0 -4.76119400 0.02061500 -0.27131100

C -1 -2.91467800 3.84212000 5.01250300

H -1 -2.91633500 3.55847900 6.05464700

H -1 -3.72485900 4.50382800 4.74378600

H 0 -1.95775000 4.32644300 4.78794600

C 0 -3.18279800 2.67429800 4.04729800

H 0 -3.08564100 3.07138100 3.02817800

H 0 -4.22826500 2.35650400 4.13686900

C 0 -2.27261700 1.44297100 4.19984200

H 0 -2.76032500 0.67143700 4.80513900

H 0 -1.33116900 1.72425000 4.68197700

C 0 -1.95269800 0.90952200 2.81655500

O 0 -0.79086400 1.14206000 2.38352900

O 0 -2.88031300 0.30661200 2.17987600

C -1 -4.95960600 9.27319600 2.03973000

H -1 -4.79838200 8.87072400 3.02892400

H -1 -5.99495700 9.53500000 1.87847100

H 0 -4.38038800 10.20107800 1.96027600

C 0 -4.46935800 8.24217700 1.00674400

H 0 -3.37111100 8.21382800 1.04086000

H 0 -4.74131200 8.55802600 -0.00965100

C 0 -5.03050500 6.83939300 1.28282000

H 0 -6.12778800 6.88206400 1.28470000

H 0 -4.72409800 6.51417400 2.28637600

C 0 -4.61975000 5.76122800 0.27261300

H 0 -4.97856700 6.02405000 -0.73258300

H 0 -5.08714600 4.81452800 0.55575400

N 0 -3.17132700 5.55259100 0.24853800

H 0 -2.58837700 6.37470600 0.16959800

C 0 -2.55285800 4.36973200 0.04086100

N 0 -3.25461300 3.25019000 -0.17622600

H 0 -2.72935600 2.35499400 -0.23901900

H 0 -4.13372500 3.27060800 -0.67450700

N 0 -1.22273300 4.29921100 0.08988800

H 0 -0.65786500 4.96763800 0.60079100

H 0 -0.71351900 3.42326600 -0.15863300

C -1 -0.50566500 -1.15567000 7.52231200

H -1 0.48552600 -0.78643800 7.30463800

H -1 -0.65186900 -1.34627000 8.57536100

H 0 -1.24009400 -0.41183500 7.19519900

C 0 -0.74128800 -2.51475800 6.83636400

H 0 -1.70405800 -2.92384200 7.17275200

H 0 0.02208700 -3.22654600 7.17906600

C 0 -0.75040600 -2.50608400 5.34084400

N 0 -0.93561500 -3.68002200 4.61901900

C 0 -0.65969700 -1.51694800 4.39724600

H 0 -0.48070500 -0.46230000 4.50674700

C 0 -0.96627900 -3.40981900 3.30593400

H 0 -1.07981400 -4.12036400 2.50291200

N 0 -0.81620500 -2.09929600 3.15241900

H 0 -0.80687700 -1.63034400 2.17125900

C -1 4.23893900 6.19916700 2.09302600

H -1 5.10465700 6.66641400 1.64586100

H -1 3.81644300 6.73853700 2.92774400

H 0 3.46194100 6.21675400 1.32105900

C 0 4.56727000 4.72565400 2.40219800

H 0 5.46744300 4.62129400 3.01657600

H 0 3.73375900 4.26777400 2.95183800

C 0 4.72419700 3.89983500 1.11693400

O 0 3.88971000 4.13019800 0.19814100

O 0 5.63281400 3.01226800 1.04098000

C -1 12.25597900 -2.65703100 -1.09539800

H -1 13.30606000 -2.42187800 -1.18776900

H -1 11.98956300 -3.54708800 -1.64605000

H 0 12.09219100 -2.91040800 -0.03729600

C 0 11.39313300 -1.43551900 -1.45773000

H 0 11.71770300 -1.01631900 -2.42005100

H 0 10.34913200 -1.74883000 -1.59845400

C 0 11.47425700 -0.35447100 -0.36406800

H 0 10.96994300 -0.72449200 0.54123600

H 0 12.52515200 -0.22104600 -0.07476900

C 0 10.92635900 1.03050400 -0.74728700

H 0 11.02945400 1.71311600 0.10626100

H 0 11.54132400 1.44850600 -1.55465900

C 0 9.48410900 1.07388400 -1.24388900

H 0 9.19291300 2.09192100 -1.51083600

H 0 9.34048900 0.44519300 -2.12637200

N 0 8.47644100 0.62437800 -0.22686700

H 0 7.51103100 0.83915000 -0.62928900

H 0 8.56946300 -0.36812900 -0.00377600

H 0 8.50033700 1.17822200 0.66803400

O 0 1.73202000 0.76095500 -3.57233300

H 0 3.55794200 0.70631100 -3.35535500

O 0 0.48121500 2.20498900 -0.30662800

O 0 1.35475800 4.13048900 1.39185000

H 0 1.06320600 3.32893500 0.88981900

H 0 2.22370200 4.31063000 0.97899200

O 0 -0.68022800 -0.06796800 -4.18910100

H 0 0.25200200 0.31138900 -4.07929800

H 0 -0.62314100 -1.04865900 -4.21353900

O 0 -3.65758400 -1.05608800 -4.02706300

H 0 -4.18951100 -1.60591900 -3.40649200

H 0 -2.95840600 -1.67912000 -4.31901500

O 0 -4.40086000 -2.88579700 -2.07643400

H 0 -3.58373300 -2.44925900 -1.70968400

H 0 -4.94113500 -3.10210600 -1.29279000

O 0 3.27737200 3.10130300 -2.13521400

H 0 3.58665500 3.46490300 -1.25281200

H 0 2.02784300 -1.60902200 -1.83551100

O 0 6.33676400 1.75295400 -1.26256300

H 0 5.90515600 2.23721700 -0.51003700

H 0 5.64182100 1.35815200 -1.83203800

O 0 4.31234700 0.56269400 -2.72199700

H 0 3.80490000 0.21656500 -1.94738800

H 0 3.89183900 2.36962800 -2.35969200

O 0 8.00713200 2.18468600 2.01266400

H 0 7.13614200 2.59565000 1.76225600

H 0 7.82643400 1.70340400 2.83458600

O 0 1.95628500 -2.00404300 -2.72583200

H 0 1.81740800 -0.17213200 -3.28975600

H 0 1.13946900 -2.54022300 -2.69966600

H 0 -1.03235400 -4.60436200 5.02068300

**^8′^L_S_4_** (for an OEC model incorporating Y_z_ and His190)

E (IEFPCM/B3LYP-D3/SDD,6-31G(d)) = -5382.895247 au

H (IEFPCM/B3LYP-D3/SDD,6-31G(d)) = -5381.360489 au

G (IEFPCM/B3LYP-D3/SDD,6-31G(d)) = -5381.594415 au

E (IEFPCM/B3LYP-D3/SDD,6-311+G(2d,p)//IEFPCM/B3LYP-D3/SDD,6-31G(d)) = -5384.589366 au

C -1 7.88262800 5.45327600 0.15982900

C 0 7.16442300 5.06271800 -1.12793800

C 0 5.64404800 5.25568800 -1.04538000

O 0 5.12921900 5.21384200 0.11832400

O 0 5.02370700 5.41782300 -2.13526600

C -1 -7.57456100 7.46034000 1.50156900

C 0 -6.88565700 6.26457900 0.87427100

C 0 -7.49963600 5.00481100 0.85353900

C 0 -5.62572500 6.37637000 0.26682800

C 0 -6.89409400 3.89826800 0.25537900

C 0 -5.00970900 5.28376500 -0.34654000

C 0 -5.63933600 4.03305300 -0.35564200

O 0 -4.98754800 2.98106600 -0.94890400

C -1 -0.70657400 6.82538000 0.22963300

C 0 -1.36989700 5.74716000 1.10567600

C 0 -0.62490500 4.40704200 1.08134600

O 0 0.63428100 4.44862500 0.98278100

O 0 -1.29868500 3.33551500 1.16290000

C 0 -6.25907900 -4.09807400 -1.56481000

C 0 -7.68045700 -3.65009200 -1.29334500

O 0 -8.09561600 -3.49860700 -0.13333100

C 0 -5.29106400 -2.93212500 -1.26710900

C 0 -3.83789100 -3.34102700 -1.48241300

C 0 -2.81230300 -2.25479400 -1.13230600

O 0 -3.14925700 -1.18704300 -0.61437600

O 0 -1.60455200 -2.64890800 -1.43201600

N 0 -8.43098000 -3.33371200 -2.38082500

C 0 -9.72053300 -2.65101700 -2.28903300

C 0 -9.58531700 -1.14219400 -2.61958200

C 0 -8.45594600 -0.42806100 -1.93216200

N 0 -8.02856400 -0.69931900 -0.64175200

C 0 -7.65657200 0.61031100 -2.35250000

C 0 -6.99811300 0.13484000 -0.35410700

N 0 -6.75598000 0.95606200 -1.36355800

C -1 3.36948500 -2.02596900 -6.85264600

C 0 4.48675800 -1.96018900 -5.81214000

O 0 4.66499300 -2.87701400 -4.99763300

C 0 2.05627100 -1.44080000 -6.27295600

C 0 1.68843100 -1.94631800 -4.90990700

N 0 1.82380200 -3.26919700 -4.50896500

C 0 1.12393200 -1.30537600 -3.83746500

C 0 1.37647700 -3.38179100 -3.23650400

N 0 0.92975000 -2.21018000 -2.81254400

N 0 5.21572200 -0.81790600 -5.79698200

C -1 6.11384800 -0.48031800 -4.69008100

C 0 5.34485800 -0.01711400 -3.43679500

C 0 4.48281800 1.22822500 -3.65541200

C 0 3.59066600 1.63646200 -2.47407500

O 0 3.45865100 0.76367900 -1.53777200

O 0 3.04981300 2.76019400 -2.52321300

C -1 7.68828000 -4.88125900 1.32444400

C 0 6.17812100 -5.20207100 1.19729100

C 0 5.33598800 -4.23727900 0.40560100

N 0 5.50380400 -3.98585100 -0.95350200

C 0 4.23401500 -3.50159500 0.76303600

C 0 4.55281800 -3.13872000 -1.38941200

N 0 3.77492400 -2.83063400 -0.35659000

C -1 0.65411600 -5.84731900 2.53305800

C 0 -0.60419600 -5.45100700 3.28383800

O 0 -1.72478200 -5.49625300 2.77875800

C 0 0.61933700 -5.48659400 1.05570800

C 0 0.79495500 -4.00359100 0.75761600

O 0 1.08382000 -3.22170500 1.71871000

O 0 0.65233500 -3.66395600 -0.44567700

N 0 -0.39705100 -5.11190400 4.58951400

C -1 -1.47539400 -4.86628700 5.51991900

C 0 -1.84876100 -3.39520800 5.74709000

O 0 -2.44028200 -3.05197500 6.77406500

N 0 -1.51705200 -2.56365600 4.74347200

C 0 -1.67785700 -1.12926800 4.80748000

C 0 -0.64004000 -0.42532100 3.92693400

O 0 -0.67424700 0.81665200 3.87216100

O 0 0.17500600 -1.20921500 3.32369100

C -1 6.20729600 -0.63409800 5.23860800

C 0 4.91992600 -0.45194300 4.42587900

C 0 5.12724800 -0.78274700 2.93889400

C 0 3.86349200 -0.59765600 2.11103700

O 0 2.84298700 -1.27689400 2.46775900

O 0 3.91165700 0.18597100 1.12709700

N 0 2.40893600 4.08007000 4.89124900

C -1 1.53096100 3.09972300 4.55155000

N 0 0.60765700 2.71407500 5.44566000

N 0 1.60669700 2.51352900 3.36801800

O 0 -0.56648800 -1.27260300 0.70436800

Ca 0 -1.12297600 1.03798300 0.05039300

Mn 0 0.00406200 -1.69796900 -1.01112300

O 0 1.10766300 0.63496000 1.47967700

Mn 0 1.02412300 -1.14140700 1.52823400

O 0 1.74762800 -1.10026000 -0.18424700

Mn 0 2.27530900 0.94950300 0.05580100

O 0 2.80774000 2.57972100 0.49119900

Mn 0 1.66168700 3.48206600 -0.72167800

O 0 0.97236900 1.74976400 -0.84210400

O 0 -0.51402600 -0.27782300 -1.71987100

O 0 2.75131600 5.20189500 -0.82235100

O 0 0.35956200 4.11580800 -2.00258200

O 0 -3.34413900 1.71938500 1.00596200

O 0 -1.84287700 2.58905200 -1.70713300

H 0 2.94524800 -2.13835900 -0.35475000

H 0 4.44878500 -2.79936000 -2.41044100

H 0 3.72602200 -3.39420200 1.70767900

H 0 8.17963200 -4.86383400 0.34516800

H 0 7.84262100 -3.90764000 1.80090300

H 0 8.17358400 -5.64969800 1.93327200

H 0 6.06287800 -6.20229100 0.75806000

H 0 5.73516800 -5.25564700 2.19751100

H 0 1.40590900 -6.02422000 0.51210300

H 0 -0.33494200 -5.79205800 0.61564900

H 0 0.73720900 -6.93838600 2.63498600

H 0 1.54146400 -5.41427100 3.00558500

H 0 -2.36588500 -5.37870300 5.14285300

H 0 -1.23089300 -5.28939600 6.49803500

H 0 0.55182100 -5.01224900 4.92157300

H 0 -2.67020900 -0.81357000 4.46064700

H 0 -1.57763400 -0.78795500 5.84327000

H 0 3.00312200 4.44294900 4.15667200

H 0 -0.04246300 1.97707300 5.16518500

H 0 0.72303800 2.93500900 6.42340300

H 0 1.00123500 1.72036700 3.12668700

H 0 2.09258700 2.90659900 2.55885900

H 0 3.53223700 5.19266900 -0.17222900

H 0 3.32882500 5.23906500 -1.63896300

H 0 7.73535000 6.51672600 0.38299400

H 0 7.48290400 4.88530300 1.00343700

H 0 8.96263800 5.26970600 0.09066500

H 0 7.54062900 5.62565300 -1.99005000

H 0 7.34127900 4.00069500 -1.35306700

H 0 7.00480900 0.01991000 4.86534200

H 0 6.04551900 -0.39694300 6.29639800

H 0 6.56799600 -1.66887400 5.17670700

H 0 4.12421800 -1.09227900 4.82426200

H 0 4.56458500 0.58322000 4.51642000

H 0 5.44283300 -1.83064000 2.84045100

H 0 5.91506300 -0.15498600 2.51164100

H 0 -3.59414400 -4.21914600 -0.86869900

H 0 -3.66600600 -3.64496200 -2.52304200

H 0 -5.53497700 -2.07186500 -1.90249800

H 0 -5.43152400 -2.60343400 -0.23322800

H 0 -6.13386200 -4.44393700 -2.59835000

H 0 -6.03789900 -4.93860200 -0.89826100

H 0 -10.02580200 -2.75768100 -1.24501400

H 0 -9.44832600 -1.01944900 -3.70121800

H 0 -10.54620500 -0.66686500 -2.37668600

H 0 -6.45300500 0.10316800 0.57803700

H 0 -7.68100500 1.12935400 -3.30056800

H 0 -8.27837200 -1.52946400 -0.10425100

H 0 -8.19415200 7.99579100 0.76923200

H 0 -8.23243400 7.15623600 2.32324300

H 0 -6.84782100 8.17840000 1.89758900

H 0 -5.63916300 2.23724500 -1.17282500

H 0 -4.04295500 5.38967400 -0.83029700

H 0 -5.11457300 7.33688600 0.26610600

H 0 -8.47340500 4.87878400 1.32287900

H 0 -7.38717500 2.93235700 0.26389100

H 0 -3.96022600 2.00395800 0.29857100

H 0 -2.87920800 2.55219200 1.25512600

H 0 -2.61424000 3.12854600 -1.46953900

H 0 -1.05482300 3.21408100 -1.79211800

H 0 0.64520100 3.70622600 -2.83798700

H 0 5.09561000 2.09976500 -3.91294000

H 0 3.80098500 1.07029800 -4.50232800

H 0 4.69544200 -0.82958500 -3.10530200

H 0 6.06262700 0.16259300 -2.62747900

H 0 6.79535100 0.29905200 -5.04484300

H 0 6.70528800 -1.37009400 -4.45751600

H 0 4.96385400 -0.08144500 -6.44199900

H 0 3.62937300 -1.48334000 -7.76745000

H 0 3.23381800 -3.07861600 -7.11890000

H 0 2.13667300 -0.35068800 -6.20764000

H 0 1.24957700 -1.64931100 -6.98800700

H 0 1.37671100 -4.29036800 -2.65643500

H 0 2.37382700 -3.96561700 -4.99252000

H 0 0.82935200 -0.27601100 -3.70528200

H 0 -0.56648300 6.45641600 -0.79131900

H 0 -1.32603300 7.73013200 0.19882100

H 0 0.27973800 7.09226000 0.61847200

H 0 -2.40625400 5.56832900 0.80262800

H 0 -1.41102300 6.08523000 2.15166400

H 0 2.10656400 4.76889000 5.56719000

C -1 -10.76425700 -3.33441500 -3.18353700

H 0 -10.89426200 -4.37991100 -2.88866800

H 0 -11.72995200 -2.82453300 -3.10107300

H 0 -10.45554700 -3.30602200 -4.23596100

H 0 6.22932200 -4.37443400 -1.54241500

H 0 -8.00143300 -3.40041100 -3.29494300

H 0 -0.94783600 -2.89938200 3.97484300

**^8′′^TS_1_** (for an OEC model incorporating Y_z_ and His190)

E (IEFPCM/B3LYP-D3/SDD,6-31G(d)) = -5382.870965 au

H (IEFPCM/B3LYP-D3/SDD,6-31G(d)) = -5381.337971 au

G (IEFPCM/B3LYP-D3/SDD,6-31G(d)) = -5381.57618 au

E (IEFPCM/B3LYP-D3/SDD,6-311+G(2d,p)//IEFPCM/B3LYP-D3/SDD,6-31G(d)) = -5384.560865 au

C -1 7.60079700 6.03933700 0.61456400

C 0 6.99818300 4.90918900 -0.22958800

C 0 5.48873000 5.05595300 -0.53305700

O 0 4.76592100 5.62541100 0.33114800

O 0 5.09292900 4.55726500 -1.63678300

C -1 -7.96780400 6.87982100 1.90417500

C 0 -7.19748700 5.76402400 1.23804100

C 0 -7.73404400 5.04866700 0.16010800

C 0 -5.92847900 5.38724000 1.70035300

C 0 -7.04580500 3.99006100 -0.43676200

C 0 -5.23122600 4.32821400 1.12410100

C 0 -5.78777800 3.61941400 0.05324500

O 0 -5.05960700 2.58579000 -0.48186700

C -1 -1.06301900 6.81100000 0.67562400

C 0 -1.66193800 5.55329200 1.31550900

C 0 -0.93311200 4.25868500 0.94918000

O 0 0.17406500 4.41067600 0.34880000

O 0 -1.45717500 3.15038100 1.24939300

C 0 -6.32373300 -3.96222700 -0.89142800

C 0 -7.78552900 -3.57376700 -1.00073000

O 0 -8.45608100 -3.26299000 -0.00524300

C 0 -5.41697200 -2.70811800 -0.93864300

C 0 -3.94156500 -3.11257200 -0.90373100

C 0 -2.91564400 -1.97790500 -1.00836900

O 0 -3.13918000 -0.87713200 -0.44615800

O 0 -1.85283700 -2.29494500 -1.64767800

N 0 -8.28444900 -3.51342500 -2.26671800

C 0 -9.56173600 -2.88130400 -2.59518600

C 0 -9.34454400 -1.44001000 -3.12867400

C 0 -8.42007400 -0.59193300 -2.30228300

N 0 -8.41133800 -0.58662600 -0.91602300

C 0 -7.45957400 0.32730000 -2.66012700

C 0 -7.45276900 0.27976100 -0.50425900

N 0 -6.86655100 0.86685500 -1.53426800

C -1 3.66914500 -1.20331600 -6.97270800

C 0 4.84100400 -1.14421000 -5.99267700

O 0 5.16867200 -2.13922400 -5.32737700

C 0 2.36410000 -0.70789300 -6.28996900

C 0 2.02848400 -1.31120600 -4.94820200

N 0 2.50047700 -2.53566400 -4.49056200

C 0 1.17046400 -0.87391700 -3.96825700

C 0 1.94678500 -2.78567300 -3.28144100

N 0 1.12230100 -1.80356800 -2.94562200

N 0 5.43278200 0.06394700 -5.83074300

C -1 6.28362300 0.36582400 -4.67071700

C 0 5.44755500 0.52021000 -3.38284300

C 0 4.49081500 1.71558800 -3.41736200

C 0 3.41794800 1.75033700 -2.33386800

O 0 3.50706800 0.93135800 -1.36531700

O 0 2.51818600 2.62710200 -2.48335900

C -1 8.11393000 -4.34136700 1.03136700

C 0 6.72257400 -4.98492900 0.87417000

C 0 5.73699800 -4.10633500 0.16878300

N 0 5.91973100 -3.66148400 -1.13177600

C 0 4.54798500 -3.53895900 0.56334600

C 0 4.87114500 -2.86112400 -1.46365800

N 0 4.02138100 -2.76885200 -0.45691400

C -1 1.15473900 -5.87232800 2.07967100

C 0 -0.11205600 -5.64069200 2.87010700

O 0 -1.22893500 -5.59646600 2.33785900

C 0 1.03203700 -5.42529600 0.62322500

C 0 0.90927700 -3.91623000 0.47126700

O 0 1.18489000 -3.22506400 1.49172400

O 0 0.56685400 -3.48121500 -0.67251000

N 0 0.05518900 -5.53941500 4.21272400

C -1 -1.05989600 -5.25864600 5.10247200

C 0 -1.42820700 -3.75261000 5.11093400

O 0 -1.32389200 -3.06723600 6.12966600

N 0 -1.85896400 -3.25786300 3.92330700

C 0 -2.10702500 -1.84113200 3.73973800

C 0 -0.86550000 -0.93099800 3.79638900

O 0 -1.02170000 0.22824000 4.20400500

O 0 0.25521900 -1.44270200 3.39350200

C -1 6.31316700 -0.50074300 5.22175000

C 0 5.07859900 -0.24708400 4.36688200

C 0 5.17716100 -0.97380100 3.01661900

C 0 3.93143800 -0.69035800 2.20698300

O 0 2.91560100 -1.41339200 2.49122000

O 0 3.97858100 0.25055600 1.36935100

N 0 2.00873700 3.99028100 5.34793000

C -1 1.39480100 2.94369700 4.74646400

N 0 0.65467900 2.09839200 5.46928300

N 0 1.53489300 2.72942100 3.44008800

O 0 -0.46602400 -1.20773100 0.73841800

Ca 0 -1.48918800 0.85263300 0.22277600

Mn 0 0.06588400 -1.55665200 -1.12498200

O 0 1.21145400 0.58545300 1.75777000

Mn 0 1.06894800 -1.18807700 1.65786500

O 0 1.95312000 -1.05120500 -0.03778900

Mn 0 2.28738900 0.93558700 0.25091900

O 0 2.37027000 2.71106800 0.40702800

Mn 0 1.26172800 3.31804400 -0.94454400

O 0 0.68044600 1.25472300 -0.78807900

O 0 -0.34091900 0.11092300 -1.67070800

O 0 2.53326900 5.12653700 -1.22931900

O 0 -0.06842300 3.69330700 -2.25252700

O 0 -3.48675000 1.42279900 1.57812700

O 0 -2.30273800 2.30743800 -1.53978200

H 0 2.74335300 -1.66504400 -0.17018400

H 0 4.77568300 -2.39038800 -2.43145300

H 0 4.03839800 -3.61346800 1.51285400

H 0 8.56222000 -4.11535400 0.05689100

H 0 8.04494100 -3.40406900 1.59393300

H 0 8.79239900 -5.01668000 1.56357800

H 0 6.82265100 -5.94055900 0.34027800

H 0 6.31131600 -5.22328700 1.86127100

H 0 1.91983200 -5.73765800 0.05927300

H 0 0.16428100 -5.88702900 0.14501000

H 0 1.36307800 -6.95103900 2.10637400

H 0 2.00169300 -5.37237800 2.55829800

H 0 -1.91791700 -5.85284400 4.77520100

H 0 -0.78912800 -5.53908700 6.12072400

H 0 0.98822000 -5.38562700 4.57066800

H 0 -2.54199500 -1.69933100 2.74457000

H 0 -2.81969300 -1.48082600 4.48522100

H 0 2.50659500 4.65470800 4.77136000

H 0 0.12523800 1.34824700 4.99813000

H 0 0.69083500 2.11637700 6.47748900

H 0 1.28355100 1.83338800 2.98017500

H 0 1.98309100 3.38690700 2.81164500

H 0 2.93875600 5.41508000 -0.37772000

H 0 3.39304700 4.80266500 -1.64101500

H 0 7.55050800 6.99745000 0.08264400

H 0 7.04114600 6.15203300 1.54805400

H 0 8.65234200 5.84127000 0.85569600

H 0 7.52984500 4.80386800 -1.18225600

H 0 7.11939900 3.95092800 0.29743200

H 0 7.22128700 -0.14911000 4.71620100

H 0 6.24793500 0.01493500 6.18734600

H 0 6.43878900 -1.57228600 5.42312300

H 0 4.17617300 -0.58702600 4.89024900

H 0 4.95632900 0.82881700 4.18694300

H 0 5.26718900 -2.05305400 3.18043900

H 0 6.05545400 -0.62654400 2.46323200

H 0 -3.72490600 -3.63065300 0.04271700

H 0 -3.72716600 -3.83101600 -1.70200700

H 0 -5.62733600 -2.13746800 -1.85221200

H 0 -5.65140000 -2.04834500 -0.09750600

H 0 -6.04246400 -4.65026700 -1.69877100

H 0 -6.18494700 -4.48531200 0.05984100

H 0 -10.10792700 -2.83073600 -1.64965600

H 0 -8.93486000 -1.48867500 -4.14508700

H 0 -10.33194900 -0.96514600 -3.21590600

H 0 -7.21307300 0.44587700 0.53632000

H 0 -7.16655300 0.63306100 -3.65476100

H 0 -8.83113400 -1.30640600 -0.33054700

H 0 -7.30131300 7.68134000 2.24266300

H 0 -8.70613400 7.31965100 1.22472800

H 0 -8.51232600 6.51881200 2.78745100

H 0 -5.65507100 1.98371700 -1.03512300

H 0 -4.25421800 4.04364900 1.49606300

H 0 -5.47705300 5.92658200 2.53034300

H 0 -8.71247000 5.32380500 -0.22827100

H 0 -7.47920000 3.45026700 -1.27170500

H 0 -4.14066100 1.66618300 0.88593800

H 0 -3.03780700 2.26803000 1.79063300

H 0 -3.05208500 2.87348400 -1.28921800

H 0 -1.52862500 2.89179600 -1.79825700

H 0 0.20288800 3.19604400 -3.04324800

H 0 5.03559800 2.66147000 -3.30183300

H 0 3.96156700 1.77048300 -4.37611000

H 0 4.87321500 -0.39862100 -3.23689900

H 0 6.12227000 0.60615000 -2.52370500

H 0 6.84188000 1.27864400 -4.89794300

H 0 6.99717100 -0.45420900 -4.55857500

H 0 5.06744600 0.84445100 -6.35954500

H 0 3.85048800 -0.59060600 -7.86179500

H 0 3.56480600 -2.24334600 -7.29360000

H 0 2.41466400 0.37892800 -6.16019100

H 0 1.53842400 -0.89139100 -6.98954600

H 0 2.17062100 -3.64147700 -2.66542000

H 0 3.31331800 -3.00645800 -4.87944800

H 0 0.58989100 0.03255700 -3.90176500

H 0 -1.04607300 6.71747000 -0.41473900

H 0 -1.65623000 7.69428600 0.94085700

H 0 -0.03292900 6.97139100 1.00655500

H 0 -2.70685600 5.43078000 1.01119800

H 0 -1.67644400 5.63062500 2.41154000

H 0 1.61330000 4.36218400 6.20038300

C -1 -10.36714800 -3.73991100 -3.58360400

H 0 -10.56119400 -4.72932400 -3.15906000

H 0 -11.32551200 -3.26074900 -3.80975200

H 0 -9.82087500 -3.86705000 -4.52631200

H 0 6.70463300 -3.88019800 -1.72996800

H 0 -7.64863200 -3.69279900 -3.03432400

H 0 -1.83186800 -3.86858200 3.11232200

**Ha_S_3_**

E (IEFPCM/B3LYP-D3/SDD,6-31G(d)) = -5854.138026 au

H (IEFPCM/B3LYP-D3/SDD,6-31G(d)) = -5852.584123 au

G (IEFPCM/B3LYP-D3/SDD,6-31G(d)) = -5852.822178 au

E (IEFPCM/B3LYP-D3/SDD,6-311+G(2d,p)//IEFPCM/B3LYP-D3/SDD,6-31G(d)) = -5855.955292 au

Mn 0 1.47674500 2.10983100 -1.81280700

Mn 0 -1.02582700 -2.52026900 -0.63165900

Mn 0 -0.12299100 0.85307500 0.03261400

Mn 0 -2.66899700 -0.39320000 0.16850700

O 0 0.45292100 0.52769800 -1.61114600

O 0 -1.87733000 1.02345100 -0.53114400

O 0 -2.41655900 -1.48756200 -1.27858200

O 0 -0.96865600 -1.07545300 0.62082000

Ca 0 -1.10655700 -0.46118600 -3.16167400

C -1 0.69595400 4.56534200 -5.60402400

H -1 0.76422100 5.50185800 -5.07035500

H -1 0.91160800 4.63080800 -6.66000400

H 0 1.56180000 4.00393100 -5.21867700

C 0 -0.56570300 3.75078700 -5.23075000

H 0 -0.92874500 3.16776100 -6.08142200

H 0 -1.38985300 4.41411000 -4.93596800

C 0 -0.37593900 2.73447500 -4.08076400

O 0 -0.94941500 1.62931700 -4.17681700

O 0 0.34755600 3.11922600 -3.08504700

C -1 -2.96945600 -6.62878600 -5.35192600

H -1 -2.01345700 -7.10994300 -5.49669700

H -1 -3.68507500 -6.76359000 -6.14949400

H 0 -3.41548200 -7.15094700 -4.49060000

C 0 -2.72788800 -5.17450800 -4.94781700

H 0 -3.66128900 -4.69764100 -4.62300500

H 0 -2.34158100 -4.57970900 -5.78285400

C 0 -1.71716500 -5.17337000 -3.79499700

H 0 -0.72027700 -5.44773900 -4.17019300

H 0 -1.98711400 -5.92785800 -3.04868200

C 0 -1.57537700 -3.84495600 -3.09330100

O 0 -1.64215300 -2.77870900 -3.71929900

O 0 -1.38690500 -3.97499600 -1.80621300

C -1 4.90477000 -4.67148400 1.14963800

H 0 5.72539700 -5.38342900 1.29855500

H 0 5.10566800 -4.11333200 0.23188700

C 0 4.83957000 -3.73728600 2.35507900

O 0 4.35366700 -4.12544700 3.43653100

C 0 3.58027800 -5.47943700 1.03022600

H 0 3.48256200 -6.11150100 1.92025800

H 0 3.63553300 -6.13907500 0.16014700

C 0 2.36800300 -4.60296400 0.90032300

N 0 2.12711300 -3.59929000 1.82706700

H 0 2.66081500 -3.49007300 2.68672600

C 0 1.36792300 -4.46539100 -0.03433700

H 0 1.17474400 -5.02533300 -0.93591400

C 0 1.07489100 -2.87194600 1.43127000

H 0 0.76142800 -1.95367300 1.89052400

N 0 0.56392000 -3.39245000 0.32362500

N 0 5.20529800 -2.46087000 2.13867300

H 0 5.54136200 -2.21640600 1.20319800

C -1 4.88737900 -1.38231900 3.07215300

H 0 5.78494600 -0.76505900 3.20670500

H 0 4.64037500 -1.83460700 4.03318300

C 0 3.71270400 -0.50748000 2.56444800

H 0 3.51690900 0.27323100 3.31052500

H 0 2.81314000 -1.12365900 2.52429200

C 0 3.94274800 0.13742000 1.18442800

H 0 4.39781400 -0.57384900 0.48404800

H 0 4.63464500 0.98426500 1.24516600

C 0 2.69719900 0.62803400 0.45714800

O 0 2.88885900 1.30209800 -0.59341700

O 0 1.55375100 0.29245500 0.91297200

C -1 -5.61325400 -3.84224500 2.13418000

H 0 -5.51034200 -3.10219400 2.93529500

H 0 -6.12431200 -4.71332300 2.56225600

C 0 -4.24464600 -4.27247700 1.61533800

H 0 -4.35443500 -5.00334600 0.80835300

H 0 -3.68402600 -4.77538600 2.41212400

C 0 -3.35444800 -3.16465700 1.06973100

O 0 -3.70922900 -1.95863100 1.13368400

O 0 -2.23733900 -3.56592600 0.59735600

C 0 -6.49322500 -3.28311800 1.02011000

O 0 -6.29041100 -3.53606700 -0.17634400

N 0 -7.54688100 -2.54054500 1.42801800

H 0 -7.61561700 -2.28348700 2.40326800

C -1 -8.51968700 -1.96823100 0.50369700

H 0 -9.46531900 -1.83051300 1.02859300

H 0 -8.66040500 -2.67341100 -0.32033700

C 0 -8.07977900 -0.59437500 -0.03639200

O 0 -8.64288000 0.44250500 0.31676100

N 0 -7.04689800 -0.63923300 -0.91291200

H 0 -6.60064300 -1.52668700 -1.11717400

C 0 -6.47180600 0.54547700 -1.50529800

H 0 -6.67200300 0.58730100 -2.58111000

H 0 -6.95229900 1.41427000 -1.04382000

C 0 -4.95437000 0.67742800 -1.32951900

O 0 -4.31957600 1.29605100 -2.19621400

O 0 -4.47378800 0.14644400 -0.25264400

C -1 -3.52627000 3.76420900 4.90316900

H -1 -3.58494300 3.47564500 5.94225000

H -1 -4.32519800 4.42057700 4.59129000

H 0 -2.61339200 4.37105700 4.81304000

C 0 -3.46597100 2.62094400 3.87530400

H 0 -3.74116700 3.03351500 2.89613600

H 0 -4.20456200 1.84154800 4.09298200

C 0 -2.06530000 2.01315500 3.75297700

H 0 -1.85480100 1.36205400 4.61158600

H 0 -1.30408100 2.79838000 3.77934100

C 0 -1.83589700 1.23891800 2.46465600

O 0 -0.69169900 1.36974700 1.93823100

O 0 -2.77641100 0.51539500 1.99856500

C -1 -5.44607400 9.19252700 1.84293300

H -1 -5.33835300 8.78645900 2.83787400

H -1 -6.47264400 9.44609600 1.62336800

H 0 -4.87355600 10.12692500 1.80261300

C 0 -4.87317200 8.17326000 0.84237200

H 0 -3.78033300 8.15855600 0.96173100

H 0 -5.06026400 8.49429400 -0.19072800

C 0 -5.44550200 6.76145500 1.05245200

H 0 -6.52488600 6.77414000 0.85770700

H 0 -5.31833000 6.46149100 2.10186400

C 0 -4.82576000 5.67965800 0.16020100

H 0 -4.92471000 5.96189500 -0.89813700

H 0 -5.35821400 4.73379700 0.30913800

N 0 -3.41647100 5.46634100 0.50167900

H 0 -2.90601200 6.26295800 0.85856600

C 0 -2.65463300 4.45082100 0.04248700

N 0 -3.15923900 3.51711000 -0.76928700

H 0 -2.69912800 2.60144700 -0.85675100

H 0 -4.06792900 3.60526200 -1.19668900

N 0 -1.37064100 4.37930600 0.39769700

H 0 -1.00295800 4.79454500 1.24761800

H 0 -0.75558200 3.64711500 0.00256700

C -1 -1.21979000 -1.22453800 7.52453400

H -1 -0.22107800 -0.84524700 7.36602100

H -1 -1.42458900 -1.42189700 8.56639200

H 0 -1.94778100 -0.49298500 7.15814600

C 0 -1.40516200 -2.59986300 6.85577900

H 0 -2.37913800 -3.01458800 7.14993000

H 0 -0.65146800 -3.29300100 7.25085900

C 0 -1.33029600 -2.61054300 5.36661300

N 0 -1.18454400 -3.78902900 4.64496900

C 0 -1.43501000 -1.62383500 4.42575800

H 0 -1.56184300 -0.56418900 4.54135300

C 0 -1.19927500 -3.53397300 3.33231600

H 0 -1.10007200 -4.25117600 2.53490000

N 0 -1.35963100 -2.21944200 3.18236100

H 0 -1.33659900 -1.72729200 2.26484700

C -1 3.76282800 6.20109100 2.41303500

H -1 4.64786400 6.67821700 2.01872900

H -1 3.28682100 6.73182300 3.22429500

H 0 3.02339600 6.17382100 1.60501100

C 0 4.09406600 4.74330500 2.81201900

H 0 5.02694100 4.68202100 3.38138100

H 0 3.27968500 4.34122100 3.42582200

C 0 4.16656400 3.89362500 1.54428800

O 0 3.10186500 3.82330800 0.87914800

O 0 5.27009600 3.35516200 1.19814200

C -1 12.03139900 -2.56639900 -0.34880800

H -1 13.08280000 -2.32138500 -0.37921300

H -1 11.80533700 -3.45591100 -0.91807000

H 0 11.79639500 -2.81957500 0.69471800

C 0 11.15039200 -1.37723700 -0.80649500

H 0 11.69005900 -0.78679400 -1.56005100

H 0 10.25063300 -1.75565400 -1.30783900

C 0 10.72774100 -0.46280400 0.35934900

H 0 9.95161400 -0.97040100 0.95010600

H 0 11.58116200 -0.32982800 1.03841300

C 0 10.25912900 0.94252900 -0.05250300

H 0 10.03979300 1.53817300 0.84384600

H 0 11.09158000 1.44767100 -0.56025700

C 0 9.06138200 1.04196200 -1.00045800

H 0 8.98667600 2.06577600 -1.37649800

H 0 9.15934500 0.37849100 -1.86281000

N 0 7.73685500 0.74253400 -0.35609800

H 0 7.00256000 1.33237800 -0.81271300

H 0 7.42541900 -0.24444000 -0.46033900

H 0 7.69577800 1.02707500 0.65078000

O 0 2.37812700 1.28400600 -3.21746600

H 0 3.13666600 1.85419600 -3.50226700

O 0 0.44495100 2.49373300 -0.25988500

O 0 0.72432400 3.90871800 2.37053200

H 0 0.36941900 3.02854800 2.15015400

H 0 1.50593000 3.97271500 1.77245300

O 0 0.99741900 -0.70697900 -4.36893400

H 0 1.48156100 0.13007000 -4.12450900

H 0 1.49606100 -1.37923300 -3.86720600

O 0 -3.37159000 -0.46736600 -4.09106800

H 0 -3.83904900 0.23723300 -3.59009500

H 0 -3.81249700 -1.29428700 -3.81724300

O 0 -4.72256100 -2.44514000 -2.30874500

H 0 -3.89106500 -2.17040000 -1.85614300

H 0 -5.16589400 -3.02469600 -1.66134400

O 0 2.45714200 3.65338500 -1.72834100

H 0 2.82531400 3.67056000 -0.80637300

H 0 3.75857800 -1.47640200 -1.80568600

O 0 0.17964500 -1.98146300 -1.84678500

O 0 5.99086900 2.70022000 -1.27282300

H 0 5.50446300 2.88885900 -0.41317200

H 0 6.54297600 3.49043500 -1.39414800

O 0 4.36953200 3.15668600 -3.55014300

H 0 4.98817800 2.81685300 -2.86790300

O 0 7.31338500 1.89672600 2.15640700

H 0 6.57079500 2.51682700 1.92574200

H 0 6.97836300 1.36884500 2.89660100

O 0 2.84371600 -1.29148900 -2.09415600

H 0 2.82382900 -0.33315100 -2.32097800

H 0 1.02995400 -1.61895800 -1.50651200

H 0 -1.08090900 -4.71350300 5.04636000

H 0 3.68494400 3.57520700 -2.96636900

Cl 0 6.11483400 -1.91804600 -1.01435100

**TS_Ha-Hb_**

E (IEFPCM/B3LYP-D3/SDD,6-31G(d)) = -5854.11845 au

H (IEFPCM/B3LYP-D3/SDD,6-31G(d)) = -5852.572339 au

G (IEFPCM/B3LYP-D3/SDD,6-31G(d)) = -5852.808686 au

E (IEFPCM/B3LYP-D3/SDD,6-311+G(2d,p)//IEFPCM/B3LYP-D3/SDD,6-31G(d)) = -5855.93505 au

Mn 0 1.42616600 2.00590700 -1.85438000

Mn 0 -1.04191100 -2.46272400 -0.71846300

Mn 0 -0.18271700 0.85039800 0.02631600

Mn 0 -2.70106100 -0.38550500 0.23517800

O 0 0.32148300 0.52353100 -1.63891900

O 0 -1.92861100 1.05884100 -0.48504400

O 0 -2.53852100 -1.44631300 -1.22724900

O 0 -0.96640900 -1.00742300 0.59270000

Ca 0 -1.42614400 -0.39457800 -3.10238400

C -1 0.60660500 4.48075700 -5.65670700

H -1 0.67744000 5.42250500 -5.13273000

H -1 0.80960300 4.53707200 -6.71577100

H 0 1.47446700 3.92210800 -5.27384000

C 0 -0.64414800 3.65987400 -5.27680100

H 0 -0.93693400 2.99316100 -6.09473800

H 0 -1.51455800 4.30412900 -5.09442600

C 0 -0.52239800 2.73466600 -4.04061800

O 0 -1.33823000 1.79615100 -3.93560200

O 0 0.41304400 3.01422300 -3.19842600

C -1 -3.00966900 -6.72492200 -5.25611300

H -1 -2.05362700 -7.20367400 -5.40772500

H -1 -3.73424700 -6.87011900 -6.04388500

H 0 -3.44085700 -7.22179700 -4.37348600

C 0 -2.79276400 -5.25103200 -4.92000800

H 0 -3.73418300 -4.79018800 -4.59587900

H 0 -2.45056500 -4.68373200 -5.79279300

C 0 -1.75858200 -5.15952200 -3.79642200

H 0 -0.75061300 -5.35428600 -4.19147900

H 0 -1.94753400 -5.92779800 -3.03920400

C 0 -1.71579200 -3.81957200 -3.10282800

O 0 -1.89298500 -2.76357700 -3.72655100

O 0 -1.48495200 -3.93651300 -1.82266500

C -1 4.93247200 -4.67506400 1.13317400

H 0 5.71341400 -5.41614600 1.33990800

H 0 5.22435300 -4.13077200 0.23084700

C 0 4.82058300 -3.72447400 2.32435600

O 0 4.23519700 -4.07935800 3.36931700

C 0 3.59088600 -5.43781900 0.92209600

H 0 3.43232900 -6.08874600 1.78975800

H 0 3.67824600 -6.08072900 0.04206700

C 0 2.39545600 -4.54125500 0.75528200

N 0 2.12476800 -3.55888800 1.69473200

H 0 2.63659300 -3.46990800 2.57184600

C 0 1.41288900 -4.39260900 -0.19929200

H 0 1.23869900 -4.94554400 -1.10933100

C 0 1.07185600 -2.83657600 1.29146500

H 0 0.73789700 -1.93264800 1.76260300

N 0 0.59036900 -3.33443600 0.16176000

N 0 5.25040300 -2.46833700 2.12442100

H 0 5.77475200 -2.26218800 1.26551000

C -1 4.92426700 -1.36812100 3.02477100

H 0 5.81242300 -0.73230500 3.12320100

H 0 4.69406300 -1.78564300 4.00623100

C 0 3.72840600 -0.53061600 2.50936600

H 0 3.52772900 0.26998700 3.23220800

H 0 2.83863200 -1.16197500 2.49915000

C 0 3.93583500 0.07728300 1.11160100

H 0 4.41540600 -0.64347600 0.43528300

H 0 4.60852100 0.94225100 1.14802900

C 0 2.66952000 0.54191300 0.39608000

O 0 2.84492800 1.13394900 -0.71067200

O 0 1.53489900 0.28112900 0.90293200

C -1 -5.57643500 -3.87835700 2.23411700

H 0 -5.49187400 -3.13998000 3.03890100

H 0 -6.05872600 -4.76750500 2.65843600

C 0 -4.19469700 -4.26681400 1.70530800

H 0 -4.29252200 -5.00627600 0.90478200

H 0 -3.61688300 -4.75190700 2.50140100

C 0 -3.31935400 -3.14947700 1.14118900

O 0 -3.69222600 -1.94183100 1.21969400

O 0 -2.21161500 -3.52943300 0.64942300

C 0 -6.47596100 -3.34082600 1.12452700

O 0 -6.28695800 -3.60963100 -0.06945900

N 0 -7.53181700 -2.60234000 1.53912900

H 0 -7.58732800 -2.33212700 2.51178700

C -1 -8.50959900 -2.03143400 0.62063500

H 0 -9.47462400 -1.96594500 1.12490000

H 0 -8.59309400 -2.70102100 -0.23928700

C 0 -8.11840500 -0.61337500 0.17222400

O 0 -8.68999800 0.38082100 0.62291600

N 0 -7.10978600 -0.56560500 -0.72997400

H 0 -6.63692100 -1.41528700 -1.02204600

C 0 -6.56424300 0.67933100 -1.22023900

H 0 -6.84485400 0.85446300 -2.26411100

H 0 -6.98816100 1.48925300 -0.61746100

C 0 -5.03570200 0.75096000 -1.14160500

O 0 -4.42734700 1.33696300 -2.05000900

O 0 -4.51637600 0.20598700 -0.09101100

C -1 -3.48815400 3.76212800 4.90675100

H -1 -3.53333800 3.48315400 5.94907900

H -1 -4.29330000 4.41234300 4.59816100

H 0 -2.58387200 4.38112600 4.80825400

C 0 -3.41087800 2.61387700 3.89496100

H 0 -3.69460400 3.00805900 2.91065500

H 0 -4.13022000 1.81943900 4.12123200

C 0 -1.99625000 2.03753200 3.78297000

H 0 -1.77571000 1.39593100 4.64563600

H 0 -1.24984500 2.83708900 3.80083500

C 0 -1.78070500 1.26455700 2.49700200

O 0 -0.66756200 1.44404000 1.91572900

O 0 -2.71156200 0.50351400 2.08206100

C -1 -5.46597600 9.15367900 1.81853600

H -1 -5.34487500 8.75739600 2.81589200

H -1 -6.49607600 9.40104100 1.60874100

H 0 -4.90427500 10.09462800 1.77048300

C 0 -4.88654700 8.14248500 0.81484300

H 0 -3.79162100 8.15825800 0.91273500

H 0 -5.10211200 8.45176200 -0.21631100

C 0 -5.41346300 6.71671900 1.04331500

H 0 -6.49768900 6.69788600 0.87843600

H 0 -5.24882000 6.42231500 2.08907700

C 0 -4.78857200 5.65356100 0.13332500

H 0 -4.92935700 5.93139500 -0.92123200

H 0 -5.28764100 4.69227100 0.29925200

N 0 -3.36236000 5.48342200 0.42808200

H 0 -2.86724600 6.28668300 0.79176600

C 0 -2.58840700 4.49423700 -0.06328800

N 0 -3.08892500 3.56469500 -0.88285500

H 0 -2.64909500 2.63581900 -0.93193600

H 0 -4.03267300 3.61436400 -1.23447900

N 0 -1.28987700 4.45336600 0.25020000

H 0 -0.91667100 4.83911800 1.11283000

H 0 -0.69205900 3.70720900 -0.13891600

C -1 -1.13055600 -1.19248400 7.54737300

H -1 -0.13532800 -0.81070900 7.37348800

H -1 -1.32222100 -1.38081200 8.59338100

H 0 -1.86336900 -0.46302300 7.18604400

C 0 -1.31585100 -2.56636000 6.87554800

H 0 -2.28888700 -2.98417500 7.16825900

H 0 -0.56017200 -3.25960400 7.26636700

C 0 -1.24075500 -2.56896800 5.38490400

N 0 -1.08032900 -3.74167800 4.65627400

C 0 -1.35926300 -1.57902300 4.44900100

H 0 -1.50097100 -0.52238600 4.57088900

C 0 -1.10172200 -3.48014300 3.34480700

H 0 -1.00695100 -4.19187800 2.54198800

N 0 -1.27715600 -2.16694300 3.20192700

H 0 -1.28587300 -1.67524600 2.28612300

C -1 3.76111300 6.20422100 2.30764400

H -1 4.63950700 6.68114000 1.89849800

H -1 3.29263100 6.74072600 3.11962100

H 0 3.01417300 6.17221500 1.50613100

C 0 4.09695900 4.74707000 2.70423300

H 0 5.01979000 4.68924700 3.29010900

H 0 3.27540100 4.34030300 3.30598000

C 0 4.19988600 3.87514600 1.44970800

O 0 3.17353900 3.82808700 0.72391300

O 0 5.29230900 3.26927500 1.18558100

C -1 12.03225900 -2.55607700 -0.46920600

H -1 13.08223800 -2.30709400 -0.51433100

H -1 11.80320000 -3.45178800 -1.02731200

H 0 11.81372400 -2.80433900 0.57956900

C 0 11.15955800 -1.35833200 -0.90015300

H 0 11.60474100 -0.87559000 -1.78195500

H 0 10.16567900 -1.70870600 -1.20559400

C 0 11.00202600 -0.33009400 0.23688800

H 0 10.26664800 -0.71283300 0.95890900

H 0 11.94987700 -0.25279300 0.78730000

C 0 10.62379400 1.09227100 -0.20557800

H 0 10.56846400 1.74892300 0.67337600

H 0 11.42919000 1.49040000 -0.83715000

C 0 9.33379200 1.24709000 -1.00923200

H 0 9.23416900 2.28113800 -1.35091400

H 0 9.32378300 0.59975500 -1.88997000

N 0 8.09406400 0.93525900 -0.22965400

H 0 7.27438200 1.38693900 -0.69787500

H 0 7.89183800 -0.08994900 -0.19015600

H 0 8.08578200 1.33646000 0.73693700

O 0 2.24008400 0.91346600 -3.28796400

H 0 3.12884800 1.30804500 -3.51501700

O 0 0.48611200 2.48590900 -0.33802700

O 0 0.78306400 3.98050700 2.23255900

H 0 0.41214900 3.09384000 2.07217000

H 0 1.55074700 4.00545600 1.61452400

O 0 0.42317700 -0.52845300 -4.69148000

H 0 1.08864300 0.13466400 -4.36795400

H 0 0.86405600 -1.38532700 -4.56457800

O 0 -3.69028200 -0.43236200 -4.02801900

H 0 -4.13012600 0.28496100 -3.52108900

H 0 -4.12777200 -1.24927900 -3.71417700

O 0 -4.92534700 -2.27990700 -2.16811100

H 0 -4.06478100 -2.05056900 -1.74072200

H 0 -5.30312600 -2.96721500 -1.58704400

O 0 2.56276700 3.45091600 -1.90186200

H 0 2.93083000 3.52412700 -0.98373900

H 0 2.89741700 -1.41955500 -1.63455700

O 0 -0.00353600 -1.88378600 -1.95478800

O 0 6.03596500 2.50012900 -1.24097800

H 0 5.56795200 2.74255800 -0.38307600

H 0 6.44261300 3.33499400 -1.52629600

O 0 4.44416500 2.43586100 -3.54799900

H 0 5.03067600 2.18221000 -2.80242200

O 0 7.54123600 2.19248500 2.22169900

H 0 6.72224300 2.68726200 1.95401500

H 0 7.23174600 1.54757700 2.87611000

O 0 2.30691700 -1.36436500 -2.40264500

H 0 2.33955000 -0.25266700 -2.78096700

H 0 -0.96454100 -4.66707400 5.05212700

H 0 3.79956200 3.01504000 -3.06194600

Cl 0 7.08030700 -2.01835500 -0.48301200

H 0 1.20591400 -1.61248100 -2.04538700

**Hb_S_3_**

E (IEFPCM/B3LYP-D3/SDD,6-31G(d)) = -5854.134007 au

H (IEFPCM/B3LYP-D3/SDD,6-31G(d)) = -5852.579047 au

G (IEFPCM/B3LYP-D3/SDD,6-31G(d)) = -5852.81495 au

E (IEFPCM/B3LYP-D3/SDD,6-311+G(2d,p)//IEFPCM/B3LYP-D3/SDD,6-31G(d)) = -5855.948003 au

Mn 0 1.42217800 2.13724100 -1.75078700

Mn 0 -0.92976500 -2.50938900 -0.65864300

Mn 0 -0.16660900 0.81370400 0.03278200

Mn 0 -2.66891300 -0.44045400 0.17562200

O 0 0.46362400 0.55440100 -1.62502200

O 0 -1.88158400 1.00072900 -0.55395000

O 0 -2.42457500 -1.51199000 -1.25275000

O 0 -0.94085600 -1.00814200 0.63319600

Ca 0 -1.14672100 -0.53221900 -3.09652800

C -1 0.66661800 4.47815600 -5.68245600

H -1 0.73150200 5.42383700 -5.16483000

H -1 0.87946100 4.52687300 -6.73996200

H 0 1.54361100 3.93541000 -5.29421100

C 0 -0.57631100 3.64966800 -5.28225300

H 0 -0.92065800 3.02595600 -6.11186200

H 0 -1.41964000 4.30231500 -5.01927700

C 0 -0.38598500 2.67801100 -4.09145900

O 0 -0.99717100 1.59263000 -4.11921600

O 0 0.38695600 3.08100000 -3.13507600

C -1 -2.94086500 -6.72849100 -5.23270100

H -1 -1.98291800 -7.20722900 -5.37181200

H -1 -3.65787500 -6.88036300 -6.02612000

H 0 -3.38044100 -7.23363700 -4.35824900

C 0 -2.70013700 -5.26512600 -4.86306400

H 0 -3.62737700 -4.78955000 -4.51881600

H 0 -2.34590500 -4.68334600 -5.72125900

C 0 -1.65397800 -5.23312900 -3.74355900

H 0 -0.66589100 -5.49662000 -4.14887100

H 0 -1.88806900 -5.98356900 -2.98108100

C 0 -1.51433700 -3.89406500 -3.05990000

O 0 -1.60682300 -2.83949800 -3.70871500

O 0 -1.30300200 -4.00056300 -1.78016200

C -1 4.93883000 -4.62209700 1.21524400

H 0 5.77914200 -5.31186300 1.36127000

H 0 5.10785300 -4.07738100 0.28317800

C 0 4.88033900 -3.66873400 2.40541800

O 0 4.42970600 -4.04536200 3.50433600

C 0 3.63098000 -5.45826400 1.13598500

H 0 3.55020700 -6.05992000 2.04854200

H 0 3.69074800 -6.14672700 0.28873100

C 0 2.41264400 -4.59710700 0.98097400

N 0 2.16354500 -3.57037500 1.87885700

H 0 2.68333100 -3.43829700 2.74185600

C 0 1.42617900 -4.47773100 0.02896100

H 0 1.24517300 -5.06135700 -0.86030500

C 0 1.11535100 -2.85159600 1.44644500

H 0 0.79488400 -1.92273700 1.87850300

N 0 0.62195000 -3.39516200 0.34548400

N 0 5.22358900 -2.38945000 2.15872800

H 0 5.50724200 -2.15513600 1.20500700

C -1 4.90926000 -1.30126300 3.08213000

H 0 5.80215200 -0.67410200 3.19949000

H 0 4.67419500 -1.74407900 4.05034800

C 0 3.72273900 -0.44082400 2.57420300

H 0 3.51747100 0.33794500 3.31963000

H 0 2.83161900 -1.06943500 2.53660900

C 0 3.93998300 0.20935800 1.19237700

H 0 4.42629200 -0.48356600 0.49417100

H 0 4.60054700 1.08043700 1.25711200

C 0 2.68064700 0.65692900 0.46119000

O 0 2.85506000 1.30768400 -0.61524000

O 0 1.54798100 0.32451200 0.93048800

C -1 -5.58079900 -3.82960700 2.21189300

H 0 -5.47859600 -3.07165200 2.99626800

H 0 -6.08974700 -4.69217600 2.65995300

C 0 -4.21153800 -4.26610500 1.69891500

H 0 -4.32473600 -5.01209400 0.90635100

H 0 -3.65328500 -4.75731400 2.50504900

C 0 -3.30409000 -3.17840200 1.13082500

O 0 -3.66472900 -1.96143900 1.17945400

O 0 -2.19756600 -3.58283200 0.67289300

C 0 -6.46791800 -3.29744400 1.09029300

O 0 -6.27846100 -3.58272400 -0.10089300

N 0 -7.51761200 -2.54181500 1.48756300

H 0 -7.57585100 -2.25635500 2.45552000

C -1 -8.50076500 -1.99811700 0.55740300

H 0 -9.44308500 -1.85185800 1.08623900

H 0 -8.64418500 -2.72538400 -0.24680300

C 0 -8.07490500 -0.63702400 -0.02286000

O 0 -8.64618400 0.40463300 0.30380000

N 0 -7.04697200 -0.69790600 -0.90318300

H 0 -6.58646500 -1.58465200 -1.07821900

C 0 -6.47375000 0.47577000 -1.51815100

H 0 -6.65651800 0.48486000 -2.59782900

H 0 -6.97087900 1.35210200 -1.08996400

C 0 -4.96047600 0.62694800 -1.32054800

O 0 -4.32511800 1.24978400 -2.18645500

O 0 -4.48567900 0.11016900 -0.23747100

C -1 -3.52607800 3.83288800 4.84726700

H -1 -3.58071200 3.56158000 5.89116500

H -1 -4.32901700 4.47985700 4.52634900

H 0 -2.61665900 4.44301800 4.74468900

C 0 -3.46313700 2.67339500 3.84052400

H 0 -3.74813600 3.06533500 2.85571000

H 0 -4.19274100 1.89092700 4.07639900

C 0 -2.05798700 2.07688500 3.72158700

H 0 -1.83950400 1.44139200 4.58956800

H 0 -1.30353500 2.86884800 3.73094400

C 0 -1.84124000 1.28330500 2.44375900

O 0 -0.70354000 1.43216200 1.89529100

O 0 -2.77195500 0.54005100 2.00709600

C -1 -5.48085200 9.19911800 1.70082500

H -1 -5.36865800 8.81036700 2.70217800

H -1 -6.50921500 9.44371200 1.47957500

H 0 -4.91054900 10.13357400 1.63972400

C 0 -4.91250000 8.15941400 0.71887500

H 0 -3.81910900 8.14690200 0.83354400

H 0 -5.10413000 8.45795900 -0.32009300

C 0 -5.48521400 6.75297100 0.96309900

H 0 -6.56423700 6.76021500 0.76652900

H 0 -5.35945000 6.47962200 2.01990300

C 0 -4.86270900 5.64951300 0.10013400

H 0 -4.96368200 5.90139900 -0.96560400

H 0 -5.38948800 4.70515100 0.27608600

N 0 -3.45168700 5.45568000 0.44613100

H 0 -2.94887300 6.26599900 0.78266600

C 0 -2.67976800 4.44087100 0.00493700

N 0 -3.17249700 3.48166600 -0.78212500

H 0 -2.70035900 2.56890700 -0.85261100

H 0 -4.08747500 3.54233800 -1.20139000

N 0 -1.39027700 4.40050400 0.35191700

H 0 -1.02576200 4.82819400 1.19660300

H 0 -0.77387300 3.66990500 -0.03375900

C -1 -1.18783600 -1.09928200 7.54646400

H -1 -0.19144600 -0.71763500 7.37907400

H -1 -1.38908000 -1.28009100 8.59199900

H 0 -1.91846500 -0.37457100 7.17141500

C 0 -1.36720700 -2.48065000 6.88912200

H 0 -2.34153000 -2.89555400 7.18190900

H 0 -0.61343100 -3.16861500 7.29328700

C 0 -1.28413300 -2.50276900 5.39957900

N 0 -1.14422300 -3.68894500 4.68898200

C 0 -1.37555600 -1.52428800 4.44871700

H 0 -1.49229300 -0.46270900 4.55517600

C 0 -1.15143900 -3.44528900 3.37371200

H 0 -1.06411900 -4.16894300 2.58070700

N 0 -1.29989100 -2.13146500 3.21048700

H 0 -1.28125400 -1.65032100 2.28488900

C -1 3.74450500 6.26419700 2.29807100

H -1 4.62616400 6.73910600 1.89363800

H -1 3.26784800 6.80614300 3.10163300

H 0 3.00395300 6.19695900 1.49380100

C 0 4.09941600 4.83040300 2.76275700

H 0 5.02454700 4.81674300 3.34747700

H 0 3.28449200 4.43970500 3.38301400

C 0 4.20993500 3.92794300 1.53848200

O 0 3.15372400 3.78478300 0.87212400

O 0 5.33786900 3.41933200 1.22253300

C -1 12.05096400 -2.50668800 -0.33630000

H -1 13.10104500 -2.25681300 -0.37344400

H -1 11.82813100 -3.40677100 -0.88987400

H 0 11.82071300 -2.74360700 0.71205200

C 0 11.15872000 -1.33481700 -0.81356600

H 0 11.69927500 -0.73768400 -1.56109100

H 0 10.27489800 -1.73250300 -1.32828500

C 0 10.69987500 -0.42267400 0.33907600

H 0 9.94396200 -0.95201800 0.93719700

H 0 11.54676500 -0.24516700 1.01596900

C 0 10.17364900 0.95324100 -0.09900600

H 0 9.92916300 1.55836100 0.78415900

H 0 10.98268500 1.48216200 -0.62001900

C 0 8.97141100 0.97579700 -1.04583300

H 0 8.85813600 1.98178800 -1.45793300

H 0 9.08995600 0.28696400 -1.88493600

N 0 7.65805900 0.65487400 -0.38404700

H 0 6.92598000 1.27052100 -0.80614300

H 0 7.33276500 -0.32216800 -0.51692200

H 0 7.63727100 0.90664400 0.63098400

O 0 2.45633500 1.21798500 -3.28784400

H 0 3.29650500 1.76661800 -3.45585900

O 0 0.42823400 2.50322100 -0.29078800

O 0 0.73617300 3.94810800 2.30907300

H 0 0.36881100 3.06683300 2.11390200

H 0 1.53107200 3.98026800 1.72729300

O 0 0.83309900 -0.73715900 -4.54494600

H 0 1.27111800 0.13387900 -4.45421700

H 0 1.44951900 -1.33349000 -4.07633000

O 0 -3.40252400 -0.54254700 -4.07269400

H 0 -3.86143400 0.16888600 -3.57296400

H 0 -3.83529600 -1.36522000 -3.77286600

O 0 -4.71367100 -2.50179700 -2.25224900

H 0 -3.88092700 -2.21132300 -1.80597500

H 0 -5.14344000 -3.08261900 -1.59721600

O 0 2.44383100 3.67200000 -1.72997100

H 0 2.84559300 3.69192800 -0.82347800

H 0 3.49079900 -1.59333800 -1.93166300

O 0 0.17974200 -1.90911300 -1.75725100

O 0 6.01784400 2.71657500 -1.22384800

H 0 5.56757400 2.94041900 -0.34787600

H 0 6.61087000 3.47109400 -1.37473700

O 0 4.36576900 2.97476200 -3.44221500

H 0 5.00440800 2.72756300 -2.73316000

O 0 7.29949000 1.79766000 2.13897900

H 0 6.58740800 2.45843100 1.93632400

H 0 6.99810900 1.32984400 2.93193600

O 0 2.71474000 -1.31405500 -2.45659400

H 0 2.71416800 0.33022900 -2.89264500

H 0 -1.05111700 -4.61052500 5.09884700

H 0 3.68735300 3.46848200 -2.90238800

Cl 0 5.86725700 -1.90466900 -1.10867200

H 0 1.88836700 -1.47837600 -1.92991900

**TS_Hb-Hc_**

E (IEFPCM/B3LYP-D3/SDD,6-31G(d)) = -5854.126207 au

H (IEFPCM/B3LYP-D3/SDD,6-31G(d)) = -5852.577285 au

G (IEFPCM/B3LYP-D3/SDD,6-31G(d)) = -5852.81112 au

E (IEFPCM/B3LYP-D3/SDD,6-311+G(2d,p)//IEFPCM/B3LYP-D3/SDD,6-31G(d)) = -5855.939318 au

Mn 0 1.41713900 2.09587800 -1.78803200

Mn 0 -0.93127300 -2.50459100 -0.66428100

Mn 0 -0.16441600 0.82367000 0.03712200

Mn 0 -2.66603800 -0.43991900 0.17734400

O 0 0.46063000 0.54689000 -1.63165000

O 0 -1.88042200 1.00638700 -0.54507400

O 0 -2.42495500 -1.50475100 -1.25589100

O 0 -0.94176600 -1.01095500 0.63397400

Ca 0 -1.14341700 -0.53390500 -3.11620400

C -1 0.65475100 4.48266500 -5.67775600

H -1 0.71940200 5.42780900 -5.15912000

H -1 0.86620900 4.53280400 -6.73547400

H 0 1.52999000 3.93762100 -5.28944600

C 0 -0.59044600 3.65612200 -5.28045900

H 0 -0.93243500 3.03197700 -6.11088100

H 0 -1.43467800 4.30951200 -5.02221400

C 0 -0.40956300 2.68308200 -4.08917400

O 0 -1.04447000 1.61204200 -4.10712600

O 0 0.37933600 3.07081200 -3.13887300

C -1 -2.94161600 -6.72788700 -5.23638700

H -1 -1.98339400 -7.20556100 -5.37726200

H -1 -3.65948700 -6.87951800 -6.02907000

H 0 -3.37938000 -7.23343000 -4.36130800

C 0 -2.70160000 -5.26406800 -4.86759200

H 0 -3.62818900 -4.79012300 -4.51921200

H 0 -2.35235700 -4.68174100 -5.72745500

C 0 -1.65029700 -5.22958800 -3.75283000

H 0 -0.66343900 -5.49003800 -4.16300100

H 0 -1.87911700 -5.98078000 -2.98952800

C 0 -1.51170400 -3.88997100 -3.06980300

O 0 -1.60084000 -2.83616100 -3.72129700

O 0 -1.30601100 -3.99444000 -1.78970000

C -1 4.94426100 -4.62151600 1.20400800

H 0 5.78311800 -5.31280100 1.35129400

H 0 5.11729900 -4.07574800 0.27341100

C 0 4.88369500 -3.66966000 2.39565100

O 0 4.42795400 -4.04701300 3.49240800

C 0 3.63546100 -5.45594600 1.11997800

H 0 3.55294000 -6.06071000 2.03035400

H 0 3.69611100 -6.14170800 0.27057700

C 0 2.41710100 -4.59435500 0.96630500

N 0 2.16693000 -3.57027000 1.86690100

H 0 2.68647600 -3.44028200 2.73041700

C 0 1.42973800 -4.47404400 0.01520600

H 0 1.24932900 -5.05558900 -0.87555800

C 0 1.11721500 -2.85221600 1.43694500

H 0 0.79568500 -1.92475100 1.87115300

N 0 0.62387000 -3.39354500 0.33491000

N 0 5.23049700 -2.39089700 2.15221200

H 0 5.52439600 -2.15678000 1.20110000

C -1 4.91393300 -1.30286800 3.07476700

H 0 5.80652800 -0.67536700 3.19377600

H 0 4.67747100 -1.74529800 4.04285900

C 0 3.72768100 -0.44310800 2.56513000

H 0 3.52090000 0.33574100 3.31010100

H 0 2.83692200 -1.07205200 2.52639700

C 0 3.94566600 0.20638200 1.18324500

H 0 4.43403800 -0.48654300 0.48637000

H 0 4.60556900 1.07787500 1.24856700

C 0 2.68577400 0.65310400 0.45101000

O 0 2.86026900 1.30156500 -0.62608300

O 0 1.55362300 0.32039100 0.92168600

C -1 -5.57483800 -3.84008800 2.21489000

H 0 -5.47365800 -3.08350700 3.00075000

H 0 -6.08114500 -4.70488000 2.66165400

C 0 -4.20505800 -4.27191000 1.69937400

H 0 -4.31734600 -5.01884200 0.90761900

H 0 -3.64389500 -4.76078900 2.50493100

C 0 -3.30132900 -3.18200900 1.12859100

O 0 -3.66314700 -1.96548500 1.17878600

O 0 -2.19606400 -3.58530000 0.66675700

C 0 -6.46418500 -3.30765900 1.09515600

O 0 -6.27572800 -3.59088000 -0.09665100

N 0 -7.51468000 -2.55424900 1.49481200

H 0 -7.57184400 -2.26949400 2.46304600

C -1 -8.49862000 -2.00944100 0.56621500

H 0 -9.44109500 -1.86567600 1.09543300

H 0 -8.64117100 -2.73492200 -0.23973600

C 0 -8.07446800 -0.64634800 -0.01079000

O 0 -8.64650000 0.39385600 0.31934300

N 0 -7.04752800 -0.70381100 -0.89241700

H 0 -6.58625400 -1.58955100 -1.07059000

C 0 -6.47701900 0.47193700 -1.50628300

H 0 -6.66267100 0.48304100 -2.58543300

H 0 -6.97399600 1.34672300 -1.07475600

C 0 -4.96327500 0.62408700 -1.31229500

O 0 -4.32993100 1.24424600 -2.18165600

O 0 -4.48575700 0.11193100 -0.22847400

C -1 -3.52398900 3.82129200 4.85651400

H -1 -3.57704400 3.54872800 5.90016500

H -1 -4.32794200 4.46787300 4.53736200

H 0 -2.61567300 4.43315300 4.75409300

C 0 -3.45902300 2.66293800 3.84881000

H 0 -3.74599300 3.05454500 2.86447400

H 0 -4.18601500 1.87809500 4.08480200

C 0 -2.05211300 2.07078200 3.72818800

H 0 -1.83042900 1.43568900 4.59568800

H 0 -1.30010300 2.86506400 3.73675600

C 0 -1.83539500 1.27817300 2.44979400

O 0 -0.69730400 1.42823200 1.90118900

O 0 -2.76593100 0.53549600 2.01314900

C -1 -5.48779600 9.18930700 1.71875400

H -1 -5.37396700 8.79950600 2.71951400

H -1 -6.51666700 9.43318700 1.49908900

H 0 -4.91928500 10.12500500 1.65906100

C 0 -4.91716900 8.15299000 0.73435100

H 0 -3.82357100 8.14412200 0.84748800

H 0 -5.11140000 8.45273700 -0.30380500

C 0 -5.48387300 6.74383400 0.97654700

H 0 -6.56374500 6.74804100 0.78444600

H 0 -5.35285500 6.46760800 2.03196400

C 0 -4.86165900 5.64500700 0.10732700

H 0 -4.96712200 5.90068400 -0.95711200

H 0 -5.38631300 4.69916500 0.28158500

N 0 -3.44917100 5.45127600 0.44732100

H 0 -2.94486400 6.26095800 0.78312400

C 0 -2.67941900 4.43598700 0.00222300

N 0 -3.17598500 3.47954500 -0.78574000

H 0 -2.70548900 2.56598600 -0.85756200

H 0 -4.09231800 3.54223700 -1.20164900

N 0 -1.38911300 4.39277200 0.34564400

H 0 -1.02409700 4.81741600 1.19185600

H 0 -0.77462900 3.65760500 -0.03829900

C -1 -1.17768900 -1.11178500 7.54704600

H -1 -0.18187200 -0.72900700 7.37883600

H -1 -1.37743800 -1.29399100 8.59262600

H 0 -1.90907100 -0.38667300 7.17415800

C 0 -1.35720000 -2.49183400 6.88671100

H 0 -2.33087200 -2.90789400 7.18004800

H 0 -0.60253600 -3.18026000 7.28839900

C 0 -1.27628100 -2.51118500 5.39675100

N 0 -1.13581700 -3.69605100 4.68391300

C 0 -1.37079800 -1.53141000 4.44744700

H 0 -1.48891400 -0.47019200 4.55590700

C 0 -1.14567400 -3.45018200 3.36895900

H 0 -1.05849300 -4.17228600 2.57448800

N 0 -1.29628200 -2.13640100 3.20801000

H 0 -1.27929800 -1.65409100 2.28230900

C -1 3.74106800 6.26239100 2.30092600

H -1 4.62176700 6.73859700 1.89592600

H -1 3.26492000 6.80295900 3.10571700

H 0 2.99885300 6.20176700 1.49754600

C 0 4.09528600 4.82391000 2.75136100

H 0 5.02366400 4.80193700 3.33052900

H 0 3.28267000 4.42827900 3.37138300

C 0 4.19647900 3.93533000 1.51617100

O 0 3.13547900 3.80214400 0.85546800

O 0 5.32140300 3.42817600 1.18581700

C -1 12.05242900 -2.49761700 -0.35409400

H -1 13.10222600 -2.24671000 -0.39227900

H -1 11.82974200 -3.39726900 -0.90842600

H 0 11.82533700 -2.73807500 0.69420400

C 0 11.15839300 -1.32343900 -0.82315300

H 0 11.69724900 -0.72139200 -1.56795200

H 0 10.27311200 -1.71762100 -1.33804600

C 0 10.70314600 -0.41933500 0.33759600

H 0 9.94532500 -0.95108700 0.93107700

H 0 11.55090200 -0.25210700 1.01601700

C 0 10.18372000 0.96411400 -0.08492500

H 0 9.94229400 1.55963600 0.80562600

H 0 10.99556300 1.49526100 -0.59928100

C 0 8.98220900 1.00621100 -1.03178000

H 0 8.86867500 2.02093800 -1.42194700

H 0 9.10131000 0.33490600 -1.88508800

N 0 7.66954000 0.66989100 -0.37794900

H 0 6.92656000 1.25601600 -0.81800700

H 0 7.36642700 -0.31696700 -0.49610800

H 0 7.63518300 0.93960300 0.63274700

O 0 2.45146700 1.28513200 -3.26371200

H 0 3.47809400 2.05433700 -3.38783100

O 0 0.43017100 2.49751900 -0.29864300

O 0 0.72799700 3.94845400 2.31253200

H 0 0.36350900 3.06633200 2.11500000

H 0 1.51692000 3.98745100 1.72380000

O 0 0.87419500 -0.64132100 -4.51589600

H 0 1.32290300 0.21338900 -4.31299900

H 0 1.46854600 -1.28573000 -4.08466900

O 0 -3.40755300 -0.54193800 -4.07331700

H 0 -3.86160600 0.16813600 -3.56696400

H 0 -3.83809700 -1.36510600 -3.77182900

O 0 -4.71491100 -2.50019100 -2.24737100

H 0 -3.88204700 -2.20683700 -1.80291600

H 0 -5.13993100 -3.08480700 -1.59264000

O 0 2.46456700 3.69538800 -1.73960300

H 0 2.82600200 3.71622200 -0.81485900

H 0 3.49664900 -1.62206200 -1.91207600

O 0 0.17475600 -1.90215200 -1.76356900

O 0 5.97384700 2.69381500 -1.25245600

H 0 5.54097300 2.93149800 -0.36790900

H 0 6.56768100 3.44336800 -1.42600300

O 0 4.19991000 2.95100100 -3.29088300

H 0 4.90332300 2.69045800 -2.63737700

O 0 7.28832500 1.83081100 2.13320300

H 0 6.57413800 2.48652600 1.92149700

H 0 6.97377800 1.35117700 2.91405300

O 0 2.73386900 -1.34297300 -2.45312000

H 0 2.72758100 0.41005100 -2.88127200

H 0 -1.04032200 -4.61818000 5.09198700

H 0 3.49109700 3.46060300 -2.61866400

Cl 0 5.93336300 -1.90908100 -1.08672000

H 0 1.90277700 -1.49328300 -1.93424100

**La_S_4_**

E (IEFPCM/B3LYP-D3/SDD,6-31G(d)) = -5854.29258 au

H (IEFPCM/B3LYP-D3/SDD,6-31G(d)) = -5852.74023 au

G (IEFPCM/B3LYP-D3/SDD,6-31G(d)) = -5852.981454 au

E (IEFPCM/B3LYP-D3/SDD,6-311+G(2d,p)//IEFPCM/B3LYP-D3/SDD,6-31G(d)) = -5856.120271 au

Mn 0 1.26639100 2.32119000 -1.85599600

Mn 0 -1.02067700 -2.46752000 -0.77954500

Mn 0 -0.06809500 0.85393800 0.10627800

Mn 0 -2.63432600 -0.42923500 0.25604400

O 0 0.33523300 0.60105400 -1.61184600

O 0 -1.88245500 1.03651800 -0.34545000

O 0 -2.46301800 -1.41223800 -1.28344500

O 0 -0.92879900 -1.14153400 0.57863100

Ca 0 -1.43766200 -0.18582400 -3.18758100

C -1 0.56368500 4.69554300 -5.45102400

H -1 0.62548200 5.61526900 -4.88816700

H -1 0.76381300 4.79832900 -6.50708700

H 0 1.36089200 4.05225000 -5.05615900

C 0 -0.79003800 3.95315900 -5.27693800

H 0 -0.99006200 3.33040700 -6.15498700

H 0 -1.61655800 4.67619000 -5.21925000

C 0 -0.89285300 3.03873100 -4.03999400

O 0 -1.48103900 1.91915700 -4.19293200

O 0 -0.40818200 3.45488100 -2.95179900

C -1 -2.93264900 -6.55518900 -5.51412300

H -1 -1.97174900 -7.01686800 -5.68711600

H -1 -3.65710300 -6.67501100 -6.30608700

H 0 -3.35419100 -7.08458800 -4.64614500

C 0 -2.72604800 -5.08473400 -5.13167400

H 0 -3.66386000 -4.65351300 -4.75834200

H 0 -2.42965800 -4.48418900 -5.99898300

C 0 -1.64764100 -5.00070800 -4.04583900

H 0 -0.65392400 -5.15543400 -4.49029300

H 0 -1.78725900 -5.80068900 -3.31097500

C 0 -1.60268200 -3.68427800 -3.29713100

O 0 -1.79485700 -2.60233000 -3.86493600

O 0 -1.34937600 -3.85841700 -2.02521900

C -1 5.00053600 -4.68961600 0.94258000

H 0 5.79695900 -5.42629800 1.10073900

H 0 5.26848600 -4.10068700 0.06205600

C 0 4.89181200 -3.79661700 2.17868400

O 0 4.31282900 -4.20371600 3.20897600

C 0 3.66712600 -5.46514800 0.72000100

H 0 3.53521700 -6.15549600 1.56156400

H 0 3.75176800 -6.06852900 -0.18805900

C 0 2.45166600 -4.58574000 0.60825500

N 0 2.19064200 -3.62973700 1.57648100

H 0 2.72073200 -3.55272800 2.44273500

C 0 1.43918600 -4.43287100 -0.31423900

H 0 1.25375900 -4.96108100 -1.23662900

C 0 1.11633100 -2.91557800 1.21841100

H 0 0.79382400 -2.01879500 1.71165900

N 0 0.60846400 -3.39892800 0.09415600

N 0 5.31022500 -2.53005700 2.03480400

H 0 5.86602200 -2.29985300 1.20422800

C -1 4.96113800 -1.46487500 2.97099500

H 0 5.84518800 -0.82716200 3.09859900

H 0 4.73630700 -1.92330200 3.93605000

C 0 3.77161800 -0.62396500 2.47450200

H 0 3.55764300 0.15555600 3.21697000

H 0 2.88254700 -1.25429400 2.42821900

C 0 4.02106100 0.00999100 1.10414500

H 0 4.49137800 -0.71257900 0.42503100

H 0 4.71610000 0.85342300 1.18211700

C 0 2.80023400 0.52721500 0.34643500

O 0 2.99887800 1.14554100 -0.70775300

O 0 1.63408100 0.23473900 0.84396400

C -1 -5.51403000 -4.05220100 2.09296600

H 0 -5.41806000 -3.33430500 2.91465600

H 0 -5.99127400 -4.95191000 2.50058700

C 0 -4.13993600 -4.42310400 1.53492400

H 0 -4.24440500 -5.14050600 0.71585000

H 0 -3.54903100 -4.92496500 2.31089700

C 0 -3.28673700 -3.27541400 0.99796500

O 0 -3.64608000 -2.07845400 1.15973000

O 0 -2.19943700 -3.63825500 0.43863700

C 0 -6.42710000 -3.48459200 1.01053400

O 0 -6.24537900 -3.70858300 -0.19406000

N 0 -7.48643900 -2.77017900 1.45710500

H 0 -7.53275200 -2.52713200 2.43739800

C -1 -8.46989000 -2.17081800 0.56291700

H 0 -9.42909100 -2.11146500 1.07869800

H 0 -8.56649600 -2.81864700 -0.31232900

C 0 -8.07805600 -0.74300800 0.14048200

O 0 -8.66841800 0.23994200 0.59215500

N 0 -7.05179400 -0.67472600 -0.74005800

H 0 -6.57730000 -1.51915300 -1.04421400

C 0 -6.50676300 0.58110400 -1.20741500

H 0 -6.78215000 0.77030000 -2.25016100

H 0 -6.93778700 1.37923400 -0.59427000

C 0 -4.97774900 0.65575100 -1.11594800

O 0 -4.35871000 1.22379900 -2.02889300

O 0 -4.46842100 0.13361200 -0.05076900

C -1 -3.50142800 3.49166700 5.07967500

H -1 -3.54155300 3.16885000 6.10952000

H -1 -4.31414800 4.14557000 4.79990800

H 0 -2.60447600 4.12483200 5.00676100

C 0 -3.40356100 2.38064400 4.02451800

H 0 -3.68569300 2.80544200 3.05297300

H 0 -4.11588100 1.57102200 4.21868800

C 0 -1.98135000 1.82470300 3.90233200

H 0 -1.75003500 1.17613500 4.75815700

H 0 -1.24819100 2.63612000 3.93416200

C 0 -1.73144500 1.06930700 2.60721100

O 0 -0.59067400 1.21523500 2.08284400

O 0 -2.67076700 0.34111200 2.14160800

C -1 -5.54286300 8.98621500 2.22313500

H -1 -5.41550400 8.54991800 3.20284400

H -1 -6.57597800 9.23114200 2.02556000

H 0 -4.98918100 9.93323200 2.21266400

C 0 -4.96589300 8.02362900 1.17514500

H 0 -3.87308500 8.01219500 1.29306200

H 0 -5.15685800 8.39461500 0.15953900

C 0 -5.52802700 6.60109200 1.31616500

H 0 -6.60717000 6.61457900 1.11867700

H 0 -5.39935800 6.25226900 2.35008600

C 0 -4.89080200 5.57448900 0.37514700

H 0 -5.00882100 5.89635400 -0.67039800

H 0 -5.39602100 4.60936800 0.48892600

N 0 -3.47693200 5.38299900 0.69963200

H 0 -2.96383700 6.19385300 1.01731300

C 0 -2.71809500 4.35953100 0.24485800

N 0 -3.24108600 3.42349700 -0.54866500

H 0 -2.75254300 2.52668400 -0.68092800

H 0 -4.09071000 3.56815700 -1.07093300

N 0 -1.44192800 4.27303600 0.61338700

H 0 -1.07274700 4.71304200 1.44877900

H 0 -0.80169400 3.56709100 0.19608700

C -1 -1.08590300 -1.54355700 7.50676500

H -1 -0.09515900 -1.14424300 7.34742600

H -1 -1.27342900 -1.77753200 8.54428700

H 0 -1.82628100 -0.80654000 7.17756800

C 0 -1.26088100 -2.88990000 6.77594900

H 0 -2.22692200 -3.33030400 7.05898500

H 0 -0.49474800 -3.59060900 7.13198500

C 0 -1.19804700 -2.83375900 5.28457900

N 0 -1.04833800 -3.97901000 4.51026800

C 0 -1.31813200 -1.80863200 4.38733800

H 0 -1.45173600 -0.75669900 4.55158200

C 0 -1.07494500 -3.66551200 3.20931700

H 0 -0.97999800 -4.34409600 2.37777800

N 0 -1.24615300 -2.34831800 3.11828100

H 0 -1.24486300 -1.82371200 2.21665700

C -1 3.71606600 6.11793400 2.57355800

H -1 4.58847400 6.62090800 2.18325500

H -1 3.24349200 6.61491800 3.40787300

H 0 2.96643800 6.11595300 1.77406300

C 0 4.07306400 4.64650300 2.89147700

H 0 5.02307500 4.56656700 3.43039700

H 0 3.28253700 4.20651700 3.51097000

C 0 4.11273000 3.84223100 1.58567900

O 0 3.04722200 3.83497600 0.92271500

O 0 5.19303400 3.26577500 1.22476200

C -1 12.07411400 -2.42941500 -0.58108500

H -1 13.12127600 -2.16763100 -0.61745600

H -1 11.85337200 -3.30341200 -1.17589400

H 0 11.85597000 -2.72018200 0.45641500

C 0 11.17666600 -1.23342600 -0.97765400

H 0 11.64665800 -0.67637500 -1.80115300

H 0 10.21575100 -1.60239600 -1.35454000

C 0 10.90346500 -0.28709200 0.20673600

H 0 10.16752300 -0.76366300 0.86901800

H 0 11.82138900 -0.17393500 0.80091600

C 0 10.43966800 1.12979100 -0.17444600

H 0 10.31597000 1.73344300 0.73529800

H 0 11.23555700 1.61309900 -0.75725400

C 0 9.16509700 1.25296200 -1.01201800

H 0 9.03729100 2.29028800 -1.33405700

H 0 9.20481900 0.62623400 -1.90660400

N 0 7.91567200 0.88000600 -0.27554800

H 0 7.09105700 1.30322500 -0.75889000

H 0 7.78502600 -0.15977900 -0.23665500

H 0 7.87216400 1.27167200 0.69521000

O 0 2.00170600 1.75607700 -3.51954200

H 0 2.77753800 2.31717000 -3.74306200

O 0 0.43998900 2.50048500 -0.09662100

O 0 0.73826100 3.83145100 2.52520200

H 0 0.39942600 2.94986900 2.28574000

H 0 1.48325100 3.93510000 1.88611600

O 0 0.52879200 -0.26398600 -4.55090400

H 0 0.93072000 0.63474100 -4.41552600

H 0 1.20513600 -0.81861600 -4.09558200

O 0 -3.70437800 -0.42720600 -4.10187300

H 0 -4.17041100 0.24622800 -3.55991500

H 0 -4.06177700 -1.27891000 -3.78117300

O 0 -4.81835100 -2.32600300 -2.22139000

H 0 -3.96509100 -2.06423300 -1.79627800

H 0 -5.17433600 -3.02654800 -1.64311500

O 0 2.25586600 3.89830100 -1.71364400

H 0 2.62199300 3.85232300 -0.79891600

H 0 2.62235300 -0.95571200 -2.18122300

O 0 0.13329900 -1.81433300 -1.97440600

O 0 5.88072200 2.53941900 -1.26985700

H 0 5.37691300 2.71054900 -0.42201400

H 0 6.36659400 3.37101000 -1.39903800

O 0 4.23369800 3.47311500 -3.44318700

H 0 4.77031800 2.90824700 -2.85056700

O 0 7.36867900 2.07461800 2.20833900

H 0 6.56504500 2.60730400 1.94980100

H 0 7.02867700 1.41058000 2.82752900

O 0 2.83927500 -0.78120600 -3.10906100

H 0 2.67571100 0.20151200 -3.17788200

H 0 0.48126700 -0.90632400 -1.69408600

H 0 -0.93054300 -4.91870400 4.86943000

H 0 3.53129400 3.79129700 -2.80456400

Cl 0 7.48292900 -2.21745900 -0.34075400

**TS_La-Lb_**

E (IEFPCM/B3LYP-D3/SDD,6-31G(d)) = -5854.27537 au

H (IEFPCM/B3LYP-D3/SDD,6-31G(d)) = -5852.729649 au

G (IEFPCM/B3LYP-D3/SDD,6-31G(d)) = -5852.967992 au

E (IEFPCM/B3LYP-D3/SDD,6-311+G(2d,p)//IEFPCM/B3LYP-D3/SDD ,6-31G(d)) = -5856.101971 au

Mn 0 1.17262400 2.24837900 -1.86622900

Mn 0 -1.00297800 -2.41484900 -0.82097100

Mn 0 -0.14633100 0.87657800 0.12250000

Mn 0 -2.65645200 -0.44124200 0.32820700

O 0 0.16312100 0.67527900 -1.59812000

O 0 -1.95791800 1.07262200 -0.25096900

O 0 -2.52755900 -1.39926200 -1.22028400

O 0 -0.91854100 -1.08059400 0.58578400

Ca 0 -1.55568000 -0.16204600 -3.05836700

C -1 0.53110100 4.54857800 -5.62440500

H -1 0.59346800 5.48320000 -5.08671000

H -1 0.72261600 4.62398900 -6.68436700

H 0 1.34569400 3.92985800 -5.22376100

C 0 -0.80223800 3.79774100 -5.39182700

H 0 -0.97652800 3.07750500 -6.19864400

H 0 -1.65258300 4.49428700 -5.42228100

C 0 -0.89187800 3.01314100 -4.06853000

O 0 -1.63123600 1.98215800 -4.04705800

O 0 -0.22719000 3.45098200 -3.08465300

C -1 -2.91923400 -6.71343700 -5.36078500

H -1 -1.95780900 -7.17579600 -5.52898100

H -1 -3.64930300 -6.85713800 -6.14356700

H 0 -3.33210100 -7.21487600 -4.47255000

C 0 -2.73490100 -5.22796100 -5.04136200

H 0 -3.68578800 -4.79560700 -4.70502900

H 0 -2.43614300 -4.66340100 -5.93187000

C 0 -1.68166200 -5.07308800 -3.94314200

H 0 -0.67269400 -5.19741800 -4.36304600

H 0 -1.80055300 -5.85957300 -3.19001400

C 0 -1.71053000 -3.73729300 -3.22817400

O 0 -1.96239700 -2.68225800 -3.82332200

O 0 -1.44913500 -3.86951200 -1.95511200

C -1 5.05599000 -4.64557500 0.98149600

H 0 5.82367000 -5.39672900 1.20078500

H 0 5.37529500 -4.09650400 0.09206600

C 0 4.92145900 -3.70833100 2.17746200

O 0 4.33576100 -4.08136700 3.21607600

C 0 3.71129100 -5.39601500 0.72648400

H 0 3.53735500 -6.06740900 1.57597900

H 0 3.81477900 -6.01985100 -0.16570600

C 0 2.51416900 -4.49844000 0.55999700

N 0 2.24180000 -3.52562700 1.50786600

H 0 2.74949600 -3.43959900 2.38590500

C 0 1.52208400 -4.34777200 -0.38629400

H 0 1.34784300 -4.89353100 -1.30093100

C 0 1.18136100 -2.80776700 1.11686400

H 0 0.85535200 -1.90351400 1.59243500

N 0 0.69299700 -3.29719700 -0.01335900

N 0 5.32750400 -2.44327000 1.98727900

H 0 5.89311500 -2.23762200 1.15731200

C -1 5.01902600 -1.36814600 2.92367500

H 0 5.90179400 -0.71872000 2.98618200

H 0 4.86286800 -1.81531000 3.90791200

C 0 3.79538400 -0.54150300 2.50424100

H 0 3.58986900 0.20506200 3.28162400

H 0 2.91762700 -1.18855200 2.46540900

C 0 3.99971100 0.14111900 1.15259300

H 0 4.56353000 -0.50923700 0.47244900

H 0 4.59964500 1.05323300 1.25404900

C 0 2.75079900 0.52901800 0.37435700

O 0 2.93102800 1.00342300 -0.76608300

O 0 1.60149700 0.28975400 0.90334500

C -1 -5.45188300 -4.01889600 2.19708600

H 0 -5.35029400 -3.27125800 2.99084200

H 0 -5.92071200 -4.90564400 2.64199700

C 0 -4.07907800 -4.40503100 1.63955800

H 0 -4.19120700 -5.13348900 0.83105900

H 0 -3.48903400 -4.89943500 2.42094200

C 0 -3.22408700 -3.26575300 1.08291000

O 0 -3.62597000 -2.07295000 1.21191500

O 0 -2.12446100 -3.60830500 0.55082100

C 0 -6.38252800 -3.50069000 1.10557500

O 0 -6.23289200 -3.79669100 -0.08722100

N 0 -7.42650700 -2.75384600 1.53866100

H 0 -7.44383500 -2.45623500 2.50483900

C -1 -8.42723500 -2.19050000 0.64080800

H 0 -9.39154200 -2.16458500 1.15094100

H 0 -8.49434300 -2.84353000 -0.23287500

C 0 -8.07895500 -0.75177300 0.22466000

O 0 -8.68146500 0.21301600 0.70068800

N 0 -7.07198700 -0.65158400 -0.67254500

H 0 -6.56693700 -1.47599900 -0.98885800

C 0 -6.53263900 0.62255000 -1.09698600

H 0 -6.81914000 0.85337700 -2.12821600

H 0 -6.95075500 1.39650300 -0.44521700

C 0 -5.00167700 0.66640800 -1.01643900

O 0 -4.37464800 1.19752300 -1.94837100

O 0 -4.50296100 0.14923400 0.05363500

C -1 -3.44729600 3.60964200 4.96619700

H -1 -3.47810900 3.31420700 6.00454700

H -1 -4.26484600 4.25267900 4.67553100

H 0 -2.54908900 4.23847900 4.87018600

C 0 -3.36160200 2.48656400 3.93728900

H 0 -3.48174700 2.93314700 2.94133100

H 0 -4.17932600 1.76617300 4.04783800

C 0 -2.01297200 1.76127300 3.98584100

H 0 -2.01097200 1.05595000 4.82542300

H 0 -1.19464000 2.46770500 4.15092000

C 0 -1.73878400 1.04090900 2.68079000

O 0 -0.62277200 1.25226700 2.12946500

O 0 -2.66167700 0.28459200 2.22879100

C -1 -5.53342400 9.01815000 1.98065800

H -1 -5.39668000 8.60859600 2.97059300

H -1 -6.56903900 9.25368600 1.78476000

H 0 -4.98101300 9.96488300 1.93781800

C 0 -4.96758800 8.02588900 0.95515800

H 0 -3.87550600 7.99886000 1.07675600

H 0 -5.14652700 8.37903000 -0.06883700

C 0 -5.55626200 6.61584600 1.11935400

H 0 -6.62476500 6.63498900 0.87273000

H 0 -5.48010200 6.30014200 2.16916300

C 0 -4.88634500 5.55144700 0.24551300

H 0 -4.92799500 5.84996900 -0.81244600

H 0 -5.42088000 4.60041600 0.34948900

N 0 -3.50057800 5.34747000 0.67000900

H 0 -3.03041800 6.14057700 1.08435200

C 0 -2.68760600 4.37404800 0.19810300

N 0 -3.12595100 3.48593500 -0.69740600

H 0 -2.67262300 2.55959300 -0.75265800

H 0 -4.04314400 3.55501700 -1.10923500

N 0 -1.42977000 4.30338400 0.62812400

H 0 -1.11648300 4.75980900 1.47501600

H 0 -0.76016500 3.60029300 0.23894300

C -1 -0.99231200 -1.34967900 7.50729700

H -1 -0.00448600 -0.95087800 7.32961500

H -1 -1.17082800 -1.55667700 8.55211500

H 0 -1.74188000 -0.63262700 7.15641600

C 0 -1.14309600 -2.72024600 6.81298800

H 0 -2.10988200 -3.16215200 7.09111700

H 0 -0.37670200 -3.40215300 7.20413400

C 0 -1.05641500 -2.70781900 5.32062500

N 0 -0.99057800 -3.87866700 4.57224100

C 0 -1.09146500 -1.69886500 4.39911000

H 0 -1.12024300 -0.63715900 4.54374900

C 0 -0.99294000 -3.59218600 3.26376600

H 0 -0.95451000 -4.29318100 2.44622100

N 0 -1.06642900 -2.26913500 3.14147800

H 0 -1.08575500 -1.76508800 2.22264000

C -1 3.73968100 6.19646200 2.33449700

H -1 4.60696000 6.69227400 1.92411400

H -1 3.27154200 6.71363300 3.15898600

H 0 2.98533300 6.15201900 1.54087800

C 0 4.11812600 4.74494000 2.71581400

H 0 5.05675500 4.70908800 3.27904800

H 0 3.32120600 4.31426500 3.33297700

C 0 4.20864100 3.88780800 1.44872100

O 0 3.15682300 3.79266200 0.77382300

O 0 5.32567200 3.35004400 1.12897300

C -1 12.10817800 -2.39909200 -0.65726200

H -1 13.15393800 -2.13427400 -0.70880800

H -1 11.88643700 -3.28947400 -1.22686400

H 0 11.90519400 -2.66898600 0.38930200

C 0 11.20896100 -1.20949900 -1.05598300

H 0 11.63869400 -0.69466900 -1.92757600

H 0 10.22155500 -1.57679400 -1.35881500

C 0 11.02872200 -0.21497800 0.10626800

H 0 10.33209500 -0.65436200 0.83390200

H 0 11.98502500 -0.09705000 0.63521700

C 0 10.55916000 1.19331000 -0.29452900

H 0 10.48998000 1.82668000 0.60060000

H 0 11.32354600 1.65065200 -0.93721700

C 0 9.24037700 1.29214300 -1.06082400

H 0 9.07703300 2.32405100 -1.38440000

H 0 9.24097200 0.65703700 -1.95051000

N 0 8.04094900 0.90378200 -0.25511500

H 0 7.18447800 1.29083000 -0.71870900

H 0 7.93371500 -0.13636500 -0.19162000

H 0 8.02374600 1.32320400 0.70377200

O 0 1.87921100 1.26515800 -3.48890700

H 0 2.76089000 1.65253300 -3.71683800

O 0 0.41896300 2.53005900 -0.09336900

O 0 0.87216800 3.77608000 2.49604500

H 0 0.44240800 2.92574100 2.29351800

H 0 1.55535700 3.83040100 1.78932800

O 0 0.15848800 -0.31346000 -4.78254100

H 0 0.79632600 0.40034400 -4.49125200

H 0 0.62910600 -1.14487200 -4.60541200

O 0 -3.81168600 -0.45691000 -4.04022800

H 0 -4.27787800 0.20468200 -3.48462100

H 0 -4.12575100 -1.31750900 -3.69912200

O 0 -4.90832700 -2.30351700 -2.07992700

H 0 -4.03902500 -2.03997900 -1.68398800

H 0 -5.21777100 -3.03872600 -1.51716000

O 0 2.36081900 3.69450100 -1.88651700

H 0 2.76065500 3.68652600 -0.98617500

H 0 2.62946800 -0.64865300 -1.71003400

O 0 -0.03594400 -1.76258900 -2.08415500

O 0 5.92590700 2.37287600 -1.27853700

H 0 5.50789500 2.68916300 -0.41981100

H 0 6.32162200 3.17914100 -1.64847500

O 0 4.21538200 2.71868700 -3.53150700

H 0 4.72792700 2.27518700 -2.82475500

O 0 7.49653900 2.18593800 2.18956500

H 0 6.69817100 2.70902000 1.90256000

H 0 7.14734200 1.54628500 2.82915200

O 0 2.20924900 -0.98672900 -2.52649900

H 0 2.06655000 0.02700100 -3.00636100

H 0 -0.94421500 -4.81659700 4.95129600

H 0 3.56098000 3.23404200 -2.97266100

Cl 0 7.53395400 -2.18100400 -0.36958700

H 0 1.12568700 -1.36015900 -2.17941500

**Lb_S_4_**

E (IEFPCM/B3LYP-D3/SDD,6-31G(d)) = -5854.294348 au

H (IEFPCM/B3LYP-D3/SDD,6-31G(d)) = -5852.740957 au

G (IEFPCM/B3LYP-D3/SDD,6-31G(d)) = -5852.980819 au

E (IEFPCM/B3LYP-D3/SDD,6-311+G(2d,p)//IEFPCM/B3LYP-D3/SDD,6-31G(d)) = -5856.120116 au

Mn 0 1.11623900 2.40808000 -1.78088000

Mn 0 -1.00256200 -2.47819300 -0.79854200

Mn 0 -0.12238200 0.81003600 0.07022000

Mn 0 -2.66011900 -0.46989500 0.27417300

O 0 0.28721300 0.72389600 -1.65898300

O 0 -1.91306600 1.00552400 -0.36600000

O 0 -2.53238900 -1.45684400 -1.23834500

O 0 -0.93770700 -1.10626000 0.59112700

Ca 0 -1.41046200 -0.29478600 -3.04986400

C -1 0.57170000 4.52065700 -5.65962400

H -1 0.63732100 5.45745800 -5.12612300

H -1 0.76925000 4.58967700 -6.71891400

H 0 1.38214800 3.89862800 -5.25531800

C 0 -0.77258400 3.78914100 -5.42009700

H 0 -1.00568900 3.13010900 -6.26237100

H 0 -1.59645800 4.51339700 -5.35421100

C 0 -0.80130400 2.92967500 -4.14513400

O 0 -1.33682800 1.78557600 -4.22047100

O 0 -0.27884100 3.42607600 -3.09889700

C -1 -2.95307100 -6.71737800 -5.35986400

H -1 -1.99380200 -7.18676100 -5.52075900

H -1 -3.67994800 -6.86015200 -6.14578000

H 0 -3.37255700 -7.21292600 -4.47143000

C 0 -2.74530900 -5.23441800 -5.03801400

H 0 -3.68052900 -4.79262100 -4.67161000

H 0 -2.46449400 -4.66753900 -5.93287200

C 0 -1.65575200 -5.10203700 -3.97008400

H 0 -0.66335800 -5.22501400 -4.42749600

H 0 -1.75643500 -5.90043900 -3.22716800

C 0 -1.65319500 -3.77804700 -3.23235400

O 0 -1.86864500 -2.70881800 -3.82225000

O 0 -1.41193900 -3.92850800 -1.96140200

C -1 5.00208800 -4.67038800 1.01430700

H 0 5.80329400 -5.39659600 1.19664700

H 0 5.26484900 -4.11044200 0.11316500

C 0 4.89470700 -3.73633800 2.21873200

O 0 4.32576000 -4.11027400 3.26591700

C 0 3.67263200 -5.45751300 0.81826900

H 0 3.53558300 -6.10897100 1.68964400

H 0 3.76392000 -6.10151800 -0.06091900

C 0 2.46415900 -4.57800800 0.65726500

N 0 2.20655500 -3.57748800 1.57970300

H 0 2.72102400 -3.46995700 2.45027200

C 0 1.46441900 -4.44990100 -0.28305100

H 0 1.28235000 -5.01717100 -1.18296500

C 0 1.14527100 -2.86570300 1.17564400

H 0 0.82683500 -1.94695400 1.62840500

N 0 0.64447200 -3.38707500 0.06710600

N 0 5.31016500 -2.47369200 2.02927800

H 0 5.85452100 -2.26737600 1.18604600

C -1 4.97624600 -1.38339100 2.94043100

H 0 5.86526300 -0.74876200 3.04808400

H 0 4.75357600 -1.81749300 3.91709000

C 0 3.78794600 -0.54490900 2.43549700

H 0 3.57154400 0.23854500 3.17293400

H 0 2.90003400 -1.17708600 2.39316600

C 0 4.02773800 0.08378900 1.05912400

H 0 4.53117600 -0.62679200 0.39068400

H 0 4.68818900 0.95606400 1.12918200

C 0 2.78729700 0.53518900 0.29206300

O 0 2.96914400 1.09703200 -0.81143900

O 0 1.64072600 0.25044200 0.80330600

C -1 -5.50771500 -3.96981600 2.17146900

H 0 -5.39519900 -3.20933300 2.95165000

H 0 -5.99943700 -4.83625400 2.63230900

C 0 -4.14532800 -4.39176200 1.63163000

H 0 -4.26493200 -5.12320000 0.82698300

H 0 -3.57127900 -4.89143200 2.42172800

C 0 -3.26533400 -3.27451600 1.07732500

O 0 -3.64228200 -2.06926500 1.19925200

O 0 -2.17447400 -3.64873400 0.55919500

C 0 -6.42815400 -3.45280700 1.07063600

O 0 -6.28077600 -3.76384900 -0.11848000

N 0 -7.46486000 -2.68925900 1.49377100

H 0 -7.47882800 -2.37998100 2.45629700

C -1 -8.46293100 -2.12978400 0.59073100

H 0 -9.42537700 -2.08634200 1.10311900

H 0 -8.53942700 -2.79600900 -0.27240800

C 0 -8.10530700 -0.70194900 0.14588100

O 0 -8.72377700 0.27387000 0.57785900

N 0 -7.07569800 -0.62303500 -0.72679500

H 0 -6.56392500 -1.45422300 -1.01301200

C 0 -6.53108900 0.64022400 -1.17409700

H 0 -6.80777500 0.84936900 -2.21279700

H 0 -6.95666800 1.42992800 -0.54607500

C 0 -5.00080500 0.69524700 -1.08054700

O 0 -4.38078200 1.27804100 -1.98705400

O 0 -4.50116900 0.14163300 -0.03115300

C -1 -3.46813600 3.65895200 4.91427200

H -1 -3.50630800 3.36877000 5.95385800

H -1 -4.27995800 4.30584700 4.61620300

H 0 -2.56594400 4.28178600 4.81682500

C 0 -3.38564600 2.51729700 3.89843600

H 0 -3.58788300 2.93237400 2.90355100

H 0 -4.15654300 1.75916800 4.07533400

C 0 -1.99868600 1.87149600 3.87055000

H 0 -1.86956600 1.22583900 4.74924600

H 0 -1.21761800 2.63450200 3.92862400

C 0 -1.75042800 1.07793600 2.59942800

O 0 -0.59902700 1.17572100 2.08463700

O 0 -2.70621000 0.37095500 2.14352200

C -1 -5.50341100 9.06628000 1.89172400

H -1 -5.37451900 8.66066300 2.88433100

H -1 -6.53643300 9.30755800 1.68923800

H 0 -4.93879900 10.00502400 1.84030000

C 0 -4.94216000 8.04739300 0.88722400

H 0 -3.85510700 7.98108600 1.03672500

H 0 -5.08123900 8.39605700 -0.14433300

C 0 -5.58444100 6.65903900 1.04531100

H 0 -6.63228200 6.70312100 0.72514400

H 0 -5.59392800 6.36830900 2.10530200

C 0 -4.89125300 5.54916400 0.24891900

H 0 -4.80028000 5.84183100 -0.80712300

H 0 -5.49210700 4.63335700 0.29505300

N 0 -3.57115300 5.26233900 0.81628100

H 0 -3.13475200 5.99421900 1.35970200

C 0 -2.75639400 4.27041000 0.39183600

N 0 -3.13288900 3.44549400 -0.58807900

H 0 -2.67411900 2.52273800 -0.67350000

H 0 -4.02682700 3.53475000 -1.04454900

N 0 -1.56879900 4.08481800 0.96797300

H 0 -1.23823600 4.64505700 1.74099600

H 0 -0.85324800 3.47136400 0.52032500

C -1 -1.05877300 -1.30375100 7.49216800

H -1 -0.06745900 -0.91221900 7.31777300

H -1 -1.24410000 -1.50451100 8.53701800

H 0 -1.80044300 -0.58068500 7.13615000

C 0 -1.22416200 -2.67427400 6.80382200

H 0 -2.19488200 -3.10505000 7.08596200

H 0 -0.46408500 -3.36260600 7.19606400

C 0 -1.13816900 -2.66722200 5.31209100

N 0 -1.04789700 -3.84163000 4.57261900

C 0 -1.18842300 -1.66489300 4.38352300

H 0 -1.24545800 -0.60270100 4.51970400

C 0 -1.04769600 -3.56358900 3.26234700

H 0 -0.99373000 -4.26944200 2.44995200

N 0 -1.14312000 -2.24311200 3.13020500

H 0 -1.14200500 -1.74548400 2.20337600

C -1 3.74918300 6.18638700 2.30803300

H -1 4.62180300 6.67457600 1.89983900

H -1 3.28010300 6.71057600 3.12754000

H 0 2.99829100 6.11467700 1.51342000

C 0 4.13883000 4.75231800 2.75125000

H 0 5.08113500 4.75368900 3.30927500

H 0 3.35000000 4.34586800 3.39423100

C 0 4.22792100 3.84276100 1.52568400

O 0 3.15424100 3.64928100 0.90682100

O 0 5.36206900 3.36830300 1.17217100

C -1 12.07721100 -2.47758200 -0.59808300

H -1 13.12492600 -2.21979100 -0.64539500

H -1 11.85267600 -3.36926700 -1.16454400

H 0 11.86308500 -2.73642400 0.44882300

C 0 11.18478700 -1.28995600 -1.02208800

H 0 11.63166400 -0.78277200 -1.88957600

H 0 10.20418700 -1.66081200 -1.34245000

C 0 10.97619300 -0.28303700 0.12458400

H 0 10.25932000 -0.71215100 0.83840200

H 0 11.91855000 -0.16023300 0.67693800

C 0 10.52178900 1.12207600 -0.30540700

H 0 10.43813200 1.76833300 0.57917400

H 0 11.30268300 1.56575900 -0.93776800

C 0 9.22036500 1.22254900 -1.10223200

H 0 9.08224100 2.24957000 -1.45187300

H 0 9.22851800 0.56761200 -1.97724700

N 0 7.99758400 0.87328700 -0.31133600

H 0 7.16046500 1.29052600 -0.77515900

H 0 7.85898600 -0.16504300 -0.24868800

H 0 7.99054000 1.28861800 0.65053100

O 0 2.05755200 1.67945000 -3.54839700

H 0 2.94431800 2.16711100 -3.59947200

O 0 0.35630100 2.51512000 -0.04721900

O 0 0.89157000 3.64078800 2.59972800

H 0 0.47701000 2.79120500 2.36202800

H 0 1.59233700 3.72071200 1.91254900

O 0 0.41734100 -0.45520700 -4.72214600

H 0 0.60771500 0.49704400 -4.81123800

H 0 1.17806200 -0.79958000 -4.21265900

O 0 -3.68671200 -0.43844100 -4.01378400

H 0 -4.13775600 0.23641200 -3.46041700

H 0 -4.04066900 -1.28938700 -3.68902200

O 0 -4.91213200 -2.30721700 -2.11789900

H 0 -4.04365700 -2.05870600 -1.70708300

H 0 -5.24189900 -3.03613600 -1.55903000

O 0 2.18998900 3.94059800 -1.66362100

H 0 2.60050200 3.86484100 -0.77076600

H 0 2.78427000 -0.38096300 -1.95591000

O 0 -0.00060400 -1.79153000 -1.95310200

O 0 5.96561700 2.55161600 -1.27451800

H 0 5.54255500 2.78907300 -0.39038800

H 0 6.48353600 3.34560600 -1.48909900

O 0 4.14504900 3.23545400 -3.30789600

H 0 4.73529200 2.79379400 -2.65601000

O 0 7.54265700 2.14720900 2.14979400

H 0 6.74457600 2.68763000 1.90032600

H 0 7.20741100 1.50470600 2.79403800

O 0 2.43852300 -0.99023000 -2.64125700

H 0 2.25556000 0.74393700 -3.30360900

H 0 -0.98712900 -4.77591000 4.95836900

H 0 3.47206900 3.67720600 -2.70380400

Cl 0 7.47642900 -2.20279800 -0.37345600

H 0 1.56161600 -1.26774100 -2.24929400

**TS_Lb-Lc_**

E (IEFPCM/B3LYP-D3/SDD,6-31G(d)) = -5854.286702 au

H (IEFPCM/B3LYP-D3/SDD,6-31G(d)) = -5852.740298 au

G (IEFPCM/B3LYP-D3/SDD,6-31G(d)) = -5852.977932 au

E (IEFPCM/B3LYP-D3/SDD,6-311+G(2d,p)//IEFPCM/B3LYP-D3/SDD,6-31G(d)) = -5856.111233 au

Mn 0 1.15148200 2.38555100 -1.79000900

Mn 0 -1.01760500 -2.49205500 -0.80354900

Mn 0 -0.10473200 0.79704800 0.05643400

Mn 0 -2.65786100 -0.46859600 0.24910400

O 0 0.34792600 0.70411300 -1.66671300

O 0 -1.88848500 0.99065100 -0.41410900

O 0 -2.53601700 -1.46273900 -1.25410500

O 0 -0.94377500 -1.11304600 0.58413800

Ca 0 -1.37238300 -0.27240100 -3.05583400

C -1 0.58476600 4.54901200 -5.62407800

H -1 0.65147600 5.48265500 -5.08526200

H -1 0.78441800 4.62350400 -6.68265500

H 0 1.39247300 3.92376400 -5.21911200

C 0 -0.76027100 3.81953500 -5.38893900

H 0 -0.98849000 3.15612500 -6.22909300

H 0 -1.58537200 4.54341800 -5.33193600

C 0 -0.79608400 2.96544300 -4.11054600

O 0 -1.35510000 1.83340700 -4.17648500

O 0 -0.25508600 3.45425100 -3.06866200

C -1 -2.96353800 -6.68314800 -5.39331400

H -1 -2.00509500 -7.15366300 -5.55520600

H -1 -3.68947300 -6.82005300 -6.18132200

H 0 -3.38525500 -7.18141700 -4.50752800

C 0 -2.74805000 -5.20336800 -5.06441200

H 0 -3.67806300 -4.75928800 -4.68773900

H 0 -2.47128300 -4.63200900 -5.95765400

C 0 -1.64830100 -5.08363300 -4.00470500

H 0 -0.66044100 -5.19975600 -4.47322000

H 0 -1.74227400 -5.89017500 -3.26986900

C 0 -1.64476500 -3.76678000 -3.25579500

O 0 -1.84433000 -2.69271500 -3.84243600

O 0 -1.42663700 -3.92907000 -1.98152700

C -1 4.98435600 -4.68827600 1.00621500

H 0 5.78812100 -5.41349300 1.18102100

H 0 5.24095900 -4.12314700 0.10661800

C 0 4.87964600 -3.76081000 2.21601000

O 0 4.31163200 -4.14002900 3.26173700

C 0 3.65523200 -5.47734100 0.81466000

H 0 3.52263600 -6.12941800 1.68623200

H 0 3.74379700 -6.12054500 -0.06539700

C 0 2.44545200 -4.59868300 0.65750900

N 0 2.19535200 -3.58947900 1.57255800

H 0 2.71077100 -3.47879100 2.44180900

C 0 1.44059700 -4.47639500 -0.27795800

H 0 1.25434400 -5.04896600 -1.17372100

C 0 1.13429900 -2.87764500 1.16764700

H 0 0.81885300 -1.95543900 1.61594400

N 0 0.62645100 -3.40701200 0.06599200

N 0 5.29434400 -2.49691600 2.03267700

H 0 5.83791100 -2.28644500 1.19003900

C -1 4.96182100 -1.41216900 2.95061500

H 0 5.85396500 -0.78432600 3.07104100

H 0 4.73013300 -1.85392000 3.92175300

C 0 3.78247900 -0.56182500 2.44652200

H 0 3.56634600 0.21636200 3.18964500

H 0 2.89062700 -1.18748700 2.39192900

C 0 4.03533700 0.07616800 1.07741800

H 0 4.53546500 -0.63391900 0.40611100

H 0 4.70273600 0.94252000 1.15615600

C 0 2.80289300 0.53981500 0.30639900

O 0 2.99490500 1.11591400 -0.78706000

O 0 1.65132800 0.24796900 0.80552800

C -1 -5.52594300 -3.97239600 2.14861300

H 0 -5.41586200 -3.21694800 2.93415100

H 0 -6.01857700 -4.84171500 2.60306400

C 0 -4.16284800 -4.39020200 1.61059000

H 0 -4.27987000 -5.11770400 0.80203500

H 0 -3.59025300 -4.89339100 2.39958500

C 0 -3.28006900 -3.27226400 1.06338400

O 0 -3.64808700 -2.06472700 1.18999600

O 0 -2.19182400 -3.65296400 0.54392700

C 0 -6.44497800 -3.44848500 1.04993200

O 0 -6.29761900 -3.75350500 -0.14068800

N 0 -7.48023700 -2.68430800 1.47572000

H 0 -7.49315800 -2.37708600 2.43888300

C -1 -8.47462600 -2.11749900 0.57300700

H 0 -9.43420600 -2.05863300 1.08894700

H 0 -8.56316800 -2.78726900 -0.28626500

C 0 -8.10122000 -0.69610700 0.11757100

O 0 -8.70919300 0.28894000 0.54351300

N 0 -7.07149400 -0.63348100 -0.75686600

H 0 -6.57079600 -1.47223700 -1.03974400

C 0 -6.52124200 0.62050400 -1.22311800

H 0 -6.78917800 0.80912600 -2.26801300

H 0 -6.95296500 1.42177500 -0.61442100

C 0 -4.99162400 0.68261100 -1.11911500

O 0 -4.36677200 1.26167900 -2.02425600

O 0 -4.49496500 0.14152200 -0.06158300

C -1 -3.47563600 3.63669200 4.93769000

H -1 -3.51619800 3.34079000 5.97552900

H -1 -4.28551400 4.28692300 4.64177600

H 0 -2.57304200 4.25954800 4.84521000

C 0 -3.39285200 2.49760500 3.91562300

H 0 -3.64211500 2.90546700 2.92848000

H 0 -4.13365000 1.71607400 4.11760000

C 0 -1.98808100 1.89718100 3.83663000

H 0 -1.79428900 1.26852400 4.71616600

H 0 -1.23226100 2.68718700 3.85580000

C 0 -1.75152200 1.09511000 2.56796600

O 0 -0.59540800 1.17391900 2.05813700

O 0 -2.71312000 0.39993800 2.10900200

C -1 -5.49430000 9.06505400 1.94176800

H -1 -5.36801000 8.65357900 2.93230400

H -1 -6.52644600 9.30955800 1.73879400

H 0 -4.92815100 10.00319700 1.89685200

C 0 -4.93043500 8.05078300 0.93276100

H 0 -3.84263400 7.99039800 1.08007700

H 0 -5.07381400 8.40181900 -0.09742900

C 0 -5.56379900 6.65777100 1.08806300

H 0 -6.61605000 6.70013300 0.78215000

H 0 -5.55757200 6.35887300 2.14571000

C 0 -4.87847200 5.55554600 0.27310000

H 0 -4.80520400 5.85597100 -0.78226900

H 0 -5.47556200 4.63768500 0.32208800

N 0 -3.54938200 5.26608000 0.81794100

H 0 -3.09259800 6.00730400 1.33137500

C 0 -2.74872100 4.26302500 0.39083500

N 0 -3.14582100 3.42584300 -0.56887500

H 0 -2.67736900 2.50991400 -0.67352500

H 0 -4.02498700 3.53585200 -1.04863800

N 0 -1.55240800 4.07864100 0.94931400

H 0 -1.21835800 4.62137200 1.73331700

H 0 -0.84556600 3.45963200 0.49799500

C -1 -1.08107800 -1.34534700 7.49204200

H -1 -0.08864100 -0.95487000 7.32157200

H -1 -1.26864800 -1.55155400 8.53541600

H 0 -1.81886400 -0.61498300 7.14293900

C 0 -1.25489900 -2.70691100 6.78948600

H 0 -2.22854000 -3.13426600 7.06674200

H 0 -0.49960100 -3.40442200 7.17445700

C 0 -1.16669700 -2.68552900 5.29765300

N 0 -1.04785900 -3.85326200 4.55175100

C 0 -1.23660300 -1.67890600 4.37442500

H 0 -1.32484700 -0.61901000 4.51494600

C 0 -1.04830800 -3.56798400 3.24318100

H 0 -0.97707700 -4.26838100 2.42747000

N 0 -1.17128600 -2.24906900 3.11800800

H 0 -1.16673800 -1.74744800 2.19313400

C -1 3.75154600 6.16357300 2.35820400

H -1 4.62591900 6.65224800 1.95435100

H -1 3.28218400 6.68418000 3.17996300

H 0 3.00048700 6.10419800 1.56252100

C 0 4.13771300 4.72258200 2.78221600

H 0 5.08341700 4.71281000 3.33417100

H 0 3.35049900 4.30878700 3.42223200

C 0 4.21575800 3.83735100 1.53903300

O 0 3.12837400 3.64340900 0.94095900

O 0 5.34490600 3.38842000 1.14589600

C -1 12.06681600 -2.50131800 -0.58144700

H -1 13.11516100 -2.24534600 -0.62545300

H -1 11.84151400 -3.38933100 -1.15321400

H 0 11.84572900 -2.76060800 0.46361500

C 0 11.17472600 -1.31484500 -1.01683200

H 0 11.64168300 -0.79544100 -1.86634300

H 0 10.20799800 -1.69199200 -1.37022700

C 0 10.91771200 -0.31837100 0.12923300

H 0 10.18274200 -0.76026700 0.81616000

H 0 11.84058700 -0.18782800 0.71195700

C 0 10.46303900 1.08547200 -0.30786100

H 0 10.35294600 1.72834200 0.57629300

H 0 11.25765700 1.53587300 -0.91802000

C 0 9.18166400 1.18607700 -1.13864400

H 0 9.06237600 2.20984300 -1.50418100

H 0 9.20439800 0.51968500 -2.00464000

N 0 7.93699700 0.85955300 -0.37079100

H 0 7.11107800 1.27465800 -0.85112400

H 0 7.79258100 -0.17819600 -0.29562100

H 0 7.92177200 1.28507800 0.58750500

O 0 2.16882700 1.83693600 -3.48048500

H 0 3.14562200 2.57522200 -3.46393800

O 0 0.36932500 2.50462200 -0.07442700

O 0 0.87460200 3.61350000 2.62010900

H 0 0.47378700 2.76033600 2.37033700

H 0 1.58219800 3.70887800 1.94197900

O 0 0.61379700 -0.32276500 -4.52275800

H 0 0.85669300 0.62525600 -4.50822000

H 0 1.29738300 -0.72262900 -3.93617000

O 0 -3.63606900 -0.43476400 -4.05055900

H 0 -4.09794900 0.22920800 -3.49289300

H 0 -3.97630500 -1.29414100 -3.73554000

O 0 -4.90616700 -2.32338700 -2.15174100

H 0 -4.04162200 -2.06650200 -1.73850900

H 0 -5.23506000 -3.04642200 -1.58516000

O 0 2.23123500 3.99239100 -1.58836900

H 0 2.60593400 3.89010100 -0.67781000

H 0 2.68228400 -0.41485700 -1.91439600

O 0 -0.00926500 -1.80039500 -1.94447000

O 0 5.90980400 2.60122200 -1.33626200

H 0 5.49795500 2.81433800 -0.44258200

H 0 6.47467500 3.37496100 -1.50263000

O 0 3.95894300 3.42842100 -3.19089300

H 0 4.63331700 2.98538900 -2.62070900

O 0 7.53126000 2.15173400 2.08410600

H 0 6.73168800 2.69784800 1.85270900

H 0 7.20865300 1.51450100 2.73995800

O 0 2.47429700 -1.16424000 -2.50616600

H 0 2.51317000 0.95352200 -3.25780700

H 0 -0.96838900 -4.78811200 4.93273800

H 0 3.21494100 3.83479400 -2.40632700

Cl 0 7.46570300 -2.21927900 -0.36179100

H 0 1.58606400 -1.45726300 -2.15392600

**Lc_S_4_**

E (IEFPCM/B3LYP-D3/SDD,6-31G(d)) = -5854.29558 au

H (IEFPCM/B3LYP-D3/SDD,6-31G(d)) = -5852.74154 au

G (IEFPCM/B3LYP-D3/SDD,6-31G(d)) = -5852.98382 au

E (IEFPCM/B3LYP-D3/SDD,6-311+G(2d,p)//IEFPCM/B3LYP-D3/SDD,6-31G(d)) = -5856.12109 au

Mn 0 1.23578800 2.27744800 -1.76927100

Mn 0 -0.91508900 -2.48638600 -0.76594500

Mn 0 -0.09498800 0.82543400 0.13789600

Mn 0 -2.62156500 -0.47251300 0.22224500

O 0 0.40588000 0.62571900 -1.55544600

O 0 -1.86887300 1.01092500 -0.38546400

O 0 -2.40995500 -1.45771400 -1.28198600

O 0 -0.91022500 -1.09564000 0.62773400

Ca 0 -1.18184000 -0.32819900 -3.08165200

C -1 0.67355800 4.56076400 -5.59342300

H -1 0.73739600 5.49509800 -5.05540100

H -1 0.88357500 4.63310600 -6.65009000

H 0 1.49025200 3.94690300 -5.18717000

C 0 -0.65388600 3.79920600 -5.35390100

H 0 -0.84952600 3.10803500 -6.18000300

H 0 -1.50274300 4.49745000 -5.32912700

C 0 -0.68874100 2.97280300 -4.05241100

O 0 -1.27878800 1.85456600 -4.07482200

O 0 -0.12051700 3.47697100 -3.03382200

C -1 -2.90654100 -6.66173600 -5.37976900

H -1 -1.94767700 -7.13496200 -5.53156500

H -1 -3.62500200 -6.79794800 -6.17453800

H 0 -3.33848400 -7.16986900 -4.50400200

C 0 -2.68062100 -5.18892700 -5.01933400

H 0 -3.60425400 -4.74080300 -4.62947300

H 0 -2.39484700 -4.60100800 -5.89888500

C 0 -1.57734000 -5.10990700 -3.95768400

H 0 -0.59832500 -5.28727700 -4.42603800

H 0 -1.71598800 -5.90004600 -3.21201600

C 0 -1.49677300 -3.78403000 -3.22693400

O 0 -1.62257800 -2.71027400 -3.83740200

O 0 -1.29561800 -3.93512400 -1.95125000

C -1 4.98432700 -4.67764700 1.09343700

H 0 5.81651500 -5.37852700 1.23428800

H 0 5.16924500 -4.11438900 0.17559700

C 0 4.92175100 -3.74998200 2.30319800

O 0 4.47097000 -4.15328800 3.39301100

C 0 3.66961400 -5.50248800 0.98090400

H 0 3.57560300 -6.11999500 1.88207600

H 0 3.73764700 -6.17741700 0.12311200

C 0 2.45542100 -4.63383800 0.82445700

N 0 2.22562600 -3.59558000 1.71334600

H 0 2.74159300 -3.46244500 2.57636300

C 0 1.45663200 -4.51394700 -0.11620700

H 0 1.26259600 -5.10380400 -0.99884500

C 0 1.17979700 -2.87080200 1.28399500

H 0 0.88678000 -1.92580600 1.70009400

N 0 0.66548000 -3.42000000 0.19617000

N 0 5.26157200 -2.46574300 2.08259700

H 0 5.55408800 -2.21257700 1.13547100

C -1 4.95162800 -1.39831700 3.03248200

H 0 5.84559700 -0.76995700 3.14663900

H 0 4.75196200 -1.86945200 3.99636200

C 0 3.76365600 -0.53670500 2.57824700

H 0 3.55133200 0.20820100 3.35623600

H 0 2.87326900 -1.16199700 2.50355900

C 0 4.01573400 0.15480500 1.23721600

H 0 4.51844700 -0.51973000 0.53411500

H 0 4.68575800 1.01314900 1.35191100

C 0 2.79650400 0.64751000 0.46889100

O 0 3.01229000 1.29458900 -0.56842600

O 0 1.63481800 0.30961300 0.93482000

C -1 -5.53470500 -3.93246500 2.13289400

H 0 -5.41420000 -3.17620400 2.91601200

H 0 -6.04447900 -4.79199400 2.58670600

C 0 -4.17568300 -4.37395800 1.59675000

H 0 -4.30420500 -5.10661900 0.79452500

H 0 -3.61336700 -4.88110600 2.39032500

C 0 -3.27381600 -3.27193700 1.03978000

O 0 -3.63546300 -2.06151200 1.14698800

O 0 -2.18342400 -3.66474800 0.53593600

C 0 -6.43561200 -3.39193100 1.02697400

O 0 -6.26905600 -3.67654900 -0.16730700

N 0 -7.47676600 -2.63224000 1.44255500

H 0 -7.50774500 -2.33369300 2.40784500

C -1 -8.46300800 -2.07227300 0.52587900

H 0 -9.40589300 -1.94089500 1.05822800

H 0 -8.60342200 -2.78439200 -0.29211600

C 0 -8.04490800 -0.69832100 -0.02731900

O 0 -8.62936200 0.33144600 0.31686800

N 0 -7.01300100 -0.73314300 -0.90202600

H 0 -6.54040100 -1.61042700 -1.09474300

C 0 -6.43529600 0.45911200 -1.47845400

H 0 -6.60421600 0.49322200 -2.55976100

H 0 -6.94220600 1.32180600 -1.03468600

C 0 -4.92350400 0.60642800 -1.25388500

O 0 -4.27950600 1.23053500 -2.11491700

O 0 -4.46620800 0.09027400 -0.16774400

C -1 -3.49136700 3.67559500 4.92988300

H -1 -3.54281900 3.38142800 5.96777500

H -1 -4.29670400 4.32747800 4.62516000

H 0 -2.58008500 4.28604600 4.84128900

C 0 -3.42785100 2.55256400 3.89071000

H 0 -3.54577500 3.01158000 2.90065200

H 0 -4.26273700 1.85170700 4.00096100

C 0 -2.09688100 1.79509900 3.91604200

H 0 -2.10601300 1.07523200 4.74341200

H 0 -1.26438200 2.48174100 4.09338500

C 0 -1.81947100 1.07393200 2.60625200

O 0 -0.66231400 1.22535700 2.11208600

O 0 -2.75294200 0.37251900 2.10368300

C -1 -5.46668600 9.10448600 1.90620900

H -1 -5.35108600 8.69429000 2.89857100

H -1 -6.49618800 9.35141400 1.69287900

H 0 -4.89804100 10.04141000 1.86660100

C 0 -4.89747400 8.09190800 0.89764400

H 0 -3.81148400 8.02731000 1.05641100

H 0 -5.02892700 8.45127600 -0.13136300

C 0 -5.53758900 6.69983500 1.03542100

H 0 -6.59212000 6.75429400 0.73893600

H 0 -5.52327400 6.38436400 2.08802300

C 0 -4.86668000 5.60561500 0.19587100

H 0 -4.81654200 5.91556600 -0.85835500

H 0 -5.46063400 4.68629200 0.24894800

N 0 -3.52773900 5.31470200 0.71288400

H 0 -3.04637300 6.07391800 1.17526500

C 0 -2.73422400 4.30383200 0.28436600

N 0 -3.14601200 3.44376800 -0.64920500

H 0 -2.68237600 2.52388900 -0.73574800

H 0 -4.01979200 3.55663500 -1.13821500

N 0 -1.52232500 4.15008900 0.81314500

H 0 -1.21082400 4.64710200 1.63627400

H 0 -0.82796100 3.49017000 0.39274800

C -1 -1.13481900 -1.30864700 7.51503200

H -1 -0.13976200 -0.92107000 7.35360400

H -1 -1.33305900 -1.51272500 8.55686400

H 0 -1.86788000 -0.57953700 7.15373800

C 0 -1.29537300 -2.67090100 6.81166600

H 0 -2.27357300 -3.09849200 7.07172100

H 0 -0.54687200 -3.36831700 7.21079700

C 0 -1.18524000 -2.65190600 5.32120500

N 0 -1.18759900 -3.82435700 4.57338200

C 0 -1.13197400 -1.64258300 4.39943200

H 0 -1.07742200 -0.58049900 4.54219000

C 0 -1.14609300 -3.53754600 3.26543700

H 0 -1.13907700 -4.23830900 2.44653700

N 0 -1.12756200 -2.21356900 3.14133600

H 0 -1.09990000 -1.71233000 2.20988700

C -1 3.76705100 6.17960700 2.41683700

H -1 4.64655500 6.66533400 2.02065900

H -1 3.29103200 6.70269800 3.23303600

H 0 3.02256100 6.14434600 1.61464000

C 0 4.11328400 4.72643400 2.81791800

H 0 5.04994000 4.67332400 3.38214300

H 0 3.30352000 4.31366000 3.42989800

C 0 4.18600400 3.89821900 1.54015200

O 0 3.10992200 3.82867700 0.89321600

O 0 5.29451000 3.39458800 1.16426300

C -1 12.08759500 -2.51157700 -0.42882400

H -1 13.13696900 -2.25851300 -0.46307400

H -1 11.86544300 -3.39992000 -1.00144200

H 0 11.85671700 -2.76711100 0.61467500

C 0 11.19383100 -1.32866100 -0.89174900

H 0 11.76292600 -0.68435100 -1.57621900

H 0 10.34791600 -1.71258100 -1.47530300

C 0 10.65193600 -0.47906100 0.27476100

H 0 9.86483200 -1.04194200 0.79674000

H 0 11.45412200 -0.33081500 1.01089900

C 0 10.14379700 0.91797200 -0.12282700

H 0 9.89143300 1.49144400 0.77911300

H 0 10.97000100 1.45670600 -0.60578000

C 0 8.95616200 0.99865300 -1.08889900

H 0 8.89559800 2.01092100 -1.49696300

H 0 9.05749800 0.30914000 -1.93015800

N 0 7.62214700 0.73018800 -0.44946700

H 0 6.89882500 1.33689400 -0.90028600

H 0 7.28724200 -0.25222900 -0.56296400

H 0 7.59282900 1.00576500 0.55986900

Cl 0 6.06893100 -1.93330400 -1.11048700

O 0 2.18600400 1.63828000 -3.32416600

H 0 3.47471100 2.69462800 -3.56626900

O 0 0.38348300 2.52281700 -0.07803000

O 0 0.83508700 3.67656300 2.60870000

H 0 0.42145500 2.82448500 2.37645800

H 0 1.51482800 3.77613700 1.90607200

O 0 0.76244200 -0.30510000 -4.54768100

H 0 1.18198000 0.55469100 -4.25600900

H 0 1.38764600 -0.96004600 -4.18395400

O 0 -3.43872500 -0.47152800 -4.09879500

H 0 -3.89983700 0.20167800 -3.54985300

H 0 -3.79148000 -1.32605600 -3.78710500

O 0 -4.67571900 -2.50912500 -2.25422800

H 0 -3.84591700 -2.18600800 -1.81822200

H 0 -5.07115800 -3.11341000 -1.59941800

O 0 2.33707400 4.01113600 -1.58328900

H 0 2.75803700 3.89953300 -0.67850700

H 0 3.47735700 -1.28428900 -2.00770700

O 0 0.16802100 -1.84118700 -1.85971900

O 0 5.88393000 2.73590900 -1.32854300

H 0 5.43032600 2.89444400 -0.44482300

H 0 6.46880800 3.50773600 -1.41211900

O 0 4.15462800 3.41858700 -3.43339100

H 0 4.77212600 3.03299600 -2.77187600

H 0 3.07030000 3.95854200 -2.26691900

O 0 7.29589700 1.86335600 2.09661700

H 0 6.56858300 2.50580500 1.87690800

H 0 6.93341600 1.31142100 2.80606000

O 0 2.72136900 -1.13226100 -2.60169500

H 0 2.59385800 0.81141000 -2.97740900

H 0 1.90203000 -1.23022200 -2.05889200

H 0 -1.21452700 -4.76268900 4.95298000
